# Metazoans evolved by taking domains from soluble proteins to expand intercellular communication network

**DOI:** 10.1038/srep09576

**Published:** 2015-04-29

**Authors:** Hyun-Jun Nam, Inhae Kim, James U. Bowie, Sanguk Kim

**Affiliations:** 1 School of Interdisciplinary Bioscience and Bioengineering, Pohang University of Science and Technology, Pohang, 790-784, Korea; 2 Department of Life Sciences, Pohang University of Science and Technology, Pohang, 790-784, Korea; 3 Department of Chemistry and Biochemistry, UCLA-DOE Institute of Genomics and Proteomics, Molecular Biology Institute, University of California, Los Angeles, Los Angeles, California 90095-1570, United States

## Abstract

A central question in animal evolution is how multicellular animals evolved from unicellular ancestors. We hypothesize that membrane proteins must be key players in the development of multicellularity because they are well positioned to form the cell-cell contacts and to provide the intercellular communication required for the creation of complex organisms. Here we find that a major mechanism for the necessary increase in membrane protein complexity in the transition from non-metazoan to metazoan life was the new incorporation of domains from soluble proteins. The membrane proteins that have incorporated soluble domains in metazoans are enriched in many of the functions unique to multicellular organisms such as cell-cell adhesion, signaling, immune defense and developmental processes. They also show enhanced protein-protein interaction (PPI) network complexity and centrality, suggesting an important role in the cellular diversification found in complex organisms. Our results expose an evolutionary mechanism that contributed to the development of higher life forms.

Transition from non-metazoa to multicellular animals is a pivotal event in the history of life. The evolution of multicellularity requires the development of stable cell adhesion and communication^1^,^2^ and the division of labor among different cell types^3^,^4^. These developments enable enormous functional innovation, such as the immune system, the nerve system, and complex developmental processes^5^,^6^. Indeed, comparative genomic analysis reveals dramatic increases in cell-adhesion receptors and extracellular matrix (ECM) associated proteins during metazoan genomic evolution^7^. Nevertheless, the evolutionary mechanisms that led to the rapid emergence of the genes required for the development of complex cellular interactions remain poorly understood.

The evolution of membrane proteins is an obvious place to look for mechanistic basis of the diversification that seen in the transition to multicellular life as they are directly positioned to interaction with other cells^8^. Yet membrane protein evolution is known to be constrained in several ways. In particular, the hydrophobic environment imposed by lipid bilayers restricts the amino acid composition and structural diversity of membrane proteins^9^ and the rate of divergence is constrained by the high level side chain burial in the transmembrane regions^10^. Furthermore, domain recombination, a major mechanism of soluble protein diversification^11^,^12^, is not common for the transmembrane domains of membrane proteins^13^. How then did membrane proteins undergo the revolution in functional diversification required for the evolution of multicellular organisms?

Recently, we discovered membrane proteins do employ recombination as a major mechanism of diversification, but rather than exchanging parts between membrane proteins, they efficiently exchange domains with soluble proteins^14^. Thus, we reasoned that domain exchanges between membrane and soluble proteins at the extra-membrane region may have been a key factor in metazoan evolution.

Here, we examine the functional expansion of membrane proteins during the evolution of metazoan species. We found that membrane proteins frequently recruit domains from soluble proteins in metazoan species. Moreover, newly incorporated soluble domains became particularly important players in intercellular PPI network. Especially, they are enriched in functions critical for multicellularity, such as cell-adhesion, immune and developmental processes. Our results suggest that domain sharing between membrane and soluble proteins was a major mechanism for generating the panoply of proteins required for cellular cooperation in metazoans.

## Results

### Domain sharing between membrane and soluble proteins

To investigate functional expansion of membrane proteins during evolution, we identified (i) “*membrane protein domains*” that are found in membrane proteins, (ii) “*soluble protein domains*” that are found in soluble proteins (iii) “*shared domains*” that are found in both membrane and soluble proteins from each non-metazoan and metazoan genomes. We first classified membrane and soluble proteins from complete genomes of 5 non-metazoan and 5 metazoan species using the UniProt database^14^, and assigned domains into membrane and soluble proteins by using profile-HMMs (HMMERs) of Pfam database (see methods and materials). Table 1 shows the numbers of shared, membrane, and soluble protein domains of non-metazoan and metazoan species.

**Figure 1:**
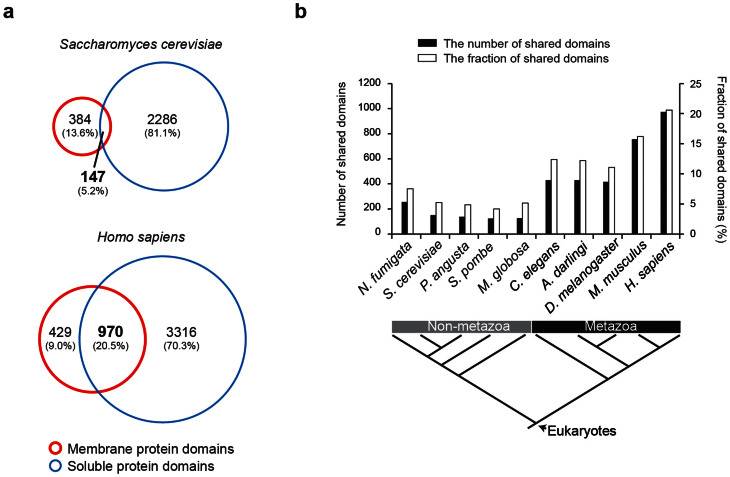
Shared domains of membrane and soluble proteins in non-metazoan and metazoan genomes. (a) Overlap analysis of membrane and soluble protein domains in yeast and human genomes. (b) The number (black bars) and fraction (white bars) of shared domains of membrane and soluble proteins in 5 non-metazoan and 5 metazoan genomes.

We found that membrane proteins share diverse functional domains with soluble proteins in metazoan species compared to non-metazoans. Among 4,715 human domains, 970 domains (20.5%) are shared by membrane and soluble proteins (Fig. 1a). In human genome, 1,276 membrane protein domains and 2,552 soluble protein domains were found. However, in yeast genome, among the total of 2,817 domains, only 137 domains (5.2%) are shared by membrane and soluble proteins. Thus, human membrane proteins have significantly more shared domains compared to yeast membrane proteins. We confirmed that this observation could not occur by random chance comparing them to datasets with randomly assigned membrane and soluble proteins (Kolmogorov-Smirnov test; *p*-value = 3.49 × 10^−96^, Supplementary Fig. S1). We analyzed 59 non-metazoan and 43 metazoan genomes and found that metazoans have on average twice more diverse shared domains than non-metazoans (Supplementary Fig. S2). The number and fraction of shared domains in representative non-metazoan and metazoan species are shown in Fig. 1b.

### Membrane proteins often recruit domains from soluble proteins and expand functional diversity of metazoan genome

We examined shared domains in non-metazoan and metazoan genomes. We quantified how frequently membrane proteins in metazoan organisms acquired pre-existing domains in non-metazoans or how frequently shared domains were created with the transition to multicellular organisms. Shared domains were divided into two groups; (1) those are found from both non-metazoan and metazoans, and (2) those are found only from metazoans. We found that shared domains exist in both non-metazoans and metazoans are more frequent than shared domains specific to metazoans. The number of pre-existing shared domains is larger than expected by random chance (Supplementary Fig. S3). In contrast, the number of shared domains specific to metazoan is smaller than expected. These results suggest that membrane proteins frequently reused pre-existing domains to gain new functions.

**Figure 2:**
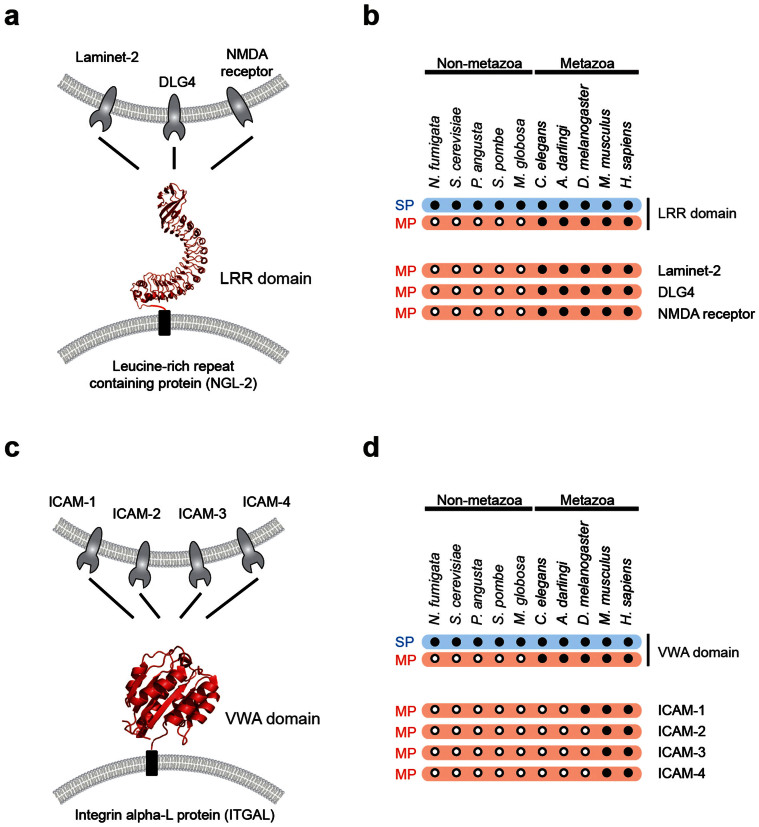
Examples of membrane proteins with shared domains that connect intercellular networks. (a) LRR domains of leucine-rich repeat-containing proteins (NGL-2) interact with Laminet-2, DLG4 and NMDA receptors. (b) Phylogenetic profiles of LRR domains and their interaction partners in non-metazoan and metazoan species. A black dot indicates the presence of shared domains. A hollow dot indicates the absence of shared domains. (c) VWA domains of integrin alpha-L proteins (ITGAL) interact with ICAM1, ICAM2, ICAM3 and ICAM4. (d) Phylogenetic profiles of VWA domains and their interaction partners.

We found that membrane proteins frequently recruit domains from soluble proteins. In metazoan species, various domains are found from both membrane and soluble proteins, but in non-metazoan species, those domains are found only from soluble proteins (Supplementary Fig. S4 and Table S1). For example, in non-metazoan species, leucine-rich repeat (LRR) domains are found only from soluble proteins, but in metazoan species, they become a shared domain and are found in both membrane and soluble proteins (Fig. 2a, b). The LRR domain of membrane protein NGL-2 is a shared domain and functions to expand intercellular PPI network in metazoan nerve system. As a synaptic adhesion protein, NGL-2 regulates the formation of expiatory synapses through LRR domain which recruits pre-and-postsynaptic proteins, such as Laminet-2, DLG4 and NMDA receptors^15^,^16^. Another example, von Willebrand factor type A (VWA) are also found from both membrane and soluble proteins of metazoan species, but they are found only from soluble proteins in non-metazoan species (Fig. 2c, d). A metazoan protein ITGAL recruits intercellular adhesion molecules, such as ICAM1, ICAM2, ICAM3 and ICAM4, and mediates adhesive interaction for immune response and surveillance^17^,^18^. The VWA domain of membrane protein integrin alpha-L (ITGAL) become a shared domain and has an important role in metazoan immune system. These results suggest that recruiting domains from soluble proteins during the evolution may have contributed to the expansion of functional diversity of membrane proteins in metazoan species.

### Shared domains expanded cell-cell communication networks of metazoan species

**Figure 3:**
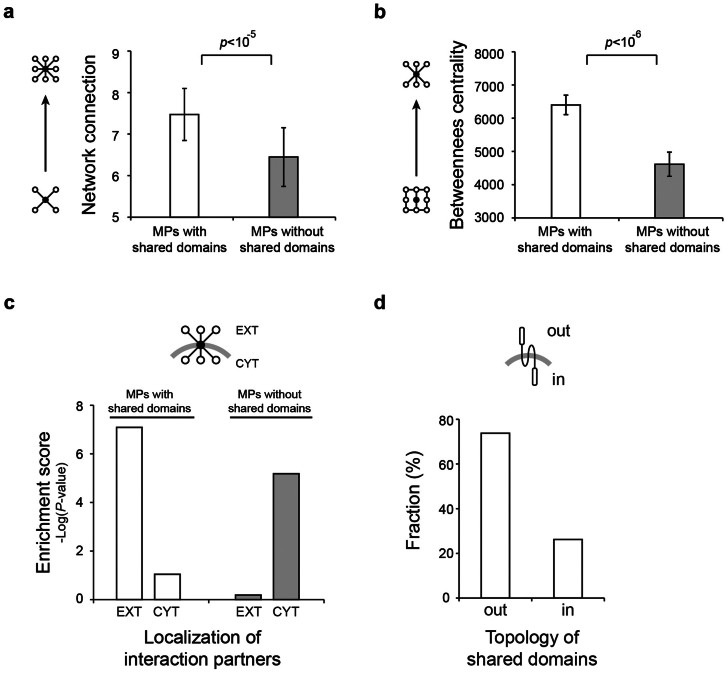
Network properties of membrane proteins with shared domains. (a) The number of network connections (degree) of membrane proteins with and without shared domains were compared in the human PPI network. (b) Comparison of betweenness centrality of membrane proteins with and without shared domains. Error bars represent the standard error. (c) Comparison of the localization enrichment of the interaction partners of membrane proteins with and without shared domains. EXT indicates extracellular interaction partners and CYT indicates cytosolic interaction partners. (d) Topological orientation of shared domains in membrane proteins. ‘Out’ indicates the extracellular side and ‘in’ indicates the cytosolic side. The topology of shared domains was determined by combining the prediction results of TMHMM and location information of Pfam domains.

It has been suggested that increased intercellular network complexity is one of the major factors that contributes to the growth of organismal complexity^19^,^20^,^21^,^22^ so we asked whether shared domains contribute to this process. Connections of membrane proteins with or without shared domain were examined in the human PPI network. We found that membrane proteins with shared domains have a significantly greater number of network connections than those without shared domains (*p*-value = 8.83 × 10^−6^; Fig. 3a). Moreover, membrane proteins with shared domains have a higher betweenness centrality than those without shared domains (*p*-value = 2.94 × 10^−7^; Fig. 3b). Betweenness centrality counts the number of shortest paths between all pairs of nodes passing through it^23^. Thus, proteins with high betweenness centrality are important for information flow in the network^24^,^25^ and tend to interact with many different functional groups^26^. In the topology analysis of fly PPI network, we also confirmed that membrane proteins with shared domains have a significantly greater number of network connection and a higher betweenness centrality than ones without shared domains (Supplementary Fig. S5). Our results suggest that membrane proteins with shared domains contributed to the expansion of diverse network connections and information flow in the cell-cell communication network.

Next, we examined the location of the interaction partners of membrane proteins with or without shared domains. Information of subcellular localization was taken from Gene Ontology^27^ and UniProt database^28^. Among the 1,751 membrane proteins with shared domains, about 80% (1,472) of them are localized at the plasma membrane (Supplementary Fig. S6). We found that membrane proteins with shared domains mostly interact with partners in the extracellular region rather than the cytoplasm (enrichment score = 8.27 × 10^−8^ and 9.08 × 10^−2^ for extracellular and cytosolic proteins, respectively; Fig. 3c), whereas the interaction partners of membrane proteins without shared domains are mainly located at cytoplasm (enrichment score = 6.49 × 10^−1^ and 6.66 × 10^−6^ for extracellular and cytosolic proteins, respectively; Fig. 3c). Moreover, we find that shared domains are generally presented at the extracellular side of membrane proteins. As shown in Fig. 3d, 73.8% (2,124) of shared domains are found on the outside of membrane proteins (extracellular side) compared to only 26.1% (754) of the shared domains that are located at the inside of membrane proteins (cytosolic side). These results suggest that shared domains located at the extracellular side of membrane proteins play a particularly important role in generating the cell-cell communication network.

### Shared domains participate in metazoan-specific functions

**Figure 4:**
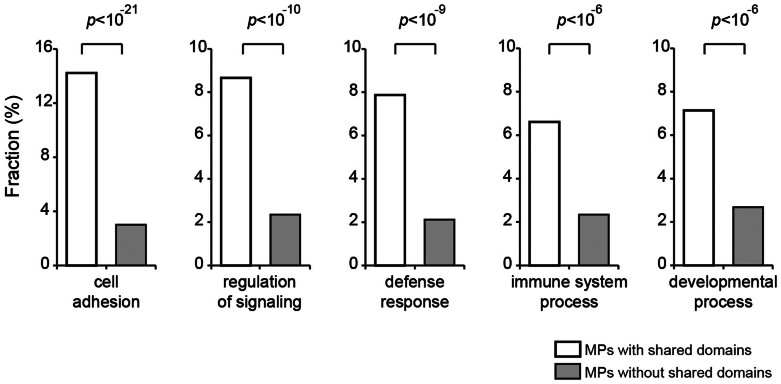
Functional enrichment of membrane proteins with and without shared domains. The fraction of membrane proteins with and without shared domains was compared in functional categories using Gene Ontology (GO). Significantly enriched functional categories at GO level 2 and level 3 are shown (*p*-value < 1.0 × 10^−4^, hypergeometric test).

We next examined the function of shared domains that dramatically increased in metazoan species. We compared the functional difference of proteins with or without shared domains by functional enrichment analysis of Gene Ontology (GO). As shown in Fig. 4, membrane proteins that have shared domains are significantly enriched in five GO categories of biological functions that are important for multicellular life, such as “cell adhesion”, “regulation of signaling”, “defense response”, “immune system process” and “developmental process”. The biological process of “cell adhesion” is significantly enriched in membrane proteins with shared domains compared to the ones without shared domains (*p*-value = 9.46 × 10^−22^; hypergeometric test). Cell-adhesion gene families are critical for metazoan evolution because integrity of multicellular organisms is sustained by the stable adhesion of neighboring cells^29^. For example, a shared domain of metazoan membrane protein, F5/8 type C domain of contactin-associated proteins-like 2, plays a critical role for the interactions between neurons and glia during nervous system development^30^ and mediate the formation of an adhesion complex for salutatory conduction^31^,^32^. Biological functions of “defense response” and “regulation of immune system process” are greatly overrepresented among membrane proteins with shared domains (*p*-value = 2.48 × 10^−10^ and *p*-value = 6.10 × 10^−7^, respectively; hypergeometric test). Multicellular organisms have developed effective defense and immune system to combat microbes and parasites^33^. For example, Necrosis Factor domains and C-type lectin domains are found to be shared domains of metazoan membrane proteins and have functional roles in immune cells to contact to their cognate receptors in other cells^34^,^35^. Moreover, the biological function of “developmental process” is significantly enriched in membrane proteins with shared domains (*p*-value = 5.79 × 10^−7^; hypergeometric test). Developmental process is a crucial progression for metazoan species to generate and organize specialized cell types and organs. Other GO categories significantly different between membrane proteins with or without shared domains are listed in Supplementary Table S2 These results suggest that shared domains are particularly important in metazoan specific functions and contribute to the transition to multicellularity.

## Discussion

Our results show that the incorporation of soluble domains into membrane proteins is a major contributor to the development of cellular diversification and cooperation enabled metazoan evolution. Previously, it has been suggested that membrane proteins expand their structural diversity by domain duplication^36^,^37^,^38^ or hetero-oligomerization^13^. However, domain recombination within membrane proteins is rare since the fold diversity of membrane proteins is limited^10^. Current approaches for understanding the functional diversity of membrane proteins have focused on transmembrane domains, rather than their extra-membrane domains^9^, but we recently showed that the incorporation of soluble protein domains is a major mechanism for the diversification of membrane proteins during evolution^14^. Our results suggest that domain sharing of membrane and soluble proteins is an important evolutionary pathway to obtain the functional diversity of membrane proteins.

Our study reveals that the incorporation of soluble extracellular domains was a particularly powerful mechanism for the evolution of multicellular life. Consistent with our finding, it has been shown that a large number of extracellular matrix proteins were exist in non-metazoans and expanded by domain shuffling during the transition to metazoans^39^,^40^. Diverse domains of soluble proteins recombined with transmembrane domains and became a key player for cell-cell communications in metazoan species. Membrane-anchored domains on cell surface are positioned to interact with proteins on neighboring cells and to detect various secreted molecules and to transfer the chemical information inside the cell. In unicellular organism, LRR domains usually function as an adapter for protein interactions of soluble proteins inside the cell^41^,^42^. However, in multicellular organisms, LRR domains of membrane proteins anchored to cell surface, thereby creating a new function as cell-adhesion or cell-cell communication units in pre-and-post synaptic cells (Fig. 2a)^15^,^16^. Moreover, membrane-anchored domains play an important role for self versus non-self recognition^43^. For example, VWA domains of membrane proteins function on the surface of immune cells in metazoan species (Fig. 2c)^17^,^18^. VWA domains of membrane-anchored integrin alpha-L proteins are known to enhance the binding affinity of immune complexes in leukocyte-endothelial cell-cell interaction and cytotoxic T-cell mediated immune responses^44^,^45^. Our findings suggest that recombination of soluble and transmembrane domains confer new biological functions for metazoan multicellularity since the combination of two domains can make a new entity with both functions.

We found that domain sharing between membrane and soluble proteins is coupled with the appearance of multicellular organisms to contribute their intercellular networks with higher complexity. Especially, metazoans turn out to have more diverse shared domains than non-metazoans (Fig. 1). One might ask that shared domains likely increase in other evolutionary transitions such as the appearance of vertebrates or mammals. However, such increase was not observed in vertebrates or mammals (Supplementary Fig. S2a). In the analysis of shared domain within metazoan species including 5 vertebrates and 12 mammals, significant increases of shared domains were not found.

We found that membrane proteins with shared extra-membrane domains have increased network connections and information flow for the extracellular PPI network (Fig. 3). Intercellular communication is the key to maintain homeostasis, developmental, defense and immune process of multicellular organism^46^. Therefore, many efforts have been invested to identify novel extracellular interactions and in constructing cell-cell communication networks^20^,^21^,^22^. Recently, comparative genomic studies on the nervous system discovered a few components of metazoan synaptic transmission and plasticity^47^,^48^,^49^. However, it has been generally difficult to characterize molecular components that form intercellular communication system important for the evolution of multicellular organisms^50^. Our findings suggest that the characterization of the domain diversity of membrane proteins may improve the understanding of the evolution of cell-cell communication network.

## Methods

### Classification of membrane and soluble proteins

We classified proteins of non-metazoan and metazoan genomes into membrane and soluble proteins by using experimental annotations and the transmembrane domains (TMDs) prediction program. The overall workflow is outlined in Supplementary Fig. S7. We first downloaded complete or reference genomes of 5 non-metazoan and 5 metazoan species from UniProt database^28^. Membrane proteins were identified by having TMDs as indicated by the UniProt and Pfam databases^51^. Proteins annotated as ‘Single-/Multi-pass membrane proteins’ and ‘Transmembrane proteins’ were included, but ‘Peripheral membrane proteins’ were excluded. For the proteins without experimental annotation, we predicted TMDs by using TMHMM^52^, which is one of most the reliable TMD prediction programs for a large number of sequences^53^,^54^. Proteins with predicted TMDs were considered to be membrane proteins^14^. We collected soluble proteins by excluding membrane proteins and putative membrane proteins. The total number of membrane and soluble proteins of each non-metazoan and metazoan genome are listed in Supplementary Table S3.

### Identification of shared domains, membrane protein and soluble protein domains

We assigned 5,952Pfam domains (Release 26)^51^ into membrane and soluble proteins of non-metazoan and metazoan genomes. All the sequences of membrane and soluble proteins were searched against the profile hidden Markov models of Pfam-A domains using pfam_scan.pl script and HMMER3^55^. From the derived domains, we identified “*shared domains*”, “*membrane protein domains*”, and “*soluble protein domains*”. Shared domains are the domains that are found in both membrane and soluble proteins, whereas, membrane protein domains represent the domains which exist in membrane proteins. Soluble protein domains represent the domains which exist in soluble proteins. Then, we calculated the fraction of three domain classes by dividing the total number of Pfam domain in individual genome. Table 1 lists the numbers and fractions of shared domains, membrane protein and soluble protein domains of all 5 non-metazoan and 5 metazoan genomes.

### Functional enrichment analysis

To investigate biological functions of proteins with shared domains, we utilized the Gene Ontology (GO) Biological Process (BP) annotations^27^ that were derived from the entire GO (version 1.2). We examined which GO BP terms were enriched in the proteins with shared domains relative to the proteins without shared domains. First, we extracted 2,011 BP terms at GO level 2 and level 3, each of which annotates at most 20,000 human proteins. Next, we selected statistically overrepresented BP terms for the proteins with shared domains and compared with the proteins without shared domains using a hypergeometric test. Only GO BP terms that were overrepresented with *p*-value lower than 1.0 × 10^−4^ were employed. The same procedure was applied for membrane and soluble protein domains. Supplementary Table S2 lists all GO BP terms which are significantly enriched in the proteins with shared domains.

### Transmembrane topology of shared domains

To investigate whether shared domains are located on the inside or outside of cells, we first predicted the transmembrane (TM) topology of the membrane proteins by using TMHMM^54^, and then assigned the sequence-position information of shared domains into the TM topology. We evaluated the reliability of localization prediction of shared domains. The results of TMHMM prediction and the experimentally confirmed annotations of UniProt database were compared. We examined the agreement of location (extracellular and cytoplasmic sides) of shared domains. The overall accuracy and precision of localization prediction of shared domains reached 92% and 94%, respectively (Supplementary Fig. S8).

### Subcellular localization analysis

Subcellular localizations of membrane proteins with shared domains were derived from UniProt database^28^. Five subcellular localizations (plasma membrane, ER membrane, Golgi membrane, mitochondrial membrane and nuclear membrane) and their transmembrane topology were listed in Supplementary Table S4.

The subcellular localization of membrane protein interaction partners was derived from Gene Ontology (GO) Cellular Component (CC) annotation (version 1.2)^27^. Extracellular and cytosolic subcellular localization were assigned. Proteins with CC terms of “extracellular region” and “extracellular space” were assigned as extracellular proteins (EXT) and those with CC terms of “cytosol” and “cytoplasmic part” were assigned as cytosol proteins (CYT). Subcellular localization information was available for 1,018 proteins of 2,362 interaction partners of membrane proteins. We used the hypergeometric distribution to calculate the enrichment score for the observed fraction of extracellular and cytosol partners of membrane proteins with or without domains.

### Construction of protein interaction network

We compiled human protein interactions from a total of 22 existing protein interaction databases^56^: the Bio-molecular Interaction Network Database (BIND), the Human Protein Reference Database (HPRD), the Molecular Interaction database (MINT), DIP, IntAct, BioGRID, Reactome, the Protein-Protein Interaction Database (PPID), BioVerse, CCS-HI1, the comprehensive resource of mammalian protein complexes (CORUM), IntNetDB, the Mammalian Protein-Protein Interaction Database (MIPS), the Online Predicted Human Interaction Database (OPHID), Ottowa, PC/Ataxia, Sager, Transcriptome, Complexex, Unilever, protein-protein interaction database for PDZ-domains (PDZBase), and a protein interaction dataset from the literature^57^. We removed low-confidence interactions that were not supported by direct experimental evidence, such as PPIs inferred from orthologous interactions or obtained by co-expression signals from microarray experiments. The final network comprised 101,777 interactions between 11,043 human proteins.

## Supplementary Material

Supplementary Information Supplementary Information

## Figures and Tables

**Table 1:** Distribution of the shared, membrane and soluble protein domains in non-metazoan and metazoan genomes. The sum of the fractions of shared domains, membrane protein domains and soluble proteins domains exceed 100%, because shared domains are included in both membrane and soluble protein domains

Taxonomy	Species	The number of domain types				The fraction of domain types (%)		
		Shared domains	Membrane protein domains	Soluble protein domains	Total domains	Shared domains	Membrane protein domains	Soluble protein domains
Non-metazoan	*Neosartorya fumigata*	253	702	2920	3369	7.51	20.84	86.67
Non-metazoan	*Saccharomyces cerevisiae*	147	531	2433	2817	5.22	18.85	86.37
Non-metazoan	*Pichia angusta*	135	489	2430	2784	4.85	17.56	87.28
Non-metazoan	*Schizosaccharomyces pombe*	120	527	2470	2877	4.17	18.32	85.85
Non-metazoan	*Malassezia globosa*	123	413	2105	2395	5.14	17.24	87.89
Metazoan	*Caenorhabditis elegans*	426	940	2927	3441	12.38	27.32	85.06
Metazoan	*Anopheles darlingi*	426	977	2943	3494	12.19	27.96	84.23
Metazoan	*Drosophila melanogaster*	413	997	3148	3732	11.07	26.71	84.35
Metazoan	*Mus musculus*	753	1308	4097	4652	16.19	28.12	88.07
Metazoan	*Homo sapiens*	970	1399	4286	4715	20.57	29.67	90.90
