# Supplementary material for: Metazoans evolved by taking domains from soluble proteins to expand intercellular communication network
Source: Sci Rep. 2015 Apr 29;5:9576. doi: 10.1038/srep09576 (PMC4894438; doi:10.1038/srep09576)
Supplement: Supplementary Information [file srep09576-s1.pdf]

Supplementary information

Metazoans evolved by taking domains from soluble proteins to expand intercellular communication network

Hyun-Jun Nam[1], Inhae Kim[2], James U. Bowie[3], Sanguk Kim[1,2,†]

[1]School of Interdisciplinary Bioscience and Bioengineering, [2]Department of Life Sciences, Pohang University of Science and Technology, Pohang, 790-784, Korea, [3]Department of Chemistry and Biochemistry, UCLA-DOE Institute of Genomics and Proteomics, Molecular Biology Institute, University of California, Los Angeles, Los Angeles, California 90095-1570, United States

Supplementary Figures and Tables

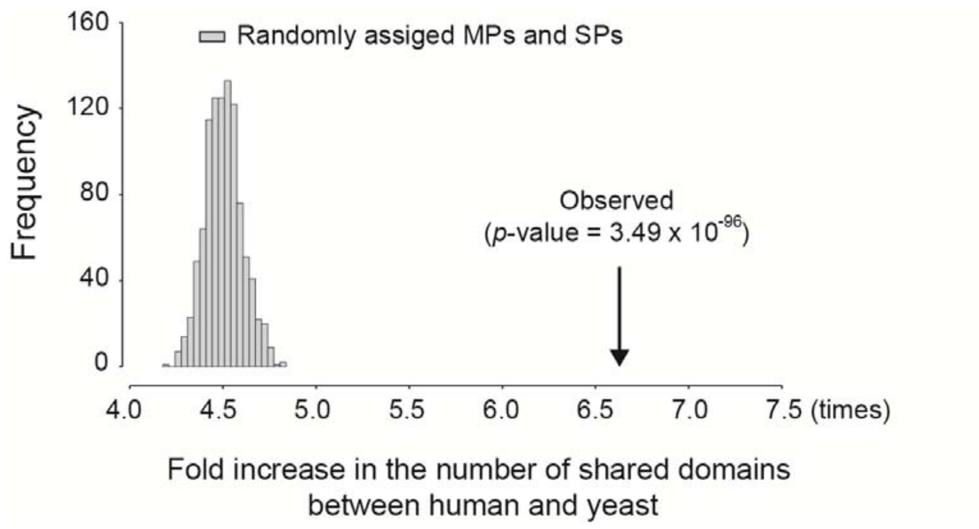

Supplementary Figure S1. Fold increase in the number of shared domains between human and yeast genome. Grey bars show the distribution of the fold increase of shared domains in dataset with randomly assigned membrane and soluble proteins.

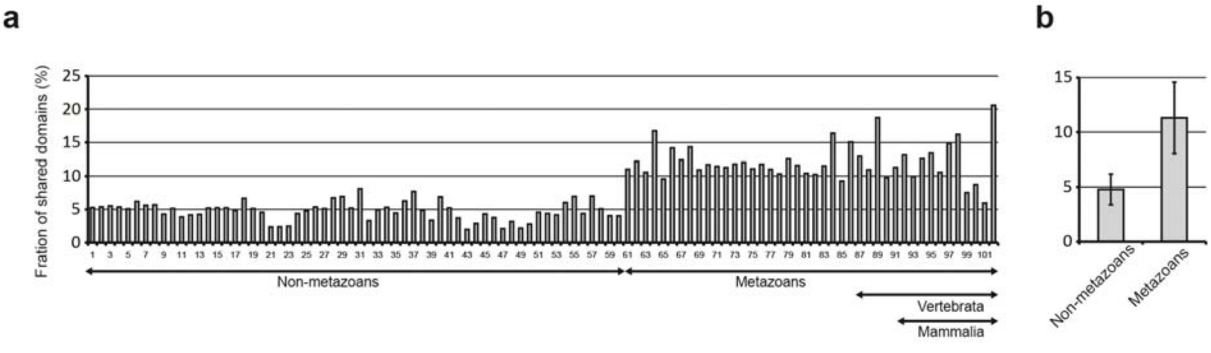

c

1. Ajellomyces capsulata
2. Ajellomyces capsulata
3. Ajellomyces capsulata
4. Ajellomyces dermatitidis
5. Ajellomyces dermatitidis
6. Arthroderma benhamiae
7. Arthroderma gypseum
8. Arthroderma otae
9. Ashbya gossypii
10. Candida albicans
11. Candida albicans
12. Candida dubliniensis
13. Candida glabrata
14. Candida tropicalis
15. Clavispora lusitaniae
16. Coccidioides posadasii
17. Coccidioides posadasii
18. Colletotrichum graminicola
19. Cryptococcus gattii serotype B
20. Debaryomyces hansenii
21. Encephalitozoon cuniculi
22. Encephalitozoon intestinalis
23. Enterocytozoon bieneusi
24. Kluyveromyces lactis
25. Lachancea thermotolerans
26. Lodderomyces elongisporus
27. Malassezia globosa
28. Metarhizium acridum
29. Metarhizium anisopliae
30. Meyerozyma guilliermondii
31. Nectria haematococca
32. Nosema ceranae
33. Paracoccidioides brasiliensis
34. Paracoccidioides brasiliensis
35. Paracoccidioides brasiliensis
36. Penicillium chrysogenum
37. Penicillium marneffei
38. Pichia angusta
39. Pichia pastoris
40. Puccinia graminis f. sp. tritici
41. Saccharomyces cerevisiae
42. Saccharomyces cerevisiae
43. Saccharomyces cerevisiae
44. Saccharomyces cerevisiae
45. Saccharomyces cerevisiae
46. Saccharomyces cerevisiae
47. Saccharomyces cerevisiae
48. Saccharomyces cerevisiae
49. Saccharomyces cerevisiae
50. Saccharomyces cerevisiae
51. Scheffersomyces stipitis
52. Schizosaccharomyces japonicus
53. Schizosaccharomyces pombe
54. Uncinocarpus reesii
55. Ustilago maydis
56. Vanderwaltozyma polyspora
57. Verticillium albo-atrum
58. Yarrowia lipolytica
59. Zygosaccharomyces rouxii
60. Acyrthosiphon pisum
61. Aedes aegypti
62. Anopheles darlingi
63. Anopheles gambiae
64. Branchiostoma floridae
65. Brugia malayi
66. Caenorhabditis briggsae
67. Caenorhabditis elegans
68. Caenorhabditis remanei
69. Ciona intestinalis
70. Culex quinquefasciatus
71. Daphnia pulex
72. Drosophila ananassae
73. Drosophila erecta
74. Drosophila grimshawi
75. Drosophila melanogaster
76. Drosophila mojavensis
77. Ixodes scapularis
78. Loa loa
79. Nematostella vectensis
80. Oikopleura dioica
81. Pediculus humanus subsp. corporis
82. Solenopsis invicta
83. Tribolium castaneum
84. Trichinella spiralis
85. Trichoplax adhaerens
86. Danio rerio
87. Gallus gallus
88. Oryzias latipes
89. Tetraodon nigroviridis
90. Xenopus tropicalis
91. Ailuropoda melanoleuca
92. Canis familiaris
93. Equus caballus
94. Bos taurus
95. Sus scrofa
96. Oryctolagus cuniculus
97. Rattus norvegicus
98. Mus musculus
99. Macaca fascicularis
100. Macaca mulatta
101. Pongo abelii
102. Homo sapiens

Supplementary Figure S2. Shared domains of membrane and soluble proteins in non-metazoan and metazoan genomes. (**a**) The fraction of shared domains of membrane and soluble proteins in 59 non-metazoan and 43 metazoans. (**b**) Average fractions of shared

domains of non-metazoans and metazoans. Error bars represent the standard deviation. (**c**) The list of 59 non-metazoan and 43 metazoan genomes that are used for examining shared domains.

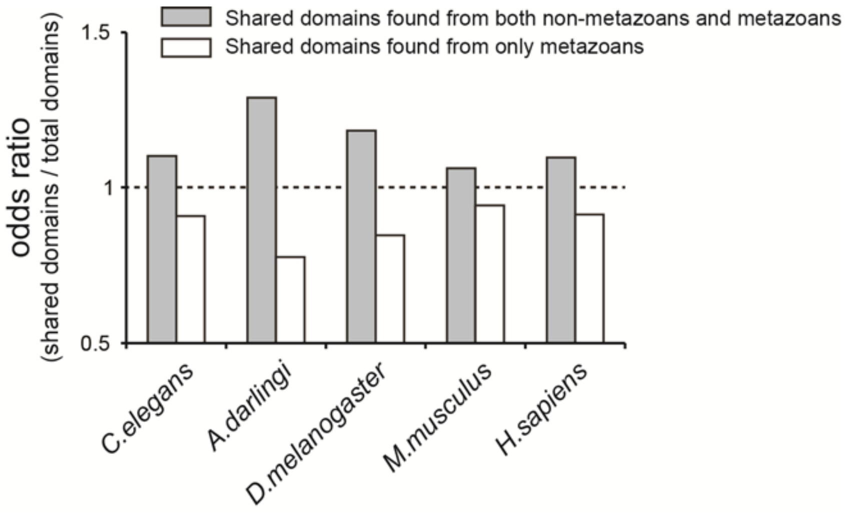

Supplementary Figure S3. Phylogenetic analysis of shared domains. Grey bars represent the shared domains that are found from both non-metazoans and metazoans (pre-existing domains). White bars represent shared domains that are found from only metazoan species (metazoan-specific domains). Odds ratio was calculated as the fraction of each group in shared domains divided by the fraction of each group in all membrane protein domains.

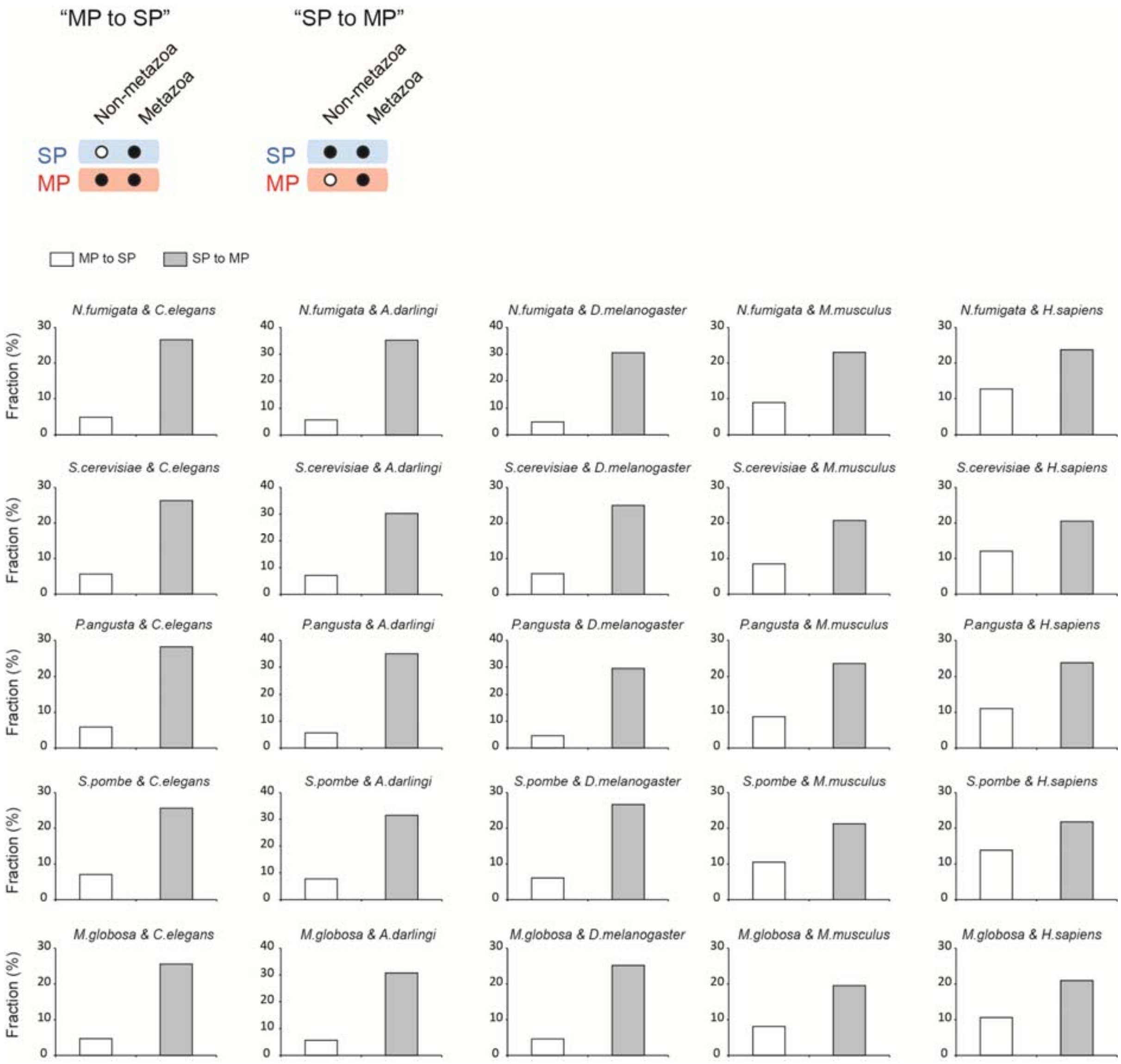

Supplementary Figure S4. Fraction of metazoan shared domains that were found from soluble or membrane proteins in non-metazoan species. White bar (MP to SP) indicates the fraction of metazoan shared domains found from membrane proteins of non-metazoan species. Grey bar (SP to MP) indicates the fraction of metazoan shared domains found from soluble proteins of non-metazoan species.

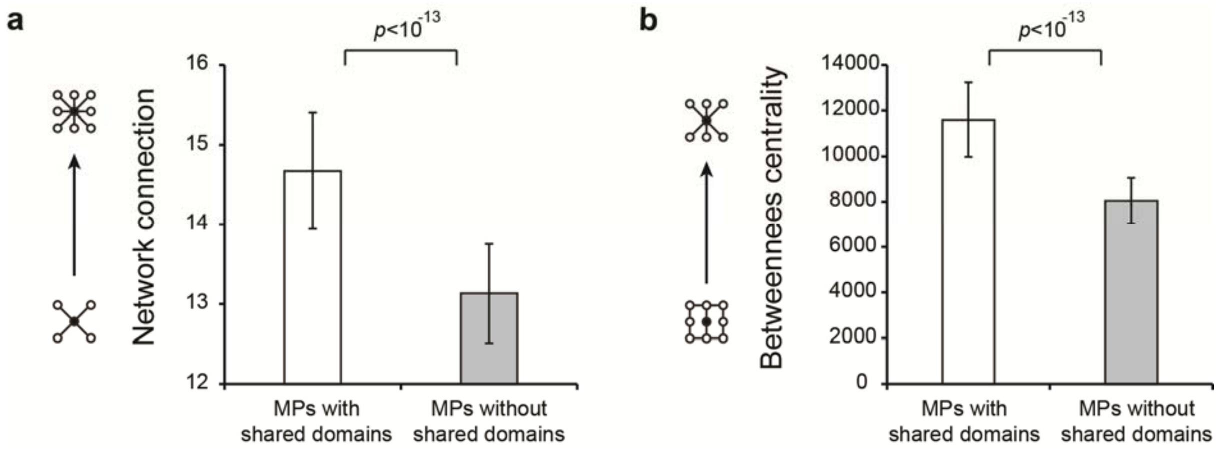

Supplementary Figure S5. Network properties of fly membrane proteins with shared domains. (**a**) The number of network connections (degree) of membrane proteins with and without shared domains were compared in the fly PPI network. (**b**) Comparison of betweenness centrality of membrane proteins with and without shared domains. Error bars represent the standard error.

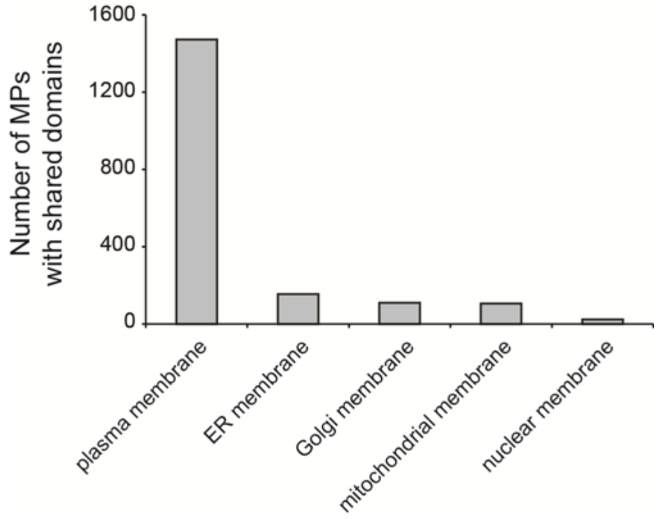

Supplementary Figure S6. Localization of membrane proteins that have shared domains.

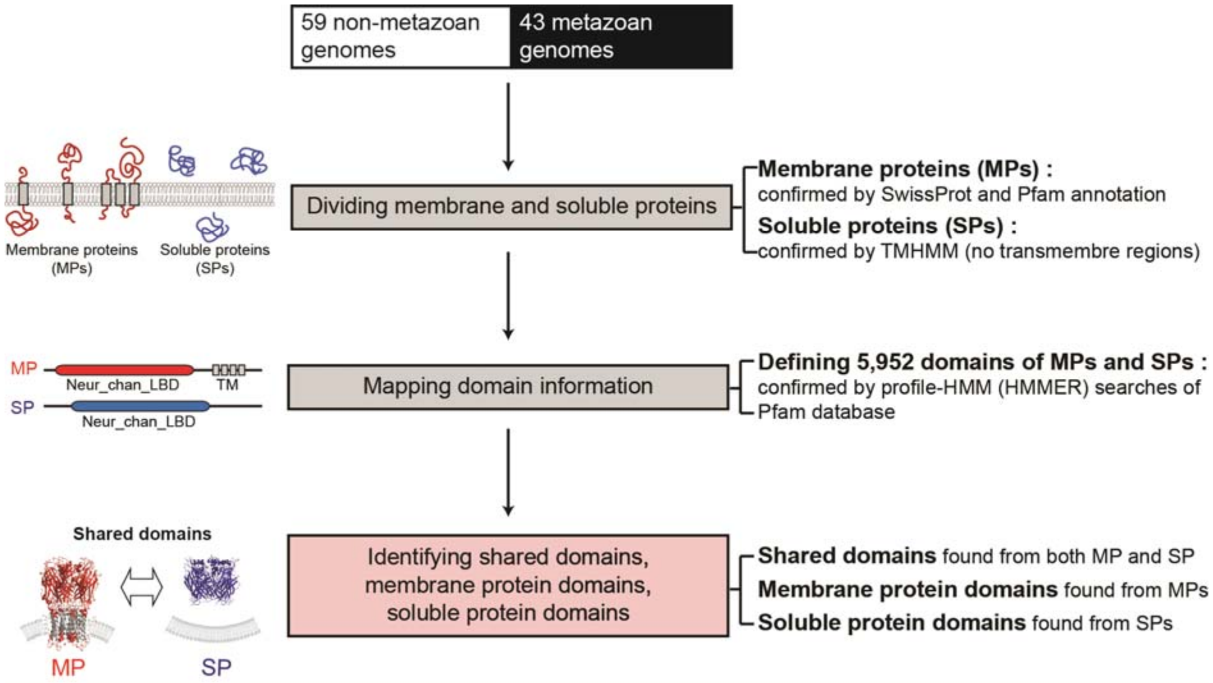

Supplementary Figure S7. Identification of shared domains of membrane and soluble proteins in non-metazoan and metazoan genomes.

		UniProt annotation	
		EXT	CYT
TMHMM prediction	EXT	1371	74
	CYT	57	150

Accuracy = 0.920 Precision = 0.948

Supplementary Figure S8. Comparison of location of shared domains by TMHMM prediction and UniProt annotations. EXT indicates extracellular side (outside of the cell) and CYT indicates cytosolic side (inside of the cell).

Supplementary Table S1. List of shared, membrane and soluble protein domains in non-metazoan and metazoan genomes

Domains	Non-metazoa					Metazoa				
	N. fumigata	*S. cerevisiae*	*P. angusta*	*S. pombe*	*M. globosa*	*C. elegans*	*A. darlingi*	*D. melanogaster*	*M. musculus*	*H. sapiens*
DAGK_cat	*S*	*S*	*S*	*S*	*S*	*S/M*	*S/M*	*S/M*	*S/M*	*S/M*
TUDOR	*S*	-	*S*	*S*	-	*S/M*	*S/M*	*S/M*	*S/M*	*S/M*
BTB	*S*	*S*	*S*	*S*	-	*S/M*	*S/M*	*S/M*	*S/M*	*S/M*
Epimerase	*S*	*S*	*S*	*S*	*S*	*S/M*	*S/M*	*S/M*	*S/M*	*S/M*
SAM_1	*S*	-	-	-	*S*	*S/M*	*S/M*	*S/M*	*S/M*	*S/M*
SAM_2	*S*	*S*	*S*	*S*	*S*	*S/M*	*S/M*	*S/M*	*S/M*	*S/M*
Y_phosphatase	*S*	*S*	*S*	*S*	*S*	*S/M*	*S/M*	*S/M*	*S/M*	*S/M*
Ras	*S*	*S*	*S*	*S*	*S*	*S/M*	*S/M*	*S/M*	*S/M*	*S/M*
Alpha-mann_mid	*S*	*S*	*S*	*S*	-	*S/M*	*S/M*	*S/M*	*S/M*	*S/M*
LRR_8	*S*	*S*	*S*	*S*	*S*	*S/M*	*S/M*	*S/M*	*S/M*	*S/M*
KH_1	*S*	*S*	*S*	*S*	*S*	*S/M*	*S/M*	*S/M*	*S/M*	*S/M*
LRR_4	*S*	*S*	*S*	*S*	*S*	*S/M*	*S/M*	*S/M*	*S/M*	*S/M*
Glyco_hydro_38	*S*	*S*	*S*	*S*	-	*S/M*	*S/M*	*S/M*	*S/M*	*S/M*
Homeobox	*S*	*S*	*S*	*S*	*S*	*S/M*	*S/M*	*S/M*	*S/M*	*S/M*
Pkinase_Tyr	*S*	-	*S*	-	-	*S/M*	*S/M*	*S/M*	*S/M*	*S/M*
Oxysterol_BP	*S*	*S*	*S*	*S*	*S*	*S/M*	*S/M*	*S/M*	*S/M*	*S/M*
C1_1	*S*	*S*	*S*	*S*	*S*	*S/M*	*S/M*	*S/M*	*S/M*	*S/M*
Glyco_hydro_38C	*S*	*S*	*S*	*S*	-	*S/M*	*S/M*	*S/M*	*S/M*	*S/M*
Guanylate_cyc	*S*	*S*	*S*	*S*	*S*	*S/M*	*S/M*	*S/M*	*S/M*	*S/M*
CRAL_TRIO	*S*	*S*	*S*	*S*	*S*	*S/M*	*S/M*	*S/M*	*S/M*	*S/M*
IBR	*S/M*	*S*	*S*	*S*	*S*	*S/M*	*S/M*	*S/M*	*S/M*	*S/M*
Disintegrin	*S/M*	-	-	*S*	-	*M*	*M*	*S/M*	*S/M*	*S/M*
ACBP	*S/M*	*S*	*S*	*S*	*S*	*S/M*	*S/M*	*S/M*	*S/M*	*S/M*
His_Phos_2	*S/M*	*S*	*S*	*S*	*S*	*S/M*	*S/M*	*S/M*	*S/M*	*S/M*
Laminin_G_3	*S/M*	-	-	*S*	*S*	*S/M*	*S/M*	*S/M*	*S/M*	*S/M*
Ribonuc_red_sm	*M*	*S*	*S*	*S*	*S*	*S/M*	*M*	*M*	*S/M*	*S/M*
Recep_L_domain	*M*	-	-	*S*	-	*S/M*	*M*	*S/M*	*S/M*	*S/M*
Inositol_P	*S/M*	*S*	*S*	*S*	*S*	*S/M*	*S/M*	*S/M*	*S/M*	*S/M*
Sulfatase	*S/M*	-	*S*	*S*	-	*S/M*	*S/M*	*S/M*	*S/M*	*S/M*
RRM_1	*S/M*	*S*	*S*	*S*	*S*	*S/M*	*S/M*	*S/M*	*S/M*	*S/M*
K_tetra	-	*S*	-	-	-	*S/M*	*S/M*	*S/M*	*S/M*	*S/M*
Lipase_GDSL	*S*	*S*	*S*	-	*M*	*S/M*	*S/M*	*S/M*	*S/M*	*S/M*
Saccharop_dh	*S*	*S*	*S*	*S*	*S/M*	*S/M*	*S/M*	*S/M*	*S/M*	*S/M*
DUF3358	*S*	*S*	*S*	*S*	*S/M*	*S/M*	*S/M*	*S/M*	*S/M*	*S/M*
Ank	*S*	-	*S*	*S*	*S/M*	*S/M*	*S/M*	*S/M*	*S/M*	*S/M*
Peptidase_M1	*S*	*S*	*S*	*S*	*S/M*	*S/M*	*S/M*	*S/M*	*S/M*	*S/M*
Kelch_5	*S/M*	*S*	*S*	*S*	*S/M*	*S/M*	*S/M*	*S/M*	*S/M*	*S/M*
COesterase	*S/M*	-	*S*	*S*	*M*	*S/M*	*S/M*	*S/M*	*S/M*	*S/M*
CHD5	*M*	*S*	*S*	*S*	*M*	*M*	*M*	*M*	*M*	*S/M*
WD40	*S/M*	*S*	*S*	*S*	*S/M*	*S/M*	*S/M*	*S/M*	*S/M*	*S/M*
UDPGT	*S/M*	*S*	*S*	-	*M*	*S/M*	*S/M*	*M*	*S/M*	*S/M*
COX6A	*S*	*M*	*S*	*S*	-	*M*	*S*	*S/M*	*S/M*	*S/M*
TPP_enzyme_M	*S/M*	*S/M*	*S*	*S*	*S*	*S/M*	*S*	*M*	*S/M*	*S/M*
TPP_enzyme_C	*S/M*	*S/M*	*S*	*S*	*S*	*S/M*	*S*	*M*	*S/M*	*S/M*
TPP_enzyme_N	*S/M*	*S/M*	*S*	*S*	*S*	*S/M*	*S*	*M*	*S/M*	*S/M*
FUN14	*S*	*M*	*S*	*S*	*M*	*M*	*S*	*S/M*	*S*	*S/M*
ADAM_spacer1	-	-	-	-	-	*S/M*	*S*	*S/M*	*S*	*S/M*
Helicase_C	*S*	*S*	*S*	*S/M*	*S/M*	*S*	*S/M*	*S/M*	*S/M*	*S/M*
DUF1977	*S*	-	*S*	*M*	*M*	*S*	*S/M*	*M*	*S/M*	*S/M*
DEAD	*S*	*S*	*S*	*S/M*	*S/M*	*S*	*S*	*S/M*	*S/M*	*S/M*
Thioredoxin_6	*S*	*S*	*S*	*S/M*	*S/M*	*S/M*	*S/M*	*S/M*	*S/M*	*S/M*
Glyco_hydro_63	*S*	*S*	*S*	*M*	*M*	*M*	-	-	*M*	*S/M*
Sec20	*S*	*S*	*S*	*M*	*M*	*M*	*M*	*M*	*M*	*S/M*
Syntaxin-18_N	*S*	*S*	*S*	*M*	-	-	*M*	*M*	*M*	*S/M*
PA14	*S*	*S/M*	*S*	*S*	-	-	-	-	*S/M*	*S/M*
DnaJ_C	*S*	*S/M*	*S*	*S*	*S/M*	*S*	*S/M*	*S/M*	*S/M*	*S/M*
2OG-FeII_Oxy	*S*	-	*S*	*S*	-	*S*	*S/M*	*S/M*	*S/M*	*S/M*
CPSase_L_chain	*S*	*S*	*S*	*S*	*S*	*S*	*S/M*	*S/M*	*S/M*	*S/M*
Glyco_hydro_20b	*S*	-	-	-	-	*S*	*S/M*	*S/M*	*M*	*S/M*
5_nucleotid_C	*S*	-	-	-	*S*	-	*S/M*	*S/M*	*S/M*	*S/M*
Glyco_hydro_35	*S*	-	-	-	-	*S*	*S/M*	*S/M*	*S/M*	*S/M*
Carboxyl_trans	*S*	*S*	*S*	*S*	*S*	*S*	*S/M*	*S/M*	*S/M*	*S/M*
ACC_central	*S*	*S*	*S*	*S*	-	*S*	*M*	*S/M*	*S/M*	*S/M*
HECT	*S*	*S*	*S*	*S*	*S*	*S*	*S/M*	*S/M*	*S/M*	*S/M*
FKBP_C	*S*	*S*	*S*	*S*	*S*	*S*	*S/M*	*S/M*	*S/M*	*S/M*
MIT	*S*	*S*	*S*	*S*	*S*	*S*	*S/M*	*S/M*	*S/M*	*S/M*
Kelch_4	*S*	*S*	-	*S*	-	*S*	*S/M*	*S/M*	*S/M*	*S/M*
CH	*S*	*S*	*S*	*S*	*S*	*S*	*S/M*	*S/M*	*S/M*	*S/M*
Biotin_carb_C	*S*	*S*	*S*	*S*	*S*	*S*	*S/M*	*S/M*	*S/M*	*S/M*
DNA_pol_A_exo1	*S*	*S*	*S*	*S*	*S*	*S*	*S/M*	*S/M*	*S/M*	*S/M*
Biotin_lipoyl	*S*	*S*	*S*	*S*	*S*	*S*	*S/M*	*S/M*	*S/M*	*S/M*
Glyco_hydro_2_C	*S*	-	-	-	-	*S*	*M*	*S/M*	*S/M*	*S/M*
DEP	*S*	*S*	*S*	*S*	*S*	*S*	*S/M*	*S/M*	*S/M*	*S/M*
CPSase_L_D2	*S*	*S*	*S*	*S*	*S*	*S*	*S/M*	*S/M*	*S/M*	*S/M*
VWA_2	*S*	*S*	*S*	*S*	*S*	*S/M*	*S/M*	*S/M*	*S/M*	*S/M*
NAD_binding_4	*S*	*S*	*S*	*S/M*	*S*	*S/M*	*S/M*	*S/M*	*S/M*	*S/M*
Dynamin_N	*S*	*S*	*S/M*	*S/M*	*S*	*S/M*	*S/M*	*S/M*	*S/M*	*S/M*
zf-LITAF-like	*S*	-	-	-	-	*M*	*S*	*S/M*	*S/M*	*S/M*
Peptidase_S24	*S*	*S*	*S/M*	*S/M*	-	*S/M*	*S*	*S/M*	*S/M*	*S/M*
Beta_helix	*S*	-	-	-	-	*S/M*	*S*	*S/M*	*S/M*	*S/M*
Glyco_hydro_20	*S*	-	-	-	-	*S/M*	*S/M*	*S/M*	*S/M*	*S/M*
Peptidase_M13	*S*	-	-	-	*S/M*	*S/M*	*S/M*	*S/M*	*S/M*	*S/M*
Peptidase_S28	*S*	-	-	-	-	*S/M*	*S/M*	*S/M*	*S/M*	*S/M*
CtaG_Cox11	*S*	*M*	*M*	*M*	*M*	*S/M*	*M*	*M*	*S/M*	*S/M*
EGF_CA	*S*	-	-	-	-	*S/M*	*S/M*	*S/M*	*S/M*	*S/M*
An_peroxidase	*S*	-	-	-	-	*S/M*	*S/M*	*S/M*	*S/M*	*S/M*
fn3	*S*	-	-	-	-	*S/M*	*S/M*	*S/M*	*S/M*	*S/M*
F5_F8_type_C	*S*	-	-	-	-	*M*	*S/M*	*S/M*	*S/M*	*S/M*
Peptidase_M13_N	*S*	-	-	-	*S/M*	*S/M*	*S/M*	*S/M*	*S/M*	*S/M*
CarboxypepD_reg	*S*	-	-	-	-	*S/M*	*S/M*	*S/M*	*S/M*	*S/M*
LETM1	*S*	*M*	*M*	*M*	*M*	*S/M*	*S/M*	*M*	*M*	*S/M*
Collagen	*S*	-	-	-	-	*S/M*	*S/M*	*S/M*	*S/M*	*S/M*
CAP	*S*	*S/M*	*S/M*	-	-	*S/M*	*S/M*	*S/M*	*S/M*	*S/M*
VCBS	*S*	-	-	-	-	*M*	*M*	*M*	*S/M*	*S/M*
Peptidase_M14	*S*	*M*	*S*	*M*	-	*S/M*	*S/M*	*S/M*	*S/M*	*S/M*
NIF	*S*	*S/M*	*S*	*S/M*	*S*	*S/M*	*S/M*	*S/M*	*S/M*	*S/M*
PH	*S*	*S/M*	*S*	*S/M*	*S*	*S/M*	*S/M*	*S/M*	*S/M*	*S/M*
G_glu_transpept	*S*	*M*	*S*	*M*	*S*	*S/M*	*S/M*	*S/M*	*S/M*	*S/M*
Ank_4	*S*	*S/M*	*S*	-	*S*	*S/M*	*S/M*	*S/M*	*S/M*	*S/M*
DAGAT	*S*	*M*	*S*	*M*	*S*	*M*	*M*	*M*	*M*	*S/M*
Ribonuc_2-5A	*S*	*M*	*M*	*M*	*S*	*S/M*	*M*	*S/M*	*S*	*S/M*
PIG-H	*S*	*M*	*M*	*M*	*S*	-	*M*	-	*M*	*S/M*
Aminotran_1_2	*S/M*	*S*	*S/M*	*S/M*	*S*	*S/M*	*S/M*	*S/M*	*S/M*	*S/M*
RGS	*S/M*	*S*	*S/M*	*S/M*	-	*S/M*	*S*	*S/M*	*S/M*	*S/M*
Glyco_transf_8	*S/M*	*S*	*S*	*S/M*	*S*	*S/M*	*S/M*	*S/M*	*S/M*	*S/M*
CoaE	*M*	*S*	*S*	*M*	*S*	*S/M*	*S/M*	*S/M*	*S/M*	*S/M*
Alpha-amylase	*S/M*	*S*	*S*	*S/M*	-	*S/M*	*S/M*	*S/M*	*S/M*	*S/M*
Abhydrolase_6	*S/M*	*S*	*S*	*S/M*	*S/M*	*S/M*	*S/M*	*S/M*	*S/M*	*S/M*
PA	*S/M*	*S*	*S/M*	*M*	*S/M*	*S/M*	*S/M*	*S/M*	*S/M*	*S/M*
EF_hand_5	*S/M*	*S*	*S/M*	*S*	*S/M*	*S/M*	*S/M*	*S/M*	*S/M*	*S/M*
Peptidase_M20	*S/M*	*S/M*	*S/M*	*S/M*	*S/M*	*S*	*S*	*S*	*S*	*S/M*
M20_dimer	*S/M*	*S/M*	*S/M*	*S/M*	*S/M*	*S*	*S*	*S*	*S*	*S/M*
PALP	*S/M*	*S/M*	*S/M*	*S*	*S/M*	*S*	*S*	*S*	*S*	*S/M*
Pex24p	*M*	*M*	*M*	*S/M*	*S/M*	-	*S*	*S*	*S/M*	*S/M*

S indicates the presence of shared domain in soluble proteins
M indicates the presence of shared domain in membrane proteins
'-' indicates the absence of shared domains in genome

Fasciclin	S/M	M	S/M	S/M	M	S	S	S	S/M	S/M
KASH	-	-	-	M	-	S	S	S	S/M	S/M
DAO	S/M	S	S/M	S/M	S/M	S	S	S	S/M	S/M
Peptidase_C13	S	M	M	S	M	S	-	S	S/M	S/M
AhpC-TSA	S	S/M	S/M	S	S	S	S/M	S	S/M	S/M
UBX	S	S/M	S/M	S	S	S	S	S	S/M	S/M
Cu_amine_oxid	S	-	S	S	-	-	-	-	S/M	S/M
GCIP	S	S	S	-	-	-	-	-	S/M	S/M
GLTP	S	-	S	-	S	S	S	S	S/M	S/M
Cu_amine_oxidN3	S	-	S	S	-	-	-	-	S/M	S/M
Ribonuclease_T2	S	S	S	-	S	S	S	S	M	S/M
MAGE	S	S	S	S	S	-	-	S	S/M	S/M
DUF647	S	-	-	-	-	-	S	S	S/M	S/M
GAS2	S	-	-	-	-	S	S	S	S/M	S/M
RA	S	S	S	S	S	S	S	S	S/M	S/M
CutA1	-	-	-	-	-	S	S	S	S/M	S/M
MBT	-	-	-	-	-	S	S	S	S/M	S/M
Filament	-	-	-	-	-	S	S	S	S/M	S/M
CRAL_TRIO_2	-	S	S	S	-	S	S	S	S/M	S/M
zf-MYND	S	S	S	S	S	S	S	S	S/M	S/M
Phospholip_A2_1	-	-	-	-	-	S	S	S	S/M	S/M
zf-NF-X1	-	S	-	-	-	S	-	S	S/M	S/M
Mab-21	-	-	-	-	-	S	S	S	S/M	S/M
Peptidase_M2	-	-	-	-	-	S	S	S	S/M	S/M
Peptidase_M8	-	-	-	-	-	S	S	S	M	S/M
Methyltransf_11	S	S	S	S	S	S	S	S	S/M	S/M
MH2	-	-	-	-	-	S	S	S	S/M	S/M
MH1	-	-	-	-	-	S	S	S	S/M	S/M
SPARC_Ca_bdg	-	-	-	-	-	S	S	S	S/M	S/M
Carb_kinase	S	S	S	S	S	S	S	S	S/M	S/M
BNIP2	-	-	-	-	-	-	S	S	S/M	S/M
bZIP_1	S	S	S	S	S	S	S	S	S/M	S/M
C4	-	-	-	-	-	S	S	S	S/M	S/M
DUF2181	-	-	-	-	-	S	S	S	S/M	S/M
zf-3CxxC	-	-	-	-	-	S	S	-	S/M	S/M
JmjC	S	S	S	S	S	S	S	S	S/M	S/M
CBM_21	S	S	S	-	-	S	S	S	S/M	S/M
CNH	S	S	S	S	S	S	S	S	S/M	S/M
Cu_amine_oxidN2	S	-	S	S	-	-	-	-	S/M	S/M
DSPc	S	S	S	S	S	S	S	S	S/M	S/M
UBA	S	S	S	S	S	S	S	S	S/M	S/M
Plectin	-	-	-	-	-	-	S	S	S/M	S/M
FYVE	S	S	S	S	S	S	S	S	S/M	S/M
Methyltransf_26	S	S	-	S	S	S	S	S	S/M	S/M
Arf	S	S	S	S	S	S	S	S	S/M	S/M
PTEN_C2	-	-	-	-	-	S	S	S	S/M	S/M
Trypsin_2	S	S	S	S	-	-	S	S	S/M	S/M
zf-C3HC4	S	S	S	S	S	S	S	S	S/M	S/M
DUF2358	-	-	-	-	-	S	S	S	S/M	S/M
zf-B_box	-	-	-	-	-	S	S	S	S/M	S/M
SOCS_box	-	-	-	-	-	S	S	S/M	S/M	S/M
Ephrin	-	-	-	-	-	S	S	M	S/M	S/M
His_Phos_1	S	S	S	S	S	S	S	S/M	S/M	S/M
PG_binding_1	-	-	-	-	-	S	S	S/M	S/M	S/M
PDZ_2	-	-	S	S	S	S	S	S/M	S/M	S/M
CS	S	S	S	S	S	S	S	S/M	S/M	S/M
FGF	-	-	-	-	-	S	S	S/M	S/M	S/M
EF_hand_6	S	-	-	-	S	S	S	S/M	S/M	S/M
Acetyltransf_1	S/M	S	S	S	S	S	S	S/M	S/M	S/M
DUF3448	S/M	S	S	S	S	S	S	S	S/M	S/M
Gal_mutarotas_2	S/M	S	S	M	S	S	S	S	S/M	S/M
TPR_12	S	-	-	S	S	S/M	S	S/M	S/M	S/M
GSHPx	S	S	S	S	S	S/M	S	S/M	S/M	S/M
Carn_acyltransf	S	S	S	-	S	S/M	S	S/M	S/M	S/M
GDPD	S	S	S	S	S	S/M	S	S/M	S/M	S/M
Erf4	S	S	-	S	-	S/M	S	S/M	S/M	S/M
PAS_9	S	-	-	S	-	M	S	S/M	S/M	S/M
GST_N_3	S	S	S	S	-	S/M	S	S	S/M	S/M
Methyltransf_3	S	-	-	S	-	S/M	-	-	S/M	S/M
UMPH-1	-	-	-	-	-	S/M	S	S	S/M	S/M
PDEase_I	S	S	S	-	-	S/M	S	S	S/M	S/M
NDT80_PhoG	S	S	-	-	-	M	-	S	S/M	S/M
DMAP_binding	S	S	S	-	-	M	S	S	S/M	S/M
Kelch_1	S	S	S	S	-	S/M	S	S	S/M	S/M
SCP2	S	-	S	-	S	S/M	S	S	S/M	S/M
Miff	-	-	-	-	-	M	S	S	S/M	S/M
MATH	S	S	-	S	S	S/M	S	S	S/M	S/M
Amino_oxidase	S	S	S	S	S	S/M	S	S	S/M	S/M
Torsin	-	-	-	-	-	S/M	S	S	S/M	S/M
3Beta_HSD	S/M	S	S	S	S	S/M	S	M	S/M	S/M
Amidase	S/M	S	S	S	S	S/M	S	S/M	S/M	S/M
FAD_binding_3	S/M	S	S/M	S	S	S/M	S	S/M	S/M	S/M
FAD_binding_4	S/M	S	S/M	S	S	S/M	S	S	S/M	S/M
GST_C_2	S/M	S	S	S	S	S/M	S	S	S/M	S/M
Glyco_trans_1_4	M	-	S	S	S	M	S	S	M	S/M
FMO-like	S/M	S	S	S	S	S/M	S	S	S/M	S/M
NAD_binding_8	S/M	S	S/M	S	S	S	S/M	S	S/M	S/M
TPR_1	S/M	S	S	S	S	S	S/M	S	S/M	S/M
Abhydrolase_2	S/M	S	S	S	S	S	S/M	S	S	S/M
Sec1	S/M	S	S	S	S	S	S	S	S	S/M
FCH	S	S	S	S	S	S	S/M	S	S	S/M
HR1	S	S	S	S	S	S	M	S	S	S/M
zf-C2H2	S	S	S	S	S	S	S/M	S	S	S/M
Ribosomal_S12	S	S	S	S	S	S	S/M	S	S	S/M
2OG-FeII_Oxy_3	S	S	S	S	S	S	S/M	S	S/M	S/M
HMG_box	S	S	S	S	S	S	S/M	S	S/M	S/M
F420_oxidored	S	S	S	S	S	S	S/M	S	S/M	S/M
Tom37	S	S	S	M	-	S	M	S	S/M	S/M
Glyco_hydro_1	S	-	-	-	-	S	S/M	S	S/M	S/M
PARP	S	-	-	-	-	S	S/M	S	S/M	S/M
Tom37_C	S	S	S	M	-	-	M	S	S/M	S/M
TPR_16	S	M	S	-	-	-	S/M	S	S/M	S/M
ubiquitin	S	S/M	S	S	S	S/M	S/M	S	S/M	S/M
zf-RING_LisH	S	S/M	S	S	-	S	S/M	S	S/M	S/M
ApoO	S	M	S	S	S	S	M	S	S/M	S/M
Myb_DNA-binding	S	S	S	S	S	S/M	S/M	S	S/M	S/M
PLDc_2	S	S	S	S	S	S/M	S/M	S	S/M	S/M
PDZ	S	S	-	-	-	S/M	S/M	S	S/M	S/M
RhoGAP	S	S	S	S	S	S/M	S/M	S	S/M	S/M
Cystatin	-	-	-	-	-	S/M	-	S	S/M	S/M
Transferrin	-	-	-	-	-	-	S/M	S	S/M	S/M
UPF0546	M	-	-	-	-	M	M	S	S/M	S/M
DUF3456	-	-	-	-	-	S/M	S/M	S	S/M	S/M
MRF_C1	-	-	-	-	-	M	-	S	S/M	S/M
Peptidase_S74	-	-	-	-	-	M	-	S	S/M	S/M
AlaDh_PNT_C	S/M	S	S	S	S	S/M	S/M	S	S/M	S/M
LEM	-	-	-	-	-	S/M	S/M	S	S/M	S/M

AlaDh_PNT_N	*S/M*	*S*	*S*	*S*	*S*	*S/M*	*S/M*	*S*	*S/M*	*S/M*
UPF0041	*S/M*	*S/M*	*S*	*M*	*S*	*S/M*	*S/M*	*S*	*S/M*	*S/M*
ECH	*S*	*S*	*S/M*	*S*	*S*	*S*	*S*	*S*	*S*	*S/M*
Mt_ATP-synt_B	*S*	*S*	*M*	*S*	*S*	*S*	*S*	*S*	*S*	*S/M*
MT	*S*	*S*	*S*	*S*	*S*	*S*	*S*	*S*	*S*	*S/M*
SMN	*S*	-	-	-	-	*S*	*S*	*S*	*S*	*S/M*
DHDPS	*S*	-	*S*	-	-	-	*S*	-	*S*	*S/M*
Prefoldin_2	*S*	*S*	*S*	*S*	*S*	*S*	*S*	*S*	*S*	*S/M*
Memo	*S*	*S*	*S*	*S*	*S*	*S*	*S*	*S*	*S*	*S/M*
Microtub_assoc	*S*	-	-	*S*	-	-	*S*	*S*	*S*	*S/M*
FTHFS	*S*	*S*	*S*	*S*	*S*	*S*	*S*	*S*	*S*	*S/M*
Hist_deacetyl	*S*	*S*	*S*	*S*	*S*	*S*	*S*	*S*	*S*	*S/M*
YjeF_N	*S*	*S*	*S*	*S*	*S*	*S*	*S*	*S*	*S*	*S/M*
Arfaptin	-	-	-	-	-	*S*	*S*	*S*	*S*	*S/M*
Profilin	*S*	*S*	*S*	*S*	*S*	*S*	*S*	*S*	*S*	*S/M*
Cullin_binding	*S*	*S*	*S*	*S*	-	*S*	*S*	*S*	*S*	*S/M*
ELMO_CED12	*S*	-	-	-	-	*S*	*S*	*S*	*S*	*S/M*
DNase_II	-	-	-	-	-	*S*	*S*	*S*	*S*	*S/M*
DUF4339	-	-	-	-	-	*S*	*S*	*S*	*S*	*S/M*
4F5	*S*	*S*	-	*S*	-	*S*	*S*	*S*	*S*	*S/M*
CAP_GLY	*S*	*S*	*S*	*S*	*S*	*S*	*S*	*S*	*S*	*S/M*
VIT	*S*	-	-	-	-	-	-	-	*S*	*S/M*
Ribosomal_S8	*S*	*S*	*S*	*S*	*S*	*S*	*S*	*S*	*S*	*S/M*
Myb_DNA-bind_5	-	-	-	-	-	-	*S*	*S*	*S*	*S/M*
DENN	*S*	-	-	*S*	-	*S*	*S*	*S*	*S*	*S/M*
APG6	*S*	*S*	*S*	*S*	*S*	*S*	*S*	*S*	*S*	*S/M*
GCS	*S*	*S*	*S*	*S*	*S*	*S*	*S*	*S*	*S*	*S/M*
SSDP	-	-	-	*S*	-	-	*S*	-	*S*	*S/M*
Citrate_synt	*S*	*S*	*S*	*S*	*S*	*S*	*S*	*S*	*S*	*S/M*
DUF2348	-	-	-	*S*	-	-	-	*S*	*S*	*S/M*
BNR_2	*S*	-	-	-	-	-	-	-	*S*	*S/M*
Proteasom_PSMB	-	-	-	*S*	-	*S*	*S*	*S*	*S*	*S/M*
RasGEF_N	*S*	*S*	*S*	*S*	*S*	*S*	*S*	*S*	*S*	*S/M*
SAND	-	-	-	-	-	*S*	*S*	*S*	*S*	*S/M*
dDENN	*S*	-	-	*S*	-	*S*	*S*	*S*	*S*	*S/M*
THF_DHG_CYH_C	*S*	*S*	*S*	*S*	*S*	*S*	*S*	*S*	*S*	*S/M*
Sec39	*S*	*S*	*S*	*S*	*S*	-	-	-	*S*	*S/M*
Bromodomain	*S*	*S*	*S*	*S*	*S*	*S*	*S*	*S*	*S*	*S/M*
Cmyb_C	-	-	-	-	-	-	*S*	*S*	*S*	*S/M*
Methyltransf_10	*S*	-	*S*	*S*	*S*	*S*	-	*S*	*S*	*S/M*
Ribonuc_red_lgC	*S*	*S*	*S*	*S*	*S*	*S*	*S*	*S*	*S*	*S/M*
Ribonuc_red_lgN	*S*	*S*	*S*	*S*	*S*	*S*	*S*	*S*	*S*	*S/M*
Mlf1IP	-	-	-	-	-	*S*	*S*	*S*	*S*	*S/M*
Thioredoxin_7	*S*	*S*	*S*	*S*	-	*S*	-	*S*	*S*	*S/M*
HELP	-	-	-	-	-	*S*	*S*	*S*	*S*	*S/M*
Ferritin	-	-	-	-	-	*S*	*S*	*S*	*S*	*S/M*
DUF1916	-	-	-	-	-	-	*S*	*S*	*S*	*S/M*
Proteasome	*S*	*S*	*S*	*S*	*S*	*S*	*S*	*S*	*S*	*S/M*
Kelch_6	-	*S*	-	-	*S*	-	*S*	*S*	*S*	*S/M*
Acetyltransf_2	*S*	-	-	-	*S*	-	-	-	*S*	*S/M*
Ndr	-	-	-	-	-	*S*	*S*	*S*	*S*	*S/M*
Glu-tRNAGln	-	-	-	-	-	*S*	*S*	*S*	*S*	*S/M*
Ubiq_cyt_C_chap	-	*S*	*S*	*S*	*S*	*S*	*S*	*S*	*S*	*S/M*
HpcH_HpaI	*S*	-	-	-	*S*	*S*	-	-	*S*	*S/M*
C8	-	-	-	-	-	-	*S*	*S*	*S*	*S/M*
TruB_N	*S*	*S*	*S*	*S*	*S*	*S*	*S*	*S*	*S*	*S/M*
NUP50	-	*S*	*S*	*S*	-	-	-	*S*	*S*	*S/M*
RUN	-	-	-	-	-	*S*	*S*	*S*	*S*	*S/M*
NYN	*S*	-	-	-	-	-	-	-	*S*	*S/M*
UBACT	*S*	*S*	*S*	*S*	*S*	*S*	*S*	*S*	*S*	*S/M*
Proteasome_A_N	*S*	*S*	*S*	*S*	*S*	*S*	*S*	*S*	*S*	*S/M*
Tubulin_C	*S*	*S*	*S*	*S*	*S*	*S*	*S*	*S*	*S*	*S/M*
Choline_kinase	*S*	*S*	*S*	*S*	*S*	*S*	*S*	*S*	*S*	*S/M*
CPSF_A	*S*	*S*	*S*	*S*	*S*	*S*	*S*	*S*	*S*	*S/M*
PFK	*S*	*S*	*S*	*S*	*S*	*S*	*S*	*S*	*S*	*S/M*
Sep15_SelM	-	-	-	-	-	*S*	-	*S*	*S*	*S/M*
Perilipin	-	-	-	-	-	-	*S*	*S*	*S*	*S/M*
Tubulin	*S*	*S*	*S*	*S*	*S*	*S*	*S*	*S*	*S*	*S/M*
RasGEF	*S*	*S*	*S*	*S*	*S*	*S*	*S*	*S*	*S*	*S/M*
FGE-sulfatase	*S*	-	-	*S*	-	-	*S*	*S*	*S*	*S/M*
uDENN	*S*	-	-	*S*	-	*S*	*S*	*S*	*S*	*S/M*
Adenylsucc_synt	*S*	*S*	*S*	*S*	*S*	*S*	*S*	*S*	*S*	*S/M*
Ribosomal_L37ae	*S*	*S*	*S*	*S*	*S*	*S*	*S*	*S*	*S*	*S/M*
THF_DHG_CYH	*S*	*S*	*S*	*S*	*S*	*S*	-	*S*	*S*	*S/M*
DUF3384	*S*	-	*S*	*S*	*S*	-	*S*	*S*	*S*	*S/M*
MMS1_N	*S*	*S*	*S*	*S*	*S*	*S*	*S*	*S*	*S*	*S/M*
FAD_binding_7	*S*	*S*	-	-	*S*	-	*S*	*S*	*S*	*S/M*
PWWP	*S*	*S*	*S*	*S*	*S*	*S*	*S*	*S*	*S*	*S/M*
DKCLD	*S*	*S*	*S*	*S*	*S*	*S*	*S*	*S*	*S*	*S/M*
DUF4205	-	-	-	-	-	-	*S*	*S*	*S*	*S/M*
SM-ATX	*S*	*S*	*S*	-	*S*	*S*	*S*	*S*	*S*	*S/M*
Dak1	*S*	*S*	*S*	*S*	*S*	*S*	-	-	*S*	*S/M*
Vps26	*S*	*S*	*S*	*S*	*S*	*S*	*S*	*S*	*S*	*S/M*
OCIA	-	-	-	-	-	-	*S*	*S*	*S*	*S/M*
THAP	-	-	-	-	-	*S*	*S*	*S*	*S*	*S/M*
COQ7	*S*	*S*	*S*	*S*	*S*	*S*	*S*	*S*	*S*	*S/M*
Cys_knot	-	-	-	-	-	*S*	*S*	*S*	*S*	*S/M*
Agenet	-	-	-	-	-	-	*S*	-	*S*	*S/M*
ParcG	-	-	-	-	-	*S*	*S*	*S*	*S*	*S/M*
Snf7	*S*	*S*	*S*	*S*	*S*	*S*	*S*	*S*	*S*	*S/M*
Tropomyosin	-	-	-	-	-	*S*	*S*	*S*	*S*	*S/M*
DNA_photolyase	*S*	*S*	-	-	-	-	*S*	*S*	*S*	*S/M*
Arylesterase	-	-	-	-	-	*M*	-	-	*S*	*S/M*
ShK	-	-	-	-	-	*S/M*	-	-	*S*	*S/M*
BRCT	*S*	*S*	*S*	*S*	*S*	*S/M*	*S*	*S*	*S*	*S/M*
SET	*S*	*S*	*S*	*S*	*S*	*S/M*	*S*	*S*	*S*	*S/M*
DUF2346	-	-	-	-	-	*S/M*	*S*	*S*	*S*	*S/M*
zf-UBR	*S*	*S*	*S*	*S*	*S*	*S/M*	*S*	*S*	*S*	*S/M*
Metallophos_2	*S*	*S*	*S*	*S*	-	*S/M*	*S*	*S*	*S*	*S/M*
RNase_H	*S*	*S*	*S*	*S*	*S*	*S/M*	*S*	*S*	*S*	*S/M*
rve	-	*S*	-	*S*	*S*	*S/M*	-	-	*S*	*S/M*
MMR_HSR1	*S*	*S*	*S*	*S*	*S*	*S/M*	*S*	*S*	*S*	*S/M*
PRKCSH	*S*	*S*	*S*	*M*	-	*S*	*S*	*S*	*S/M*	*S/M*
RRM_5	*S*	*S*	*S*	*S/M*	*S*	*S*	*S*	*S*	*S*	*S/M*
CTP_synth_N	*S*	*S*	*S*	*S*	*M*	*S*	*S*	*S*	*S*	*S/M*
HA2	*S*	*S*	*S*	*S*	*S/M*	*S*	*S*	*S*	*S*	*S/M*
EF_hand_4	*S*	-	*S*	-	*M*	*S*	*S*	*S*	*S*	*S/M*
UDP-g_GGTase	*M*	*S*	*S*	*S*	*M*	*S*	*S*	*S*	*S*	*S/M*
PP-binding	*S/M*	*S*	*S*	*S/M*	*S/M*	*S*	*S*	*S*	*S*	*S/M*
Glyco_hydro_18	*S/M*	*M*	*S*	-	*S*	*S/M*	*S/M*	*S/M*	*S*	*S/M*
Sulfotransfer_3	*M*	-	-	-	-	*M*	*M*	*M*	*S*	*S/M*
Peptidase_C1	-	-	-	-	-	*S/M*	*S/M*	*S/M*	*S*	*S/M*
G2F	-	-	-	-	-	*M*	*M*	*S*	*S*	*S/M*
OST-HTH	*S*	-	-	-	-	-	*S/M*	*S*	*S*	*S/M*
Pyrid_oxidase_2	*S*	-	*S*	-	*S*	-	*M*	*S*	*S*	*S/M*
Ets	-	-	-	-	-	*S*	*S/M*	*S*	*S*	*S/M*
BCS1_N	*S/M*	*M*	*S*	*M*	*S*	*S*	*M*	*S*	*S*	*S/M*

SelP_N	-	-	-	-	-	-	M	-	S	S/M
TPR_17	-	-	-	-	-	-	-	-	S	S/M
CFC	-	-	-	-	-	-	-	-	S	S/M
DUF737	-	-	-	-	-	-	-	-	S	S/M
DUF1619	-	-	-	-	-	-	-	M	S	S/M
Glyco_hydro_59	-	-	-	-	-	S	-	-	-	S/M
GSDH	-	-	-	-	-	-	-	-	S	S/M
Fox-1_C	-	-	-	-	-	-	-	-	S	S/M
Urotensin_II	-	-	-	-	-	-	-	-	S	S/M
Limkain-b1	-	-	-	-	-	-	-	-	S	S/M
UPF0004	-	-	-	-	-	S	S/M	S/M	S	S/M
fn1	-	-	-	-	-	-	-	-	S	S/M
Mesothelin	-	-	-	-	-	-	-	-	S	S/M
Pur_DNA_glyco	-	-	-	-	-	-	-	-	S	S/M
NMU	-	-	-	-	-	-	-	-	S	S/M
LMSTEN	-	-	-	-	-	-	-	-	S	S/M
Vitellogenin_N	-	-	-	-	-	S	S/M	S/M	S	S/M
DUF1943	-	-	-	-	-	S	S/M	S/M	S	S/M
TB	-	-	-	-	-	-	-	-	S	S/M
NDUF_B12	M	-	-	-	M	S	-	M	S	S/M
Pro-MCH	-	-	-	-	-	-	-	-	S	S/M
Fib_alpha	-	-	-	-	-	-	-	-	S	S/M
ITI_HC_C	-	-	-	-	-	-	-	-	S	S/M
DUF4209	-	-	-	-	-	-	-	-	S	S/M
DUF4174	-	-	-	-	-	-	-	-	S	S/M
ART	-	-	-	-	-	-	-	-	S	S/M
Ependymin	-	-	-	-	-	-	-	-	S	S/M
DUF4371	-	-	-	-	-	-	-	-	S	S/M
LIM	S	S	S	S	S	S	S/M	S/M	S	S/M
CN_hydrolase	S	S	S	S	S	S	S/M	S/M	S	S/M
Myb_DNA-bind_6	S	S	S	S	S	S	S	S/M	S	S/M
A_deaminase	S	S	S	S	S	S	S	S/M	S	S/M
CBM_14	S	-	-	-	-	S	S/M	S/M	S	S/M
RCC1	S	S/M	S	S	S	S	S/M	S/M	S	S/M
TRAM	-	S	-	S	S	S	S/M	S/M	S	S/M
MTP18	M	-	S	-	S	S	M	M	S	S/M
Palm_thioest	S	-	S	M	S	S	S	M	S	S/M
Methyltransf_23	S	S/M	S	S/M	S	S	S	S/M	S	S/M
Rdx	S	-	S	-	S	S	S	S/M	S	S/M
RVT_1	S	S/M	S	S	S	S	S	-	S	S/M
Pyr_redox	S/M	S/M	S/M	S	S/M	S	S	S/M	S/M	S/M
LACT	M	S/M	M	M	S/M	S	S	M	S/M	S/M
NOD	-	-	-	-	-	S/M	S	M	S/M	S/M
TGF_beta	-	-	-	-	-	S/M	S	S/M	S/M	S/M
Nexin_C	M	M	M	M	M	S/M	S	M	S/M	S/M
A_deaminase_N	-	-	-	-	-	-	S	S/M	-	S/M
BNIP3	-	-	-	-	-	M	S	M	S/M	S/M
NTR	-	-	-	-	-	M	S	S/M	S/M	S/M
SE	M	M	M	M	M	-	-	-	M	S/M
Crisp	-	-	-	-	-	-	S	-	S/M	S/M
Reprolysin_2	-	-	-	-	-	M	S	S/M	S/M	S/M
Peptidase_M10	-	-	-	-	-	S/M	S	S/M	S/M	S/M
Pentaxin	-	-	-	-	-	-	S	-	S/M	S/M
TM231	-	-	-	-	-	M	S	M	M	S/M
Cu2_monooxygen	-	-	-	-	-	S/M	S	S/M	S/M	S/M
FG-GAP	-	-	-	-	-	M	S	M	S/M	S/M
DUF3740	-	-	-	-	-	-	S	M	S/M	S/M
DUF1640	M	S/M	M	M	S/M	-	-	-	M	S/M
NODP	-	-	-	-	-	S/M	S	M	S/M	S/M
Asp_Arg_Hydrox	-	-	-	-	-	M	S	M	S/M	S/M
Syntaxin-6_N	M	M	-	M	M	-	-	M	M	S/M
NDUF_C2	-	-	-	-	-	M	S	M	M	S/M
FERM_N	-	-	-	-	-	S/M	S	S/M	S/M	S/M
Golgin_A5	-	-	-	-	-	S/M	S	S/M	S/M	S/M
Death	-	-	-	-	-	S/M	S	S/M	S/M	S/M
Popeye	-	-	-	-	-	-	S	M	M	S/M
LCCL	M	M	M	-	M	-	-	-	S/M	S/M
DUF3166	-	-	-	-	-	-	S	-	S/M	S/M
Mannosyl_trans2	M	M	-	M	M	M	-	M	M	S/M
Hint	-	-	-	-	-	S/M	S	M	S/M	S/M
wnt	-	-	-	-	-	S/M	S	S/M	S/M	S/M
Hemopexin	-	-	-	-	-	M	S	S/M	S/M	S/M
DUF3454	-	-	-	-	-	-	S	M	S/M	S/M
DUF2404	M	M	S/M	M	M	M	S	M	M	S/M
KCNQ_channel	-	-	-	-	-	M	S	M	M	S/M
TGFb_propeptide	-	-	-	-	-	S/M	S	S/M	S/M	S/M
Ndc1_Nup	M	M	M	M	M	M	-	M	M	S/M
HSNSD	-	-	-	-	-	M	S	M	S/M	S/M
CaMBD	-	-	-	-	-	M	S	M	M	S/M
Cu2_monoox_C	-	-	-	-	-	S/M	S	S/M	S/M	S/M
MORN	-	-	-	-	-	M	S	S/M	S/M	S/M
COX4	M	M	S/M	M	S	S	S	M	M	S/M
UCH	S/M	S/M	S/M	S/M	S	S/M	S	S/M	S/M	S/M
FAD_binding_1	S/M	S	S	S/M	S/M	S/M	S	S/M	S/M	S/M
Flavodoxin_1	S/M	S/M	S	S/M	S/M	S/M	S	S/M	S/M	S/M
SH3_2	S/M	-	S	-	M	S/M	S	S	S/M	S/M
Abhydrolase_3	S/M	S	S	S/M	S/M	S/M	S	S	S/M	S/M
Sarcoglycan_2	-	-	-	-	-	M	M	M	S/M	S/M
Galactosyl_T	-	-	-	-	S	S/M	S/M	S/M	S/M	S/M
DPPIV_N	S/M	M	M	M	-	S/M	S/M	S/M	S/M	S/M
DUF2668	-	-	-	-	-	-	-	-	S/M	S/M
VWA	-	-	-	-	-	S/M	S/M	M	S/M	S/M
ATP_Ca_trans_C	-	-	-	-	-	M	-	M	M	S/M
VWC	-	-	-	-	-	S/M	S/M	S/M	S/M	S/M
REJ	-	-	-	-	-	-	M	S/M	S/M	S/M
NICE-3	-	-	-	-	-	M	M	M	M	S/M
RVT_thumb	-	-	-	-	-	-	-	-	-	S/M
UPF0560	-	-	-	-	-	-	-	-	S/M	S/M
ICAM_N	-	-	-	-	-	-	-	-	S/M	S/M
Fz	-	-	-	-	-	S/M	S/M	S/M	S/M	S/M
V-set	-	-	-	-	-	S/M	S/M	S/M	S/M	S/M
Notch	-	-	-	-	-	S/M	S/M	M	S/M	S/M
AIG1	-	-	-	-	-	-	-	-	S/M	S/M
FerA	-	-	-	-	-	S/M	-	-	M	S/M
FerB	-	-	-	-	-	S/M	M	-	S/M	S/M
LBR_tudor	-	-	-	-	-	-	-	-	M	S/M
Laminin_N	-	-	-	-	-	S/M	S/M	S/M	S/M	S/M
Fzo_mitofusin	-	-	-	-	-	M	M	S/M	S/M	S/M
FPN1	M	-	-	-	-	M	-	-	M	S/M
Glyco_transf_49	-	-	-	-	-	S/M	M	S/M	M	S/M
CLPTM1	M	-	-	-	-	M	M	M	M	S/M
Exostosin	-	-	-	-	-	S/M	M	M	S/M	S/M
Neur_chan_LBD	-	-	-	-	-	S/M	S/M	S/M	S/M	S/M
SPC25	M	M	M	M	-	M	M	M	M	S/M
ANKH	-	-	-	-	-	-	-	-	M	S/M
Carb_anhydrase	-	-	-	-	-	S/M	S/M	S/M	S/M	S/M
Protocadherin	-	-	-	-	-	-	-	-	S/M	S/M

DUF3399	-	-	-	-	-	M	M	M	M	S/M
UCN2	-	-	-	-	-	-	-	-	S/M	S/M
RYDR_ITPR	-	-	-	-	-	M	M	M	S/M	S/M
DAGK_acc	-	-	-	-	-	S/M	S/M	S/M	S/M	S/M
Lectin_N	-	-	-	-	-	-	-	-	S/M	S/M
LBP_BPI_CETP	-	-	-	-	-	S/M	-	-	S/M	S/M
NHL	-	-	-	-	-	S/M	S/M	S/M	S/M	S/M
V-SNARE	M	M	M	M	S	M	M	M	S/M	S/M
Zona_pellucida	-	-	-	-	-	S/M	S/M	S/M	S/M	S/M
Tmemb_cc2	-	-	-	-	-	M	M	M	S/M	S/M
ST7	-	-	-	-	-	M	M	M	M	S/M
Integrin_alpha2	-	-	-	-	-	M	S/M	M	S/M	S/M
Glycos_transf_2	S/M	M	S/M	S/M	S	S/M	S/M	S/M	S/M	S/M
C2-set	-	-	-	-	-	-	-	-	S/M	S/M
T4_deiodinase	-	-	-	-	-	-	-	-	S/M	S/M
CD45	-	-	-	-	-	-	-	-	M	S/M
Trypsin	-	-	-	-	-	S/M	S/M	S/M	S/M	S/M
GBP_C	-	-	-	-	-	M	-	-	S/M	S/M
IN_DBD_C	-	-	-	-	-	-	-	-	-	S/M
DUF2054	-	-	-	-	-	-	M	M	M	S/M
DUF2053	-	-	-	-	-	M	M	M	M	S/M
BRICHOS	-	-	-	-	-	M	M	M	S/M	S/M
Xlink	-	-	-	-	-	-	-	-	S/M	S/M
MIP	M	M	M	M	-	M	M	M	M	S/M
Bestrophin	S/M	-	-	-	-	M	M	M	M	S/M
Gal_Lectin	-	-	-	-	-	M	S/M	M	S/M	S/M
Laminin_EGF	-	-	-	-	-	S/M	S/M	S/M	S/M	S/M
DUF1973	-	-	-	-	-	-	M	-	S/M	S/M
APP_N	-	-	-	-	-	M	-	M	M	S/M
Sel1	S/M	S/M	-	S/M	S	M	S/M	M	S/M	S/M
Tmemb_18A	-	-	-	-	-	M	M	M	M	S/M
XK-related	-	-	-	-	-	M	M	M	S/M	S/M
Bcl-2	-	-	-	-	-	M	M	M	S/M	S/M
PRY	-	-	-	-	-	-	-	-	S/M	S/M
MRVI1	-	-	-	-	-	-	-	-	S/M	S/M
Sp38	-	-	-	-	-	-	-	-	S/M	S/M
Fringe	M	-	-	-	-	S/M	S/M	S/M	S/M	S/M
MRP	-	-	-	-	-	-	-	-	S/M	S/M
HNOBA	-	-	-	-	-	S/M	S/M	S/M	S/M	S/M
HRM	-	-	-	-	-	M	M	M	S/M	S/M
TRAP_beta	-	-	-	-	-	M	M	M	M	S/M
DUF1370	-	-	-	-	-	-	M	M	M	S/M
CaKB	-	-	-	-	-	-	-	-	S/M	S/M
Calcyon	-	-	-	-	-	-	-	-	S/M	S/M
NCD3G	-	-	-	-	-	S/M	M	M	S/M	S/M
AA_permease_N	-	-	-	-	-	-	-	-	M	S/M
APP_Cu_bd	-	-	-	-	-	M	-	M	M	S/M
Cys_rich_FGFR	-	-	-	-	-	M	M	M	S/M	S/M
Astacin	-	-	-	-	-	S/M	S/M	S/M	S/M	S/M
Far-17a_AIG1	M	M	M	M	-	M	M	M	M	S/M
Metallophos	S/M	S/M	S/M	S/M	S	S/M	S/M	S/M	S/M	S/M
DUF3497	-	-	-	-	-	M	M	M	M	S/M
BK_channel_a	-	-	-	-	-	M	M	M	S/M	S/M
S_100	-	-	-	-	-	-	-	-	S/M	S/M
HERV-K_env_2	-	-	-	-	-	-	-	-	-	S/M
RR_TM4-6	-	-	-	-	-	M	M	M	M	S/M
PKD	-	-	-	-	-	-	-	-	M	S/M
Gla	-	-	-	-	-	-	-	-	S/M	S/M
LBP_BPI_CETP_C	-	-	-	-	-	S/M	-	-	S/M	S/M
APG9	M	M	M	M	-	M	M	M	M	S/M
Prion	-	-	-	-	-	-	-	-	M	S/M
NIDO	-	-	-	-	-	M	M	S/M	S/M	S/M
VKG_Carbox	-	-	-	-	-	-	M	M	M	S/M
Pecanex_C	-	-	-	-	-	M	M	M	S/M	S/M
Orai-1	-	-	-	-	-	M	-	M	S/M	S/M
Integrin_beta	-	-	-	-	-	S/M	S/M	M	S/M	S/M
Ceramidase_alk	M	-	-	-	-	-	S/M	M	M	S/M
Integrin_alpha	-	-	-	-	-	M	-	-	M	S/M
Fmp27_GFWDK	M	S/M	S/M	M	S	M	M	M	S/M	S/M
Ig_3	-	-	-	-	-	M	S/M	M	S/M	S/M
Ig_2	-	-	-	-	-	S/M	S/M	S/M	S/M	S/M
Laminin_G_1	-	-	-	-	-	-	S/M	S/M	S/M	S/M
Ldl_recept_a	-	-	-	-	-	S/M	S/M	S/M	S/M	S/M
Ldl_recept_b	-	-	-	-	-	S/M	S/M	S/M	S/M	S/M
DUF2233	-	-	-	-	-	-	-	-	M	S/M
Lamp	-	-	-	-	-	M	M	M	M	S/M
MNNL	-	-	-	-	-	-	-	M	M	S/M
C5-epim_C	-	-	-	-	-	M	M	M	M	S/M
TGF_beta_GS	-	-	-	-	-	M	M	M	S/M	S/M
ASC	-	-	-	-	-	M	S/M	S/M	S/M	S/M
TFR_dimer	M	M	S/M	M	S	M	-	-	M	S/M
Mit_proteolip	-	-	-	-	-	-	-	-	M	S/M
SEA	-	-	-	-	-	M	M	M	S/M	S/M
BH4	-	-	-	-	-	M	-	-	S/M	S/M
GnHR_trans	-	-	-	-	-	-	-	-	M	S/M
MOSC_N	S/M	-	S/M	-	-	S/M	S/M	S/M	S/M	S/M
TRIC	-	-	-	-	-	M	M	S/M	S/M	S/M
LRR_5	-	-	-	-	-	M	S/M	S/M	S/M	S/M
TRP_2	-	-	-	-	-	M	M	M	S/M	S/M
Hormone_2	-	-	-	-	-	-	-	-	S/M	S/M
Hormone_3	-	-	-	-	-	-	-	-	M	S/M
Flt3_lig	-	-	-	-	-	-	-	-	S/M	S/M
MOSC	S/M	-	S/M	-	-	S/M	S/M	S/M	S/M	S/M
PAN_1	M	-	-	-	-	S/M	S/M	S/M	S/M	S/M
Membralin	-	-	-	-	-	M	M	M	M	S/M
Rib_hydrolayse	-	-	-	-	-	-	-	-	S/M	S/M
Integrin_B_tail	-	-	-	-	-	M	M	M	M	S/M
Gal-3-0_sulfotr	-	-	-	-	-	-	-	-	M	S/M
AAA	S/M	S/M	S/M	S/M	S	S/M	S/M	S/M	S/M	S/M
Lectin_C	-	-	-	-	-	S/M	S/M	S/M	S/M	S/M
DUF3595	-	-	-	-	-	M	M	M	M	S/M
Lig_chan-Glu_bd	-	-	-	-	-	M	M	M	M	S/M
CIMR	-	-	-	-	-	-	M	M	S/M	S/M
Cadherin_2	-	-	-	-	-	-	-	-	S/M	S/M
Calx-beta	-	-	-	-	-	M	M	M	S/M	S/M
Activin_recp	-	-	-	-	-	S/M	M	M	S/M	S/M
DOMON	-	-	-	-	-	S/M	S/M	S/M	S/M	S/M
Cadherin_C	-	-	-	-	-	M	S/M	M	S/M	S/M
NDUF_B5	-	-	-	-	-	M	M	M	S/M	S/M
NDUF_B4	-	-	-	-	-	M	M	M	M	S/M
SOXp	-	-	-	-	-	-	-	-	S/M	S/M
fn2	-	-	-	-	-	-	-	-	S/M	S/M
TSP_1	-	-	-	-	-	S/M	S/M	S/M	S/M	S/M
Granin	-	-	-	-	-	-	-	-	S/M	S/M
GRIM-19	M	-	M	-	S	M	M	M	M	S/M
Meckelin	-	-	-	-	-	M	M	M	-	S/M
Sema	-	-	-	-	-	S/M	S/M	S/M	S/M	S/M

Kua-UEV1_localn	-	-	-	-	-	M	M	M	M	S/M
Tmem26	-	-	-	-	-	M	M	M	M	S/M
SRCR	-	-	-	-	-	M	S/M	S/M	S/M	S/M
IL6Ra-bind	-	-	-	-	-	-	-	-	S/M	S/M
Malectin	-	-	-	-	-	M	M	M	M	S/M
ATP-synt_S1	M	M	M	M	-	M	-	-	S/M	S/M
RAMP4	-	-	-	-	-	M	M	M	M	S/M
GSG-1	-	-	-	-	-	-	-	-	M	S/M
UXS1_N	-	-	-	-	-	-	-	-	M	S/M
Glyco_transf_6	-	-	-	-	-	-	-	-	S/M	S/M
FERM_C	-	-	-	-	-	S/M	S/M	S/M	S/M	S/M
TACI-CRD2	-	-	-	-	-	-	-	-	S/M	S/M
FERM_M	-	-	-	-	-	S/M	S/M	S/M	S/M	S/M
TIL	-	-	-	-	-	S/M	S/M	S/M	S/M	S/M
Laminin_G_2	-	-	-	-	-	S/M	S/M	S/M	S/M	S/M
Syndecan	-	-	-	-	-	M	M	M	S/M	S/M
Sulfotransfer_2	-	-	-	-	-	S/M	S/M	M	S/M	S/M
Sulfotransfer_1	-	-	-	-	-	S/M	S/M	S/M	S/M	S/M
Nicastrin	-	-	-	-	-	M	M	M	M	S/M
Xylo_C	-	-	-	-	-	M	M	M	S/M	S/M
Cadherin_pro	-	-	-	-	-	-	-	-	S/M	S/M
SEFIR	-	-	-	-	-	M	-	-	S/M	S/M
EMI	-	-	-	-	-	-	-	S/M	S/M	S/M
Ins145_P3_rec	-	-	-	-	-	M	S/M	M	S/M	S/M
IFNGR1	-	-	-	-	-	-	-	-	M	S/M
GBP	-	-	-	-	-	M	S/M	M	S/M	S/M
TILa	-	-	-	-	-	-	-	-	S/M	S/M
zf-CDGSH	-	-	-	-	-	S/M	S/M	S/M	S/M	S/M
Anth_Ig	-	-	-	-	-	-	-	-	S/M	S/M
PX	S/M	S/M	S/M	S/M	S	S/M	S/M	S/M	S/M	S/M
Neurexophilin	-	-	-	-	-	-	-	-	S/M	S/M
RINGv	M	M	M	M	-	S/M	M	M	S/M	S/M
Kazal_2	-	-	-	-	-	S/M	S/M	S/M	S/M	S/M
DUF3733	-	-	-	-	-	-	-	-	S/M	S/M
C2-set_2	-	-	-	-	-	M	S/M	S/M	S/M	S/M
Kazal_1	-	-	-	-	-	S/M	S/M	S/M	S/M	S/M
Beta-APP	-	-	-	-	-	-	-	-	M	S/M
RIH_assoc	-	-	-	-	-	M	M	M	M	S/M
NPIP	-	-	-	-	-	-	-	-	-	S/M
PSI	-	-	-	-	-	M	M	M	S/M	S/M
Peptidase_M41	S/M	S/M	S/M	M	S	S/M	M	M	S/M	S/M
Phospholip_B	-	-	-	-	-	S/M	M	M	S/M	S/M
ANP	-	-	-	-	-	-	-	-	S/M	S/M
Glyco_transf_64	-	-	-	-	-	M	M	M	S/M	S/M
ApoL	-	-	-	-	-	M	-	-	S/M	S/M
MRF_C2	-	-	-	-	-	M	-	-	S/M	S/M
ITAM	-	-	-	-	-	-	-	-	M	S/M
Hydrolase_like2	M	M	M	M	-	-	M	M	S/M	S/M
CD99L2	-	-	-	-	-	-	-	-	M	S/M
MHC_II_alpha	-	-	-	-	-	-	-	-	M	S/M
EGF	-	-	-	-	-	S/M	S/M	S/M	S/M	S/M
Defensin_beta_2	-	-	-	-	-	-	-	-	S/M	S/M
SRPRB	M	M	M	M	-	M	M	M	M	S/M
CHGN	-	-	-	-	-	S/M	S/M	S/M	S/M	S/M
OLF	-	-	-	-	-	S/M	M	M	S/M	S/M
TNFR_c6	-	-	-	-	-	-	-	M	S/M	S/M
Apt1	M	S/M	S/M	M	S	M	M	M	M	S/M
AChE_tetra	-	-	-	-	-	-	-	-	S/M	S/M
Cadherin	-	-	-	-	-	M	S/M	S/M	S/M	S/M
Tmemb_55A	-	-	-	-	-	M	M	M	S/M	S/M
C1-set	-	-	-	-	-	-	-	-	S/M	S/M
Longin	S/M	S/M	S/M	S/M	S	S/M	S/M	S/M	S/M	S/M
Latrophilin	-	-	-	-	-	-	-	-	S/M	S/M
Mtc	M	M	M	M	-	M	S/M	M	S/M	S/M
WAP	-	-	-	-	-	S/M	S/M	S/M	S/M	S/M
Heme_oxygenase	-	M	M	-	-	-	M	M	S/M	S/M
TM2	-	-	-	-	-	M	S/M	S/M	M	S/M
Sushi	-	-	-	-	-	S/M	S/M	S/M	S/M	S/M
PIG-P	M	M	M	M	-	M	M	M	M	S/M
BSMAP	-	-	-	-	-	-	-	-	S/M	S/M
PIG-F	M	M	M	M	-	M	M	M	M	S/M
IL8	-	-	-	-	-	-	-	-	S/M	S/M
Bap31	M	M	M	M	S	M	M	M	M	S/M
WSC	S/M	M	M	M	-	M	M	M	S/M	S/M
UPAR_LY6	-	-	-	-	-	-	-	-	S/M	S/M
Cadherin-like	-	-	-	-	-	-	-	-	M	S/M
GNT-I	-	-	-	-	-	M	M	M	S/M	S/M
Band_3_cyto	-	-	-	-	-	M	M	S/M	S/M	S/M
LST1	-	-	-	-	-	-	-	-	M	S/M
LAP1C	-	-	-	-	-	-	-	-	S/M	S/M
SNF	-	-	-	-	-	S/M	M	M	S/M	S/M
EPO_TPO	-	-	-	-	-	-	-	-	S/M	S/M
Lep_receptor_Ig	-	-	-	-	-	-	-	-	S/M	S/M
Neur_chan_memb	-	-	-	-	-	M	S/M	M	M	S/M
MGC-24	-	-	-	-	-	M	-	M	M	S/M
Gly_rich	-	-	-	-	-	-	M	M	S/M	S/M
COX7a	-	-	-	-	-	-	M	M	M	S/M
Man-6-P_recep	M	-	-	M	-	-	-	-	M	S/M
PKD_channel	-	-	-	-	-	M	M	M	M	S/M
Glyco_transf_10	-	-	-	-	-	M	S/M	S/M	S/M	S/M
G8	-	-	-	-	-	-	-	-	S/M	S/M
RAMP	-	-	-	-	-	-	-	-	S/M	S/M
Thymopoietin	-	-	-	-	-	-	-	-	S/M	S/M
UPF0258	-	-	-	-	-	-	-	-	S/M	S/M
CLCA_N	-	-	-	-	-	-	M	-	S/M	S/M
CD225	-	-	-	-	-	-	-	-	M	S/M
DUF423	-	-	-	M	-	M	M	M	M	S/M
MHC_I	-	-	-	-	-	-	-	-	S/M	S/M
PGAP1	M	S/M	S/M	M	S	S/M	M	M	M	S/M
CSF-1	-	-	-	-	-	-	-	-	S/M	S/M
Prion_bPrPp	-	-	-	-	-	-	-	-	M	S/M
EGF_3	-	-	-	-	-	S/M	S/M	S/M	S/M	S/M
EGF_2	-	-	-	-	-	M	S/M	M	S/M	S/M
MHC_II_beta	-	-	-	-	-	-	-	-	M	S/M
DUF2217	-	-	-	-	-	M	M	M	S/M	S/M
PV-1	-	-	-	-	-	-	-	-	M	S/M
TLV_coat	-	-	-	-	-	-	-	-	M	S/M
Tmp39	-	-	-	-	-	M	M	M	S/M	S/M
START	-	-	-	-	-	S/M	S/M	S/M	S/M	S/M
GCC2_GCC3	-	-	-	-	-	S/M	M	M	S/M	S/M
DUF872	-	-	-	-	-	M	M	M	M	S/M
DUF2367	-	-	-	-	-	M	M	M	S/M	S/M
Ribophorin_II	-	M	M	M	-	M	M	M	M	S/M
ADAM_CR	-	-	-	-	-	M	M	S/M	S/M	S/M
MSC	M	M	S/M	M	S	M	M	S/M	M	S/M
Fibrinogen_C	-	-	-	-	-	S/M	S/M	S/M	S/M	S/M
TIR	-	-	-	-	-	M	M	M	S/M	S/M

Ricin_B_lectin	-	-	-	-	-	S/M	S/M	M	S/M	S/M
Rhomboid_SP	-	-	-	-	-	-	-	-	S/M	S/M
AMOP	-	-	-	-	-	M	M	M	S/M	S/M
Folate_rec	-	-	-	-	-	-	-	-	S/M	S/M
Kringle	-	-	-	-	-	S/M	M	M	S/M	S/M
ZU5	-	-	-	-	-	S/M	S/M	S/M	S/M	S/M
WIF	-	-	-	-	-	M	M	S/M	S/M	S/M
Interferon	-	-	-	-	-	-	-	-	S/M	S/M
APP_E2	-	-	-	-	-	M	M	M	M	S/M
CUB	-	-	-	-	-	S/M	S/M	S/M	S/M	S/M
KcnmB2_inactiv	-	-	-	-	-	-	-	-	M	S/M
Shisa	-	-	-	-	-	M	-	-	S/M	S/M
HH_signal	-	-	-	-	-	-	-	M	S/M	S/M
Glyco_transf_7C	-	-	-	-	-	S/M	S/M	M	S/M	S/M
NKAIN	-	-	-	-	-	M	M	M	M	S/M
Folate_carrier	-	-	-	-	-	M	M	M	S/M	S/M
Glyco_transf_7N	-	-	-	-	-	M	S/M	M	S/M	S/M
Tmpp129	-	-	-	-	-	M	M	M	S/M	S/M
AGTRAP	-	-	-	-	-	-	M	M	M	S/M
SBP_bac_3	-	-	-	-	-	M	M	M	M	S/M
GRAM	S/M	S/M	S/M	S/M	S	S/M	S/M	S/M	S/M	S/M
IRK	-	-	-	-	-	M	S/M	M	S/M	S/M
Ten_N	-	-	-	-	-	-	-	-	S/M	S/M
Glyco_transf_29	-	-	-	-	-	-	M	M	S/M	S/M
Integrase_Zn	-	-	-	-	-	-	-	-	-	S/M
DUF3651	-	-	-	-	-	M	-	M	S/M	S/M
AA_permease_C	-	-	-	-	-	M	M	M	M	S/M
TNF	-	-	-	-	-	-	M	M	S/M	S/M
Bombesin	-	-	-	-	-	-	-	-	S/M	S/M
Myelin-PO_C	-	-	-	-	-	-	-	-	M	S/M
UT	-	-	-	-	-	-	-	-	M	S/M
Furin-like	-	-	-	-	-	M	M	S/M	S/M	S/M
DUF1053	-	-	-	-	-	-	M	M	M	S/M
PLAT	-	-	-	-	-	S/M	-	S/M	S/M	S/M
Kunitz_BPTI	-	-	-	-	-	S/M	S/M	S/M	S/M	S/M
Hepsin-SRCR	-	-	-	-	-	-	-	-	M	S/M
Coiled-coil_56	-	-	-	M	-	M	M	M	S/M	S/M
DUF1356	-	-	-	-	-	-	-	-	S/M	S/M
MitoNEET_N	-	-	-	-	-	-	M	M	M	S/M
SCF	-	-	-	-	-	-	-	-	M	S/M
VWD	-	-	-	-	-	S/M	S/M	S/M	S/M	S/M
COLFI	-	-	-	-	-	-	S/M	-	S/M	S/M
SP_C-Propep	-	-	-	-	-	-	-	-	M	S/M
TAFA	-	-	-	-	-	-	-	-	S/M	S/M
MANEC	-	-	-	-	-	-	M	M	S/M	S/M
Tweety	-	-	-	-	-	M	M	M	M	S/M
Tissue_fac	-	-	-	-	-	-	-	-	S/M	S/M
Pep_M12B_propep	-	-	-	-	-	S/M	S/M	S/M	S/M	S/M
Peptidase_S9	S/M	M	M	S/M	S	S/M	S/M	S/M	S/M	S/M
DUF1872	-	-	-	-	-	-	-	-	S/M	S/M
PTP_N	-	-	-	-	-	-	-	-	S/M	S/M
I-set	-	-	-	-	-	S/M	S/M	S/M	S/M	S/M
RyR	-	-	-	-	-	M	M	M	M	S/M
OATP	-	-	-	-	-	M	M	M	S/M	S/M
Na_K-ATPase	-	-	-	-	-	M	S/M	M	S/M	S/M
Interfer-bind	-	-	-	-	-	-	-	-	S/M	S/M
KRAB	-	-	-	-	-	-	-	-	S/M	S/M
CD20	-	-	-	-	-	M	-	M	M	S/M
MACPF	-	-	-	-	-	-	-	-	S/M	S/M
Plexin_cytopl	-	-	-	-	-	M	M	M	S/M	S/M
Galanin	-	-	-	-	-	-	-	-	S/M	S/M
Ephrin_lbd	-	-	-	-	-	M	M	M	S/M	S/M
Choline_transpo	M	M	M	M	-	M	M	M	M	S/M
Defensin_beta	-	-	-	-	-	-	-	-	S/M	S/M
Glyco_transf_54	-	-	-	-	-	-	M	M	S/M	S/M
Thyroglobulin_1	-	-	-	-	-	S/M	S/M	S/M	S/M	S/M
Presenilin	-	-	-	-	-	M	M	M	M	S/M
Branch	-	-	-	-	-	S/M	M	M	S/M	S/M
ANF_receptor	-	-	-	-	-	S/M	S/M	S/M	S/M	S/M
EPTP	-	-	-	-	-	-	-	-	S/M	S/M
RGM_C	-	-	-	-	-	M	-	-	S/M	S/M
RGM_N	-	-	-	-	-	M	-	-	S/M	S/M
Dpy19	-	-	-	-	-	M	-	M	M	S/M
Endomucin	-	-	-	-	-	-	-	-	M	S/M
SDF	-	-	-	-	-	M	M	M	M	S/M
KRTAP	-	-	-	-	-	-	-	-	S/M	S/M
Asp-B-Hydro_N	-	-	-	-	-	-	-	-	S/M	S/M
GVQW	-	-	-	-	-	-	-	-	-	S/M
C1q	-	-	-	-	-	-	-	-	S/M	S/M
Reprolysin	-	-	-	-	-	S/M	S/M	S/M	S/M	S/M
Bravo_FIGEY	-	-	-	-	-	M	M	M	S/M	S/M
Tmemb_161AB	-	-	-	-	-	M	M	M	S/M	S/M
DUF2615	-	-	-	-	-	M	M	M	S/M	S/M
RnaseA	-	-	-	-	-	-	-	-	S/M	S/M
EpoR_lig-bind	-	-	-	-	-	-	-	-	S/M	S/M
P2X_receptor	-	-	-	-	-	-	-	-	S/M	S/M
ig	-	-	-	-	-	S/M	-	S/M	S/M	S/M
PLDc_3	-	-	-	-	-	S/M	M	S/M	M	S/M
Sad1_UNC	S/M	M	M	S/M	S	S/M	S/M	S/M	S/M	S/M
Sarcoglycan_1	-	-	-	-	-	M	S/M	M	S/M	S/M
Receptor_IA-2	-	-	-	-	-	-	-	-	S/M	S/M
Tmemb_9	-	-	-	-	-	M	M	M	S/M	S/M
CUE	S/M	S/M	S/M	S	S	S/M	S/M	S	S/M	S/M
Aldedh	S/M	S/M	S/M	S	S	S/M	S/M	S/M	S/M	S/M
HSP70	S/M	S/M	S/M	S/M	S/M	S/M	S/M	S	S/M	S/M
ThiF	S/M	S/M	S/M	S/M	S/M	S/M	S/M	S	S/M	S/M
Gpi16	M	M	M	M	M	M	M	S	S/M	S/M
Peptidase_S10	S/M	S/M	S/M	S/M	S/M	S/M	S/M	S	S/M	S/M
TPR_11	S/M	S/M	S/M	S/M	S/M	S/M	S/M	S/M	S/M	S/M
Seipin	M	-	-	M	M	M	M	M	M	S/M
Calreticulin	M	M	M	M	M	S/M	S/M	S/M	S/M	S/M
NAD_binding_6	M	M	M	M	S/M	M	M	M	M	S/M
NAD_binding_1	S/M	S/M	S/M	S/M	S/M	S/M	S/M	S/M	S/M	S/M
Cation_efflux	S/M	S/M	M	S/M	M	S/M	M	M	S/M	S/M
Chitin_synth_2	M	M	M	-	M	M	M	M	M	S/M
Cyt-b5	S/M	S/M	S/M	S/M	S/M	S/M	S/M	S/M	S/M	S/M
GDA1_CD39	M	M	M	M	S/M	S/M	M	S/M	S/M	S/M
Sulfate_transp	M	M	M	M	M	M	M	M	M	S/M
ERG4_ERG24	S/M	M	M	M	M	M	M	M	M	S/M
Glycos_transf_1	S/M	S/M	S/M	S/M	S/M	S/M	S/M	S/M	S/M	S/M
DAD	M	M	M	M	M	M	M	M	M	S/M
HAD	S/M	S/M	S/M	S/M	S/M	S/M	S/M	S/M	S/M	S/M
Ceramidase	M	M	M	-	M	M	M	M	M	S/M
Gpi1	M	M	M	M	M	M	M	M	M	S/M
EMP70	M	M	M	M	M	M	M	M	S/M	S/M
Na_H_Exchanger	M	M	M	M	M	M	M	M	M	S/M
Zip	M	M	M	M	M	M	M	M	M	S/M

DUF2370	M	M	M	M	M	-	M	M	M	S/M
Serinc	M	M	M	M	M	M	M	M	S/M	S/M
MIR	M	M	M	M	M	S/M	S/M	S/M	S/M	S/M
SCAMP	-	-	-	-	M	M	M	M	S/M	S/M
TRAM_LAG1_CLN8	M	M	M	M	M	M	M	M	S/M	S/M
Synaptobrevin	S/M	S/M	S/M	S/M	S/M	S/M	S/M	S/M	S/M	S/M
DUF1077	M	M	M	M	M	M	M	M	M	S/M
Lectin_leg-like	M	M	M	M	M	M	M	M	S/M	S/M
Steroid_dh	M	M	M	M	M	M	M	M	M	S/M
Evr1_Alr	S/M	S/M	S/M	S/M	S/M	S/M	S/M	S/M	S/M	S/M
Thioredoxin	S/M	S/M	S/M	S/M	S/M	S/M	S/M	S/M	S/M	S/M
STAS	M	M	M	M	M	M	M	M	M	S/M
Syntaxin	M	M	M	M	M	M	S/M	M	S/M	S/M
HMA	S/M	S/M	S/M	S/M	M	S/M	S/M	S/M	S/M	S/M
Tim17	S/M	S/M	S/M	S/M	S/M	S/M	S/M	S/M	S/M	S/M
Rhomboid	M	M	M	M	M	M	M	M	M	S/M
PQ-loop	M	M	M	M	M	M	M	M	S/M	S/M
ABC_tran	S/M	S/M	S/M	S/M	S/M	S/M	S/M	S/M	S/M	S/M
FA_desaturase	M	M	M	M	M	M	M	S/M	S/M	S/M
Mito_carr	S/M	S/M	S/M	S/M	S/M	S/M	S/M	S/M	S/M	S/M
Nuc_sug_transp	M	-	M	M	M	M	M	M	M	S/M
CDC50	M	M	M	M	M	M	M	M	M	S/M
V-SNARE_C	M	M	M	M	S/M	M	S/M	M	S/M	S/M
Sulfate_tra_GLY	M	M	M	M	M	M	M	M	M	S/M
CBS	S/M	S/M	S/M	S/M	S/M	S/M	S/M	S/M	S/M	S/M
Vma12	M	M	M	M	M	M	M	M	M	S/M
CLN3	M	M	M	M	M	M	M	M	M	S/M
Xan_ur_permease	M	-	M	M	M	M	M	M	M	S/M
DUF747	M	M	M	M	M	M	M	M	M	S/M
ABC_membrane_2	M	S/M	M	-	M	M	M	M	S/M	S/M
DUF21	M	M	M	M	M	M	M	M	S/M	S/M
ER_lumen_recept	M	M	M	M	M	M	M	M	M	S/M
MBOAT	M	M	M	M	M	M	M	M	S/M	S/M
zf-DHHC	M	M	M	M	M	M	M	M	M	S/M
UPF0016	M	M	M	M	M	M	M	M	M	S/M
DnaJ	S/M	S/M	S/M	S/M	S/M	S/M	S/M	S/M	S/M	S/M
V_ATPase_I	M	-	M	-	M	M	M	M	S/M	S/M
HlyIII	M	M	M	M	M	M	M	M	M	S/M
Sec63	S/M	S/M	S/M	S/M	S/M	S/M	S/M	S/M	S/M	S/M
Pkinase	S/M	S/M	S/M	S/M	S/M	S/M	S/M	S/M	S/M	S/M
SPRY	S/M	S/M	S/M	S/M	S/M	S/M	S/M	S/M	S/M	S/M
Glyco_hydro_47	S/M	S/M	S/M	S/M	S/M	S/M	S/M	M	S/M	S/M
AA_permease	M	M	M	M	M	M	M	M	M	S/M
UQ_con	S/M	S/M	S/M	S/M	S/M	S/M	S/M	S/M	S/M	S/M
MFS_1	S/M	M	M	M	M	M	M	M	M	S/M
C2	S/M	S/M	S/M	S/M	S/M	S/M	S/M	S/M	S/M	S/M
Lipid_DES	M	-	-	M	M	M	M	M	S/M	S/M
ORMDL	M	M	M	M	M	-	M	M	M	S/M
Peptidase_M28	S/M	S/M	S/M	M	S/M	S/M	S/M	S/M	S/M	S/M
SNARE	M	S/M	S/M	S/M	S/M	S/M	S/M	S/M	S/M	S/M
Motile_Sperm	-	M	M	M	M	S/M	M	S/M	S/M	S/M
EMP24_GP25L	S/M	M	S/M	M	S/M	S/M	S/M	M	S/M	S/M
Exo_endo_phos	S/M	S/M	S/M	S/M	S/M	S/M	S/M	S/M	S/M	S/M
Voltage_CLC	M	M	M	M	M	M	M	M	M	S/M
UAA	M	M	M	M	M	M	M	M	M	S/M
P_proprotein	M	M	M	M	M	S/M	S/M	S/M	S/M	S/M
Nramp	M	M	M	M	M	M	M	M	M	S/M
Phosphodiest	M	S/M	M	M	M	S/M	M	M	S/M	S/M
PXA	M	S/M	S/M	M	M	M	S/M	M	S/M	S/M
YIF1	M	M	M	M	M	M	M	M	M	S/M
Miro	S/M	M	M	M	M	S/M	S/M	S/M	S/M	S/M
Patatin	S/M	S/M	M	S/M	M	S/M	S/M	S/M	S/M	S/M
DER1	M	M	M	M	M	M	M	M	M	S/M
Na_Ca_ex	M	M	M	M	M	M	M	M	M	S/M
Use1	M	M	M	M	M	M	M	M	S/M	S/M
CDP-OH_P_transf	M	M	M	S/M	S/M	M	S/M	M	M	S/M
adh_short	S/M	S/M	S/M	S/M	S/M	S/M	S/M	S/M	S/M	S/M
ERGIC_N	S/M	M	M	M	M	M	S/M	M	S/M	S/M
Abhydrolase_5	S/M	S/M	S/M	M	S/M	S/M	S/M	S/M	S/M	S/M
E1-E2_ATPase	M	M	M	M	M	M	M	M	S/M	S/M
Cation_ATPase_N	M	M	M	M	M	M	M	M	S/M	S/M
Yos1	-	M	M	M	M	M	M	M	S/M	S/M
COPIIcoated_ERV	S/M	M	M	M	M	M	S/M	M	S/M	S/M
Peptidase_S8	S/M	S/M	S/M	S/M	S/M	S/M	S/M	S/M	S/M	S/M
FAD_binding_6	S/M	S/M	S/M	S/M	S/M	S/M	M	S/M	S/M	S/M
FAD_binding_8	M	M	M	M	S/M	M	M	M	M	S/M
cNMP_binding	S/M	S/M	S/M	S/M	S/M	S/M	S/M	S/M	S/M	S/M
Ribophorin_I	M	M	M	M	M	M	M	M	M	S/M
p450	S/M	S/M	S/M	S/M	S/M	S/M	S/M	S/M	S/M	S/M
Band_7	S/M	S/M	S/M	S/M	S/M	S/M	S/M	S/M	S/M	S/M
Ank_2	S/M	S/M	S/M	S/M	S/M	S/M	S/M	S/M	S/M	S/M
TIG	M	M	M	M	M	S/M	S/M	S/M	S/M	S/M
zf-RING_2	S/M	S/M	S/M	S/M	S/M	S/M	S/M	S/M	S/M	S/M
PRA1	M	M	M	M	M	M	M	M	M	S/M
Per1	M	M	M	M	M	M	M	M	M	S/M
Alg6_Alg8	M	M	M	M	M	M	M	M	M	S/M
Glyco_transf_22	M	M	M	M	M	M	M	M	S/M	S/M
Asp	S/M	S/M	S/M	S/M	S/M	S/M	M	S/M	S/M	S/M
Acyltransferase	S/M	S/M	S/M	M	S/M	S/M	S/M	S/M	S/M	S/M
Endonuclease_NS	M	M	S	M	M	S	S/M	S/M	S/M	S/M
Syja_N	S/M	S/M	S	S/M	S/M	S/M	S/M	S/M	S/M	S/M
AMP-binding	S/M	S/M	S	S/M	S/M	S/M	S/M	S/M	S/M	S/M
RabGAP-TBC	S/M	S/M	S	S/M	S/M	S/M	S/M	S/M	S/M	S/M
PIG-X	M	M	S	M	-	-	M	M	S/M	S/M
Lipase_3	S/M	S/M	S	S	S/M	S/M	M	M	M	S/M
zf-C3HC4_2	S/M	S/M	S	S	S/M	S/M	S/M	S/M	S/M	S/M
Pyr_redox_2	S/M	S/M	S/M	S	S/M	S/M	S/M	S/M	S/M	S/M
zf-C3HC4_3	S/M	S/M	S/M	S	M	S/M	S/M	S/M	S/M	S/M
Pex2_Pex12	S/M	S/M	S	S	S/M	S/M	S/M	S	S/M	S/M
Spectrin	-	-	-	-	-	S	S/M	S	S/M	S/M
Serpin	-	-	-	-	-	S	S/M	S	S/M	S/M
BACK	-	-	-	-	-	S	S/M	S	S/M	S/M
Occludin_ELL	-	-	-	-	-	S	M	S	S/M	S/M
Cu-oxidase_2	S/M	S/M	S/M	S	S/M	S	S/M	S	S/M	S/M
Cu-oxidase_3	S/M	S/M	S	S	S/M	S	S/M	S	S/M	S/M
Glyco_hydro_31	S/M	S	S	S/M	S	S	S/M	S/M	S/M	S/M
Thiol-ester_cl	-	-	-	-	-	S	S/M	S/M	S/M	S/M
Trefoil	-	-	-	-	-	S	M	-	S/M	S/M
Cache_1	-	-	-	-	-	S	S/M	M	S/M	S/M
A2M_N	-	-	-	-	-	S	S/M	S/M	S/M	S/M
CBAH	-	-	-	-	-	S	-	-	S/M	S/M
LRAT	-	-	-	-	-	S	-	-	S/M	S/M
DUF829	S/M	-	-	-	-	S	S/M	S/M	M	S/M
A2M_recep	-	-	-	-	-	S	S/M	S/M	S/M	S/M
FSA_C	-	-	-	-	-	S	M	-	S/M	S/M
A2M_N_2	-	-	-	-	-	S	S/M	S/M	S/M	S/M
P4Ha_N	-	-	-	-	-	S	M	S/M	S/M	S/M

AhpC-TSA_2	-	-	-	-	-	*S*	-	-	*S/M*	*S/M*
VWA_N	-	-	-	-	-	*S*	*S/M*	*M*	*S/M*	*S/M*
MAM	-	-	-	-	-	*S*	*M*	*S/M*	*S/M*	*S/M*
Somatomedin_B	-	-	-	-	-	*S*	*S/M*	*S/M*	*S/M*	*S/M*
WRW	-	-	-	-	-	*S*	*M*	*M*	*M*	*S/M*
MAPEG	*M*	-	-	-	*M*	-	*M*	*M*	*S/M*	*S/M*
Tom7	*M*	*S*	-	*M*	-	*S*	-	*M*	*M*	*S/M*
Fmp27	*M*	*S*	-	*M*	-	-	*M*	*M*	*M*	*S/M*
GDNF	-	-	-	-	-	*S*	*M*	*S/M*	*S/M*	*S/M*
PC-Esterase	-	-	-	-	*M*	*S*	*M*	*M*	*S/M*	*S/M*
A2M_comp	-	-	-	-	-	*S*	*S/M*	*S/M*	*S/M*	*S/M*
cEGF	-	-	-	-	-	*S*	*S/M*	*S/M*	*S/M*	*S/M*
Glyco_hydro_56	-	-	-	-	-	*S*	*M*	-	*S/M*	*S/M*
Arm_2	-	-	-	-	-	*S*	-	-	*S/M*	*S/M*
A2M	-	-	-	-	-	*S*	*S/M*	*S/M*	*S/M*	*S/M*
FA	-	-	-	-	-	*S*	*S/M*	*S/M*	*S/M*	*S/M*
Reeler	-	-	-	-	-	*S*	*S/M*	*S/M*	*S/M*	*S/M*
Glyco_hydro_2_N	*S/M*	-	-	-	-	*S*	*M*	*S/M*	*S/M*	*S/M*
IL17	-	-	-	-	-	*S*	-	-	*S/M*	*S/M*
AAA_4	-	-	-	-	-	*S*	-	-	*S/M*	*S/M*
LRRNT	-	-	-	-	-	*S*	*M*	*S/M*	*S/M*	*S/M*
Filamin	-	-	-	-	-	*S*	*S/M*	*S/M*	*S/M*	*S/M*
UPF0139	-	-	*S*	*M*	-	*S*	*M*	*M*	*S/M*	*S/M*
Alpha_kinase	-	-	-	-	-	*S*	-	-	*S/M*	*S/M*
PEX11	*S/M*	*S*	*S*	*S*	*S*	*S*	*S/M*	*S/M*	*S/M*	*S/M*
DHO_dh	*M*	*S*	*S*	*S*	*M*	*S*	*S/M*	*S/M*	*S/M*	*S/M*
Mpv17_PMP22	*S/M*	*S/M*	*S/M*	*M*	-	*S*	*S/M*	*S/M*	*S/M*	*S/M*
zf-H2C2_2	*S/M*	*S/M*	*S/M*	*S/M*	*S*	*S*	*S/M*	*S/M*	*S/M*	*S/M*
FHA	*S/M*	*S/M*	*S/M*	*S/M*	*S/M*	*S*	*S/M*	*S/M*	*S/M*	*S/M*
HMG-CoA_red	*M*	*M*	*M*	*M*	*M*	*S*	*M*	*M*	*S/M*	*S/M*
PTH2	*M*	*M*	*M*	*M*	*M*	*S*	*S/M*	*S/M*	*S/M*	*S/M*
NDUF_B8	*M*	-	*M*	-	*M*	*S*	*M*	*M*	*S/M*	*S/M*
Pro_isomerase	*S/M*	*S/M*	*S/M*	*S*	*S/M*	*S*	*S/M*	*S/M*	*S/M*	*S/M*
Radical_SAM	*S/M*	*S/M*	*S*	*S*	*S*	*S*	*S/M*	*S/M*	*S/M*	*S/M*
ERG2_Sigma1R	*M*	*M*	*S*	*S*	*M*	*S*	-	-	*M*	*S/M*
Wyosine_form	*M*	*M*	-	*S*	-	-	-	-	*M*	*S/M*
CBM_20	*S/M*	-	-	-	-	*S*	*S*	*S/M*	*S/M*	*S/M*

Supplementary Table S2. Functional enrichment of membrane proteins with shared domains.

Gene ontology terms	Enrichment score	SwissProt ID	Protein name	Shared domains
		ADA12_HUMAN	Disintegrin and metalloproteinase domain-containing protein 12	ADAM_CR
		ADA15_HUMAN	Disintegrin and metalloproteinase domain-containing protein 15	Pep_M12B_propep
		ADAM2_HUMAN	Disintegrin and metalloproteinase domain-containing protein 2	ADAM_CR
		ADAM8_HUMAN	Disintegrin and metalloproteinase domain-containing protein 8	ADAM_CR,Disintegrin
		AMGO1_HUMAN	Amphoterin-induced protein 1	LRR_1,V-set
		AMGO2_HUMAN	Amphoterin-induced protein 2	Ig_2
		AOC3_HUMAN	Membrane primary amine oxidase	Cu_amine_oxidN2,Cu_amine_oxid
		ASTL_HUMAN	Astacin-like metalloendopeptidase	Astacin
		ATP4B_HUMAN	Potassium-transporting ATPase subunit beta	Na_K-ATPase
		ATS13_HUMAN	A disintegrin and metalloproteinase with thrombospondin motifs 13	TSP_1,Reprolysin
		BAI1_HUMAN	Brain-specific angiogenesis inhibitor 1	DUF3497,TSP_1
		BCAM_HUMAN	Basal cell adhesion molecule	C2-set_2,Ig_3,Ig_2
		BOC_HUMAN	Brother of CDO	Ig_2
		C1QR1_HUMAN	Complement component C1q receptor	cEGF
		CAD15_HUMAN	Cadherin-15	Cadherin,Cadherin_C
		CAD17_HUMAN	Cadherin-17	Cadherin
		CAD18_HUMAN	Cadherin-18	Cadherin_C
		CAD19_HUMAN	Cadherin-19	Cadherin,Cadherin_C
		CAD22_HUMAN	Cadherin-22	Cadherin_C
		CAD23_HUMAN	Cadherin-23	Cadherin
		CAD24_HUMAN	Cadherin-24	Cadherin
		CAD26_HUMAN	Cadherin-like protein 26	Cadherin
		CADH2_HUMAN	Cadherin-2	Cadherin_C
		CADH3_HUMAN	Cadherin-3	Cadherin
		CADH6_HUMAN	Cadherin-6	Cadherin
		CADH7_HUMAN	Cadherin-7	Cadherin
		CADM3_HUMAN	Cell adhesion molecule 3	Ig_2
		CD226_HUMAN	CD226 antigen	V-set
		CD22_HUMAN	B-cell receptor CD22	ig
		CD33_HUMAN	Myeloid cell surface antigen CD33	V-set,ig
		CDHR1_HUMAN	Cadherin-related family member 1	Cadherin
		CDHR2_HUMAN	Cadherin-related family member 2	Cadherin
		CDHR3_HUMAN	Cadherin-related family member 3	Cadherin
		CDHR4_HUMAN	Cadherin-related family member 4	Cadherin
		CDON_HUMAN	Cell adhesion molecule-related/down-regulated by oncogenes	I-set,fn3,Ig_2
		CELR1_HUMAN	Cadherin EGF LAG seven-pass G-type receptor 1	Cadherin,Laminin_G_2,EGF
		CELR2_HUMAN	Cadherin EGF LAG seven-pass G-type receptor 2	Cadherin
		CELR3_HUMAN	Cadherin EGF LAG seven-pass G-type receptor 3	DUF3497
		CHL1_HUMAN	Neural cell adhesion molecule L1-like protein	I-set,Ig_2
		CHST4_HUMAN	Carbohydrate sulfotransferase 4	Sulfotransfer_1
		CLC4A_HUMAN	C-type lectin domain family 4 member A	Lectin_C
		CLCA2_HUMAN	Calcium-activated chloride channel regulator 2	VWA_2
		CLM8_HUMAN	CMRF35-like molecule 8	V-set
		CNTP1_HUMAN	Contactin-associated protein 1	Laminin_G_2
		CNTP3_HUMAN	Contactin-associated protein-like 3	Laminin_G_2
		CNTP4_HUMAN	Contactin-associated protein-like 4	Laminin_G_2
		CNTP5_HUMAN	Contactin-associated protein-like 5	Laminin_G_2
		COEA1_HUMAN	Collagen alpha-1(XIV) chain	Collagen,fn3
		CSTN1_HUMAN	Calsyntenin-1	Laminin_G_3
		CUZD1_HUMAN	CUB and zona pellucida-like domain-containing protein 1	CUB
		DCBD1_HUMAN	Discoidin, CUB and LCCL domain-containing protein 1	F5_F8_type_C,CUB
		DDR2_HUMAN	Discoidin domain-containing receptor 2	Pkinase_Tyr
		DSC1_HUMAN	Desmocollin-1	Cadherin,Cadherin_pro,Cadherin_C
		DSC2_HUMAN	Desmocollin-2	Cadherin_C
		DSC3_HUMAN	Desmocollin-3	Cadherin,Cadherin_pro
		DSCAM_HUMAN	Down syndrome cell adhesion molecule	I-set,fn3
		DSCL1_HUMAN	Down syndrome cell adhesion molecule-like protein 1	I-set,Ig_2
		DSG2_HUMAN	Desmoglein-2	Cadherin,Cadherin_C
		DSG4_HUMAN	Desmoglein-4	Cadherin,Cadherin_C
		EGLN_HUMAN	Endoglin	Zona_pellucida
		EMR1_HUMAN	EGF-like module-containing mucin-like hormone receptor-like 1	cEGF
		EPHA3_HUMAN	Ephrin type-A receptor 3	fn3
		EPHA8_HUMAN	Ephrin type-A receptor 8	Pkinase_Tyr
		EPHB3_HUMAN	Ephrin type-B receptor 3	Ephrin_lbd
		ESAM_HUMAN	Endothelial cell-selective adhesion molecule	V-set,Ig_2
		FAT1_HUMAN	Protocadherin Fat 1	Cadherin,EGF_CA
		FAT2_HUMAN	Protocadherin Fat 2	Cadherin
		FAT3_HUMAN	Protocadherin Fat 3	Cadherin
		FAT4_HUMAN	Protocadherin Fat 4	Cadherin
		FGF6_HUMAN	Fibroblast growth factor 6	FGF
		FLRT1_HUMAN	Leucine-rich repeat transmembrane protein FLRT1	LRR_8,LRR_1
		FLRT3_HUMAN	Leucine-rich repeat transmembrane protein FLRT3	LRR_8
		FND3A_HUMAN	Fibronectin type-III domain-containing protein 3A	fn3
		FREM2_HUMAN	FRAS1-related extracellular matrix protein 2	Calx-beta
		GP1BA_HUMAN	Platelet glycoprotein Ib alpha chain	LRRNT
		GPIX_HUMAN	Platelet glycoprotein IX	LRR_8
		GPR98_HUMAN	G-protein coupled receptor 98	Calx-beta,Laminin_G_3
		GPV_HUMAN	Platelet glycoprotein V	LRR_8,LRR_7
		HPLN4_HUMAN	Hyaluronan and proteoglycan link protein 4	Xlink
		ICAM5_HUMAN	Intercellular adhesion molecule 5	Ig_2
		IGS11_HUMAN	Immunoglobulin superfamily member 11	V-set
		ITA10_HUMAN	Integrin alpha-10	Integrin_alpha2
		ITA11_HUMAN	Integrin alpha-11	FG-GAP
		ITA1_HUMAN	Integrin alpha-1	Integrin_alpha2,VWA
		ITA4_HUMAN	Integrin alpha-4	FG-GAP
		ITA6_HUMAN	Integrin alpha-6	Integrin_alpha2
		ITA9_HUMAN	Integrin alpha-9	FG-GAP
		ITAD_HUMAN	Integrin alpha-D	Integrin_alpha2,VWA
		ITAE_HUMAN	Integrin alpha-E	Integrin_alpha2,VWA
		ITAX_HUMAN	Integrin alpha-X	FG-GAP,Integrin_alpha2,VWA
		ITB5_HUMAN	Integrin beta-5	Integrin_B_tail
		ITB6_HUMAN	Integrin beta-6	EGF_2
		JAML1_HUMAN	Junctional adhesion molecule-like	V-set
cell adhesion	9.46E-22	LRFN3_HUMAN	Leucine-rich repeat and fibronectin type-III domain-containing protein 3	LRR_8
		LRRN2_HUMAN	Leucine-rich repeat neuronal protein 2	LRR_8
		LY6D_HUMAN	Lymphocyte antigen 6D	UPAR_LY6
		LY9_HUMAN	T-lymphocyte surface antigen Ly-9	Ig_3
		LYAM2_HUMAN	E-selectin	Sushi
		LYAM3_HUMAN	P-selectin	Sushi
		MEG10_HUMAN	Multiple epidermal growth factor-like domains protein 10	Laminin_EGF
		MEG11_HUMAN	Multiple epidermal growth factor-like domains protein 11	Laminin_EGF
		MPZL3_HUMAN	Myelin protein zero-like protein 3	V-set
		MSLN_HUMAN	Mesothelin	Mesothelin
		MUC16_HUMAN	Mucin-16	SEA
		MUC4_HUMAN	Mucin-4	VWD,NIDO
		NEO1_HUMAN	Neogenin	fn3
		NET1_HUMAN	Netrin-1	Laminin_N
		NPHN_HUMAN	Nephrin	V-set,C2-set_2,fn3,Ig_2
		NRCAM_HUMAN	Neuronal cell adhesion molecule	fn3,Ig_3
		OMGP_HUMAN	Oligodendrocyte-myelin glycoprotein	LRRNT,LRR_4
		OTOR_HUMAN	Otoraplin	SH3_2
		PC11Y_HUMAN	Protocadherin-11 Y-linked	Cadherin_2
		PCD12_HUMAN	Protocadherin-12	Cadherin,Cadherin_2
		PCD16_HUMAN	Protocadherin-16	Cadherin
		PCD23_HUMAN	Protocadherin-23	Cadherin
		PCDA4_HUMAN	Protocadherin alpha-4	Cadherin
		PCDA5_HUMAN	Protocadherin alpha-5	Cadherin
		PCDAA_HUMAN	Protocadherin alpha-10	Cadherin
		PCDAB_HUMAN	Protocadherin alpha-11	Cadherin
		PCDAC_HUMAN	Protocadherin alpha-12	Cadherin
		PCDB1_HUMAN	Protocadherin beta-1	Cadherin
		PCDB3_HUMAN	Protocadherin beta-3	Cadherin,Cadherin_2
		PCDB4_HUMAN	Protocadherin beta-4	Cadherin
		PCDB5_HUMAN	Protocadherin beta-5	Cadherin
		PCDB6_HUMAN	Protocadherin beta-6	Cadherin_2
		PCDB8_HUMAN	Protocadherin beta-8	Cadherin,Cadherin_2
		PCDB9_HUMAN	Protocadherin beta-9	Cadherin_2
		PCDBB_HUMAN	Protocadherin beta-11	Cadherin_2
		PCDBD_HUMAN	Protocadherin beta-13	Cadherin
		PCDBG_HUMAN	Protocadherin beta-16	Cadherin,Cadherin_2

		PCDBI_HUMAN	Putative protocadherin beta-18	Cadherin,Cadherin_2
		PCDC2_HUMAN	Protocadherin alpha-C2	Cadherin
		PCDG2_HUMAN	Protocadherin gamma-A2	Cadherin
		PCDG4_HUMAN	Protocadherin gamma-A4	Cadherin
		PCDG5_HUMAN	Protocadherin gamma-A5	Cadherin
		PCDG6_HUMAN	Protocadherin gamma-A6	Cadherin_2
		PCDG7_HUMAN	Protocadherin gamma-A7	Cadherin
		PCDG8_HUMAN	Protocadherin gamma-A8	Cadherin
		PCDG9_HUMAN	Protocadherin gamma-A9	Cadherin
		PCDGB_HUMAN	Protocadherin gamma-A11	Cadherin
		PCDGC_HUMAN	Protocadherin gamma-A12	Cadherin,Cadherin_2
		PCDGE_HUMAN	Protocadherin gamma-B2	Cadherin
		PCDGF_HUMAN	Protocadherin gamma-B3	Cadherin,Cadherin_2
		PCDGG_HUMAN	Protocadherin gamma-B4	Cadherin
		PCDGH_HUMAN	Protocadherin gamma-B5	Cadherin
		PCDGI_HUMAN	Protocadherin gamma-B6	Cadherin
		PCDGJ_HUMAN	Protocadherin gamma-B7	Cadherin
		PCDGK_HUMAN	Protocadherin gamma-C3	Cadherin
		PCDGL_HUMAN	Protocadherin gamma-C4	Cadherin
		PCDGM_HUMAN	Protocadherin gamma-C5	Cadherin
		PCDH7_HUMAN	Protocadherin-7	Cadherin
		PCDH9_HUMAN	Protocadherin-9	Cadherin
		PECA1_HUMAN	Platelet endothelial cell adhesion molecule	Ig_2
		PK1L1_HUMAN	Polycystic kidney disease protein 1-like 1	REJ,PKD_channel,PKD,PLAT
		PKD1_HUMAN	Polycystin-1	PKD
		PLXC1_HUMAN	Plexin-C1	TIG,PSI
		PTPRF_HUMAN	Receptor-type tyrosine-protein phosphatase F	fn3
		PTPRS_HUMAN	Receptor-type tyrosine-protein phosphatase S	fn3
		PVRL2_HUMAN	Poliovirus receptor-related protein 2	Ig_2
		PVRL3_HUMAN	Poliovirus receptor-related protein 3	V-set
		SDK1_HUMAN	Protein sidekick-1	I-set,fn3,Ig_2
		SDK2_HUMAN	Protein sidekick-2	fn3,Ig_2
		SEM5A_HUMAN	Semaphorin-5A	PSI,TSP_1
		SIG10_HUMAN	Sialic acid-binding Ig-like lectin 10	C2-set_2,Ig_2
		SIG11_HUMAN	Sialic acid-binding Ig-like lectin 11	Ig_2
		SIG12_HUMAN	Sialic acid-binding Ig-like lectin 12	V-set
		SIG14_HUMAN	Sialic acid-binding Ig-like lectin 14	Ig_2
		SIG16_HUMAN	Sialic acid-binding Ig-like lectin 16	I-set,Ig_2
		SIGL5_HUMAN	Sialic acid-binding Ig-like lectin 5	ig,Ig_2
		SIGL6_HUMAN	Sialic acid-binding Ig-like lectin 6	ig
		SIGL7_HUMAN	Sialic acid-binding Ig-like lectin 7	V-set,Ig_2
		SIGL8_HUMAN	Sialic acid-binding Ig-like lectin 8	ig
		SIGL9_HUMAN	Sialic acid-binding Ig-like lectin 9	V-set,ig,Ig_2
		SLAF5_HUMAN	SLAM family member 5	Ig_3
		SLAF7_HUMAN	SLAM family member 7	Ig_3
		SN_HUMAN	Sialoadhesin	C2-set_2,Ig_2
		STAB1_HUMAN	Stabilin-1	EGF_3,Fasciclin
		STAB2_HUMAN	Stabilin-2	EGF_3,Fasciclin
		SUSD5_HUMAN	Sushi domain-containing protein 5	Sushi,Xlink
		TACT_HUMAN	T-cell surface protein tactile	Ig_2
		TEN3_HUMAN	Teneurin-3	Ten_N
		TENA_HUMAN	Tenascin	EGF_2,fn3
		TPBG_HUMAN	Trophoblast glycoprotein	LRR_8
		TYRO3_HUMAN	Tyrosine-protein kinase receptor TYRO3	fn3
		UFO_HUMAN	Tyrosine-protein kinase receptor UFO	Pkinase_Tyr,V-set,fn3,Ig_2
		VCAM1_HUMAN	Vascular cell adhesion protein 1	ig
		ZAN_HUMAN	Zonadhesin	TIL,VWD,TILa,EGF
		ADAM8_HUMAN	Disintegrin and metalloproteinase domain-containing protein 8	ADAM_CR,Disintegrin
		AMGO1_HUMAN	Amphoterin-induced protein 1	LRR_1,V-set
		AMGO2_HUMAN	Amphoterin-induced protein 2	Ig_2
		C1QR1_HUMAN	Complement component C1q receptor	cEGF
		CAD15_HUMAN	Cadherin-15	Cadherin,Cadherin_C
		CAD17_HUMAN	Cadherin-17	Cadherin
		CAD18_HUMAN	Cadherin-18	Cadherin_C
		CAD19_HUMAN	Cadherin-19	Cadherin,Cadherin_C
		CAD22_HUMAN	Cadherin-22	Cadherin_C
		CAD23_HUMAN	Cadherin-23	Cadherin
		CAD24_HUMAN	Cadherin-24	Cadherin
		CAD26_HUMAN	Cadherin-like protein 26	Cadherin
		CADH2_HUMAN	Cadherin-2	Cadherin_C
		CADH3_HUMAN	Cadherin-3	Cadherin
		CADH6_HUMAN	Cadherin-6	Cadherin
		CADH7_HUMAN	Cadherin-7	Cadherin
		CADM3_HUMAN	Cell adhesion molecule 3	Ig_2
		CDHR1_HUMAN	Cadherin-related family member 1	Cadherin
		CDHR2_HUMAN	Cadherin-related family member 2	Cadherin
		CDHR3_HUMAN	Cadherin-related family member 3	Cadherin
		CDHR4_HUMAN	Cadherin-related family member 4	Cadherin
		CELR1_HUMAN	Cadherin EGF LAG seven-pass G-type receptor 1	Cadherin,Laminin_G_2,EGF
		CELR2_HUMAN	Cadherin EGF LAG seven-pass G-type receptor 2	Cadherin
		CELR3_HUMAN	Cadherin EGF LAG seven-pass G-type receptor 3	DUF3497
		CHST4_HUMAN	Carbohydrate sulfotransferase 4	Sulfotransfer_1
		COEA1_HUMAN	Collagen alpha-1(XIV) chain	Collagen,fn3
		CSTN1_HUMAN	Calsyntenin-1	Laminin_G_3
		DSC1_HUMAN	Desmocollin-1	Cadherin,Cadherin_pro,Cadherin_C
		DSC2_HUMAN	Desmocollin-2	Cadherin_C
		DSC3_HUMAN	Desmocollin-3	Cadherin,Cadherin_pro
		DSCL1_HUMAN	Down syndrome cell adhesion molecule-like protein 1	I-set,Ig_2
		DSG2_HUMAN	Desmoglein-2	Cadherin,Cadherin_C
		DSG4_HUMAN	Desmoglein-4	Cadherin,Cadherin_C
		ESAM_HUMAN	Endothelial cell-selective adhesion molecule	V-set,Ig_2
		FAT1_HUMAN	Protocadherin Fat 1	Cadherin,EGF_CA
		FAT2_HUMAN	Protocadherin Fat 2	Cadherin
		FAT3_HUMAN	Protocadherin Fat 3	Cadherin
		FAT4_HUMAN	Protocadherin Fat 4	Cadherin
		FGF6_HUMAN	Fibroblast growth factor 6	FGF
		FND3A_HUMAN	Fibronectin type-III domain-containing protein 3A	fn3
		FREM2_HUMAN	FRAS1-related extracellular matrix protein 2	Calx-beta
		GPR98_HUMAN	G-protein coupled receptor 98	Calx-beta,Laminin_G_3
		ICAM5_HUMAN	Intercellular adhesion molecule 5	Ig_2
		ITA4_HUMAN	Integrin alpha-4	FG-GAP
		ITAD_HUMAN	Integrin alpha-D	Integrin_alpha2,VWA
		JAML1_HUMAN	Junctional adhesion molecule-like	V-set
		LYAM2_HUMAN	E-selectin	Sushi
		LYAM3_HUMAN	P-selectin	Sushi
		MEG10_HUMAN	Multiple epidermal growth factor-like domains protein 10	Laminin_EGF
		MEG11_HUMAN	Multiple epidermal growth factor-like domains protein 11	Laminin_EGF
		NET1_HUMAN	Netrin-1	Laminin_N
cell-cell adhesion	5.62E-12	NRCAM_HUMAN	Neuronal cell adhesion molecule	fn3,Ig_3
		OTOR_HUMAN	Otoraplin	SH3_2
		PC11Y_HUMAN	Protocadherin-11 Y-linked	Cadherin_2
		PCD12_HUMAN	Protocadherin-12	Cadherin,Cadherin_2
		PCD16_HUMAN	Protocadherin-16	Cadherin
		PCD23_HUMAN	Protocadherin-23	Cadherin
		PCDA4_HUMAN	Protocadherin alpha-4	Cadherin
		PCDA5_HUMAN	Protocadherin alpha-5	Cadherin
		PCDAA_HUMAN	Protocadherin alpha-10	Cadherin
		PCDAB_HUMAN	Protocadherin alpha-11	Cadherin
		PCDAC_HUMAN	Protocadherin alpha-12	Cadherin
		PCDB1_HUMAN	Protocadherin beta-1	Cadherin
		PCDB3_HUMAN	Protocadherin beta-3	Cadherin,Cadherin_2
		PCDB4_HUMAN	Protocadherin beta-4	Cadherin
		PCDB5_HUMAN	Protocadherin beta-5	Cadherin
		PCDB6_HUMAN	Protocadherin beta-6	Cadherin_2
		PCDB8_HUMAN	Protocadherin beta-8	Cadherin,Cadherin_2
		PCDB9_HUMAN	Protocadherin beta-9	Cadherin_2
		PCDBB_HUMAN	Protocadherin beta-11	Cadherin_2
		PCDBD_HUMAN	Protocadherin beta-13	Cadherin
		PCDBG_HUMAN	Protocadherin beta-16	Cadherin,Cadherin_2
		PCDBI_HUMAN	Putative protocadherin beta-18	Cadherin,Cadherin_2
		PCDC2_HUMAN	Protocadherin alpha-C2	Cadherin
		PCDG2_HUMAN	Protocadherin gamma-A2	Cadherin

		PCDG4_HUMAN	Protocadherin gamma-A4	Cadherin
		PCDG5_HUMAN	Protocadherin gamma-A5	Cadherin
		PCDG6_HUMAN	Protocadherin gamma-A6	Cadherin_2
		PCDG7_HUMAN	Protocadherin gamma-A7	Cadherin
		PCDG8_HUMAN	Protocadherin gamma-A8	Cadherin
		PCDG9_HUMAN	Protocadherin gamma-A9	Cadherin
		PCDGB_HUMAN	Protocadherin gamma-A11	Cadherin
		PCDGC_HUMAN	Protocadherin gamma-A12	Cadherin,Cadherin_2
		PCDGE_HUMAN	Protocadherin gamma-B2	Cadherin
		PCDGF_HUMAN	Protocadherin gamma-B3	Cadherin,Cadherin_2
		PCDGG_HUMAN	Protocadherin gamma-B4	Cadherin
		PCDGH_HUMAN	Protocadherin gamma-B5	Cadherin
		PCDGI_HUMAN	Protocadherin gamma-B6	Cadherin
		PCDGJ_HUMAN	Protocadherin gamma-B7	Cadherin
		PCDGK_HUMAN	Protocadherin gamma-C3	Cadherin
		PCDGL_HUMAN	Protocadherin gamma-C4	Cadherin
		PCDGM_HUMAN	Protocadherin gamma-C5	Cadherin
		PCDH7_HUMAN	Protocadherin-7	Cadherin
		PCDH9_HUMAN	Protocadherin-9	Cadherin
		PK1L1_HUMAN	Polycystic kidney disease protein 1-like 1	REJ,PKD_channel,PKD,PLAT
		PKD1_HUMAN	Polycystin-1	PKD
		PVRL2_HUMAN	Poliovirus receptor-related protein 2	Ig_2
		PVRL3_HUMAN	Poliovirus receptor-related protein 3	V-set
		SLAF5_HUMAN	SLAM family member 5	Ig_3
		SN_HUMAN	Sialoadhesin	C2-set_2,Ig_2
		TEN3_HUMAN	Teneurin-3	Ten_N
		TYRO3_HUMAN	Tyrosine-protein kinase receptor TYRO3	fn3
		VCAM1_HUMAN	Vascular cell adhesion protein 1	ig
		ZAN_HUMAN	Zonadhesin	TIL,VWD,TILa,EGF
		ACHB4_HUMAN	Neuronal acetylcholine receptor subunit beta-4	Neur_chan_memb
		ACSL5_HUMAN	Long-chain-fatty-acid--CoA ligase 5	AMP-binding
		ACVL1_HUMAN	Serine/threonine-protein kinase receptor R3	Activin_recp
		ADAM8_HUMAN	Disintegrin and metalloproteinase domain-containing protein 8	ADAM_CR,Disintegrin
		ASPH_HUMAN	Aspartyl/asparaginyl beta-hydroxylase	TPR_16
		BMP10_HUMAN	Bone morphogenetic protein 10	TGFb_propeptide
		CAC1C_HUMAN	Voltage-dependent L-type calcium channel subunit alpha-1C	Ion_trans
		CADH2_HUMAN	Cadherin-2	Cadherin_C
		CD180_HUMAN	CD180 antigen	LRR_8,LRR_1,LRR_4
		CD226_HUMAN	CD226 antigen	V-set
		CD27_HUMAN	CD27 antigen	TNFR_c6
		CD80_HUMAN	T-lymphocyte activation antigen CD80	V-set
		CD8A_HUMAN	T-cell surface glycoprotein CD8 alpha chain	V-set
		CDON_HUMAN	Cell adhesion molecule-related/down-regulated by oncogenes	I-set,fn3,Ig_2
		CLC6A_HUMAN	C-type lectin domain family 6 member A	Lectin_C
		CNGA1_HUMAN	cGMP-gated cation channel alpha-1	Ion_trans
		CP26A_HUMAN	Cytochrome P450 26A1	p450
		CSF1_HUMAN	Macrophage colony-stimulating factor 1	CSF-1
		CSPG4_HUMAN	Chondroitin sulfate proteoglycan 4	Laminin_G_2
		DLK1_HUMAN	Protein delta homolog 1	EGF
		DLK2_HUMAN	Protein delta homolog 2	hEGF
		DLL1_HUMAN	Delta-like protein 1	EGF
		EGF_HUMAN	Pro-epidermal growth factor	EGF_CA,Ldl_recept_b
		EGLN_HUMAN	Endoglin	Zona_pellucida
		ENPP1_HUMAN	Ectonucleotide pyrophosphatase/phosphodiesterase family member 1	Somatomedin_B
		EPHA7_HUMAN	Ephrin type-A receptor 7	fn3,SAM_2
		EPHA8_HUMAN	Ephrin type-A receptor 8	Pkinase_Tyr
		ERN1_HUMAN	Serine/threonine-protein kinase/endoribonuclease IRE1	Pkinase
		FBN2_HUMAN	Fibrillin-2	EGF_CA,TB
		FCERA_HUMAN	High affinity immunoglobulin epsilon receptor subunit alpha	Ig_2
		FGF19_HUMAN	Fibroblast growth factor 19	FGF
		G6PC2_HUMAN	Glucose-6-phosphatase 2	PAP2
		GHRHR_HUMAN	Growth hormone-releasing hormone receptor	HRM
		GLP1R_HUMAN	Glucagon-like peptide 1 receptor	HRM
		GLRA1_HUMAN	Glycine receptor subunit alpha-1	Neur_chan_memb
		GP124_HUMAN	G-protein coupled receptor 124	HRM,LRR_8
		GUC2D_HUMAN	Retinal guanylyl cyclase 1	Pkinase_Tyr,ANF_receptor
		GUC2F_HUMAN	Retinal guanylyl cyclase 2	ANF_receptor
		HG2A_HUMAN	HLA class II histocompatibility antigen gamma chain	Thyroglobulin_1
		HGF_HUMAN	Hepatocyte growth factor	Trypsin
		HTRA4_HUMAN	Serine protease HTRA4	PDZ_2,Kazal_2
		IL18R_HUMAN	Interleukin-18 receptor 1	Ig_3
		IL31R_HUMAN	Interleukin-31 receptor subunit alpha	IL6Ra-bind,fn3
		IL6RA_HUMAN	Interleukin-6 receptor subunit alpha	IL6Ra-bind
		IL6RB_HUMAN	Interleukin-6 receptor subunit beta	fn3
		IL8_HUMAN	Interleukin-8	IL8
		INGR2_HUMAN	Interferon gamma receptor 2	Interfer-bind
		INSR_HUMAN	Insulin receptor	fn3
		ITA1_HUMAN	Integrin alpha-1	Integrin_alpha2,VWA
		ITA6_HUMAN	Integrin alpha-6	Integrin_alpha2
		ITPR2_HUMAN	Inositol 1,4,5-trisphosphate receptor type 2	Ion_trans,Ins145_P3_rec
regulation of signaling	3.11E-11	JAG1_HUMAN	Protein jagged-1	EGF_CA
		KLK5_HUMAN	Kallikrein-5	Trypsin
		KLOTB_HUMAN	Beta-klotho	Glyco_hydro_1
		KLOT_HUMAN	Klotho	Glyco_hydro_1
		LEMD2_HUMAN	LEM domain-containing protein 2	MSC
		LGR5_HUMAN	Leucine-rich repeat-containing G-protein coupled receptor 5	LRR_1,LRR_4,LRR_7
		LGR6_HUMAN	Leucine-rich repeat-containing G-protein coupled receptor 6	LRR_1
		LRIT3_HUMAN	Leucine-rich repeat, immunoglobulin-like domain and transmembrane domain-containing protein 3	LRR_8,I-set
		LRP1_HUMAN	Prolow-density lipoprotein receptor-related protein 1	cEGF,Ldl_recept_b
		LRP8_HUMAN	Low-density lipoprotein receptor-related protein 8	Ldl_recept_b
		LYAM3_HUMAN	P-selectin	Sushi
		MAN1_HUMAN	Inner nuclear membrane protein Man1	LEM
		MIRO2_HUMAN	Mitochondrial Rho GTPase 2	Miro,Ras
		MYOF_HUMAN	Myoferlin	C2
		NAT8L_HUMAN	N-acetylaspartate synthetase	Acetyltransf_1
		NMDE2_HUMAN	Glutamate receptor ionotropic, NMDA 2B	ANF_receptor
		NOTC2_HUMAN	Neurogenic locus notch homolog protein 2	EGF
		NTRK1_HUMAN	High affinity nerve growth factor receptor	Ig_2
		NTRK3_HUMAN	NT-3 growth factor receptor	ig
		P2RX5_HUMAN	P2X purinoceptor 5	P2X_receptor
		P3IP1_HUMAN	Phosphoinositide-3-kinase-interacting protein 1	Kringle
		PGFRB_HUMAN	Platelet-derived growth factor receptor beta	Pkinase_Tyr,ig
		PLA2R_HUMAN	Secretory phospholipase A2 receptor	Lectin_C
		PLCB_HUMAN	1-acyl-sn-glycerol-3-phosphate acyltransferase beta	Acyltransferase
		PTPRJ_HUMAN	Receptor-type tyrosine-protein phosphatase eta	fn3
		PXDN_HUMAN	Peroxidasin homolog	I-set,VWC
		RFNG_HUMAN	Beta-1,3-N-acetylglucosaminyltransferase radical fringe	Fringe
		RHG36_HUMAN	Rho GTPase-activating protein 36	RhoGAP
		RNF43_HUMAN	E3 ubiquitin-protein ligase RNF43	zf-RING_2
		RON_HUMAN	Macrophage-stimulating protein receptor	TIG,Sema
		SFRP1_HUMAN	Secreted frizzled-related protein 1	Fz
		SNX13_HUMAN	Sorting nexin-13	PX
		SNX14_HUMAN	Sorting nexin-14	PX
		SULF2_HUMAN	Extracellular sulfatase Sulf-2	DUF3740
		TAOK2_HUMAN	Serine/threonine-protein kinase TAO2	Pkinase
		TEN1_HUMAN	Teneurin-1	Ten_N
		TLR3_HUMAN	Toll-like receptor 3	LRR_1
		TLR5_HUMAN	Toll-like receptor 5	LRR_1
		TLR7_HUMAN	Toll-like receptor 7	LRR_1
		TLR9_HUMAN	Toll-like receptor 9	LRR_7
		TNF10_HUMAN	Tumor necrosis factor ligand superfamily member 10	TNF
		TNF15_HUMAN	Tumor necrosis factor ligand superfamily member 15	TNF
		TNR1A_HUMAN	Tumor necrosis factor receptor superfamily member 1A	TNFR_c6
		TRI13_HUMAN	E3 ubiquitin-protein ligase TRIM13	zf-C3HC4_2
		TRPM4_HUMAN	Transient receptor potential cation channel subfamily M member 4	Ion_trans
		TYRO3_HUMAN	Tyrosine-protein kinase receptor TYRO3	fn3
		UCN2_HUMAN	Urocortin-2	UCN2
		UFO_HUMAN	Tyrosine-protein kinase receptor UFO	Pkinase_Tyr,V-set,fn3,Ig_2
		UNC5B_HUMAN	Netrin receptor UNC5B	I-set,TSP_1,ZU5
		VGFR2_HUMAN	Vascular endothelial growth factor receptor 2	I-set,V-set
		ZDH17_HUMAN	Palmitoyltransferase ZDHHC17	Ank
		ZNT1_HUMAN	Zinc transporter 1	Cation_efflux

		ZP3_HUMAN	Zona pellucida sperm-binding protein 3	Zona_pellucida
		ADAM8_HUMAN	Disintegrin and metalloproteinase domain-containing protein 8	ADAM_CR,Disintegrin
		AOC3_HUMAN	Membrane primary amine oxidase	Cu_amine_oxidN2,Cu_amine_oxid
		BPI_HUMAN	Bactericidal permeability-increasing protein	LBP_BPI_CETP_C
		C163A_HUMAN	Scavenger receptor cysteine-rich type 1 protein M130	SRCR
		CCL22_HUMAN	C-C motif chemokine 22	IL8
		CCL24_HUMAN	C-C motif chemokine 24	IL8
		CD180_HUMAN	CD180 antigen	LRR_8,LRR_1,LRR_4
		CD19_HUMAN	B-lymphocyte antigen CD19	Ig_2
		CD3Z_HUMAN	T-cell surface glycoprotein CD3 zeta chain	ITAM
		CD80_HUMAN	T-lymphocyte activation antigen CD80	V-set
		CD83_HUMAN	CD83 antigen	V-set
		CD8A_HUMAN	T-cell surface glycoprotein CD8 alpha chain	V-set
		CHST1_HUMAN	Carbohydrate sulfotransferase 1	Sulfotransfer_1
		CHST4_HUMAN	Carbohydrate sulfotransferase 4	Sulfotransfer_1
		CLC10_HUMAN	C-type lectin domain family 10 member A	Lectin_C
		CLC1B_HUMAN	C-type lectin domain family 1 member B	Lectin_C
		CLC2A_HUMAN	C-type lectin domain family 2 member A	Lectin_C
		CLC4A_HUMAN	C-type lectin domain family 4 member A	Lectin_C
		CLC4C_HUMAN	C-type lectin domain family 4 member C	Lectin_C
		CLC4D_HUMAN	C-type lectin domain family 4 member D	Lectin_C
		CLC4K_HUMAN	C-type lectin domain family 4 member K	Lectin_C
		CLC5A_HUMAN	C-type lectin domain family 5 member A	Lectin_C
		CLC6A_HUMAN	C-type lectin domain family 6 member A	Lectin_C
		CLM2_HUMAN	CMRF35-like molecule 2	V-set
		CLM6_HUMAN	CMRF35-like molecule 6	V-set
		CLM7_HUMAN	CMRF35-like molecule 7	V-set
		COL12_HUMAN	Collectin-12	Collagen,Lectin_C
		CR1_HUMAN	Complement receptor type 1	Sushi
		CR2_HUMAN	Complement receptor type 2	Sushi
		CSF1_HUMAN	Macrophage colony-stimulating factor 1	CSF-1
		CST11_HUMAN	Cystatin-11	Cystatin
		CXCL7_HUMAN	Platelet basic protein	IL8
		D107A_HUMAN	Beta-defensin 107	Defensin_beta_2
		DB133_HUMAN	Beta-defensin 133	Defensin_beta_2
		DB134_HUMAN	Beta-defensin 134	Defensin_beta_2
		EGF_HUMAN	Pro-epidermal growth factor	EGF_CA,Ldl_recept_b
		FCERA_HUMAN	High affinity immunoglobulin epsilon receptor subunit alpha	Ig_2
		FCG2A_HUMAN	Low affinity immunoglobulin gamma Fc region receptor II-a	Ig_2
		FCGR1_HUMAN	High affinity immunoglobulin gamma Fc receptor I	ig,Ig_2
		FCN2_HUMAN	Ficolin-2	Collagen,Fibrinogen_C
		FGF19_HUMAN	Fibroblast growth factor 19	FGF
		FGF6_HUMAN	Fibroblast growth factor 6	FGF
		GBRA5_HUMAN	Gamma-aminobutyric acid receptor subunit alpha-5	Neur_chan_memb
		GRAM_HUMAN	Granzyme M	Trypsin
		HG2A_HUMAN	HLA class II histocompatibility antigen gamma chain	Thyroglobulin_1
		HV301_HUMAN	Ig heavy chain V-III region TRO	V-set
		I10R2_HUMAN	Interleukin-10 receptor subunit beta	Tissue_fac
		I17RB_HUMAN	Interleukin-17 receptor B	SEFIR
		I17RE_HUMAN	Interleukin-17 receptor E	SEFIR
		I18RA_HUMAN	Interleukin-18 receptor accessory protein	Ig_2
defense response	2.48E-10	I22R1_HUMAN	Interleukin-22 receptor subunit alpha-1	Tissue_fac
		I27RA_HUMAN	Interleukin-27 receptor subunit alpha	fn3
		IFM1_HUMAN	Interferon-induced transmembrane protein 1	CD225
		IFNK_HUMAN	Interferon kappa	Interferon
		IL25_HUMAN	Interleukin-25	IL17
		IL31R_HUMAN	Interleukin-31 receptor subunit alpha	IL6Ra-bind,fn3
		IL6RA_HUMAN	Interleukin-6 receptor subunit alpha	IL6Ra-bind
		IL8_HUMAN	Interleukin-8	IL8
		ILRL2_HUMAN	Interleukin-1 receptor-like 2	TIR
		ITAX_HUMAN	Integrin alpha-X	FG-GAP,Integrin_alpha2,VWA
		ITB6_HUMAN	Integrin beta-6	EGF_2
		ITPR2_HUMAN	Inositol 1,4,5-trisphosphate receptor type 2	Ion_trans,Ins145_P3_rec
		KI2S5_HUMAN	Killer cell immunoglobulin-like receptor 2DS5	ig
		KLOTB_HUMAN	Beta-klotho	Glyco_hydro_1
		KLOT_HUMAN	Klotho	Glyco_hydro_1
		KLRG1_HUMAN	Killer cell lectin-like receptor subfamily G member 1	Lectin_C
		LY75_HUMAN	Lymphocyte antigen 75	Lectin_C
		LYAM2_HUMAN	E-selectin	Sushi
		LYAM3_HUMAN	P-selectin	Sushi
		MARCO_HUMAN	Macrophage receptor MARCO	Collagen
		MCP_HUMAN	Membrane cofactor protein	Sushi
		MEP1B_HUMAN	Meprin A subunit beta	MAM,MATH
		NCTR2_HUMAN	Natural cytotoxicity triggering receptor 2	V-set
		NKG2C_HUMAN	NKG2-C type II integral membrane protein	Lectin_C
		NMDE2_HUMAN	Glutamate receptor ionotropic, NMDA 2B	ANF_receptor
		NOTC2_HUMAN	Neurogenic locus notch homolog protein 2	EGF
		PA2GE_HUMAN	Group IIE secretory phospholipase A2	Phospholip_A2_1
		PGFRB_HUMAN	Platelet-derived growth factor receptor beta	Pkinase_Tyr,ig
		RON_HUMAN	Macrophage-stimulating protein receptor	TIG,Sema
		SIG14_HUMAN	Sialic acid-binding Ig-like lectin 14	Ig_2
		SIG15_HUMAN	Sialic acid-binding Ig-like lectin 15	Ig_2
		SIG16_HUMAN	Sialic acid-binding Ig-like lectin 16	I-set,Ig_2
		SIRB1_HUMAN	Signal-regulatory protein beta-1	C1-set
		SLAF5_HUMAN	SLAM family member 5	Ig_3
		SLAF7_HUMAN	SLAM family member 7	Ig_3
		SLAF8_HUMAN	SLAM family member 8	Ig_3
		SN_HUMAN	Sialoadhesin	C2-set_2,Ig_2
		STAB1_HUMAN	Stabilin-1	EGF_3,Fasciclin
		STAB2_HUMAN	Stabilin-2	EGF_3,Fasciclin
		TAP1_HUMAN	Antigen peptide transporter 1	ABC_membrane
		TLR3_HUMAN	Toll-like receptor 3	LRR_1
		TLR5_HUMAN	Toll-like receptor 5	LRR_1
		TLR7_HUMAN	Toll-like receptor 7	LRR_1
		TLR8_HUMAN	Toll-like receptor 8	LRR_1
		TLR9_HUMAN	Toll-like receptor 9	LRR_7
		TNR1A_HUMAN	Tumor necrosis factor receptor superfamily member 1A	TNFR_c6
		TNR4_HUMAN	Tumor necrosis factor receptor superfamily member 4	TNFR_c6
		TRIL_HUMAN	TLR4 interactor with leucine rich repeats	LRR_8
		TRML1_HUMAN	Trem-like transcript 1 protein	V-set
		UFO_HUMAN	Tyrosine-protein kinase receptor UFO	Pkinase_Tyr,V-set,fn3,Ig_2
		VCAM1_HUMAN	Vascular cell adhesion protein 1	ig
		ACHB4_HUMAN	Neuronal acetylcholine receptor subunit beta-4	Neur_chan_memb
		ACVL1_HUMAN	Serine/threonine-protein kinase receptor R3	Activin_recp
		ADAM8_HUMAN	Disintegrin and metalloproteinase domain-containing protein 8	ADAM_CR,Disintegrin
		AFG32_HUMAN	AFG3-like protein 2	AAA
		AMGO1_HUMAN	Amphoterin-induced protein 1	LRR_1,V-set
		APLD1_HUMAN	Apolipoprotein L domain-containing protein 1	ApoL
		ASPH_HUMAN	Aspartyl/asparaginyl beta-hydroxylase	TPR_16
		ASTL_HUMAN	Astacin-like metalloendopeptidase	Astacin
		BAI1_HUMAN	Brain-specific angiogenesis inhibitor 1	DUF3497,TSP_1
		BMP10_HUMAN	Bone morphogenetic protein 10	TGFb_propeptide
		BOC_HUMAN	Brother of CDO	Ig_2
		BPI_HUMAN	Bactericidal permeability-increasing protein	LBP_BPI_CETP_C
		CAC1C_HUMAN	Voltage-dependent L-type calcium channel subunit alpha-1C	Ion_trans
		CCL24_HUMAN	C-C motif chemokine 24	IL8
		CD27_HUMAN	CD27 antigen	TNFR_c6
		CD80_HUMAN	T-lymphocyte activation antigen CD80	V-set
		CD83_HUMAN	CD83 antigen	V-set
		CDON_HUMAN	Cell adhesion molecule-related/down-regulated by oncogenes	I-set,fn3,Ig_2
		CELR1_HUMAN	Cadherin EGF LAG seven-pass G-type receptor 1	Cadherin,Laminin_G_2,EGF
		CLC4E_HUMAN	C-type lectin domain family 4 member E	Lectin_C
		CLC5A_HUMAN	C-type lectin domain family 5 member A	Lectin_C
		CLC6A_HUMAN	C-type lectin domain family 6 member A	Lectin_C
		CLC9A_HUMAN	C-type lectin domain family 9 member A	Lectin_C
		CO4A2_HUMAN	Collagen alpha-2(IV) chain	Collagen
		COEA1_HUMAN	Collagen alpha-1(XIV) chain	Collagen,fn3
		CORIN_HUMAN	Atrial natriuretic peptide-converting enzyme	Ldl_recept_a
		CRTAM_HUMAN	Cytotoxic and regulatory T-cell molecule	C2-set_2
		CSF1_HUMAN	Macrophage colony-stimulating factor 1	CSF-1
		DDR2_HUMAN	Discoidin domain-containing receptor 2	Pkinase_Tyr
		DLL1_HUMAN	Delta-like protein 1	EGF

		DLL4_HUMAN	Delta-like protein 4	hEGF
		DOA_HUMAN	HLA class II histocompatibility antigen, DO alpha chain	MHC_II_alpha
		DOPO_HUMAN	Dopamine beta-hydroxylase	DOMON
		DSC2_HUMAN	Desmocollin-2	Cadherin_C
		DSCAM_HUMAN	Down syndrome cell adhesion molecule	I-set,fn3
		DSG2_HUMAN	Desmoglein-2	Cadherin,Cadherin_C
		EGF_HUMAN	Pro-epidermal growth factor	EGF_CA,Ldl_recept_b
		ENPP1_HUMAN	Ectonucleotide pyrophosphatase/phosphodiesterase family member 1	Somatomedin_B
		EPCR_HUMAN	Endothelial protein C receptor	MHC_I
		EPHA3_HUMAN	Ephrin type-A receptor 3	fn3
		EPHB3_HUMAN	Ephrin type-B receptor 3	Ephrin_lbd
		FBN2_HUMAN	Fibrillin-2	EGF_CA,TB
		FCERA_HUMAN	High affinity immunoglobulin epsilon receptor subunit alpha	Ig_2
		GHRHR_HUMAN	Growth hormone-releasing hormone receptor	HRM
		GLRA1_HUMAN	Glycine receptor subunit alpha-1	Neur_chan_memb
		GP124_HUMAN	G-protein coupled receptor 124	HRM,LRR_8
		GP1BA_HUMAN	Platelet glycoprotein Ib alpha chain	LRRNT
		GPV_HUMAN	Platelet glycoprotein V	LRR_8,LRR_7
		HCN4_HUMAN	Potassium/sodium hyperpolarization-activated cyclic nucleotide-gated channel 4	Ion_trans_N
		HG2A_HUMAN	HLA class II histocompatibility antigen gamma chain	Thyroglobulin_1
		HGF_HUMAN	Hepatocyte growth factor	Trypsin
		I12R1_HUMAN	Interleukin-12 receptor subunit beta-1	fn3
		I27RA_HUMAN	Interleukin-27 receptor subunit alpha	fn3
		IFM1_HUMAN	Interferon-induced transmembrane protein 1	CD225
		IFM5_HUMAN	Interferon-induced transmembrane protein 5	CD225
		IL18R_HUMAN	Interleukin-18 receptor 1	Ig_3
		IL6RA_HUMAN	Interleukin-6 receptor subunit alpha	IL6Ra-bind
		IL6RB_HUMAN	Interleukin-6 receptor subunit beta	fn3
		ILRL2_HUMAN	Interleukin-1 receptor-like 2	TIR
		INSR_HUMAN	Insulin receptor	fn3
regulation of multicellular organismal process	1.90E-08	JAG1_HUMAN	Protein jagged-1	EGF_CA
		K0319_HUMAN	Dyslexia-associated protein KIAA0319	REJ
		KLOT_HUMAN	Klotho	Glyco_hydro_1
		LAG3_HUMAN	Lymphocyte activation gene 3 protein	Ig_2
		LIGO1_HUMAN	Leucine-rich repeat and immunoglobulin-like domain-containing nogo receptor-interacting protein 1	LRR_1
		LRC32_HUMAN	Leucine-rich repeat-containing protein 32	LRR_8,LRR_1
		LRC4B_HUMAN	Leucine-rich repeat-containing protein 4B	LRR_8
		LRC4C_HUMAN	Leucine-rich repeat-containing protein 4C	LRR_1
		LRP8_HUMAN	Low-density lipoprotein receptor-related protein 8	Ldl_recept_b
		LTBP3_HUMAN	Latent-transforming growth factor beta-binding protein 3	cEGF,EGF_CA
		LYAM3_HUMAN	P-selectin	Sushi
		MEG10_HUMAN	Multiple epidermal growth factor-like domains protein 10	Laminin_EGF
		MMP20_HUMAN	Matrix metalloproteinase-20	Hemopexin,PG_binding_1
		MUSK_HUMAN	Muscle, skeletal receptor tyrosine-protein kinase	Fz,Ig_2
		NAT8L_HUMAN	N-acetylaspartate synthetase	Acetyltransf_1
		NET1_HUMAN	Netrin-1	Laminin_N
		NMDE2_HUMAN	Glutamate receptor ionotropic, NMDA 2B	ANF_receptor
		NPHN_HUMAN	Nephrin	V-set,C2-set_2,fn3,Ig_2
		NRCAM_HUMAN	Neuronal cell adhesion molecule	fn3,Ig_3
		NTRK1_HUMAN	High affinity nerve growth factor receptor	Ig_2
		NTRK3_HUMAN	NT-3 growth factor receptor	ig
		OMGP_HUMAN	Oligodendrocyte-myelin glycoprotein	LRRNT,LRR_4
		PD1L1_HUMAN	Programmed cell death 1 ligand 1	C2-set_2
		PDE3A_HUMAN	cGMP-inhibited 3',5'-cyclic phosphodiesterase A	PDEase_I
		PGFRB_HUMAN	Platelet-derived growth factor receptor beta	Pkinase_Tyr,ig
		PLCB_HUMAN	1-acyl-sn-glycerol-3-phosphate acyltransferase beta	Acyltransferase
		PLXA4_HUMAN	Plexin-A4	TIG,PSI
		PLXD1_HUMAN	Plexin-D1	TIG,Sema,PSI
		PPAC3_HUMAN	Probable lipid phosphate phosphatase PPAPDC3	PAP2
		PTPRJ_HUMAN	Receptor-type tyrosine-protein phosphatase eta	fn3
		S22A5_HUMAN	Solute carrier family 22 member 5	Sugar_tr
		SCNAA_HUMAN	Sodium channel protein type 10 subunit alpha	Ion_trans
		SEZ6_HUMAN	Seizure protein 6 homolog	Sushi
		SFRP1_HUMAN	Secreted frizzled-related protein 1	Fz
		SIG15_HUMAN	Sialic acid-binding Ig-like lectin 15	Ig_2
		STAB1_HUMAN	Stabilin-1	EGF_3,Fasciclin
		SULF2_HUMAN	Extracellular sulfatase Sulf-2	DUF3740
		TEN3_HUMAN	Teneurin-3	Ten_N
		TIE1_HUMAN	Tyrosine-protein kinase receptor Tie-1	Ig_2
		TLR3_HUMAN	Toll-like receptor 3	LRR_1
		TLR5_HUMAN	Toll-like receptor 5	LRR_1
		TLR7_HUMAN	Toll-like receptor 7	LRR_1
		TLR8_HUMAN	Toll-like receptor 8	LRR_1
		TLR9_HUMAN	Toll-like receptor 9	LRR_7
		TMPS6_HUMAN	Transmembrane protease serine 6	Ldl_recept_a
		TNF15_HUMAN	Tumor necrosis factor ligand superfamily member 15	TNF
		TNR1A_HUMAN	Tumor necrosis factor receptor superfamily member 1A	TNFR_c6
		TNR4_HUMAN	Tumor necrosis factor receptor superfamily member 4	TNFR_c6
		TNR9_HUMAN	Tumor necrosis factor receptor superfamily member 9	TNFR_c6
		TRIL_HUMAN	TLR4 interactor with leucine rich repeats	LRR_8
		TRPM4_HUMAN	Transient receptor potential cation channel subfamily M member 4	Ion_trans
		TRPV2_HUMAN	Transient receptor potential cation channel subfamily V member 2	Ank_2
		TRPV4_HUMAN	Transient receptor potential cation channel subfamily V member 4	Ion_trans
		TSCOT_HUMAN	Thymic stromal cotransporter homolog	MFS_1
		UCN2_HUMAN	Urocortin-2	UCN2
		UFO_HUMAN	Tyrosine-protein kinase receptor UFO	Pkinase_Tyr,V-set,fn3,Ig_2
		VGFR2_HUMAN	Vascular endothelial growth factor receptor 2	I-set,V-set
		VLDLR_HUMAN	Very low-density lipoprotein receptor	Ldl_recept_a
		ZNT1_HUMAN	Zinc transporter 1	Cation_efflux
		ZP3_HUMAN	Zona pellucida sperm-binding protein 3	Zona_pellucida
		ZP4_HUMAN	Zona pellucida sperm-binding protein 4	Zona_pellucida
		ACVL1_HUMAN	Serine/threonine-protein kinase receptor R3	Activin_recp
		ADAM8_HUMAN	Disintegrin and metalloproteinase domain-containing protein 8	ADAM_CR,Disintegrin
		AMGO1_HUMAN	Amphoterin-induced protein 1	LRR_1,V-set
		APLD1_HUMAN	Apolipoprotein L domain-containing protein 1	ApoL
		AT10A_HUMAN	Probable phospholipid-transporting ATPase VA	HAD,E1-E2_ATPase
		BAI1_HUMAN	Brain-specific angiogenesis inhibitor 1	DUF3497,TSP_1
		BMP10_HUMAN	Bone morphogenetic protein 10	TGFb_propeptide
		BOC_HUMAN	Brother of CDO	Ig_2
		CAD15_HUMAN	Cadherin-15	Cadherin,Cadherin_C
		CADH2_HUMAN	Cadherin-2	Cadherin_C
		CCL24_HUMAN	C-C motif chemokine 24	IL8
		CD27_HUMAN	CD27 antigen	TNFR_c6
		CD80_HUMAN	T-lymphocyte activation antigen CD80	V-set
		CD83_HUMAN	CD83 antigen	V-set
		CDON_HUMAN	Cell adhesion molecule-related/down-regulated by oncogenes	I-set,fn3,Ig_2
		CELR1_HUMAN	Cadherin EGF LAG seven-pass G-type receptor 1	Cadherin,Laminin_G_2,EGF
		CO4A2_HUMAN	Collagen alpha-2(IV) chain	Collagen
		COEA1_HUMAN	Collagen alpha-1(XIV) chain	Collagen,fn3
		CSF1_HUMAN	Macrophage colony-stimulating factor 1	CSF-1
		DDR2_HUMAN	Discoidin domain-containing receptor 2	Pkinase_Tyr
		DLK2_HUMAN	Protein delta homolog 2	hEGF
		DLL1_HUMAN	Delta-like protein 1	EGF
		DLL4_HUMAN	Delta-like protein 4	hEGF
		DOA_HUMAN	HLA class II histocompatibility antigen, DO alpha chain	MHC_II_alpha
		DSCAM_HUMAN	Down syndrome cell adhesion molecule	I-set,fn3
		EGF_HUMAN	Pro-epidermal growth factor	EGF_CA,Ldl_recept_b
		ENPP1_HUMAN	Ectonucleotide pyrophosphatase/phosphodiesterase family member 1	Somatomedin_B
		EPHA3_HUMAN	Ephrin type-A receptor 3	fn3
		EPHB3_HUMAN	Ephrin type-B receptor 3	Ephrin_lbd
		FBN2_HUMAN	Fibrillin-2	EGF_CA,TB
		FND3B_HUMAN	Fibronectin type III domain-containing protein 3B	fn3
		GP124_HUMAN	G-protein coupled receptor 124	HRM,LRR_8
		GPR98_HUMAN	G-protein coupled receptor 98	Calx-beta,Laminin_G_3
		HEXB_HUMAN	Beta-hexosaminidase subunit beta	Glyco_hydro_20b
		HG2A_HUMAN	HLA class II histocompatibility antigen gamma chain	Thyroglobulin_1
		HGF_HUMAN	Hepatocyte growth factor	Trypsin
		I12R1_HUMAN	Interleukin-12 receptor subunit beta-1	fn3
		I27RA_HUMAN	Interleukin-27 receptor subunit alpha	fn3
		IFM1_HUMAN	Interferon-induced transmembrane protein 1	CD225
		IFM5_HUMAN	Interferon-induced transmembrane protein 5	CD225
		IL6RA_HUMAN	Interleukin-6 receptor subunit alpha	IL6Ra-bind

		IL6RB_HUMAN	Interleukin-6 receptor subunit beta	fn3
		ILRL2_HUMAN	Interleukin-1 receptor-like 2	TIR
		INSR_HUMAN	Insulin receptor	fn3
		JAG1_HUMAN	Protein jagged-1	EGF_CA
regulation of developmental process	5.79E-07	K0319_HUMAN	Dyslexia-associated protein KIAA0319	REJ
		KLOT_HUMAN	Klotho	Glyco_hydro_1
		LIGO1_HUMAN	Leucine-rich repeat and immunoglobulin-like domain-containing nogo receptor-interacting protein 1	LRR_1
		LRC4B_HUMAN	Leucine-rich repeat-containing protein 4B	LRR_8
		LRC4C_HUMAN	Leucine-rich repeat-containing protein 4C	LRR_1
		LRP8_HUMAN	Low-density lipoprotein receptor-related protein 8	Ldl_recept_b
		LST1_HUMAN	Leukocyte-specific transcript 1 protein	LST1
		LTBP3_HUMAN	Latent-transforming growth factor beta-binding protein 3	cEGF,EGF_CA
		MEG10_HUMAN	Multiple epidermal growth factor-like domains protein 10	Laminin_EGF
		MMP20_HUMAN	Matrix metalloproteinase-20	Hemopexin,PG_binding_1
		MSRE_HUMAN	Macrophage scavenger receptor types I and II	SRCR,Collagen
		MUSK_HUMAN	Muscle, skeletal receptor tyrosine-protein kinase	Fz,Ig_2
		NEO1_HUMAN	Neogenin	fn3
		NET1_HUMAN	Netrin-1	Laminin_N
		NOTC2_HUMAN	Neurogenic locus notch homolog protein 2	EGF
		NRCAM_HUMAN	Neuronal cell adhesion molecule	fn3,Ig_3
		NTRK1_HUMAN	High affinity nerve growth factor receptor	Ig_2
		NTRK3_HUMAN	NT-3 growth factor receptor	ig
		OMGP_HUMAN	Oligodendrocyte-myelin glycoprotein	LRRNT,LRR_4
		PA2GX_HUMAN	Group 10 secretory phospholipase A2	Phospholip_A2_1
		PDE3A_HUMAN	cGMP-inhibited 3',5'-cyclic phosphodiesterase A	PDEase_I
		PDZD8_HUMAN	PDZ domain-containing protein 8	C1_1,PDZ_2
		PGFRB_HUMAN	Platelet-derived growth factor receptor beta	Pkinase_Tyr,ig
		PLXA4_HUMAN	Plexin-A4	TIG,PSI
		PLXD1_HUMAN	Plexin-D1	TIG,Sema,PSI
		PPAC3_HUMAN	Probable lipid phosphate phosphatase PPAPDC3	PAP2
		PTPRQ_HUMAN	Phosphatidylinositol phosphatase PTPRQ	fn3
		SEZ6_HUMAN	Seizure protein 6 homolog	Sushi
		SFRP1_HUMAN	Secreted frizzled-related protein 1	Fz
		SIG15_HUMAN	Sialic acid-binding Ig-like lectin 15	Ig_2
		STAB1_HUMAN	Stabilin-1	EGF_3,Fasciclin
		SYNE3_HUMAN	Nesprin-3	KASH
		TAOK2_HUMAN	Serine/threonine-protein kinase TAO2	Pkinase
		TEN3_HUMAN	Teneurin-3	Ten_N
		TIE1_HUMAN	Tyrosine-protein kinase receptor Tie-1	Ig_2
		TLR3_HUMAN	Toll-like receptor 3	LRR_1
		TNR1A_HUMAN	Tumor necrosis factor receptor superfamily member 1A	TNFR_c6
		TRPV2_HUMAN	Transient receptor potential cation channel subfamily V member 2	Ank_2
		TRPV4_HUMAN	Transient receptor potential cation channel subfamily V member 4	Ion_trans
		TSCOT_HUMAN	Thymic stromal cotransporter homolog	MFS_1
		UFO_HUMAN	Tyrosine-protein kinase receptor UFO	Pkinase_Tyr,V-set,fn3,Ig_2
		USH2A_HUMAN	Usherin	Laminin_N,Laminin_G_2,fn3,Laminin_G_3,Laminin_EGF
		VGFR2_HUMAN	Vascular endothelial growth factor receptor 2	I-set,V-set
		VLDLR_HUMAN	Very low-density lipoprotein receptor	Ldl_recept_a
		ZP3_HUMAN	Zona pellucida sperm-binding protein 3	Zona_pellucida
		1A80_HUMAN	HLA class I histocompatibility antigen, A-80 alpha chain	MHC_I_C
		ADA10_HUMAN	Disintegrin and metalloproteinase domain-containing protein 10	Pep_M12B_propep
		ADAM8_HUMAN	Disintegrin and metalloproteinase domain-containing protein 8	ADAM_CR,Disintegrin
		BPI_HUMAN	Bactericidal permeability-increasing protein	LBP_BPI_CETP_C
		CD19_HUMAN	B-lymphocyte antigen CD19	Ig_2
		CD226_HUMAN	CD226 antigen	V-set
		CD27_HUMAN	CD27 antigen	TNFR_c6
		CD3Z_HUMAN	T-cell surface glycoprotein CD3 zeta chain	ITAM
		CD5_HUMAN	T-cell surface glycoprotein CD5	SRCR
		CD79A_HUMAN	B-cell antigen receptor complex-associated protein alpha chain	V-set
		CD79B_HUMAN	B-cell antigen receptor complex-associated protein beta chain	V-set
		CD80_HUMAN	T-lymphocyte activation antigen CD80	V-set
		CD83_HUMAN	CD83 antigen	V-set
		CD8A_HUMAN	T-cell surface glycoprotein CD8 alpha chain	V-set
		COL12_HUMAN	Collectin-12	Collagen,Lectin_C
		CR1_HUMAN	Complement receptor type 1	Sushi
		CR2_HUMAN	Complement receptor type 2	Sushi
		CRTAM_HUMAN	Cytotoxic and regulatory T-cell molecule	C2-set_2
		CSF1_HUMAN	Macrophage colony-stimulating factor 1	CSF-1
		DLL1_HUMAN	Delta-like protein 1	EGF
		DOA_HUMAN	HLA class II histocompatibility antigen, DO alpha chain	MHC_II_alpha
		EGF_HUMAN	Pro-epidermal growth factor	EGF_CA,Ldl_recept_b
		FCER2_HUMAN	Low affinity immunoglobulin epsilon Fc receptor	Lectin_C
		FCERA_HUMAN	High affinity immunoglobulin epsilon receptor subunit alpha	Ig_2
		FCG2A_HUMAN	Low affinity immunoglobulin gamma Fc region receptor II-a	Ig_2
		FCGR1_HUMAN	High affinity immunoglobulin gamma Fc receptor I	ig,Ig_2
		FCGRB_HUMAN	High affinity immunoglobulin gamma Fc receptor IB	Ig_2
		FCN2_HUMAN	Ficolin-2	Collagen,Fibrinogen_C
		FGF19_HUMAN	Fibroblast growth factor 19	FGF
		FGF6_HUMAN	Fibroblast growth factor 6	FGF
		HG2A_HUMAN	HLA class II histocompatibility antigen gamma chain	Thyroglobulin_1
		HV301_HUMAN	Ig heavy chain V-III region TRO	V-set
		I12R1_HUMAN	Interleukin-12 receptor subunit beta-1	fn3
		I27RA_HUMAN	Interleukin-27 receptor subunit alpha	fn3
		IFM1_HUMAN	Interferon-induced transmembrane protein 1	CD225
		IFNK_HUMAN	Interferon kappa	Interferon
		IL31R_HUMAN	Interleukin-31 receptor subunit alpha	IL6Ra-bind,fn3
		IL6RA_HUMAN	Interleukin-6 receptor subunit alpha	IL6Ra-bind
		IL6RB_HUMAN	Interleukin-6 receptor subunit beta	fn3
		IL8_HUMAN	Interleukin-8	IL8
		ILRL2_HUMAN	Interleukin-1 receptor-like 2	TIR
regulation of immune system process	6.10E-07	INGR2_HUMAN	Interferon gamma receptor 2	Interfer-bind
		ITA4_HUMAN	Integrin alpha-4	FG-GAP
		ITPR2_HUMAN	Inositol 1,4,5-trisphosphate receptor type 2	Ion_trans,Ins145_P3_rec
		JAG1_HUMAN	Protein jagged-1	EGF_CA
		JAML1_HUMAN	Junctional adhesion molecule-like	V-set
		KLOTB_HUMAN	Beta-klotho	Glyco_hydro_1
		KLOT_HUMAN	Klotho	Glyco_hydro_1
		KLRG1_HUMAN	Killer cell lectin-like receptor subfamily G member 1	Lectin_C
		LAG3_HUMAN	Lymphocyte activation gene 3 protein	Ig_2
		LRC32_HUMAN	Leucine-rich repeat-containing protein 32	LRR_8,LRR_1
		LST1_HUMAN	Leukocyte-specific transcript 1 protein	LST1
		LYAM2_HUMAN	E-selectin	Sushi
		LYAM3_HUMAN	P-selectin	Sushi
		MARCO_HUMAN	Macrophage receptor MARCO	Collagen
		MCP_HUMAN	Membrane cofactor protein	Sushi
		PA2GX_HUMAN	Group 10 secretory phospholipase A2	Phospholip_A2_1
		PD1L1_HUMAN	Programmed cell death 1 ligand 1	C2-set_2
		PD1L2_HUMAN	Programmed cell death 1 ligand 2	Ig_2
		PGFRB_HUMAN	Platelet-derived growth factor receptor beta	Pkinase_Tyr,ig
		PTPRJ_HUMAN	Receptor-type tyrosine-protein phosphatase eta	fn3
		PVRL2_HUMAN	Poliovirus receptor-related protein 2	Ig_2
		SFRP1_HUMAN	Secreted frizzled-related protein 1	Fz
		SIG15_HUMAN	Sialic acid-binding Ig-like lectin 15	Ig_2
		SLAF7_HUMAN	SLAM family member 7	Ig_3
		TACT_HUMAN	T-cell surface protein tactile	Ig_2
		TAP1_HUMAN	Antigen peptide transporter 1	ABC_membrane
		TLR3_HUMAN	Toll-like receptor 3	LRR_1
		TLR5_HUMAN	Toll-like receptor 5	LRR_1
		TLR7_HUMAN	Toll-like receptor 7	LRR_1
		TLR8_HUMAN	Toll-like receptor 8	LRR_1
		TLR9_HUMAN	Toll-like receptor 9	LRR_7
		TNF18_HUMAN	Tumor necrosis factor ligand superfamily member 18	TNF
		TNR4_HUMAN	Tumor necrosis factor receptor superfamily member 4	TNFR_c6
		TR13B_HUMAN	Tumor necrosis factor receptor superfamily member 13B	TACI-CRD2
		TRIL_HUMAN	TLR4 interactor with leucine rich repeats	LRR_8
		TRPM4_HUMAN	Transient receptor potential cation channel subfamily M member 4	Ion_trans
		TSCOT_HUMAN	Thymic stromal cotransporter homolog	MFS_1
		TYRO3_HUMAN	Tyrosine-protein kinase receptor TYRO3	fn3
		UFO_HUMAN	Tyrosine-protein kinase receptor UFO	Pkinase_Tyr,V-set,fn3,Ig_2
		VCAM1_HUMAN	Vascular cell adhesion protein 1	ig
		VTCN1_HUMAN	V-set domain-containing T-cell activation inhibitor 1	V-set
		ZP3_HUMAN	Zona pellucida sperm-binding protein 3	Zona_pellucida

		ZP4_HUMAN	Zona pellucida sperm-binding protein 4	Zona_pellucida
		ACHB4_HUMAN	Neuronal acetylcholine receptor subunit beta-4	Neur_chan_memb
		ACVL1_HUMAN	Serine/threonine-protein kinase receptor R3	Activin_recp
		ADA10_HUMAN	Disintegrin and metalloproteinase domain-containing protein 10	Pep_M12B_propep
		ADAM8_HUMAN	Disintegrin and metalloproteinase domain-containing protein 8	ADAM_CR,Disintegrin
		AMD_HUMAN	Peptidyl-glycine alpha-amidating monooxygenase	NHL
		ASPH_HUMAN	Aspartyl/asparaginyl beta-hydroxylase	TPR_16
		AT2B4_HUMAN	Plasma membrane calcium-transporting ATPase 4	Hydrolase,ATP_Ca_trans_C
		BMP10_HUMAN	Bone morphogenetic protein 10	TGFb_propeptide
		CAC1C_HUMAN	Voltage-dependent L-type calcium channel subunit alpha-1C	Ion_trans
		CALY_HUMAN	Neuron-specific vesicular protein calcyon	Calcyon
		CCL24_HUMAN	C-C motif chemokine 24	IL8
		CD27_HUMAN	CD27 antigen	TNFR_c6
		CELR2_HUMAN	Cadherin EGF LAG seven-pass G-type receptor 2	Cadherin
		CELR3_HUMAN	Cadherin EGF LAG seven-pass G-type receptor 3	DUF3497
		CLC4E_HUMAN	C-type lectin domain family 4 member E	Lectin_C
		CLC5A_HUMAN	C-type lectin domain family 5 member A	Lectin_C
		CLC6A_HUMAN	C-type lectin domain family 6 member A	Lectin_C
		CLC9A_HUMAN	C-type lectin domain family 9 member A	Lectin_C
		CORIN_HUMAN	Atrial natriuretic peptide-converting enzyme	Ldl_recept_a
		CRTAM_HUMAN	Cytotoxic and regulatory T-cell molecule	C2-set_2
		CSF1_HUMAN	Macrophage colony-stimulating factor 1	CSF-1
		CTSR1_HUMAN	Cation channel sperm-associated protein 1	Ion_trans
		DDR2_HUMAN	Discoidin domain-containing receptor 2	Pkinase_Tyr
		DLL4_HUMAN	Delta-like protein 4	hEGF
		DRS7C_HUMAN	Dehydrogenase/reductase SDR family member 7C	adh_short
		DSCAM_HUMAN	Down syndrome cell adhesion molecule	I-set,fn3
		EGF_HUMAN	Pro-epidermal growth factor	EGF_CA,Ldl_recept_b
		EGLN_HUMAN	Endoglin	Zona_pellucida
		ENPP1_HUMAN	Ectonucleotide pyrophosphatase/phosphodiesterase family member 1	Somatomedin_B
		ENPP2_HUMAN	Ectonucleotide pyrophosphatase/phosphodiesterase family member 2	Endonuclease_NS
		FCERA_HUMAN	High affinity immunoglobulin epsilon receptor subunit alpha	Ig_2
		FGF19_HUMAN	Fibroblast growth factor 19	FGF
		G6PC2_HUMAN	Glucose-6-phosphatase 2	PAP2
		GHRHR_HUMAN	Growth hormone-releasing hormone receptor	HRM
		GLP1R_HUMAN	Glucagon-like peptide 1 receptor	HRM
		GLRA1_HUMAN	Glycine receptor subunit alpha-1	Neur_chan_memb
		GP124_HUMAN	G-protein coupled receptor 124	HRM,LRR_8
		HAS2_HUMAN	Hyaluronan synthase 2	Chitin_synth_2
		HG2A_HUMAN	HLA class II histocompatibility antigen gamma chain	Thyroglobulin_1
		HGF_HUMAN	Hepatocyte growth factor	Trypsin
		IFM1_HUMAN	Interferon-induced transmembrane protein 1	CD225
		IL18R_HUMAN	Interleukin-18 receptor 1	Ig_3
		IL6RA_HUMAN	Interleukin-6 receptor subunit alpha	IL6Ra-bind
		IL8_HUMAN	Interleukin-8	IL8
		INSR_HUMAN	Insulin receptor	fn3
		ITPR2_HUMAN	Inositol 1,4,5-trisphosphate receptor type 2	Ion_trans,Ins145_P3_rec
regulation of localization	1.35E-06	JAG1_HUMAN	Protein jagged-1	EGF_CA
		JAG2_HUMAN	Protein jagged-2	hEGF,EGF_CA,EGF
		LRC32_HUMAN	Leucine-rich repeat-containing protein 32	LRR_8,LRR_1
		LRP1_HUMAN	Prolow-density lipoprotein receptor-related protein 1	cEGF,Ldl_recept_b
		LYAM2_HUMAN	E-selectin	Sushi
		LYAM3_HUMAN	P-selectin	Sushi
		MSRE_HUMAN	Macrophage scavenger receptor types I and II	SRCR,Collagen
		NAT8L_HUMAN	N-acetylaspartate synthetase	Acetyltransf_1
		NET1_HUMAN	Netrin-1	Laminin_N
		NPHN_HUMAN	Nephrin	V-set,C2-set_2,fn3,Ig_2
		NTRK3_HUMAN	NT-3 growth factor receptor	ig
		P2RX5_HUMAN	P2X purinoceptor 5	P2X_receptor
		PA2GX_HUMAN	Group 10 secretory phospholipase A2	Phospholip_A2_1
		PD1L1_HUMAN	Programmed cell death 1 ligand 1	C2-set_2
		PGFRB_HUMAN	Platelet-derived growth factor receptor beta	Pkinase_Tyr,ig
		PKD1_HUMAN	Polycystin-1	PKD
		PLA2R_HUMAN	Secretory phospholipase A2 receptor	Lectin_C
		PLPL2_HUMAN	Patatin-like phospholipase domain-containing protein 2	Patatin
		PLXA2_HUMAN	Plexin-A2	TIG
		PLXA4_HUMAN	Plexin-A4	TIG,PSI
		PLXD1_HUMAN	Plexin-D1	TIG,Sema,PSI
		PTPRJ_HUMAN	Receptor-type tyrosine-protein phosphatase eta	fn3
		RYR1_HUMAN	Ryanodine receptor 1	SPRY
		S26A6_HUMAN	Solute carrier family 26 member 6	STAS
		SCNAA_HUMAN	Sodium channel protein type 10 subunit alpha	Ion_trans
		SFRP1_HUMAN	Secreted frizzled-related protein 1	Fz
		TEN1_HUMAN	Teneurin-1	Ten_N
		TIE1_HUMAN	Tyrosine-protein kinase receptor Tie-1	Ig_2
		TLR3_HUMAN	Toll-like receptor 3	LRR_1
		TLR5_HUMAN	Toll-like receptor 5	LRR_1
		TLR7_HUMAN	Toll-like receptor 7	LRR_1
		TLR8_HUMAN	Toll-like receptor 8	LRR_1
		TLR9_HUMAN	Toll-like receptor 9	LRR_7
		TNF15_HUMAN	Tumor necrosis factor ligand superfamily member 15	TNF
		TNR1A_HUMAN	Tumor necrosis factor receptor superfamily member 1A	TNFR_c6
		TNR4_HUMAN	Tumor necrosis factor receptor superfamily member 4	TNFR_c6
		TNR9_HUMAN	Tumor necrosis factor receptor superfamily member 9	TNFR_c6
		TPC2_HUMAN	Two pore calcium channel protein 2	Ion_trans
		TRPV2_HUMAN	Transient receptor potential cation channel subfamily V member 2	Ank_2
		TRPV6_HUMAN	Transient receptor potential cation channel subfamily V member 6	Ank_2
		UCN2_HUMAN	Urocortin-2	UCN2
		VGFR2_HUMAN	Vascular endothelial growth factor receptor 2	I-set,V-set
		VMAT2_HUMAN	Synaptic vesicular amine transporter	MFS_1
		ZNT1_HUMAN	Zinc transporter 1	Cation_efflux
		ZNT2_HUMAN	Zinc transporter 2	Cation_efflux
		ZP3_HUMAN	Zona pellucida sperm-binding protein 3	Zona_pellucida
		ZP4_HUMAN	Zona pellucida sperm-binding protein 4	Zona_pellucida
		CD180_HUMAN	CD180 antigen	LRR_8,LRR_1,LRR_4
		CD19_HUMAN	B-lymphocyte antigen CD19	Ig_2
		CD3Z_HUMAN	T-cell surface glycoprotein CD3 zeta chain	ITAM
		CD80_HUMAN	T-lymphocyte activation antigen CD80	V-set
		CLC10_HUMAN	C-type lectin domain family 10 member A	Lectin_C
		CLC2A_HUMAN	C-type lectin domain family 2 member A	Lectin_C
		CLC4A_HUMAN	C-type lectin domain family 4 member A	Lectin_C
		CLC4C_HUMAN	C-type lectin domain family 4 member C	Lectin_C
		CLC4D_HUMAN	C-type lectin domain family 4 member D	Lectin_C
		CLC5A_HUMAN	C-type lectin domain family 5 member A	Lectin_C
		CLC6A_HUMAN	C-type lectin domain family 6 member A	Lectin_C
		CLM2_HUMAN	CMRF35-like molecule 2	V-set
		CLM7_HUMAN	CMRF35-like molecule 7	V-set
		COL12_HUMAN	Collectin-12	Collagen,Lectin_C
		CR1_HUMAN	Complement receptor type 1	Sushi
		CR2_HUMAN	Complement receptor type 2	Sushi
		CSF1_HUMAN	Macrophage colony-stimulating factor 1	CSF-1
		EGF_HUMAN	Pro-epidermal growth factor	EGF_CA,Ldl_recept_b
		FCERA_HUMAN	High affinity immunoglobulin epsilon receptor subunit alpha	Ig_2
		FCG2A_HUMAN	Low affinity immunoglobulin gamma Fc region receptor II-a	Ig_2
		FCGR1_HUMAN	High affinity immunoglobulin gamma Fc receptor I	ig,Ig_2
		FCN2_HUMAN	Ficolin-2	Collagen,Fibrinogen_C
		FGF19_HUMAN	Fibroblast growth factor 19	FGF
		FGF6_HUMAN	Fibroblast growth factor 6	FGF
innate immune response	3.92E-06	GRAM_HUMAN	Granzyme M	Trypsin
		HV301_HUMAN	Ig heavy chain V-III region TRO	V-set
		ITPR2_HUMAN	Inositol 1,4,5-trisphosphate receptor type 2	Ion_trans,Ins145_P3_rec
		KI2S5_HUMAN	Killer cell immunoglobulin-like receptor 2DS5	ig
		KLOTB_HUMAN	Beta-klotho	Glyco_hydro_1
		KLOT_HUMAN	Klotho	Glyco_hydro_1
		KLRG1_HUMAN	Killer cell lectin-like receptor subfamily G member 1	Lectin_C
		MARCO_HUMAN	Macrophage receptor MARCO	Collagen
		MCP_HUMAN	Membrane cofactor protein	Sushi
		NCTR2_HUMAN	Natural cytotoxicity triggering receptor 2	V-set
		NKG2C_HUMAN	NKG2-C type II integral membrane protein	Lectin_C
		PGFRB_HUMAN	Platelet-derived growth factor receptor beta	Pkinase_Tyr,ig
		RON_HUMAN	Macrophage-stimulating protein receptor	TIG,Sema
		SIG14_HUMAN	Sialic acid-binding Ig-like lectin 14	Ig_2

		SIG15_HUMAN	Sialic acid-binding Ig-like lectin 15	Ig_2
		SIG16_HUMAN	Sialic acid-binding Ig-like lectin 16	I-set,Ig_2
		SIRB1_HUMAN	Signal-regulatory protein beta-1	C1-set
		SLAF7_HUMAN	SLAM family member 7	Ig_3
		TLR3_HUMAN	Toll-like receptor 3	LRR_1
		TLR5_HUMAN	Toll-like receptor 5	LRR_1
		TLR7_HUMAN	Toll-like receptor 7	LRR_1
		TLR8_HUMAN	Toll-like receptor 8	LRR_1
		TLR9_HUMAN	Toll-like receptor 9	LRR_7
		TRIL_HUMAN	TLR4 interactor with leucine rich repeats	LRR_8
		TRML1_HUMAN	Trem-like transcript 1 protein	V-set
		UFO_HUMAN	Tyrosine-protein kinase receptor UFO	Pkinase_Tyr,V-set,fn3,Ig_2
cellular response to chemical stimulus	8.85E-06	1A80_HUMAN	HLA class I histocompatibility antigen, A-80 alpha chain	MHC_I_C
		ACVL1_HUMAN	Serine/threonine-protein kinase receptor R3	Activin_recp
		ADAM8_HUMAN	Disintegrin and metalloproteinase domain-containing protein 8	ADAM_CR,Disintegrin
		AMHR2_HUMAN	Anti-Muellerian hormone type-2 receptor	Pkinase
		ASPH_HUMAN	Aspartyl/asparaginyl beta-hydroxylase	TPR_16
		CCL24_HUMAN	C-C motif chemokine 24	IL8
		CD180_HUMAN	CD180 antigen	LRR_8,LRR_1,LRR_4
		CD19_HUMAN	B-lymphocyte antigen CD19	Ig_2
		CD80_HUMAN	T-lymphocyte activation antigen CD80	V-set
		CO4A2_HUMAN	Collagen alpha-2(IV) chain	Collagen
		COL12_HUMAN	Collectin-12	Collagen,Lectin_C
		CP26A_HUMAN	Cytochrome P450 26A1	p450
		CXCL7_HUMAN	Platelet basic protein	IL8
		DLL4_HUMAN	Delta-like protein 4	hEGF
		DUOX1_HUMAN	Dual oxidase 1	FAD_binding_8
		DUOX2_HUMAN	Dual oxidase 2	An_peroxidase
		EGF_HUMAN	Pro-epidermal growth factor	EGF_CA,Ldl_recept_b
		EGLN_HUMAN	Endoglin	Zona_pellucida
		ENPP1_HUMAN	Ectonucleotide pyrophosphatase/phosphodiesterase family member 1	Somatomedin_B
		EPHA3_HUMAN	Ephrin type-A receptor 3	fn3
		EPOR_HUMAN	Erythropoietin receptor	EpoR_lig-bind,fn3
		ERN1_HUMAN	Serine/threonine-protein kinase/endoribonuclease IRE1	Pkinase
		FCGR1_HUMAN	High affinity immunoglobulin gamma Fc receptor I	ig,Ig_2
		FCGRB_HUMAN	High affinity immunoglobulin gamma Fc receptor IB	Ig_2
		FGF19_HUMAN	Fibroblast growth factor 19	FGF
		FGF6_HUMAN	Fibroblast growth factor 6	FGF
		GHRHR_HUMAN	Growth hormone-releasing hormone receptor	HRM
		GLR_HUMAN	Glucagon receptor	HRM
		GTR8_HUMAN	Solute carrier family 2, facilitated glucose transporter member 8	Sugar_tr
		HAS2_HUMAN	Hyaluronan synthase 2	Chitin_synth_2
		HG2A_HUMAN	HLA class II histocompatibility antigen gamma chain	Thyroglobulin_1
		HGF_HUMAN	Hepatocyte growth factor	Trypsin
		HYOU1_HUMAN	Hypoxia up-regulated protein 1	HSP70
		I12R1_HUMAN	Interleukin-12 receptor subunit beta-1	fn3
		I13R2_HUMAN	Interleukin-13 receptor subunit alpha-2	IL6Ra-bind
		I17RB_HUMAN	Interleukin-17 receptor B	SEFIR
		IFM1_HUMAN	Interferon-induced transmembrane protein 1	CD225
		IFNK_HUMAN	Interferon kappa	Interferon
		IL31R_HUMAN	Interleukin-31 receptor subunit alpha	IL6Ra-bind,fn3
		IL6RA_HUMAN	Interleukin-6 receptor subunit alpha	IL6Ra-bind
		IL6RB_HUMAN	Interleukin-6 receptor subunit beta	fn3
		IL8_HUMAN	Interleukin-8	IL8
		INGR2_HUMAN	Interferon gamma receptor 2	Interfer-bind
		INSR_HUMAN	Insulin receptor	fn3
		ITA1_HUMAN	Integrin alpha-1	Integrin_alpha2,VWA
		ITA9_HUMAN	Integrin alpha-9	FG-GAP
		ITPR2_HUMAN	Inositol 1,4,5-trisphosphate receptor type 2	Ion_trans,Ins145_P3_rec
		JAML1_HUMAN	Junctional adhesion molecule-like	V-set
		KLOTB_HUMAN	Beta-klotho	Glyco_hydro_1
		KLOT_HUMAN	Klotho	Glyco_hydro_1
		LIGO1_HUMAN	Leucine-rich repeat and immunoglobulin-like domain-containing nogo receptor-interacting protein 1	LRR_1
		LRP8_HUMAN	Low-density lipoprotein receptor-related protein 8	Ldl_recept_b
		LTBP3_HUMAN	Latent-transforming growth factor beta-binding protein 3	cEGF,EGF_CA
		NTRK1_HUMAN	High affinity nerve growth factor receptor	Ig_2
		NTRK3_HUMAN	NT-3 growth factor receptor	ig
		OMGP_HUMAN	Oligodendrocyte-myelin glycoprotein	LRRNT,LRR_4
		PCSK5_HUMAN	Proprotein convertase subtilisin/kexin type 5	PLAC
		PDE3A_HUMAN	cGMP-inhibited 3',5'-cyclic phosphodiesterase A	PDEase_I
		PGFRB_HUMAN	Platelet-derived growth factor receptor beta	Pkinase_Tyr,ig
		PLA2R_HUMAN	Secretory phospholipase A2 receptor	Lectin_C
		PLXA4_HUMAN	Plexin-A4	TIG,PSI
		PXDN_HUMAN	Peroxidasin homolog	I-set,VWC
		RON_HUMAN	Macrophage-stimulating protein receptor	TIG,Sema
		RYR1_HUMAN	Ryanodine receptor 1	SPRY
		RYR3_HUMAN	Ryanodine receptor 3	SPRY
		S26A6_HUMAN	Solute carrier family 26 member 6	STAS
		SFRP1_HUMAN	Secreted frizzled-related protein 1	Fz
		SIG15_HUMAN	Sialic acid-binding Ig-like lectin 15	Ig_2
		SLAF8_HUMAN	SLAM family member 8	Ig_3
		TLR9_HUMAN	Toll-like receptor 9	LRR_7
		TNF18_HUMAN	Tumor necrosis factor ligand superfamily member 18	TNF
		TNR1A_HUMAN	Tumor necrosis factor receptor superfamily member 1A	TNFR_c6
		TRPM4_HUMAN	Transient receptor potential cation channel subfamily M member 4	Ion_trans
		UCN2_HUMAN	Urocortin-2	UCN2
		UFO_HUMAN	Tyrosine-protein kinase receptor UFO	Pkinase_Tyr,V-set,fn3,Ig_2
		VASN_HUMAN	Vasorin	LRR_8,LRRNT
		VCAM1_HUMAN	Vascular cell adhesion protein 1	ig
		VGFR2_HUMAN	Vascular endothelial growth factor receptor 2	I-set,V-set
		VMAT2_HUMAN	Synaptic vesicular amine transporter	MFS_1
		WFKN2_HUMAN	WAP, Kazal, immunoglobulin, Kunitz and NTR domain-containing protein 2	Kazal_2
		4F2_HUMAN	4F2 cell-surface antigen heavy chain	Alpha-amylase
		ACVL1_HUMAN	Serine/threonine-protein kinase receptor R3	Activin_recp
		ADAM8_HUMAN	Disintegrin and metalloproteinase domain-containing protein 8	ADAM_CR,Disintegrin
		AT1A4_HUMAN	Sodium/potassium-transporting ATPase subunit alpha-4	E1-E2_ATPase,Cation_ATPase_N
		AT1B3_HUMAN	Sodium/potassium-transporting ATPase subunit beta-3	Na_K-ATPase
		BASI_HUMAN	Basigin	I-set
		BOC_HUMAN	Brother of CDO	Ig_2
		CAC1C_HUMAN	Voltage-dependent L-type calcium channel subunit alpha-1C	Ion_trans
		CADH2_HUMAN	Cadherin-2	Cadherin_C
		CCL24_HUMAN	C-C motif chemokine 24	IL8
		CD244_HUMAN	Natural killer cell receptor 2B4	Ig_2
		CELR1_HUMAN	Cadherin EGF LAG seven-pass G-type receptor 1	Cadherin,Laminin_G_2,EGF
		CELR2_HUMAN	Cadherin EGF LAG seven-pass G-type receptor 2	Cadherin
		CELR3_HUMAN	Cadherin EGF LAG seven-pass G-type receptor 3	DUF3497
		CHL1_HUMAN	Neural cell adhesion molecule L1-like protein	I-set,Ig_2
		CHST4_HUMAN	Carbohydrate sulfotransferase 4	Sulfotransfer_1
		CNTP1_HUMAN	Contactin-associated protein 1	Laminin_G_2
		CO4A2_HUMAN	Collagen alpha-2(IV) chain	Collagen
		CSPG4_HUMAN	Chondroitin sulfate proteoglycan 4	Laminin_G_2
		CTSR1_HUMAN	Cation channel sperm-associated protein 1	Ion_trans
		CTSR3_HUMAN	Cation channel sperm-associated protein 3	Ion_trans
		CTSR4_HUMAN	Cation channel sperm-associated protein 4	Ion_trans
		CXCL7_HUMAN	Platelet basic protein	IL8
		DNER_HUMAN	Delta and Notch-like epidermal growth factor-related receptor	hEGF
		DOPO_HUMAN	Dopamine beta-hydroxylase	DOMON
		EGLN_HUMAN	Endoglin	Zona_pellucida
		ENPP2_HUMAN	Ectonucleotide pyrophosphatase/phosphodiesterase family member 2	Endonuclease_NS
		EPHA3_HUMAN	Ephrin type-A receptor 3	fn3
		EPHA7_HUMAN	Ephrin type-A receptor 7	fn3,SAM_2
		EPHA8_HUMAN	Ephrin type-A receptor 8	Pkinase_Tyr
		EPHB3_HUMAN	Ephrin type-B receptor 3	Ephrin_lbd
		ESAM_HUMAN	Endothelial cell-selective adhesion molecule	V-set,Ig_2
		FAT1_HUMAN	Protocadherin Fat 1	Cadherin,EGF_CA
		FAT2_HUMAN	Protocadherin Fat 2	Cadherin
		FGF19_HUMAN	Fibroblast growth factor 19	FGF
		FUT7_HUMAN	Alpha-(1,3)-fucosyltransferase	Glyco_transf_10
		GP124_HUMAN	G-protein coupled receptor 124	HRM,LRR_8
		GPVI_HUMAN	Platelet glycoprotein VI	Ig_2
		HEXB_HUMAN	Beta-hexosaminidase subunit beta	Glyco_hydro_20b
		HGF_HUMAN	Hepatocyte growth factor	Trypsin

		IGSF8_HUMAN	Immunoglobulin superfamily member 8	V-set
cellular component movement	1.17E-05	IL6RA_HUMAN	Interleukin-6 receptor subunit alpha	IL6Ra-bind
		IL8_HUMAN	Interleukin-8	IL8
		ITA1_HUMAN	Integrin alpha-1	Integrin_alpha2,VWA
		ITA4_HUMAN	Integrin alpha-4	FG-GAP
		ITA6_HUMAN	Integrin alpha-6	Integrin_alpha2
		ITA9_HUMAN	Integrin alpha-9	FG-GAP
		ITAX_HUMAN	Integrin alpha-X	FG-GAP,Integrin_alpha2,VWA
		JAML1_HUMAN	Junctional adhesion molecule-like	V-set
		K0319_HUMAN	Dyslexia-associated protein KIAA0319	REJ
		LYAM2_HUMAN	E-selectin	Sushi
		LYAM3_HUMAN	P-selectin	Sushi
		MIRO2_HUMAN	Mitochondrial Rho GTPase 2	Miro,Ras
		MOT4_HUMAN	Monocarboxylate transporter 4	MFS_1
		NEO1_HUMAN	Neogenin	fn3
		NET1_HUMAN	Netrin-1	Laminin_N
		NFASC_HUMAN	Neurofascin	fn3,Ig_2
		NRCAM_HUMAN	Neuronal cell adhesion molecule	fn3,Ig_3
		NTRK1_HUMAN	High affinity nerve growth factor receptor	Ig_2
		NTRK3_HUMAN	NT-3 growth factor receptor	ig
		PA2GX_HUMAN	Group 10 secretory phospholipase A2	Phospholip_A2_1
		PECA1_HUMAN	Platelet endothelial cell adhesion molecule	Ig_2
		PGFRB_HUMAN	Platelet-derived growth factor receptor beta	Pkinase_Tyr,ig
		PGRC1_HUMAN	Membrane-associated progesterone receptor component 1	Cyt-b5
		PLXA1_HUMAN	Plexin-A1	TIG,PSI
		PLXA2_HUMAN	Plexin-A2	TIG
		PLXA4_HUMAN	Plexin-A4	TIG,PSI
		PLXC1_HUMAN	Plexin-C1	TIG,PSI
		PLXD1_HUMAN	Plexin-D1	TIG,Sema,PSI
		RON_HUMAN	Macrophage-stimulating protein receptor	TIG,Sema
		SCN8A_HUMAN	Sodium channel protein type 8 subunit alpha	Ion_trans
		SEM5A_HUMAN	Semaphorin-5A	PSI,TSP_1
		SEM6D_HUMAN	Semaphorin-6D	PSI
		SG196_HUMAN	Probable inactive protein kinase-like protein SgK196	Pkinase_Tyr
		SLAF5_HUMAN	SLAM family member 5	Ig_3
		SOX1_HUMAN	Transcription factor SOX-1	SOXp
		SPIT2_HUMAN	Kunitz-type protease inhibitor 2	Kunitz_BPTI
		TAOK2_HUMAN	Serine/threonine-protein kinase TAO2	Pkinase
		TRPM4_HUMAN	Transient receptor potential cation channel subfamily M member 4	Ion_trans
		TYRO3_HUMAN	Tyrosine-protein kinase receptor TYRO3	fn3
		UFO_HUMAN	Tyrosine-protein kinase receptor UFO	Pkinase_Tyr,V-set,fn3,Ig_2
		UNC5B_HUMAN	Netrin receptor UNC5B	I-set,TSP_1,ZU5
		VGFR2_HUMAN	Vascular endothelial growth factor receptor 2	I-set,V-set
		ACVL1_HUMAN	Serine/threonine-protein kinase receptor R3	Activin_recp
		ADA10_HUMAN	Disintegrin and metalloproteinase domain-containing protein 10	Pep_M12B_propep
		ADAM8_HUMAN	Disintegrin and metalloproteinase domain-containing protein 8	ADAM_CR,Disintegrin
		BMP10_HUMAN	Bone morphogenetic protein 10	TGFb_propeptide
		CCL24_HUMAN	C-C motif chemokine 24	IL8
		CSF1_HUMAN	Macrophage colony-stimulating factor 1	CSF-1
		DDR2_HUMAN	Discoidin domain-containing receptor 2	Pkinase_Tyr
		DLL4_HUMAN	Delta-like protein 4	hEGF
		DSCAM_HUMAN	Down syndrome cell adhesion molecule	I-set,fn3
		EGLN_HUMAN	Endoglin	Zona_pellucida
		ENPP2_HUMAN	Ectonucleotide pyrophosphatase/phosphodiesterase family member 2	Endonuclease_NS
		GP124_HUMAN	G-protein coupled receptor 124	HRM,LRR_8
		HAS2_HUMAN	Hyaluronan synthase 2	Chitin_synth_2
		HG2A_HUMAN	HLA class II histocompatibility antigen gamma chain	Thyroglobulin_1
		HGF_HUMAN	Hepatocyte growth factor	Trypsin
		IFM1_HUMAN	Interferon-induced transmembrane protein 1	CD225
		IL6RA_HUMAN	Interleukin-6 receptor subunit alpha	IL6Ra-bind
regulation of locomotion	2.36E-05	IL8_HUMAN	Interleukin-8	IL8
		INSR_HUMAN	Insulin receptor	fn3
		JAG1_HUMAN	Protein jagged-1	EGF_CA
		JAG2_HUMAN	Protein jagged-2	hEGF,EGF_CA,EGF
		LRP1_HUMAN	Prolow-density lipoprotein receptor-related protein 1	cEGF,Ldl_recept_b
		LYAM2_HUMAN	E-selectin	Sushi
		LYAM3_HUMAN	P-selectin	Sushi
		NET1_HUMAN	Netrin-1	Laminin_N
		NTRK3_HUMAN	NT-3 growth factor receptor	ig
		PGFRB_HUMAN	Platelet-derived growth factor receptor beta	Pkinase_Tyr,ig
		PLXA2_HUMAN	Plexin-A2	TIG
		PLXA4_HUMAN	Plexin-A4	TIG,PSI
		PLXD1_HUMAN	Plexin-D1	TIG,Sema,PSI
		PTPRJ_HUMAN	Receptor-type tyrosine-protein phosphatase eta	fn3
		SFRP1_HUMAN	Secreted frizzled-related protein 1	Fz
		TIE1_HUMAN	Tyrosine-protein kinase receptor Tie-1	Ig_2
		VGFR2_HUMAN	Vascular endothelial growth factor receptor 2	I-set,V-set
		ZP3_HUMAN	Zona pellucida sperm-binding protein 3	Zona_pellucida
		4F2_HUMAN	4F2 cell-surface antigen heavy chain	Alpha-amylase
		ACVL1_HUMAN	Serine/threonine-protein kinase receptor R3	Activin_recp
		ADAM8_HUMAN	Disintegrin and metalloproteinase domain-containing protein 8	ADAM_CR,Disintegrin
		AT1B3_HUMAN	Sodium/potassium-transporting ATPase subunit beta-3	Na_K-ATPase
		BASI_HUMAN	Basigin	I-set
		CADH2_HUMAN	Cadherin-2	Cadherin_C
		CCL24_HUMAN	C-C motif chemokine 24	IL8
		CD244_HUMAN	Natural killer cell receptor 2B4	Ig_2
		CELR1_HUMAN	Cadherin EGF LAG seven-pass G-type receptor 1	Cadherin,Laminin_G_2,EGF
		CELR2_HUMAN	Cadherin EGF LAG seven-pass G-type receptor 2	Cadherin
		CELR3_HUMAN	Cadherin EGF LAG seven-pass G-type receptor 3	DUF3497
		CHL1_HUMAN	Neural cell adhesion molecule L1-like protein	I-set,Ig_2
		CSPG4_HUMAN	Chondroitin sulfate proteoglycan 4	Laminin_G_2
		CXCL7_HUMAN	Platelet basic protein	IL8
		DNER_HUMAN	Delta and Notch-like epidermal growth factor-related receptor	hEGF
		DOPO_HUMAN	Dopamine beta-hydroxylase	DOMON
		EGLN_HUMAN	Endoglin	Zona_pellucida
		EPHA3_HUMAN	Ephrin type-A receptor 3	fn3
		EPHA8_HUMAN	Ephrin type-A receptor 8	Pkinase_Tyr
		EPHB3_HUMAN	Ephrin type-B receptor 3	Ephrin_lbd
		ESAM_HUMAN	Endothelial cell-selective adhesion molecule	V-set,Ig_2
		FAT1_HUMAN	Protocadherin Fat 1	Cadherin,EGF_CA
		FAT2_HUMAN	Protocadherin Fat 2	Cadherin
		FGF19_HUMAN	Fibroblast growth factor 19	FGF
		FUT7_HUMAN	Alpha-(1,3)-fucosyltransferase	Glyco_transf_10
		GP124_HUMAN	G-protein coupled receptor 124	HRM,LRR_8
		GPVI_HUMAN	Platelet glycoprotein VI	Ig_2
cell migration	2.77E-05	HEXB_HUMAN	Beta-hexosaminidase subunit beta	Glyco_hydro_20b
		HGF_HUMAN	Hepatocyte growth factor	Trypsin
		IL6RA_HUMAN	Interleukin-6 receptor subunit alpha	IL6Ra-bind
		IL8_HUMAN	Interleukin-8	IL8
		ITA1_HUMAN	Integrin alpha-1	Integrin_alpha2,VWA
		ITA4_HUMAN	Integrin alpha-4	FG-GAP
		ITA6_HUMAN	Integrin alpha-6	Integrin_alpha2
		ITA9_HUMAN	Integrin alpha-9	FG-GAP
		ITAX_HUMAN	Integrin alpha-X	FG-GAP,Integrin_alpha2,VWA
		JAML1_HUMAN	Junctional adhesion molecule-like	V-set
		K0319_HUMAN	Dyslexia-associated protein KIAA0319	REJ
		LYAM2_HUMAN	E-selectin	Sushi
		LYAM3_HUMAN	P-selectin	Sushi
		MOT4_HUMAN	Monocarboxylate transporter 4	MFS_1
		NET1_HUMAN	Netrin-1	Laminin_N
		NRCAM_HUMAN	Neuronal cell adhesion molecule	fn3,Ig_3
		NTRK3_HUMAN	NT-3 growth factor receptor	ig
		PECA1_HUMAN	Platelet endothelial cell adhesion molecule	Ig_2
		PGFRB_HUMAN	Platelet-derived growth factor receptor beta	Pkinase_Tyr,ig
		PLXA2_HUMAN	Plexin-A2	TIG
		PLXD1_HUMAN	Plexin-D1	TIG,Sema,PSI
		SG196_HUMAN	Probable inactive protein kinase-like protein SgK196	Pkinase_Tyr
		SLAF5_HUMAN	SLAM family member 5	Ig_3
		SOX1_HUMAN	Transcription factor SOX-1	SOXp
		TAOK2_HUMAN	Serine/threonine-protein kinase TAO2	Pkinase
		TRPM4_HUMAN	Transient receptor potential cation channel subfamily M member 4	Ion_trans
		TYRO3_HUMAN	Tyrosine-protein kinase receptor TYRO3	fn3

		UFO_HUMAN	Tyrosine-protein kinase receptor UFO	Pkinase_Tyr,V-set,fn3,Ig_2
		VGFR2_HUMAN	Vascular endothelial growth factor receptor 2	I-set,V-set
response to oxygen-containing compound	7.30E-05	AMD_HUMAN	Peptidyl-glycine alpha-amidating monooxygenase	NHL
		ASPH_HUMAN	Aspartyl/asparaginyl beta-hydroxylase	TPR_16
		ATP4B_HUMAN	Potassium-transporting ATPase subunit beta	Na_K-ATPase
		BASI_HUMAN	Basigin	I-set
		CAB45_HUMAN	45 kDa calcium-binding protein	EF_hand_5
		CD180_HUMAN	CD180 antigen	LRR_8,LRR_1,LRR_4
		CD27_HUMAN	CD27 antigen	TNFR_c6
		COL12_HUMAN	Collectin-12	Collagen,Lectin_C
		CP26A_HUMAN	Cytochrome P450 26A1	p450
		DOPO_HUMAN	Dopamine beta-hydroxylase	DOMON
		DUOX1_HUMAN	Dual oxidase 1	FAD_binding_8
		DUOX2_HUMAN	Dual oxidase 2	An_peroxidase
		ENPP1_HUMAN	Ectonucleotide pyrophosphatase/phosphodiesterase family member 1	Somatomedin_B
		EPHA3_HUMAN	Ephrin type-A receptor 3	fn3
		FGF19_HUMAN	Fibroblast growth factor 19	FGF
		FGF6_HUMAN	Fibroblast growth factor 6	FGF
		GHRHR_HUMAN	Growth hormone-releasing hormone receptor	HRM
		GLR_HUMAN	Glucagon receptor	HRM
		GPC6A_HUMAN	G-protein coupled receptor family C group 6 member A	NCD3G,ANF_receptor
		GTR8_HUMAN	Solute carrier family 2, facilitated glucose transporter member 8	Sugar_tr
		IL8_HUMAN	Interleukin-8	IL8
		INSR_HUMAN	Insulin receptor	fn3
		ITPR2_HUMAN	Inositol 1,4,5-trisphosphate receptor type 2	Ion_trans,Ins145_P3_rec
		JAG1_HUMAN	Protein jagged-1	EGF_CA
		KLOTB_HUMAN	Beta-klotho	Glyco_hydro_1
		KLOT_HUMAN	Klotho	Glyco_hydro_1
		LYAM2_HUMAN	E-selectin	Sushi
		LYAM3_HUMAN	P-selectin	Sushi
		MRP2_HUMAN	Canalicular multispecific organic anion transporter 1	ABC_membrane
		NMDE2_HUMAN	Glutamate receptor ionotropic, NMDA 2B	ANF_receptor
		NTRK1_HUMAN	High affinity nerve growth factor receptor	Ig_2
		NTRK3_HUMAN	NT-3 growth factor receptor	ig
		PDE3A_HUMAN	cGMP-inhibited 3',5'-cyclic phosphodiesterase A	PDEase_I
		PGFRB_HUMAN	Platelet-derived growth factor receptor beta	Pkinase_Tyr,ig
		PXDN_HUMAN	Peroxidasin homolog	I-set,VWC
		RYR3_HUMAN	Ryanodine receptor 3	SPRY
		S26A6_HUMAN	Solute carrier family 26 member 6	STAS
		SFRP1_HUMAN	Secreted frizzled-related protein 1	Fz
		TIE1_HUMAN	Tyrosine-protein kinase receptor Tie-1	Ig_2
		TLR9_HUMAN	Toll-like receptor 9	LRR_7
		TNR1A_HUMAN	Tumor necrosis factor receptor superfamily member 1A	TNFR_c6
		TRPA1_HUMAN	Transient receptor potential cation channel subfamily A member 1	Ion_trans,Ank
		TRPM2_HUMAN	Transient receptor potential cation channel subfamily M member 2	Ion_trans
		UFO_HUMAN	Tyrosine-protein kinase receptor UFO	Pkinase_Tyr,V-set,fn3,Ig_2
		VCAM1_HUMAN	Vascular cell adhesion protein 1	ig
		VMAT2_HUMAN	Synaptic vesicular amine transporter	MFS_1

Supplementary Table S3. Distributuion of membrane and soluble proteins of non-metazoan and metazoan genomes.

Taxonomy	Species Name	Membrane proteins	Soluble proteins	Total proteins
Non-metazoan	Neosartorya fumigata	1988	7954	9942
Non-metazoan	Saccharomyces cerevisiae	1423	5198	6621
Non-metazoan	Pichia angusta	1017	4321	5338
Non-metazoan	Schizosaccharomyces pombe	952	4153	5105
Non-metazoan	Malassezia globosa	659	3615	4274
Metazoan	Caenorhabditis elegans	8038	17980	26018
Metazoan	Anopheles darlingi	2606	7847	10453
Metazoan	Drosophila melanogaster	5060	15064	20124
Metazoan	Mus musculus	9806	33731	43537
Metazoan	Homo sapiens	11631	56751	68382

Supplementary Table S4. Transmembrane topology of membrane proteins with shared domains in five subcellular localizations

Subcellular localization	SwissProt ID	Protein name	Shared domains	Transmembrane topology
	PLCA_HUMAN	1-acylglycerol-3-phosphate O-acyltransferase 1 (lysophosphatidic acid acyltransferase, alpha)	Acyltransferase	alpha-helical transmembrane proteins
	PLCC_HUMAN	1-acylglycerol-3-phosphate O-acyltransferase 3	Acyltransferase	alpha-helical transmembrane proteins
	Q96B60_HUMAN	5'-nucleotidase, ecto (CD73)	Metallophos	N/A
	5NTD_HUMAN	5'-nucleotidase, ecto (CD73)	5_nucleotid_C,Metallophos	N/A
	5HT3D_HUMAN	5-hydroxytryptamine (serotonin) receptor 3 family member D	Neur_chan_LBD,Neur_chan_memb	alpha-helical transmembrane proteins
	5HT3C_HUMAN	5-hydroxytryptamine (serotonin) receptor 3, family member C	Neur_chan_LBD,Neur_chan_memb	alpha-helical transmembrane proteins
	5HT3E_HUMAN	5-hydroxytryptamine (serotonin) receptor 3, family member E	Neur_chan_LBD,Neur_chan_memb	alpha-helical transmembrane proteins
	Q9UEP2_HUMAN	5-hydroxytryptamine (serotonin) receptor 3A	Neur_chan_memb	N/A
	5HT3A_HUMAN	5-hydroxytryptamine (serotonin) receptor 3A	Neur_chan_LBD,Neur_chan_memb	alpha-helical transmembrane proteins
	5HT3B_HUMAN	5-hydroxytryptamine (serotonin) receptor 3B	Neur_chan_LBD,Neur_chan_memb	alpha-helical transmembrane proteins
	ADA10_HUMAN	ADAM metallopeptidase domain 10	Disintegrin,Pep_M12B_propep,Reprolysin_2	alpha-helical transmembrane proteins
	ADA11_HUMAN	ADAM metallopeptidase domain 11	ADAM_CR,Disintegrin,Pep_M12B_propep,Reprolysin	alpha-helical transmembrane proteins
	ADA12_HUMAN	ADAM metallopeptidase domain 12	ADAM_CR,Disintegrin,Pep_M12B_propep,Reprolysin	alpha-helical transmembrane proteins
	ADA17_HUMAN	ADAM metallopeptidase domain 17	Disintegrin,Pep_M12B_propep	alpha-helical transmembrane proteins
	ADAM2_HUMAN	ADAM metallopeptidase domain 2	ADAM_CR,Disintegrin,Pep_M12B_propep,Reprolysin	alpha-helical transmembrane proteins
	Q6P2G0_HUMAN	ADAM metallopeptidase domain 2	ADAM_CR,Disintegrin,Pep_M12B_propep,Reprolysin	N/A
	ADA23_HUMAN	ADAM metallopeptidase domain 23	ADAM_CR,Disintegrin,Pep_M12B_propep,Reprolysin	alpha-helical transmembrane proteins
	ADA28_HUMAN	ADAM metallopeptidase domain 28	ADAM_CR,Disintegrin,Pep_M12B_propep,Reprolysin	alpha-helical transmembrane proteins
	ADA29_HUMAN	ADAM metallopeptidase domain 29	ADAM_CR,Disintegrin,Pep_M12B_propep,Reprolysin	alpha-helical transmembrane proteins
	ADAM7_HUMAN	ADAM metallopeptidase domain 7	ADAM_CR,Disintegrin,Pep_M12B_propep,Reprolysin	alpha-helical transmembrane proteins
	ADAM8_HUMAN	ADAM metallopeptidase domain 8	ADAM_CR,Disintegrin,Pep_M12B_propep,Reprolysin	alpha-helical transmembrane proteins
	ADAM9_HUMAN	ADAM metallopeptidase domain 9 (meltrin gamma)	ADAM_CR,Disintegrin,Pep_M12B_propep,Reprolysin	alpha-helical transmembrane proteins
	NAR3_HUMAN	ADP-ribosyltransferase 3	ART	N/A
	ABCA1_HUMAN	ATP-binding cassette, sub-family A (ABC1), member 1	ABC_tran	alpha-helical transmembrane proteins
	ABCA3_HUMAN	ATP-binding cassette, sub-family A (ABC1), member 3	ABC_tran	alpha-helical transmembrane proteins
	ABCA4_HUMAN	ATP-binding cassette, sub-family A (ABC1), member 4	ABC_tran	alpha-helical transmembrane proteins
	ABCA7_HUMAN	ATP-binding cassette, sub-family A (ABC1), member 7	ABC_tran	alpha-helical transmembrane proteins
	ABCA8_HUMAN	ATP-binding cassette, sub-family A (ABC1), member 8	ABC_tran	alpha-helical transmembrane proteins
	MDR1_HUMAN	ATP-binding cassette, sub-family B (MDR/TAP), member 1	ABC_tran	alpha-helical transmembrane proteins
	ABCBB_HUMAN	ATP-binding cassette, sub-family B (MDR/TAP), member 11	ABC_tran	alpha-helical transmembrane proteins
	MDR3_HUMAN	ATP-binding cassette, sub-family B (MDR/TAP), member 4	ABC_tran	alpha-helical transmembrane proteins
	ABCB5_HUMAN	ATP-binding cassette, sub-family B (MDR/TAP), member 5; small nuclear ribonucleoprotein 35kDa (U11/U12)	ABC_tran	alpha-helical transmembrane proteins
	MRP1_HUMAN	ATP-binding cassette, sub-family C (CFTR/MRP), member 1	ABC_tran	alpha-helical transmembrane proteins
	MRP7_HUMAN	ATP-binding cassette, sub-family C (CFTR/MRP), member 10	ABC_tran	alpha-helical transmembrane proteins
	MRP2_HUMAN	ATP-binding cassette, sub-family C (CFTR/MRP), member 2	ABC_tran	alpha-helical transmembrane proteins
	MRP3_HUMAN	ATP-binding cassette, sub-family C (CFTR/MRP), member 3	ABC_tran	alpha-helical transmembrane proteins
	MRP4_HUMAN	ATP-binding cassette, sub-family C (CFTR/MRP), member 4	ABC_tran	alpha-helical transmembrane proteins
	MRP5_HUMAN	ATP-binding cassette, sub-family C (CFTR/MRP), member 5	ABC_tran	alpha-helical transmembrane proteins
	ABCC8_HUMAN	ATP-binding cassette, sub-family C (CFTR/MRP), member 8	ABC_tran	alpha-helical transmembrane proteins
	ABCC9_HUMAN	ATP-binding cassette, sub-family C (CFTR/MRP), member 9	ABC_tran	alpha-helical transmembrane proteins
	MRP6_HUMAN	ATP-binding cassette, sub-family C, member 6 pseudogene 2; ATP-binding cassette, sub-family C (CFTR/MRP), member 6	ABC_tran	alpha-helical transmembrane proteins
	ABCG1_HUMAN	ATP-binding cassette, sub-family G (WHITE), member 1	ABC_tran	alpha-helical transmembrane proteins
	ABCG2_HUMAN	ATP-binding cassette, sub-family G (WHITE), member 2	ABC_tran	alpha-helical transmembrane proteins
	ABCG5_HUMAN	ATP-binding cassette, sub-family G (WHITE), member 5	ABC_tran	alpha-helical transmembrane proteins
	ABCG8_HUMAN	ATP-binding cassette, sub-family G (WHITE), member 8	ABC_tran	alpha-helical transmembrane proteins
	AT1B4_HUMAN	ATPase, (Na+)/K+ transporting, beta 4 polypeptide	Na_K-ATPase	alpha-helical transmembrane proteins
	AT2A2_HUMAN	ATPase, Ca++ transporting, cardiac muscle, slow twitch 2	E1-E2_ATPase,Cation_ATPase_N	alpha-helical transmembrane proteins
	AT2B1_HUMAN	ATPase, Ca++ transporting, plasma membrane 1	E1-E2_ATPase,Cation_ATPase_N,ATP_Ca_trans_C	alpha-helical transmembrane proteins
	AT2B2_HUMAN	ATPase, Ca++ transporting, plasma membrane 2	ATP_Ca_trans_C,E1-E2_ATPase,Cation_ATPase_N	alpha-helical transmembrane proteins
	AT2B3_HUMAN	ATPase, Ca++ transporting, plasma membrane 3	ATP_Ca_trans_C,Cation_ATPase_N,E1-E2_ATPase	alpha-helical transmembrane proteins
	AT2B4_HUMAN	ATPase, Ca++ transporting, plasma membrane 4	Cation_ATPase_N,ATP_Ca_trans_C,E1-E2_ATPase	alpha-helical transmembrane proteins
	ATP7A_HUMAN	ATPase, Cu++ transporting, alpha polypeptide	HMA,E1-E2_ATPase	alpha-helical transmembrane proteins
	ATP7B_HUMAN	ATPase, Cu++ transporting, beta polypeptide	HMA,E1-E2_ATPase	alpha-helical transmembrane proteins
	VPP4_HUMAN	ATPase, H+ transporting, lysosomal V0 subunit a4	V_ATPase_I	alpha-helical transmembrane proteins
	ATP4A_HUMAN	ATPase, H+/K+ exchanging, alpha polypeptide	Cation_ATPase_N,E1-E2_ATPase	alpha-helical transmembrane proteins
	AT12A_HUMAN	ATPase, H+/K+ transporting, nongastric, alpha polypeptide	E1-E2_ATPase,Cation_ATPase_N	alpha-helical transmembrane proteins
	Q5TC01_HUMAN	ATPase, Na+/K+ transporting, alpha 1 polypeptide	Cation_ATPase_N	N/A
	AT1A1_HUMAN	ATPase, Na+/K+ transporting, alpha 1 polypeptide	Cation_ATPase_N,E1-E2_ATPase	alpha-helical transmembrane proteins
	Q5TC02_HUMAN	ATPase, Na+/K+ transporting, alpha 1 polypeptide	Cation_ATPase_N	N/A
	AT1A2_HUMAN	ATPase, Na+/K+ transporting, alpha 2 (+) polypeptide	E1-E2_ATPase,Cation_ATPase_N	alpha-helical transmembrane proteins
	AT1A3_HUMAN	ATPase, Na+/K+ transporting, alpha 3 polypeptide	Cation_ATPase_N,E1-E2_ATPase	alpha-helical transmembrane proteins
	AT1A4_HUMAN	ATPase, Na+/K+ transporting, alpha 4 polypeptide	E1-E2_ATPase,Cation_ATPase_N	alpha-helical transmembrane proteins
	AT1B1_HUMAN	ATPase, Na+/K+ transporting, beta 1 polypeptide	Na_K-ATPase	alpha-helical transmembrane proteins
	AT1B2_HUMAN	ATPase, Na+/K+ transporting, beta 2 polypeptide	Na_K-ATPase	alpha-helical transmembrane proteins
	AT1B3_HUMAN	ATPase, Na+/K+ transporting, beta 3 polypeptide	Na_K-ATPase	alpha-helical transmembrane proteins
	AT8B1_HUMAN	ATPase, class I, type 8B, member 1	HAD,E1-E2_ATPase	alpha-helical transmembrane proteins
	UFO_HUMAN	AXL receptor tyrosine kinase	Pkinase_Tyr,fn3,V-set,Ig_2	alpha-helical transmembrane proteins
	BTLA_HUMAN	B and T lymphocyte associated	Ig_2	alpha-helical transmembrane proteins
	BAP31_HUMAN	B-cell receptor-associated protein 31	Bap31	alpha-helical transmembrane proteins
	B2CL1_HUMAN	BCL2-like 1	Bcl-2,BH4	alpha-helical transmembrane proteins
	BOC_HUMAN	Boc homolog (mouse)	I-set,fn3,Ig_2	alpha-helical transmembrane proteins
	CLC1A_HUMAN	C-type lectin domain family 1, member A	Lectin_C	alpha-helical transmembrane proteins
	CLC1B_HUMAN	C-type lectin domain family 1, member B	Lectin_C	alpha-helical transmembrane proteins
	CLC10_HUMAN	C-type lectin domain family 10, member A	Lectin_N,Lectin_C	alpha-helical transmembrane proteins
	CL12B_HUMAN	C-type lectin domain family 12, member A; C-type lectin domain family 12, member B	Lectin_C	alpha-helical transmembrane proteins
	CL12A_HUMAN	C-type lectin domain family 12, member A; C-type lectin domain family 12, member B	Lectin_C	alpha-helical transmembrane proteins
	CLC2A_HUMAN	C-type lectin domain family 2, member A	Lectin_C	alpha-helical transmembrane proteins
	CLC2B_HUMAN	C-type lectin domain family 2, member B	Lectin_C	alpha-helical transmembrane proteins
	CLC2D_HUMAN	C-type lectin domain family 2, member D	Lectin_C	alpha-helical transmembrane proteins
	CLC4A_HUMAN	C-type lectin domain family 4, member A	Lectin_C	alpha-helical transmembrane proteins
	CLC4M_HUMAN	C-type lectin domain family 4, member M	Lectin_C	alpha-helical transmembrane proteins
	Q14DL9_HUMAN	C-type lectin domain family 5, member A	Lectin_C	N/A
	CLC5A_HUMAN	C-type lectin domain family 5, member A	Lectin_C	alpha-helical transmembrane proteins
	CLC7A_HUMAN	C-type lectin domain family 7, member A	Lectin_C	alpha-helical transmembrane proteins
	C163A_HUMAN	CD163 molecule	SRCR	alpha-helical transmembrane proteins
	C163B_HUMAN	CD163 molecule-like 1	SRCR	alpha-helical transmembrane proteins
	MUC24_HUMAN	CD164 molecule, sialomucin	MGC-24	alpha-helical transmembrane proteins
	CD180_HUMAN	CD180 molecule	LRR_8	alpha-helical transmembrane proteins
	CD1A_HUMAN	CD1a molecule	C1-set,MHC_I	alpha-helical transmembrane proteins
	CD1B_HUMAN	CD1b molecule	C1-set	alpha-helical transmembrane proteins
	CD1C_HUMAN	CD1c molecule	C1-set	alpha-helical transmembrane proteins
	CD1D_HUMAN	CD1d molecule	C1-set	alpha-helical transmembrane proteins
	CD1E_HUMAN	CD1e molecule	C1-set	alpha-helical transmembrane proteins
	CD2_HUMAN	CD2 molecule	WSC,ABC_tran,ABC_membrane_2,zf-MYND,Thyroglobulin_1,HECT,C2-set,K_tetra,C1_1,Glyco_transf_54,TRAM_LAG1_CLN8,adh_short,PH,Pkinase,V-set	alpha-helical transmembrane proteins
	OX2G_HUMAN	CD200 molecule	ig	alpha-helical transmembrane proteins
	MO2R1_HUMAN	CD200 receptor 1	C2-set_2	alpha-helical transmembrane proteins
	CD22_HUMAN	CD22 molecule	C2-set_2,ig,Ig_2	alpha-helical transmembrane proteins
	CD226_HUMAN	CD226 molecule	V-set	alpha-helical transmembrane proteins
	CD244_HUMAN	CD244 molecule, natural killer cell receptor 2B4	Ig_2	alpha-helical transmembrane proteins
	CD3Z_HUMAN	CD247 molecule	ITAM	alpha-helical transmembrane proteins
	CD27_HUMAN	CD27 molecule	TNFR_c6	alpha-helical transmembrane proteins
	PD1L1_HUMAN	CD274 molecule	C2-set_2,V-set	alpha-helical transmembrane proteins
	CD276_HUMAN	CD276 molecule	C2-set_2,V-set	alpha-helical transmembrane proteins
	CD28_HUMAN	CD28 molecule	V-set	alpha-helical transmembrane proteins
	CLM7_HUMAN	CD300 molecule-like family member b	V-set	alpha-helical transmembrane proteins
	CLM4_HUMAN	CD300 molecule-like family member d	V-set	alpha-helical transmembrane proteins
	CLM1_HUMAN	CD300 molecule-like family member f	V-set	alpha-helical transmembrane proteins
	CLM9_HUMAN	CD300 molecule-like family member g	V-set	alpha-helical transmembrane proteins

CLM8_HUMAN	CD300a molecule	V-set	alpha-helical transmembrane proteins
CLM6_HUMAN	CD300c molecule	V-set	alpha-helical transmembrane proteins
CLM2_HUMAN	CD300e molecule	V-set	alpha-helical transmembrane proteins
LY75_HUMAN	CD302 molecule; lymphocyte antigen 75	fn2,Lectin_C	alpha-helical transmembrane proteins
CD302_HUMAN	CD302 molecule; lymphocyte antigen 75	Lectin_C	alpha-helical transmembrane proteins
CD33_HUMAN	CD33 molecule	C2,V-set,ig	alpha-helical transmembrane proteins
CD38_HUMAN	CD38 molecule	Rib_hydrolayse	alpha-helical transmembrane proteins
CD3E_HUMAN	CD3e molecule, epsilon (CD3-TCR complex)	ITAM	alpha-helical transmembrane proteins
CD3G_HUMAN	CD3g molecule, gamma (CD3-TCR complex)	ITAM	alpha-helical transmembrane proteins
CD4_HUMAN	CD4 molecule	C2-set,RabGAP-TBC,ABC_tran,HECT,V-set,ABC_membrane_2,C2,PH	alpha-helical transmembrane proteins
CD40L_HUMAN	CD40 ligand	TNF	alpha-helical transmembrane proteins
Q3L8U2_HUMAN	CD40 ligand	TNF	N/A
TNR5_HUMAN	CD40 molecule, TNF receptor superfamily member 5	TNFR_c6	alpha-helical transmembrane proteins
CD44_HUMAN	CD44 molecule (Indian blood group)	Xlink	alpha-helical transmembrane proteins
MCP_HUMAN	CD46 molecule, complement regulatory protein	Sushi	alpha-helical transmembrane proteins
CD48_HUMAN	CD48 molecule	V-set,Ig_2	N/A
CD5_HUMAN	CD5 molecule	C1_1,DAGK_cat,RabGAP-TBC,SRCR,FA_desaturase,C2,EF_hand_5,DAGK_acc	alpha-helical transmembrane proteins
CD6_HUMAN	CD6 molecule	SRCR,EF_hand_6,EF_hand_5,PAN_1,Pkinase	alpha-helical transmembrane proteins
CD68_HUMAN	CD68 molecule	Lamp	alpha-helical transmembrane proteins
CD69_HUMAN	CD69 molecule	Lectin_C	alpha-helical transmembrane proteins
CD7_HUMAN	CD7 molecule	RabGAP-TBC,Oxysterol_BP,Glyco_hydro_38C,V-set,Alpha-mann_mid,Glyco_hydro_38	alpha-helical transmembrane proteins
CD70_HUMAN	CD70 molecule	TNF	alpha-helical transmembrane proteins
CD72_HUMAN	CD72 molecule	Lectin_C	alpha-helical transmembrane proteins
HG2A_HUMAN	CD74 molecule, major histocompatibility complex, class II invariant chain	Thyroglobulin_1	alpha-helical transmembrane proteins
CD79A_HUMAN	CD79a molecule, immunoglobulin-associated alpha	V-set,ITAM	alpha-helical transmembrane proteins
CD79B_HUMAN	CD79b molecule, immunoglobulin-associated beta	V-set	alpha-helical transmembrane proteins
CD80_HUMAN	CD80 molecule	C2-set_2,V-set,DUF4174	alpha-helical transmembrane proteins
CD83_HUMAN	CD83 molecule	V-set	alpha-helical transmembrane proteins
SLAF5_HUMAN	CD84 molecule	Ig_3	alpha-helical transmembrane proteins
CD86_HUMAN	CD86 molecule	V-set	alpha-helical transmembrane proteins
CD8A_HUMAN	CD8a molecule	V-set	alpha-helical transmembrane proteins
CD8B_HUMAN	CD8b molecule	V-set	alpha-helical transmembrane proteins
C1QR1_HUMAN	CD93 molecule	cEGF,Lectin_C,EGF_CA	alpha-helical transmembrane proteins
TACT_HUMAN	CD96 molecule	Ig_2	alpha-helical transmembrane proteins
CD97_HUMAN	CD97 molecule	EGF_CA	alpha-helical transmembrane proteins
CD99_HUMAN	CD99 molecule	CD99L2	alpha-helical transmembrane proteins
C99L2_HUMAN	CD99 molecule-like 2	CD99L2	alpha-helical transmembrane proteins
CDIPT_HUMAN	CDP-diacylglycerol--inositol 3-phosphatidyltransferase (phosphatidylinositol synthase)	CDP-OH_P_transf	alpha-helical transmembrane proteins
CRFM7_HUMAN	CHRNA7 (cholinergic receptor, nicotinic, alpha 7, exons 5-10) and FAM7A (family with sequence similarity 7A, exons A-E) fusion; cholinergic receptor, nicotinic, alpha 7	Neur_chan_LBD,Neur_chan_memb	alpha-helical transmembrane proteins
ACHA7_HUMAN	CHRNA7 (cholinergic receptor, nicotinic, alpha 7, exons 5-10) and FAM7A (family with sequence similarity 7A, exons A-E) fusion; cholinergic receptor, nicotinic, alpha 7	Neur_chan_LBD,Neur_chan_memb	alpha-helical transmembrane proteins
CSMD2_HUMAN	CUB and Sushi multiple domains 2	Sushi,CUB	alpha-helical transmembrane proteins
CSMD3_HUMAN	CUB and Sushi multiple domains 3	Sushi,CUB	alpha-helical transmembrane proteins
DJC25_HUMAN	DnaJ (Hsp40) homolog, subfamily C , member 25; guanine nucleotide binding protein (G protein), gamma 10; DNAJC25-GNG10 readthrough transcript	DnaJ	alpha-helical transmembrane proteins
DJC15_HUMAN	DnaJ (Hsp40) homolog, subfamily C, member 15	DnaJ	alpha-helical transmembrane proteins
DNJC5_HUMAN	DnaJ (Hsp40) homolog, subfamily C, member 5	DnaJ	N/A
DSCAM_HUMAN	Down syndrome cell adhesion molecule	I-set,fn3	alpha-helical transmembrane proteins
Q8WY19_HUMAN	Down syndrome cell adhesion molecule	I-set,fn3	N/A
ELTD1_HUMAN	EGF, latrophilin and seven transmembrane domain containing 1	EGF_CA,DUF3497	alpha-helical transmembrane proteins
EGFLA_HUMAN	EGF-like, fibronectin type III and laminin G domains	Laminin_G_2,fn3,EGF,Laminin_G_1	N/A
EPHA1_HUMAN	EPH receptor A1	Pkinase_Tyr,Ephrin_lbd,fn3,SAM_1	alpha-helical transmembrane proteins
EPHA2_HUMAN	EPH receptor A2	Pkinase_Tyr,Ephrin_lbd,fn3,SAM_1	alpha-helical transmembrane proteins
EPHA3_HUMAN	EPH receptor A3	Pkinase_Tyr,Ephrin_lbd,fn3,GCC2_GCC3,SAM_2	alpha-helical transmembrane proteins
EPHA4_HUMAN	EPH receptor A4	Pkinase_Tyr,Ephrin_lbd,fn3,SAM_2	alpha-helical transmembrane proteins
EPHA6_HUMAN	EPH receptor A6	Pkinase_Tyr,SAM_1,fn3,Ephrin_lbd	alpha-helical transmembrane proteins
EPHA7_HUMAN	EPH receptor A7	Pkinase_Tyr,Ephrin_lbd,fn3,SAM_1,GCC2_GCC3	alpha-helical transmembrane proteins
EPHA8_HUMAN	EPH receptor A8	Pkinase_Tyr,Ephrin_lbd,fn3,SAM_1	alpha-helical transmembrane proteins
EPHB1_HUMAN	EPH receptor B1	Pkinase_Tyr,Ephrin_lbd,fn3,SAM_1	alpha-helical transmembrane proteins
EPHB2_HUMAN	EPH receptor B2	Pkinase_Tyr,Ephrin_lbd,fn3,SAM_1,GCC2_GCC3	alpha-helical transmembrane proteins
EPHB3_HUMAN	EPH receptor B3	Pkinase_Tyr,Ephrin_lbd,fn3,SAM_1,GCC2_GCC3	alpha-helical transmembrane proteins
Q96L35_HUMAN	EPH receptor B4	Pkinase_Tyr,SAM_1,fn3,GCC2_GCC3,Ephrin_lbd	N/A
EPHB4_HUMAN	EPH receptor B4	Pkinase_Tyr,Ephrin_lbd,fn3,SAM_1,GCC2_GCC3	alpha-helical transmembrane proteins
EPHB6_HUMAN	EPH receptor B6	Pkinase_Tyr,Ephrin_lbd,fn3,GCC2_GCC3,SAM_2	alpha-helical transmembrane proteins
JAM1_HUMAN	F11 receptor	V-set,Ig_2	alpha-helical transmembrane proteins
FAT1_HUMAN	FAT tumor suppressor homolog 1 (Drosophila)	Cadherin,EGF_CA,Laminin_G_2,EGF	alpha-helical transmembrane proteins
FAT2_HUMAN	FAT tumor suppressor homolog 2 (Drosophila)	Cadherin,Laminin_G_2	alpha-helical transmembrane proteins
FRMD5_HUMAN	FERM domain containing 5	FERM_C,FA,FERM_N,FERM_M	alpha-helical transmembrane proteins
FKBP8_HUMAN	FK506 binding protein 8, 38kDa	TPR_16,FKBP_C	alpha-helical transmembrane proteins
FREM2_HUMAN	FRAS1 related extracellular matrix protein 2	Calx-beta	alpha-helical transmembrane proteins
FAIM3_HUMAN	Fas apoptotic inhibitory molecule 3	ig	alpha-helical transmembrane proteins
TNFL6_HUMAN	Fas ligand (TNF superfamily, member 6)	TNF	alpha-helical transmembrane proteins
FCERA_HUMAN	Fc fragment of IgE, high affinity I, receptor for; alpha polypeptide	Ig_2	alpha-helical transmembrane proteins
FCERG_HUMAN	Fc fragment of IgE, high affinity I, receptor for; gamma polypeptide	ITAM	alpha-helical transmembrane proteins
FCER2_HUMAN	Fc fragment of IgE, low affinity II, receptor for (CD23)	Lectin_C	alpha-helical transmembrane proteins
FCGRB_HUMAN	Fc fragment of IgG, high affinity Ib, receptor (CD64)	Ig_2	alpha-helical transmembrane proteins
FCGR1_HUMAN	Fc fragment of IgG, high affinity Ic, receptor (CD64); Fc fragment of IgG, high affinity Ia, receptor (CD64)	Ig_2	alpha-helical transmembrane proteins
FCG3A_HUMAN	Fc fragment of IgG, low affinity IIIa, receptor (CD16a)	Ig_2	alpha-helical transmembrane proteins
FCG3B_HUMAN	Fc fragment of IgG, low affinity IIIb, receptor (CD16b)	Ig_2	N/A
FCG2A_HUMAN	Fc fragment of IgG, low affinity IIa, receptor (CD32)	Ig_2	alpha-helical transmembrane proteins
FCG2C_HUMAN	Fc fragment of IgG, low affinity IIb, receptor (CD32); Fc fragment of IgG, low affinity IIc, receptor for (CD32)	Ig_2	alpha-helical transmembrane proteins
FCG2B_HUMAN	Fc fragment of IgG, low affinity IIb, receptor (CD32); Fc fragment of IgG, low affinity IIc, receptor for (CD32)	Ig_2	alpha-helical transmembrane proteins
FCGRN_HUMAN	Fc fragment of IgG, receptor, transporter, alpha	C1-set,MHC_I	alpha-helical transmembrane proteins
FCAMR_HUMAN	Fc receptor, IgA, IgM, high affinity	V-set	alpha-helical transmembrane proteins
FCRL1_HUMAN	Fc receptor-like 1	Ig_3,Ig_2	alpha-helical transmembrane proteins
FCRL2_HUMAN	Fc receptor-like 2	Ig_3,Ig_2	alpha-helical transmembrane proteins
FCRL3_HUMAN	Fc receptor-like 3	Ig_3,Ig_2	alpha-helical transmembrane proteins
FCRL4_HUMAN	Fc receptor-like 4	ig,Ig_2	alpha-helical transmembrane proteins
FCRL5_HUMAN	Fc receptor-like 5	Ig_3,Ig_2	alpha-helical transmembrane proteins
FRAS1_HUMAN	Fraser syndrome 1	Calx-beta,VWC	alpha-helical transmembrane proteins
GP110_HUMAN	G protein-coupled receptor 110	SEA	alpha-helical transmembrane proteins
GP112_HUMAN	G protein-coupled receptor 112	Pentaxin	alpha-helical transmembrane proteins
GP113_HUMAN	G protein-coupled receptor 113	HRM,DUF3497	alpha-helical transmembrane proteins
GP116_HUMAN	G protein-coupled receptor 116	I-set,SEA	alpha-helical transmembrane proteins
GP124_HUMAN	G protein-coupled receptor 124	HRM,LRR_8	alpha-helical transmembrane proteins
GP125_HUMAN	G protein-coupled receptor 125	LRR_8,I-set	alpha-helical transmembrane proteins
GP126_HUMAN	G protein-coupled receptor 126	Pentaxin,CUB	alpha-helical transmembrane proteins
GP133_HUMAN	G protein-coupled receptor 133	Laminin_G_3	alpha-helical transmembrane proteins
GP144_HUMAN	G protein-coupled receptor 144	Pentaxin	alpha-helical transmembrane proteins
GPR98_HUMAN	G protein-coupled receptor 98	Calx-beta,Laminin_G_3,EPTP	alpha-helical transmembrane proteins
GPC6A_HUMAN	G protein-coupled receptor, family C, group 6, member A	NCD3G,ANF_receptor	alpha-helical transmembrane proteins
GFRAL_HUMAN	GDNF family receptor alpha like	GDNF	alpha-helical transmembrane proteins
GRM1A_HUMAN	GRAM domain containing 1A	GRAM	alpha-helical transmembrane proteins
IHH_HUMAN	Indian hedgehog homolog (Drosophila)	HH_signal,Hint	N/A
K0319_HUMAN	KIAA0319	REJ	alpha-helical transmembrane proteins
NAGT1_HUMAN	KIAA1919	MFS_1	alpha-helical transmembrane proteins
SCF_HUMAN	KIT ligand	SCF	alpha-helical transmembrane proteins
KELL_HUMAN	Kell blood group, metallo-endopeptidase	Peptidase_M13_N,Peptidase_M13	alpha-helical transmembrane proteins
L1CAM_HUMAN	L1 cell adhesion molecule	I-set,fn3,Bravo_FIGEY,Ig_2	alpha-helical transmembrane proteins
MDGA2_HUMAN	MAM domain containing glycosylphosphatidylinositol anchor 2	I-set,MAM,Ig_2	N/A

MALD2_HUMAN	MARVEL domain containing 2	Occludin_ELL	alpha-helical transmembrane proteins
Q5SS58_HUMAN	MHC class I polypeptide-related sequence A	C1-set	N/A
MICA_HUMAN	MHC class I polypeptide-related sequence A	C1-set,MHC_I	alpha-helical transmembrane proteins
Q96QC4_HUMAN	MHC class I polypeptide-related sequence A	C1-set,MHC_I	N/A
NALDL_HUMAN	N-acetylated alpha-linked acidic dipeptidase-like 1	TFR_dimer,Peptidase_M28,PA	alpha-helical transmembrane proteins
ASAH2_HUMAN	N-acylsphingosine amidohydrolase (non-lysosomal ceramidase) 2B	Ceramidase_alk	alpha-helical transmembrane proteins
ASAH2_HUMAN	N-acylsphingosine amidohydrolase (non-lysosomal ceramidase) 2C; N-acylsphingosine amidohydrolase (non-lysosomal ceramidase) 2	Ceramidase_alk	alpha-helical transmembrane proteins
NOX1_HUMAN	NADPH oxidase 1	RRM_1,NAD_binding_6,FAD_binding_8	alpha-helical transmembrane proteins
NOX3_HUMAN	NADPH oxidase 3	NAD_binding_6,FAD_binding_8	alpha-helical transmembrane proteins
NOX4_HUMAN	NADPH oxidase 4	NAD_binding_6,FAD_binding_8	alpha-helical transmembrane proteins
NKAI1_HUMAN	Na+/K+ transporting ATPase interacting 1	NKAIN	alpha-helical transmembrane proteins
NKAI2_HUMAN	Na+/K+ transporting ATPase interacting 2	NKAIN	alpha-helical transmembrane proteins
NKAI3_HUMAN	Na+/K+ transporting ATPase interacting 3	NKAIN	alpha-helical transmembrane proteins
NKAI4_HUMAN	Na+/K+ transporting ATPase interacting 4	NKAIN	alpha-helical transmembrane proteins
NOTC2_HUMAN	Notch homolog 2 (Drosophila)	EGF_CA,Ank_2,NOD,DUF3454,NODP,EGF	alpha-helical transmembrane proteins
CRCM1_HUMAN	ORAI calcium release-activated calcium modulator 1	Orai-1	alpha-helical transmembrane proteins
PTK7_HUMAN	PTK7 protein tyrosine kinase 7	Pkinase_Tyr,I-set,Ig_2	alpha-helical transmembrane proteins
Q86X91_HUMAN	PTK7 protein tyrosine kinase 7	I-set,Ig_2	N/A
RYK_HUMAN	RYK receptor-like tyrosine kinase	Pkinase_Tyr,WIF	alpha-helical transmembrane proteins
PRAF1_HUMAN	Rab acceptor 1 (prenylated)	PRA1	alpha-helical transmembrane proteins
SLAF6_HUMAN	SLAM family member 6	V-set	alpha-helical transmembrane proteins
SIAT9_HUMAN	ST3 beta-galactoside alpha-2,3-sialyltransferase 5	Glyco_transf_29	alpha-helical transmembrane proteins
SIA7F_HUMAN	ST6 (alpha-N-acetyl-neuraminyl-2,3-beta-galactosyl-1,3)-N-acetylgalactosaminide alpha-2,6-sialyltransferase 6	Glyco_transf_29	alpha-helical transmembrane proteins
STEA3_HUMAN	STEAP family member 3	F420_oxidored	alpha-helical transmembrane proteins
STEA4_HUMAN	STEAP family member 4	F420_oxidored	alpha-helical transmembrane proteins
SVOP_HUMAN	SV2 related protein homolog (rat)	MFS_1	alpha-helical transmembrane proteins
TIGIT_HUMAN	T cell immunoreceptor with Ig and ITIM domains	V-set	alpha-helical transmembrane proteins
VPP3_HUMAN	T-cell, immune regulator 1, ATPase, H+ transporting, lysosomal V0 subunit A3	V_ATPase_I	alpha-helical transmembrane proteins
TPSNR_HUMAN	TAP binding protein-like	C1-set,V-set	alpha-helical transmembrane proteins
TIE2_HUMAN	TEK tyrosine kinase, endothelial	Pkinase_Tyr,fn3	alpha-helical transmembrane proteins
TM2D1_HUMAN	TM2 domain containing 1	TM2	alpha-helical transmembrane proteins
TNF13_HUMAN	TNFSF12-TNFSF13 readthrough transcript; tumor necrosis factor (ligand) superfamily, member 12; tumor necrosis factor (ligand) superfamily, member 13	TNF	N/A
TNF12_HUMAN	TNFSF12-TNFSF13 readthrough transcript; tumor necrosis factor (ligand) superfamily, member 12; tumor necrosis factor (ligand) superfamily, member 13	TNF	alpha-helical transmembrane proteins
TYRO3_HUMAN	TYRO3 protein tyrosine kinase	Pkinase_Tyr,I-set,fn3	alpha-helical transmembrane proteins
UD13_HUMAN	UDP glucuronosyltransferase 1 family, polypeptide A3; UDP glucuronosyltransferase 1 family, polypeptide A5; UDP glucuronosyltransferase 1 family, polypeptide A4; UDP glucuronosyltransferase 1 family, polypeptide A7; UDP glucuronosyltransferase 1 family, polypeptide A6; UDP glucuronosyltransferase 1 family, polypeptide A10; UDP glucuronosyltransferase 1 family, polypeptide A9; UDP glucuronosyltransferase 1 family, polypeptide A8; UDP glucuronosyltransferase 1 family, polypeptide A1	UDPGT	alpha-helical transmembrane proteins
UD16_HUMAN	UDP glucuronosyltransferase 1 family, polypeptide A3; UDP glucuronosyltransferase 1 family, polypeptide A5; UDP glucuronosyltransferase 1 family, polypeptide A4; UDP glucuronosyltransferase 1 family, polypeptide A7; UDP glucuronosyltransferase 1 family, polypeptide A6; UDP glucuronosyltransferase 1 family, polypeptide A10; UDP glucuronosyltransferase 1 family, polypeptide A9; UDP glucuronosyltransferase 1 family, polypeptide A8; UDP glucuronosyltransferase 1 family, polypeptide A1	UDPGT	alpha-helical transmembrane proteins
UD110_HUMAN	UDP glucuronosyltransferase 1 family, polypeptide A3; UDP glucuronosyltransferase 1 family, polypeptide A5; UDP glucuronosyltransferase 1 family, polypeptide A4; UDP glucuronosyltransferase 1 family, polypeptide A7; UDP glucuronosyltransferase 1 family, polypeptide A6; UDP glucuronosyltransferase 1 family, polypeptide A10; UDP glucuronosyltransferase 1 family, polypeptide A9; UDP glucuronosyltransferase 1 family, polypeptide A8; UDP glucuronosyltransferase 1 family, polypeptide A1	UDPGT	alpha-helical transmembrane proteins
UD18_HUMAN	UDP glucuronosyltransferase 1 family, polypeptide A3; UDP glucuronosyltransferase 1 family, polypeptide A5; UDP glucuronosyltransferase 1 family, polypeptide A4; UDP glucuronosyltransferase 1 family, polypeptide A7; UDP glucuronosyltransferase 1 family, polypeptide A6; UDP glucuronosyltransferase 1 family, polypeptide A10; UDP glucuronosyltransferase 1 family, polypeptide A9; UDP glucuronosyltransferase 1 family, polypeptide A8; UDP glucuronosyltransferase 1 family, polypeptide A1	UDPGT	alpha-helical transmembrane proteins
UD19_HUMAN	UDP glucuronosyltransferase 1 family, polypeptide A3; UDP glucuronosyltransferase 1 family, polypeptide A5; UDP glucuronosyltransferase 1 family, polypeptide A4; UDP glucuronosyltransferase 1 family, polypeptide A7; UDP glucuronosyltransferase 1 family, polypeptide A6; UDP glucuronosyltransferase 1 family, polypeptide A10; UDP glucuronosyltransferase 1 family, polypeptide A9; UDP glucuronosyltransferase 1 family, polypeptide A8; UDP glucuronosyltransferase 1 family, polypeptide A1	UDPGT	alpha-helical transmembrane proteins
UD14_HUMAN	UDP glucuronosyltransferase 1 family, polypeptide A3; UDP glucuronosyltransferase 1 family, polypeptide A5; UDP glucuronosyltransferase 1 family, polypeptide A4; UDP glucuronosyltransferase 1 family, polypeptide A7; UDP glucuronosyltransferase 1 family, polypeptide A6; UDP glucuronosyltransferase 1 family, polypeptide A10; UDP glucuronosyltransferase 1 family, polypeptide A9; UDP glucuronosyltransferase 1 family, polypeptide A8; UDP glucuronosyltransferase 1 family, polypeptide A1	UDPGT	alpha-helical transmembrane proteins
UD15_HUMAN	UDP glucuronosyltransferase 1 family, polypeptide A3; UDP glucuronosyltransferase 1 family, polypeptide A5; UDP glucuronosyltransferase 1 family, polypeptide A4; UDP glucuronosyltransferase 1 family, polypeptide A7; UDP glucuronosyltransferase 1 family, polypeptide A6; UDP glucuronosyltransferase 1 family, polypeptide A10; UDP glucuronosyltransferase 1 family, polypeptide A9; UDP glucuronosyltransferase 1 family, polypeptide A8; UDP glucuronosyltransferase 1 family, polypeptide A1	UDPGT	alpha-helical transmembrane proteins
UD11_HUMAN	UDP glucuronosyltransferase 1 family, polypeptide A3; UDP glucuronosyltransferase 1 family, polypeptide A5; UDP glucuronosyltransferase 1 family, polypeptide A4; UDP glucuronosyltransferase 1 family, polypeptide A7; UDP glucuronosyltransferase 1 family, polypeptide A6; UDP glucuronosyltransferase 1 family, polypeptide A10; UDP glucuronosyltransferase 1 family, polypeptide A9; UDP glucuronosyltransferase 1 family, polypeptide A8; UDP glucuronosyltransferase 1 family, polypeptide A1	UDPGT	alpha-helical transmembrane proteins
UD17_HUMAN	UDP glucuronosyltransferase 1 family, polypeptide A3; UDP glucuronosyltransferase 1 family, polypeptide A5; UDP glucuronosyltransferase 1 family, polypeptide A4; UDP glucuronosyltransferase 1 family, polypeptide A7; UDP glucuronosyltransferase 1 family, polypeptide A6; UDP glucuronosyltransferase 1 family, polypeptide A10; UDP glucuronosyltransferase 1 family, polypeptide A9; UDP glucuronosyltransferase 1 family, polypeptide A8; UDP glucuronosyltransferase 1 family, polypeptide A1	UDPGT	alpha-helical transmembrane proteins
Q86XA6_HUMAN	UDP-Gal:betaGlcNAc beta 1,4- galactosyltransferase, polypeptide 1	Glyco_transf_7N	N/A
B4GT1_HUMAN	UDP-Gal:betaGlcNAc beta 1,4- galactosyltransferase, polypeptide 1	Glyco_transf_7N,Glyco_transf_7C	alpha-helical transmembrane proteins
B3GN3_HUMAN	UDP-GlcNAc:betaGal beta-1,3-N-acetylglucosaminyltransferase 3	Galactosyl_T	alpha-helical transmembrane proteins
N2DL1_HUMAN	UL16 binding protein 1	MHC_I	N/A
N2DL2_HUMAN	UL16 binding protein 2	MHC_I	N/A
USH2A_HUMAN	Usher syndrome 2A (autosomal recessive, mild)	fn3,Laminin_G_2,Laminin_G_3,Laminin_EGF	alpha-helical transmembrane proteins
VSIG2_HUMAN	V-set and immunoglobulin domain containing 2	I-set,V-set	alpha-helical transmembrane proteins
VSIG4_HUMAN	V-set and immunoglobulin domain containing 4	V-set,Ig_3	alpha-helical transmembrane proteins
Q5T2L0_HUMAN	V-set domain containing T cell activation inhibitor 1	V-set	N/A
VTCN1_HUMAN	V-set domain containing T cell activation inhibitor 1	V-set	alpha-helical transmembrane proteins
VAPA_HUMAN	VAMP (vesicle-associated membrane protein)-associated protein A, 33kDa	Motile_Sperm	alpha-helical transmembrane proteins
VAPB_HUMAN	VAMP (vesicle-associated membrane protein)-associated protein B and C	Motile_Sperm	alpha-helical transmembrane proteins
XKR3_HUMAN	XK, Kell blood group complex subunit-related family, member 3	XK-related	alpha-helical transmembrane proteins
XKR2_HUMAN	XK, Kell blood group complex subunit-related, X-linked	XK-related	alpha-helical transmembrane proteins
ACVL1_HUMAN	activin A receptor type II-like 1	TGF_beta_GS,Pkinase	alpha-helical transmembrane proteins
ACVR1_HUMAN	activin A receptor, type I	TGF_beta_GS,Pkinase,Activin_recp	alpha-helical transmembrane proteins
ACV1B_HUMAN	activin A receptor, type IB	TGF_beta_GS,Pkinase,Activin_recp	alpha-helical transmembrane proteins
ACV1C_HUMAN	activin A receptor, type IC	TGF_beta_GS,Pkinase,Activin_recp	alpha-helical transmembrane proteins
AVR2A_HUMAN	activin A receptor, type IIA	Pkinase,Activin_recp	alpha-helical transmembrane proteins
AVR2B_HUMAN	activin A receptor, type IIB	Pkinase,Activin_recp	alpha-helical transmembrane proteins
ACSL1_HUMAN	acyl-CoA synthetase long-chain family member 1	AMP-binding	alpha-helical transmembrane proteins
ACSL4_HUMAN	acyl-CoA synthetase long-chain family member 4	AMP-binding	alpha-helical transmembrane proteins
ACSL6_HUMAN	acyl-CoA synthetase long-chain family member 6	AMP-binding	alpha-helical transmembrane proteins
ADCY1_HUMAN	adenylate cyclase 1 (brain)	Guanylate_cyc	alpha-helical transmembrane proteins
ADCYA_HUMAN	adenylate cyclase 10 (soluble)	Guanylate_cyc	N/A
ADCY2_HUMAN	adenylate cyclase 2 (brain)	Guanylate_cyc,DUF1053	alpha-helical transmembrane proteins
ADCY3_HUMAN	adenylate cyclase 3	Guanylate_cyc	alpha-helical transmembrane proteins
B3KT86_HUMAN	adenylate cyclase 3	Guanylate_cyc	N/A
Q6ZTM6_HUMAN	adenylate cyclase 4	Guanylate_cyc,DUF1053	N/A
ADCY4_HUMAN	adenylate cyclase 4	DUF1053,Guanylate_cyc	alpha-helical transmembrane proteins
ADCY5_HUMAN	adenylate cyclase 5	Guanylate_cyc,DUF1053	alpha-helical transmembrane proteins
ADCY6_HUMAN	adenylate cyclase 6	Guanylate_cyc,DUF1053	alpha-helical transmembrane proteins
ADCY7_HUMAN	adenylate cyclase 7	DUF1053,Guanylate_cyc	alpha-helical transmembrane proteins
ADCY8_HUMAN	adenylate cyclase 8 (brain)	Guanylate_cyc,DUF1053	alpha-helical transmembrane proteins
ADCY9_HUMAN	adenylate cyclase 9	Guanylate_cyc	alpha-helical transmembrane proteins
PACR_HUMAN	adenylate cyclase activating polypeptide 1 (pituitary) receptor type I	HRM	alpha-helical transmembrane proteins
AMGO2_HUMAN	adhesion molecule with Ig-like domain 2	LRR_8	alpha-helical transmembrane proteins
JAML1_HUMAN	adhesion molecule, interacts with CXADR antigen 1	V-set	alpha-helical transmembrane proteins

Q5SSZ3_HUMAN	advanced glycosylation end product-specific receptor	C2-set_2,Ig_2	N/A
Q5SSZ2_HUMAN	advanced glycosylation end product-specific receptor	C2-set_2,Ig_2	N/A
RAGE_HUMAN	advanced glycosylation end product-specific receptor	C2-set_2,Ig_2	alpha-helical transmembrane proteins
AMPN_HUMAN	alanyl (membrane) aminopeptidase	Peptidase_M1,DUF3358	alpha-helical transmembrane proteins
AL3A2_HUMAN	aldehyde dehydrogenase 3 family, member A2	Aldedh	alpha-helical transmembrane proteins
AOC3_HUMAN	amine oxidase, copper containing 3 (vascular adhesion protein 1)	Cu_amine_oxidN3,Cu_amine_oxidN2,Cu_amine_oxid	alpha-helical transmembrane proteins
A4_HUMAN	amyloid beta (A4) precursor protein	APP_N,APP_Cu_bd,Kunitz_BPTI,APP_E2,Beta-APP	alpha-helical transmembrane proteins
APLP1_HUMAN	amyloid beta (A4) precursor-like protein 1	APP_Cu_bd,APP_E2,APP_N	alpha-helical transmembrane proteins
APLP2_HUMAN	amyloid beta (A4) precursor-like protein 2	APP_Cu_bd,APP_N,Kunitz_BPTI,APP_E2	alpha-helical transmembrane proteins
AUP1_HUMAN	ancient ubiquitous protein 1	CUE	alpha-helical transmembrane proteins
ACE2_HUMAN	angiotensin I converting enzyme (peptidyl-dipeptidase A) 2	Asp,Peptidase_M2	alpha-helical transmembrane proteins
ANKH_HUMAN	ankylosis, progressive homolog (mouse)	ANKH	alpha-helical transmembrane proteins
ANTR1_HUMAN	anthrax toxin receptor 1	VWA,Anth_Ig	alpha-helical transmembrane proteins
ANTR2_HUMAN	anthrax toxin receptor 2	VWA,Anth_Ig	alpha-helical transmembrane proteins
AMHR2_HUMAN	anti-Mullerian hormone receptor, type II	Pkinase,Activin_recp	alpha-helical transmembrane proteins
APOB_HUMAN	apolipoprotein B (including Ag(x) antigen)	Vitellogenin_N,DUF1943	N/A
APLD1_HUMAN	apolipoprotein L domain containing 1	ApoL	alpha-helical transmembrane proteins
AQP1_HUMAN	aquaporin 1 (Colton blood group)	MIP	alpha-helical transmembrane proteins
AQP11_HUMAN	aquaporin 11	MIP	alpha-helical transmembrane proteins
AQP2_HUMAN	aquaporin 2 (collecting duct)	MIP	alpha-helical transmembrane proteins
AQP3_HUMAN	aquaporin 3 (Gill blood group)	MIP	alpha-helical transmembrane proteins
AQP4_HUMAN	aquaporin 4	MIP	alpha-helical transmembrane proteins
AQP5_HUMAN	aquaporin 5	MIP	alpha-helical transmembrane proteins
AQP6_HUMAN	aquaporin 6, kidney specific	MIP	alpha-helical transmembrane proteins
Q5T5L5_HUMAN	aquaporin 7	MIP	N/A
Q08E94_HUMAN	aquaporin 7	MIP	N/A
Q6P5T0_HUMAN	aquaporin 7	MIP	N/A
Q5T5L3_HUMAN	aquaporin 7	MIP	N/A
AQP7_HUMAN	aquaporin 7	MIP	alpha-helical transmembrane proteins
Q5T5L4_HUMAN	aquaporin 7	MIP	N/A
Q5T5M1_HUMAN	aquaporin 7	MIP	N/A
AQP8_HUMAN	aquaporin 8	MIP	alpha-helical transmembrane proteins
AQP9_HUMAN	aquaporin 9	MIP	alpha-helical transmembrane proteins
ASGR1_HUMAN	asialoglycoprotein receptor 1	Lectin_N,Lectin_C	alpha-helical transmembrane proteins
ATRN_HUMAN	attractin	Kelch_1,PSI,CUB,Kelch_4	alpha-helical transmembrane proteins
BPI_HUMAN	bactericidal/permeability-increasing protein	LBP_BPI_CETP,LBP_BPI_CETP_C	N/A
BCAM_HUMAN	basal cell adhesion molecule (Lutheran blood group)	C2-set_2,Ig_3,Ig_2	alpha-helical transmembrane proteins
BASI_HUMAN	basigin (Ok blood group)	I-set	alpha-helical transmembrane proteins
BEST1_HUMAN	bestrophin 1	Bestrophin	alpha-helical transmembrane proteins
BEST2_HUMAN	bestrophin 2	Bestrophin	alpha-helical transmembrane proteins
BEST3_HUMAN	bestrophin 3	Bestrophin	alpha-helical transmembrane proteins
BEST4_HUMAN	bestrophin 4	Bestrophin	alpha-helical transmembrane proteins
B4GN1_HUMAN	beta-1,4-N-acetyl-galactosaminyl transferase 1	Glycos_transf_2	alpha-helical transmembrane proteins
BACE1_HUMAN	beta-site APP-cleaving enzyme 1	Asp	alpha-helical transmembrane proteins
BFAR_HUMAN	bifunctional apoptosis regulator	SAM_1,zf-C3HC4_2	alpha-helical transmembrane proteins
BST1_HUMAN	bone marrow stromal cell antigen 1	Rib_hydrolayse	N/A
BMR1A_HUMAN	bone morphogenetic protein receptor, type IA; similar to ALK-3	TGF_beta_GS,Pkinase,Activin_recp	alpha-helical transmembrane proteins
BMR1B_HUMAN	bone morphogenetic protein receptor, type IB	TGF_beta_GS,Pkinase,Activin_recp	alpha-helical transmembrane proteins
BMPR2_HUMAN	bone morphogenetic protein receptor, type II (serine/threonine kinase)	Pkinase,Activin_recp	alpha-helical transmembrane proteins
BAI1_HUMAN	brain-specific angiogenesis inhibitor 1	DUF3497,TSP_1	alpha-helical transmembrane proteins
BAI2_HUMAN	brain-specific angiogenesis inhibitor 2	DUF3497,TSP_1	alpha-helical transmembrane proteins
BAI3_HUMAN	brain-specific angiogenesis inhibitor 3	DUF3497,TSP_1	alpha-helical transmembrane proteins
BT1A1_HUMAN	butyrophilin, subfamily 1, member A1	V-set,C2-set_2,PRY,SPRY	alpha-helical transmembrane proteins
BT2A1_HUMAN	butyrophilin, subfamily 2, member A1	PRY,V-set,SPRY	alpha-helical transmembrane proteins
MERTK_HUMAN	c-mer proto-oncogene tyrosine kinase	Pkinase_Tyr,I-set,fn3,Ig_2	alpha-helical transmembrane proteins
CADH1_HUMAN	cadherin 1, type 1, E-cadherin (epithelial)	Cadherin,Cadherin_pro,Cadherin_C	alpha-helical transmembrane proteins
Q9UII8_HUMAN	cadherin 1, type 1, E-cadherin (epithelial)	Cadherin,Cadherin_pro,Cadherin_C	N/A
CAD10_HUMAN	cadherin 10, type 2 (T2-cadherin)	Cadherin,Cadherin_C	alpha-helical transmembrane proteins
CAD11_HUMAN	cadherin 11, type 2, OB-cadherin (osteoblast)	Cadherin,Cadherin_C	alpha-helical transmembrane proteins
CAD12_HUMAN	cadherin 12, type 2 (N-cadherin 2)	Cadherin,Cadherin_C	alpha-helical transmembrane proteins
CAD15_HUMAN	cadherin 15, type 1, M-cadherin (myotubule)	Cadherin,Cadherin_C	alpha-helical transmembrane proteins
CAD16_HUMAN	cadherin 16, KSP-cadherin	Cadherin	alpha-helical transmembrane proteins
CAD17_HUMAN	cadherin 17, LI cadherin (liver-intestine)	Cadherin	alpha-helical transmembrane proteins
CAD18_HUMAN	cadherin 18, type 2	Cadherin,Cadherin_C	alpha-helical transmembrane proteins
CAD19_HUMAN	cadherin 19, type 2	Cadherin,Cadherin_C	alpha-helical transmembrane proteins
CADH2_HUMAN	cadherin 2, type 1, N-cadherin (neuronal)	Cadherin,Cadherin_pro,Cadherin_C	alpha-helical transmembrane proteins
CAD20_HUMAN	cadherin 20, type 2	Cadherin,Cadherin_C	alpha-helical transmembrane proteins
CADH3_HUMAN	cadherin 3, type 1, P-cadherin (placental)	Cadherin,Cadherin_C	alpha-helical transmembrane proteins
CADH4_HUMAN	cadherin 4, type 1, R-cadherin (retinal)	Cadherin,Cadherin_C,Cadherin_pro	alpha-helical transmembrane proteins
CADH5_HUMAN	cadherin 5, type 2 (vascular endothelium)	Cadherin,Cadherin_C	alpha-helical transmembrane proteins
CADH6_HUMAN	cadherin 6, type 2, K-cadherin (fetal kidney)	Cadherin,Cadherin_C	alpha-helical transmembrane proteins
CADH7_HUMAN	cadherin 7, type 2	Cadherin,Cadherin_C	alpha-helical transmembrane proteins
CADH8_HUMAN	cadherin 8, type 2	Cadherin,Cadherin_C	alpha-helical transmembrane proteins
CADH9_HUMAN	cadherin 9, type 2 (T1-cadherin)	Cadherin,Cadherin_C	alpha-helical transmembrane proteins
CELR1_HUMAN	cadherin, EGF LAG seven-pass G-type receptor 1 (flamingo homolog, Drosophila)	Cadherin,DUF3497,Laminin_G_2,EGF,Laminin_EGF	alpha-helical transmembrane proteins
CELR2_HUMAN	cadherin, EGF LAG seven-pass G-type receptor 2 (flamingo homolog, Drosophila)	Cadherin,DUF3497,Laminin_G_2,EGF,Laminin_EGF	alpha-helical transmembrane proteins
CAD22_HUMAN	cadherin-like 22	Cadherin,Cadherin_C	alpha-helical transmembrane proteins
CAD23_HUMAN	cadherin-like 23	Cadherin	alpha-helical transmembrane proteins
CAD24_HUMAN	cadherin-like 24	Cadherin,Cadherin_C	alpha-helical transmembrane proteins
CAD26_HUMAN	cadherin-like 26	Cadherin	alpha-helical transmembrane proteins
A4D1G6_HUMAN	calcitonin receptor	HRM	N/A
CALCR_HUMAN	calcitonin receptor	HRM	alpha-helical transmembrane proteins
CALRL_HUMAN	calcitonin receptor-like	HRM	alpha-helical transmembrane proteins
CASR_HUMAN	calcium-sensing receptor	NCD3G,ANF_receptor	alpha-helical transmembrane proteins
CALY_HUMAN	calcyon neuron-specific vesicular protein	Calcyon	alpha-helical transmembrane proteins
Q5SR54_HUMAN	calsyntenin 1	Cadherin,Laminin_G_3	N/A
CSTN1_HUMAN	calsyntenin 1	Cadherin,Laminin_G_3	alpha-helical transmembrane proteins
CSTN2_HUMAN	calsyntenin 2	Cadherin,Laminin_G_3	alpha-helical transmembrane proteins
CSTN3_HUMAN	calsyntenin 3	Laminin_G_3	alpha-helical transmembrane proteins
CAH4_HUMAN	carbonic anhydrase IV	Carb_anhydrase	N/A
CAH9_HUMAN	carbonic anhydrase IX	Carb_anhydrase	alpha-helical transmembrane proteins
CAH12_HUMAN	carbonic anhydrase XII	Carb_anhydrase	alpha-helical transmembrane proteins
CEAM1_HUMAN	carcinoembryonic antigen-related cell adhesion molecule 1 (biliary glycoprotein)	V-set,Ig_3,ig,Ig_2	alpha-helical transmembrane proteins
CEAM4_HUMAN	carcinoembryonic antigen-related cell adhesion molecule 4	V-set	alpha-helical transmembrane proteins
COMT_HUMAN	catechol-O-methyltransferase	Methyltransf_3	alpha-helical transmembrane proteins
CADM1_HUMAN	cell adhesion molecule 1	C2-set_2,V-set,Ig_2	alpha-helical transmembrane proteins
CADM3_HUMAN	cell adhesion molecule 3	C2-set_2,V-set,Ig_2	alpha-helical transmembrane proteins
CHL1_HUMAN	cell adhesion molecule with homology to L1CAM (close homolog of L1)	fn3,Pro-MCH,I-set,Bravo_FIGEY,Ig_2,S_100	alpha-helical transmembrane proteins
Q9UBD8_HUMAN	ceroid-lipofuscinosis, neuronal 3	CLN3	N/A
CLN3_HUMAN	ceroid-lipofuscinosis, neuronal 3	PKD_channel,CLN3	alpha-helical transmembrane proteins
Q2TA70_HUMAN	ceroid-lipofuscinosis, neuronal 3	CLN3	N/A
X3CL1_HUMAN	chemokine (C-X3-C motif) ligand 1	IL8	alpha-helical transmembrane proteins
CLCN1_HUMAN	chloride channel 1, skeletal muscle	Voltage_CLC	alpha-helical transmembrane proteins
CLCN3_HUMAN	chloride channel 3	CBS,Voltage_CLC	alpha-helical transmembrane proteins
CLCN5_HUMAN	chloride channel 5	CBS,Voltage_CLC	alpha-helical transmembrane proteins
CLCKA_HUMAN	chloride channel Ka	Voltage_CLC	alpha-helical transmembrane proteins
CLCKB_HUMAN	chloride channel Kb	CBS,Voltage_CLC	alpha-helical transmembrane proteins
CLCA2_HUMAN	chloride channel accessory 2	VWA_2,CLCA_N,DUF1973	alpha-helical transmembrane proteins
CLCA4_HUMAN	chloride channel accessory 4	CLCA_N,VWA_2,DUF1973	alpha-helical transmembrane proteins
ACHA_HUMAN	cholinergic receptor, nicotinic, alpha 1 (muscle)	Neur_chan_LBD,Neur_chan_memb	alpha-helical transmembrane proteins
ACH10_HUMAN	cholinergic receptor, nicotinic, alpha 10	Neur_chan_LBD,Neur_chan_memb	alpha-helical transmembrane proteins
ACHA2_HUMAN	cholinergic receptor, nicotinic, alpha 2 (neuronal)	Neur_chan_LBD,Neur_chan_memb	alpha-helical transmembrane proteins
ACHA3_HUMAN	cholinergic receptor, nicotinic, alpha 3	Neur_chan_LBD,Neur_chan_memb	alpha-helical transmembrane proteins

ACHA4_HUMAN	cholinergic receptor, nicotinic, alpha 4	Neur_chan_LBD,Neur_chan_memb	alpha-helical transmembrane proteins
ACHA5_HUMAN	cholinergic receptor, nicotinic, alpha 5	Neur_chan_LBD,Neur_chan_memb	alpha-helical transmembrane proteins
ACHA6_HUMAN	cholinergic receptor, nicotinic, alpha 6	Neur_chan_LBD,Neur_chan_memb	alpha-helical transmembrane proteins
ACHA9_HUMAN	cholinergic receptor, nicotinic, alpha 9	Neur_chan_LBD,Neur_chan_memb	alpha-helical transmembrane proteins
ACHB_HUMAN	cholinergic receptor, nicotinic, beta 1 (muscle)	Neur_chan_LBD,Neur_chan_memb	alpha-helical transmembrane proteins
ACHB2_HUMAN	cholinergic receptor, nicotinic, beta 2 (neuronal)	Neur_chan_LBD,Neur_chan_memb	alpha-helical transmembrane proteins
ACHB3_HUMAN	cholinergic receptor, nicotinic, beta 3	Neur_chan_LBD,Neur_chan_memb	alpha-helical transmembrane proteins
ACHB4_HUMAN	cholinergic receptor, nicotinic, beta 4	Neur_chan_LBD,Neur_chan_memb	alpha-helical transmembrane proteins
ACHD_HUMAN	cholinergic receptor, nicotinic, delta	Neur_chan_LBD,Neur_chan_memb	alpha-helical transmembrane proteins
ACHE_HUMAN	cholinergic receptor, nicotinic, epsilon	Neur_chan_LBD,Neur_chan_memb	alpha-helical transmembrane proteins
ACHG_HUMAN	cholinergic receptor, nicotinic, gamma	Neur_chan_LBD,Neur_chan_memb	alpha-helical transmembrane proteins
CSPG4_HUMAN	chondroitin sulfate proteoglycan 4	Laminin_G_2,Laminin_G_1	alpha-helical transmembrane proteins
CLPT1_HUMAN	cleft lip and palate associated transmembrane protein 1	CLPTM1	alpha-helical transmembrane proteins
TF_HUMAN	coagulation factor III (thromboplastin, tissue factor)	Cystatin,Helicase_C,bZIP_1,Bromodomain,zf-C3HC4_2,Sulfotransfer_1,Tissue_fac,Peptidase_C1,Interfer-bind	alpha-helical transmembrane proteins
FA10_HUMAN	coagulation factor X	Gla,Trypsin,EGF	N/A
COHA1_HUMAN	collagen, type XVII, alpha 1	Collagen	alpha-helical transmembrane proteins
COPA1_HUMAN	collagen, type XXV, alpha 1	Collagen	alpha-helical transmembrane proteins
CSF1_HUMAN	colony stimulating factor 1 (macrophage)	CSF-1	alpha-helical transmembrane proteins
CSF1R_HUMAN	colony stimulating factor 1 receptor	Pkinase_Tyr,ig,Ig_3	alpha-helical transmembrane proteins
CSF2R_HUMAN	colony stimulating factor 2 receptor, alpha, low-affinity (granulocyte-macrophage)	IL6Ra-bind	alpha-helical transmembrane proteins
IL3RB_HUMAN	colony stimulating factor 2 receptor, beta, low-affinity (granulocyte-macrophage)	IL6Ra-bind,fn3	alpha-helical transmembrane proteins
CSF3R_HUMAN	colony stimulating factor 3 receptor (granulocyte)	fn3,Lep_receptor_Ig	alpha-helical transmembrane proteins
Q5SR44_HUMAN	complement component (3b/4b) receptor 1 (Knops blood group)	Sushi	N/A
CR1_HUMAN	complement component (3b/4b) receptor 1 (Knops blood group)	A_deaminase,Proteasome,F420_oxidored,Sushi,NAD_binding_4,A_deaminase_N	alpha-helical transmembrane proteins
CR2_HUMAN	complement component (3d/Epstein Barr virus) receptor 2	F420_oxidored,Sushi,NAD_binding_4,Bromodomain	alpha-helical transmembrane proteins
CO8B_HUMAN	complement component 8, beta polypeptide	MACPF,Ldl_recept_a	N/A
CNTP1_HUMAN	contactin associated protein 1	F5_F8_type_C,Laminin_G_2	alpha-helical transmembrane proteins
CNTP3_HUMAN	contactin associated protein-like 3; contactin associated protein-like 3B	F5_F8_type_C,Laminin_G_2	alpha-helical transmembrane proteins
CORIN_HUMAN	corin, serine peptidase	Fz,Ldl_recept_a,Trypsin	alpha-helical transmembrane proteins
CRFR1_HUMAN	corticotropin releasing hormone receptor 1	HRM	alpha-helical transmembrane proteins
CRFR2_HUMAN	corticotropin releasing hormone receptor 2	HRM	alpha-helical transmembrane proteins
CXAR_HUMAN	coxsackie virus and adenovirus receptor pseudogene 2; coxsackie virus and adenovirus receptor	V-set,Ig_2	alpha-helical transmembrane proteins
CRUM1_HUMAN	crumbs homolog 1 (Drosophila)	Laminin_G_2,EGF	alpha-helical transmembrane proteins
CRUM2_HUMAN	crumbs homolog 2 (Drosophila)	Laminin_G_2,EGF	alpha-helical transmembrane proteins
CNGA1_HUMAN	cyclic nucleotide gated channel alpha 1	cNMP_binding	alpha-helical transmembrane proteins
CNNM3_HUMAN	cyclin M3	DUF21	alpha-helical transmembrane proteins
CNNM4_HUMAN	cyclin M4	DUF21	alpha-helical transmembrane proteins
CRIM1_HUMAN	cysteine rich transmembrane BMP regulator 1 (chordin-like)	VWC	alpha-helical transmembrane proteins
CHIC1_HUMAN	cysteine-rich hydrophobic domain 1	Erf4	N/A
CHIC2_HUMAN	cysteine-rich hydrophobic domain 2	Erf4	N/A
CFTR_HUMAN	cystic fibrosis transmembrane conductance regulator (ATP-binding cassette sub-family C, member 7)	ABC_tran	alpha-helical transmembrane proteins
CY24B_HUMAN	cytochrome b-245, beta polypeptide	NAD_binding_6,FAD_binding_8	alpha-helical transmembrane proteins
CYB5_HUMAN	cytochrome b5 type A (microsomal)	Cyt-b5	alpha-helical transmembrane proteins
CTLA4_HUMAN	cytotoxic T-lymphocyte-associated protein 4	V-set	alpha-helical transmembrane proteins
CRTAM_HUMAN	cytotoxic and regulatory T cell molecule	C2-set_2,V-set	alpha-helical transmembrane proteins
PCD16_HUMAN	dachsous 1 (Drosophila)	Cadherin	alpha-helical transmembrane proteins
DEGS1_HUMAN	degenerative spermatocyte homolog 1, lipid desaturase (Drosophila)	Lipid_DES,FA_desaturase	alpha-helical transmembrane proteins
IOD1_HUMAN	deiodinase, iodothyronine, type I	T4_deiodinase	alpha-helical transmembrane proteins
IOD3_HUMAN	deiodinase, iodothyronine, type III	T4_deiodinase	alpha-helical transmembrane proteins
DLL1_HUMAN	delta-like 1 (Drosophila)	EGF,MNNL	alpha-helical transmembrane proteins
DNER_HUMAN	delta/notch-like EGF repeat containing	EGF	alpha-helical transmembrane proteins
DSC1_HUMAN	desmocollin 1	Cadherin,Cadherin_pro,Cadherin_C	alpha-helical transmembrane proteins
DSC2_HUMAN	desmocollin 2	Cadherin,Cadherin_pro,Cadherin_C	alpha-helical transmembrane proteins
DSC3_HUMAN	desmocollin 3	Cadherin,Cadherin_pro,Cadherin_C	alpha-helical transmembrane proteins
DSG2_HUMAN	desmoglein 2	Cadherin,Cadherin_C	alpha-helical transmembrane proteins
DSG4_HUMAN	desmoglein 4	Cadherin,Cadherin_C	alpha-helical transmembrane proteins
DGLA_HUMAN	diacylglycerol lipase, alpha	Lipase_3	alpha-helical transmembrane proteins
DGLB_HUMAN	diacylglycerol lipase, beta	Lipase_3	alpha-helical transmembrane proteins
DPP4_HUMAN	dipeptidyl-peptidase 4	Peptidase_S9,DPPIV_N	alpha-helical transmembrane proteins
DDR1_HUMAN	discoidin domain receptor tyrosine kinase 1	Pkinase_Tyr,F5_F8_type_C	alpha-helical transmembrane proteins
Q6ZNR9_HUMAN	discoidin domain receptor tyrosine kinase 1	Pkinase_Tyr	N/A
DDR2_HUMAN	discoidin domain receptor tyrosine kinase 2	Pkinase_Tyr,F5_F8_type_C	alpha-helical transmembrane proteins
DCBD2_HUMAN	discoidin, CUB and LCCL domain containing 2	F5_F8_type_C,LCCL,CUB	alpha-helical transmembrane proteins
DUOX1_HUMAN	dual oxidase 1	NAD_binding_6,EF_hand_5,An_peroxidase,FAD_binding_8	alpha-helical transmembrane proteins
DUOX2_HUMAN	dual oxidase 2	NAD_binding_6,EF_hand_5,An_peroxidase,FAD_binding_8	alpha-helical transmembrane proteins
DYSF_HUMAN	dysferlin, limb girdle muscular dystrophy 2B (autosomal recessive)	FerA,C2,FerB,Pex24p	alpha-helical transmembrane proteins
Q5T0V7_HUMAN	dystonin	CH	N/A
EDA_HUMAN	ectodysplasin A	Collagen,EMP24_GP25L,TNF	alpha-helical transmembrane proteins
EDAR_HUMAN	ectodysplasin A receptor	Death	alpha-helical transmembrane proteins
ENTP1_HUMAN	ectonucleoside triphosphate diphosphohydrolase 1	GDA1_CD39	alpha-helical transmembrane proteins
ENTP3_HUMAN	ectonucleoside triphosphate diphosphohydrolase 3	GDA1_CD39	alpha-helical transmembrane proteins
ENTP8_HUMAN	ectonucleoside triphosphate diphosphohydrolase 8	GDA1_CD39	alpha-helical transmembrane proteins
ENPP1_HUMAN	ectonucleotide pyrophosphatase/phosphodiesterase 1	Somatomedin_B,Phosphodiest,Endonuclease_NS	alpha-helical transmembrane proteins
ENPP2_HUMAN	ectonucleotide pyrophosphatase/phosphodiesterase 2	Somatomedin_B,Phosphodiest,Endonuclease_NS	N/A
ENPP3_HUMAN	ectonucleotide pyrophosphatase/phosphodiesterase 3	Somatomedin_B,Phosphodiest,Endonuclease_NS	alpha-helical transmembrane proteins
ENPP5_HUMAN	ectonucleotide pyrophosphatase/phosphodiesterase 5 (putative function)	Phosphodiest	alpha-helical transmembrane proteins
EMR1_HUMAN	egf-like module containing, mucin-like, hormone receptor-like 1	EGF_CA	alpha-helical transmembrane proteins
EMR2_HUMAN	egf-like module containing, mucin-like, hormone receptor-like 2	EGF_CA	alpha-helical transmembrane proteins
EMR3_HUMAN	egf-like module containing, mucin-like, hormone receptor-like 3	HRM,EGF_CA,OLF,Gal_Lectin,DUF3497,Latrophilin	alpha-helical transmembrane proteins
ENW1_HUMAN	endogenous retroviral family W, env(C7), member 1	TLV_coat	alpha-helical transmembrane proteins
ENK11_HUMAN	endogenous retroviral sequence K, 6	HERV-K_env_2	alpha-helical transmembrane proteins
EGLN_HUMAN	endoglin	Zona_pellucida	alpha-helical transmembrane proteins
MUCEN_HUMAN	endomucin	Endomucin	alpha-helical transmembrane proteins
ESAM_HUMAN	endothelial cell adhesion molecule	V-set,Ig_2	alpha-helical transmembrane proteins
ECE1_HUMAN	endothelin converting enzyme 1	Peptidase_M13_N,Peptidase_M13	alpha-helical transmembrane proteins
ECEL1_HUMAN	endothelin converting enzyme-like 1	Peptidase_M13_N,Peptidase_M13	alpha-helical transmembrane proteins
EFNB1_HUMAN	ephrin-B1	Ephrin	alpha-helical transmembrane proteins
EFNB2_HUMAN	ephrin-B2	Ephrin	alpha-helical transmembrane proteins
EFNB3_HUMAN	ephrin-B3	Ephrin	alpha-helical transmembrane proteins
EGF_HUMAN	epidermal growth factor (beta-urogastrone)	EGF_CA,EGF,Ldl_recept_b	alpha-helical transmembrane proteins
EGFR_HUMAN	epidermal growth factor receptor (erythroblastic leukemia viral (v-erb-b) oncogene homolog, avian)	Pkinase_Tyr,Furin-like,Recep_L_domain	alpha-helical transmembrane proteins
Q504U8_HUMAN	epidermal growth factor receptor (erythroblastic leukemia viral (v-erb-b) oncogene homolog, avian)	Pkinase_Tyr,Furin-like,Recep_L_domain	N/A
EPCAM_HUMAN	epithelial cell adhesion molecule	Thyroglobulin_1	alpha-helical transmembrane proteins
ERMAP_HUMAN	erythroblast membrane-associated protein (Scianna blood group)	PRY,V-set,SPRY	alpha-helical transmembrane proteins
EPOR_HUMAN	erythropoietin receptor	EpoR_lig-bind,fn3	alpha-helical transmembrane proteins
FA57A_HUMAN	family with sequence similarity 57, member A	TRAM_LAG1_CLN8	alpha-helical transmembrane proteins
ESYT2_HUMAN	family with sequence similarity 62 (C2 domain containing), member B	C2	alpha-helical transmembrane proteins
ESYT3_HUMAN	family with sequence similarity 62 (C2 domain containing), member C	C2	alpha-helical transmembrane proteins
FADS2_HUMAN	fatty acid desaturase 2	Cyt-b5,FA_desaturase	alpha-helical transmembrane proteins
FLVC1_HUMAN	feline leukemia virus subgroup C cellular receptor 1	MFS_1	alpha-helical transmembrane proteins
FLVC2_HUMAN	feline leukemia virus subgroup C cellular receptor family, member 2	MFS_1	alpha-helical transmembrane proteins
FIBG_HUMAN	fibrinogen gamma chain	Fibrinogen_C,Fib_alpha	N/A
SEPR_HUMAN	fibroblast activation protein, alpha	Peptidase_S9,DPPIV_N	alpha-helical transmembrane proteins
FGF6_HUMAN	fibroblast growth factor 6	FGF	N/A
FGFR1_HUMAN	fibroblast growth factor receptor 1	Pkinase_Tyr,I-set	alpha-helical transmembrane proteins
FGFR2_HUMAN	fibroblast growth factor receptor 2	Pkinase_Tyr,I-set,Ig_2	alpha-helical transmembrane proteins
FGFR3_HUMAN	fibroblast growth factor receptor 3	Pkinase_Tyr,I-set,ig	alpha-helical transmembrane proteins
FGRL1_HUMAN	fibroblast growth factor receptor-like 1	I-set	alpha-helical transmembrane proteins
FLRT1_HUMAN	fibronectin leucine rich transmembrane protein 1	LRR_8,LRRNT	alpha-helical transmembrane proteins

FLRT2_HUMAN	fibronectin leucine rich transmembrane protein 2	LRR_8,fn3	alpha-helical transmembrane proteins
FLRT3_HUMAN	fibronectin leucine rich transmembrane protein 3	LRR_8	alpha-helical transmembrane proteins
Q5VTU6_HUMAN	fms-related tyrosine kinase 3	Pkinase_Tyr,ig	N/A
FLT3_HUMAN	fms-related tyrosine kinase 3	Pkinase_Tyr,ig	alpha-helical transmembrane proteins
FLT3L_HUMAN	fms-related tyrosine kinase 3 ligand	Flt3_lig	alpha-helical transmembrane proteins
VGFR3_HUMAN	fms-related tyrosine kinase 4	Pkinase_Tyr,I-set,Ig_2	alpha-helical transmembrane proteins
FOLH1_HUMAN	folate hydrolase (prostate-specific membrane antigen) 1	TFR_dimer,Peptidase_M28,PA	alpha-helical transmembrane proteins
FOLR1_HUMAN	folate receptor 1 (adult)	Folate_rec	N/A
FSHR_HUMAN	follicle stimulating hormone receptor	GnHR_trans,LRR_8,LRRNT	alpha-helical transmembrane proteins
FZD10_HUMAN	frizzled homolog 10 (Drosophila)	Fz	alpha-helical transmembrane proteins
FZD2_HUMAN	frizzled homolog 2 (Drosophila)	Fz	alpha-helical transmembrane proteins
FZD3_HUMAN	frizzled homolog 3 (Drosophila)	Fz	alpha-helical transmembrane proteins
FZD5_HUMAN	frizzled homolog 5 (Drosophila)	Fz	alpha-helical transmembrane proteins
FZD6_HUMAN	frizzled homolog 6 (Drosophila)	Fz	alpha-helical transmembrane proteins
FZD7_HUMAN	frizzled homolog 7 (Drosophila)	Fz	alpha-helical transmembrane proteins
FZD9_HUMAN	frizzled homolog 9 (Drosophila)	Fz	alpha-helical transmembrane proteins
FURIN_HUMAN	furin (paired basic amino acid cleaving enzyme)	P_proprotein,Peptidase_S8	alpha-helical transmembrane proteins
G3ST1_HUMAN	galactose-3-O-sulfotransferase 1	Gal-3-0_sulfotr	alpha-helical transmembrane proteins
GBRA1_HUMAN	gamma-aminobutyric acid (GABA) A receptor, alpha 1	Neur_chan_LBD,Neur_chan_memb	alpha-helical transmembrane proteins
GBRA2_HUMAN	gamma-aminobutyric acid (GABA) A receptor, alpha 2	Neur_chan_LBD,Neur_chan_memb	alpha-helical transmembrane proteins
GBRA3_HUMAN	gamma-aminobutyric acid (GABA) A receptor, alpha 3	Neur_chan_LBD,Neur_chan_memb	alpha-helical transmembrane proteins
GBRA4_HUMAN	gamma-aminobutyric acid (GABA) A receptor, alpha 4	Neur_chan_LBD,Neur_chan_memb	alpha-helical transmembrane proteins
GBRA5_HUMAN	gamma-aminobutyric acid (GABA) A receptor, alpha 5	Neur_chan_LBD,Neur_chan_memb	alpha-helical transmembrane proteins
GBRA6_HUMAN	gamma-aminobutyric acid (GABA) A receptor, alpha 6	Neur_chan_LBD,Neur_chan_memb	alpha-helical transmembrane proteins
GBRB1_HUMAN	gamma-aminobutyric acid (GABA) A receptor, beta 1	Neur_chan_LBD,Neur_chan_memb	alpha-helical transmembrane proteins
GBRB2_HUMAN	gamma-aminobutyric acid (GABA) A receptor, beta 2	Neur_chan_LBD,Neur_chan_memb	alpha-helical transmembrane proteins
GBRB3_HUMAN	gamma-aminobutyric acid (GABA) A receptor, beta 3	Neur_chan_LBD,Neur_chan_memb	alpha-helical transmembrane proteins
GBRD_HUMAN	gamma-aminobutyric acid (GABA) A receptor, delta	Neur_chan_LBD,Neur_chan_memb	alpha-helical transmembrane proteins
GBRE_HUMAN	gamma-aminobutyric acid (GABA) A receptor, epsilon	Neur_chan_LBD,Neur_chan_memb	alpha-helical transmembrane proteins
GBRG1_HUMAN	gamma-aminobutyric acid (GABA) A receptor, gamma 1	Neur_chan_LBD,Neur_chan_memb	alpha-helical transmembrane proteins
GBRG2_HUMAN	gamma-aminobutyric acid (GABA) A receptor, gamma 2	Neur_chan_LBD,Neur_chan_memb	alpha-helical transmembrane proteins
GBRG3_HUMAN	gamma-aminobutyric acid (GABA) A receptor, gamma 3	Neur_chan_LBD,Neur_chan_memb	alpha-helical transmembrane proteins
GBRP_HUMAN	gamma-aminobutyric acid (GABA) A receptor, pi	Neur_chan_LBD,Neur_chan_memb	alpha-helical transmembrane proteins
GABR1_HUMAN	gamma-aminobutyric acid (GABA) B receptor, 1	Sushi,ANF_receptor	alpha-helical transmembrane proteins
GABR2_HUMAN	gamma-aminobutyric acid (GABA) B receptor, 2	ANF_receptor	alpha-helical transmembrane proteins
GBRR1_HUMAN	gamma-aminobutyric acid (GABA) receptor, rho 1	Neur_chan_LBD,Neur_chan_memb	alpha-helical transmembrane proteins
GBRR2_HUMAN	gamma-aminobutyric acid (GABA) receptor, rho 2	Neur_chan_LBD,Neur_chan_memb	alpha-helical transmembrane proteins
GBRR3_HUMAN	gamma-aminobutyric acid (GABA) receptor, rho 3	Neur_chan_LBD,Neur_chan_memb	alpha-helical transmembrane proteins
GBRT_HUMAN	gamma-aminobutyric acid (GABA) receptor, theta	Neur_chan_LBD,Neur_chan_memb	alpha-helical transmembrane proteins
GGT5_HUMAN	gamma-glutamyltransferase 5	G_glu_transpept	alpha-helical transmembrane proteins
GIPR_HUMAN	gastric inhibitory polypeptide receptor	HRM	alpha-helical transmembrane proteins
GLDN_HUMAN	gliomedin	Collagen,OLF	alpha-helical transmembrane proteins
GLR_HUMAN	glucagon receptor	HRM,Glyco_hydro_2_N,Glyco_hydro_2_C	alpha-helical transmembrane proteins
GLP1R_HUMAN	glucagon-like peptide 1 receptor	HRM	alpha-helical transmembrane proteins
GLP2R_HUMAN	glucagon-like peptide 2 receptor	HRM	alpha-helical transmembrane proteins
GRIA3_HUMAN	glutamate receptor, ionotrophic, AMPA 3	Lig_chan-Glu_bd,ANF_receptor	alpha-helical transmembrane proteins
GRIA4_HUMAN	glutamate receptor, ionotrophic, AMPA 4	Lig_chan-Glu_bd,ANF_receptor	alpha-helical transmembrane proteins
GRIA1_HUMAN	glutamate receptor, ionotropic, AMPA 1	SBP_bac_3,ANF_receptor	alpha-helical transmembrane proteins
GRIA2_HUMAN	glutamate receptor, ionotropic, AMPA 2	Lig_chan-Glu_bd,ANF_receptor	alpha-helical transmembrane proteins
Q5VSF9_HUMAN	glutamate receptor, ionotropic, N-methyl D-aspartate 1	SBP_bac_3,ANF_receptor	N/A
NMDZ1_HUMAN	glutamate receptor, ionotropic, N-methyl D-aspartate 1	SBP_bac_3,ANF_receptor	alpha-helical transmembrane proteins
NMDE1_HUMAN	glutamate receptor, ionotropic, N-methyl D-aspartate 2A	SBP_bac_3,ANF_receptor	alpha-helical transmembrane proteins
NMDE2_HUMAN	glutamate receptor, ionotropic, N-methyl D-aspartate 2B	SBP_bac_3,ANF_receptor	alpha-helical transmembrane proteins
NMDE3_HUMAN	glutamate receptor, ionotropic, N-methyl D-aspartate 2C	SBP_bac_3,ANF_receptor	alpha-helical transmembrane proteins
NMDE4_HUMAN	glutamate receptor, ionotropic, N-methyl D-aspartate 2D	SBP_bac_3,ANF_receptor	alpha-helical transmembrane proteins
NMD3A_HUMAN	glutamate receptor, ionotropic, N-methyl-D-aspartate 3A	SBP_bac_3	alpha-helical transmembrane proteins
NMD3B_HUMAN	glutamate receptor, ionotropic, N-methyl-D-aspartate 3B	SBP_bac_3	alpha-helical transmembrane proteins
GRID1_HUMAN	glutamate receptor, ionotropic, delta 1	SBP_bac_3,ANF_receptor	alpha-helical transmembrane proteins
GRID2_HUMAN	glutamate receptor, ionotropic, delta 2	SBP_bac_3,ANF_receptor	alpha-helical transmembrane proteins
GRIK1_HUMAN	glutamate receptor, ionotropic, kainate 1	Lig_chan-Glu_bd,ANF_receptor	alpha-helical transmembrane proteins
GRIK2_HUMAN	glutamate receptor, ionotropic, kainate 2	Lig_chan-Glu_bd,ANF_receptor	alpha-helical transmembrane proteins
A9Z1Z8_HUMAN	glutamate receptor, ionotropic, kainate 3	Lig_chan-Glu_bd,ANF_receptor	N/A
GRIK3_HUMAN	glutamate receptor, ionotropic, kainate 3	Lig_chan-Glu_bd,ANF_receptor	alpha-helical transmembrane proteins
GRIK4_HUMAN	glutamate receptor, ionotropic, kainate 4	Lig_chan-Glu_bd,ANF_receptor	alpha-helical transmembrane proteins
GRIK5_HUMAN	glutamate receptor, ionotropic, kainate 5	Lig_chan-Glu_bd,ANF_receptor	alpha-helical transmembrane proteins
GRM1_HUMAN	glutamate receptor, metabotropic 1	NCD3G,ANF_receptor	alpha-helical transmembrane proteins
GRM2_HUMAN	glutamate receptor, metabotropic 2	NCD3G,ANF_receptor	alpha-helical transmembrane proteins
GRM3_HUMAN	glutamate receptor, metabotropic 3	NCD3G,ANF_receptor	alpha-helical transmembrane proteins
GRM4_HUMAN	glutamate receptor, metabotropic 4	NCD3G,ANF_receptor	alpha-helical transmembrane proteins
GRM5_HUMAN	glutamate receptor, metabotropic 5	NCD3G,ANF_receptor	alpha-helical transmembrane proteins
GRM6_HUMAN	glutamate receptor, metabotropic 6	NCD3G,ANF_receptor	alpha-helical transmembrane proteins
GRM7_HUMAN	glutamate receptor, metabotropic 7	NCD3G,ANF_receptor	alpha-helical transmembrane proteins
GRM8_HUMAN	glutamate receptor, metabotropic 8	NCD3G,ANF_receptor	alpha-helical transmembrane proteins
AMPE_HUMAN	glutamyl aminopeptidase (aminopeptidase A)	Peptidase_M1,DUF3358	alpha-helical transmembrane proteins
GDPD2_HUMAN	glycerophosphodiester phosphodiesterase domain containing 2	GDPD	alpha-helical transmembrane proteins
Q14C71_HUMAN	glycine receptor, alpha 1	Neur_chan_LBD,Neur_chan_memb	N/A
GLRA1_HUMAN	glycine receptor, alpha 1	Neur_chan_LBD,Neur_chan_memb	alpha-helical transmembrane proteins
GLRA2_HUMAN	glycine receptor, alpha 2	Neur_chan_LBD,Neur_chan_memb	alpha-helical transmembrane proteins
GLRA3_HUMAN	glycine receptor, alpha 3	Neur_chan_LBD,Neur_chan_memb	alpha-helical transmembrane proteins
GLRA4_HUMAN	glycine receptor, alpha 4	Neur_chan_LBD,Neur_chan_memb	alpha-helical transmembrane proteins
GLRB_HUMAN	glycine receptor, beta	Neur_chan_LBD,Neur_chan_memb	alpha-helical transmembrane proteins
GPNMB_HUMAN	glycoprotein (transmembrane) nmb	PKD	alpha-helical transmembrane proteins
GP2_HUMAN	glycoprotein 2 (zymogen granule membrane)	cNMP_binding,PAN_1,RhoGAP,Zona_pellucida,FCH,Trypsin,Fibrinogen_C,Pkinase	N/A
GPA33_HUMAN	glycoprotein A33 (transmembrane)	V-set,Ig_2	alpha-helical transmembrane proteins
GPIX_HUMAN	glycoprotein IX (platelet)	LRR_8,LRRNT	alpha-helical transmembrane proteins
GP1BA_HUMAN	glycoprotein Ib (platelet), alpha polypeptide	LRR_8,LRRNT	alpha-helical transmembrane proteins
GP1BB_HUMAN	glycoprotein Ib (platelet), beta polypeptide	LRRNT	alpha-helical transmembrane proteins
GPV_HUMAN	glycoprotein V (platelet)	LRR_8	alpha-helical transmembrane proteins
GPVI_HUMAN	glycoprotein VI (platelet)	Ig_3,Ig_2	alpha-helical transmembrane proteins
GHR_HUMAN	growth hormone receptor	EpoR_lig-bind,fn3	alpha-helical transmembrane proteins
GHRHR_HUMAN	growth hormone releasing hormone receptor	HRM	alpha-helical transmembrane proteins
GUC2D_HUMAN	guanylate cyclase 2D, membrane (retina-specific)	Pkinase_Tyr,HNOBA,ANF_receptor,Guanylate_cyc	alpha-helical transmembrane proteins
GUC2F_HUMAN	guanylate cyclase 2F, retinal	Pkinase_Tyr,HNOBA,ANF_receptor,Guanylate_cyc	alpha-helical transmembrane proteins
HMOX1_HUMAN	heme oxygenase (decycling) 1	Heme_oxygenase	N/A
HMOX2_HUMAN	heme oxygenase (decycling) 2	Heme_oxygenase	N/A
HFE_HUMAN	hemochromatosis	C1-set,MHC_I	alpha-helical transmembrane proteins
Q6B0J5_HUMAN	hemochromatosis	C1-set,MHC_I	N/A
RGMC_HUMAN	hemochromatosis type 2 (juvenile)	RGM_C,RGM_N	N/A
HS3SB_HUMAN	heparan sulfate (glucosamine) 3-O-sulfotransferase 3B1	Sulfotransfer_1	alpha-helical transmembrane proteins
H6ST1_HUMAN	heparan sulfate 6-O-sulfotransferase 1	Sulfotransfer_2	alpha-helical transmembrane proteins
HECAM_HUMAN	hepatocyte cell adhesion molecule; HEPACAM opposite strand 1	V-set,Ig_2	alpha-helical transmembrane proteins
HEPS_HUMAN	hepsin	Trypsin,Hepsin-SRCR	alpha-helical transmembrane proteins
HCN2_HUMAN	hyperpolarization activated cyclic nucleotide-gated potassium channel 2	cNMP_binding	alpha-helical transmembrane proteins
CD166_HUMAN	hypothetical protein LOC100133690; activated leukocyte cell adhesion molecule	C2-set_2,V-set,Ig_2	alpha-helical transmembrane proteins
IGDC4_HUMAN	immunoglobulin superfamily, DCC subclass, member 4	I-set,fn3,Ig_2	alpha-helical transmembrane proteins
IGS11_HUMAN	immunoglobulin superfamily, member 11	V-set,Ig_3	alpha-helical transmembrane proteins
IGSF2_HUMAN	immunoglobulin superfamily, member 2	I-set,V-set	alpha-helical transmembrane proteins
IGSF6_HUMAN	immunoglobulin superfamily, member 6	V-set	alpha-helical transmembrane proteins
TUTLA_HUMAN	immunoglobulin superfamily, member 9	I-set,fn3,Ig_2	alpha-helical transmembrane proteins
TUTLB_HUMAN	immunoglobulin superfamily, member 9B	V-set,I-set,fn3,Ig_2	alpha-helical transmembrane proteins
ITPR3_HUMAN	inositol 1,4,5-triphosphate receptor, type 3	RIH_assoc,RYDR_ITPR,MIR,Ins145_P3_rec	alpha-helical transmembrane proteins
INSR_HUMAN	insulin receptor	Pkinase_Tyr,fn3,Furin-like,Recep_L_domain	alpha-helical transmembrane proteins

	INSRR_HUMAN	insulin receptor-related receptor	Pkinase_Tyr,Furin-like,fn3,Recep_L_domain	alpha-helical transmembrane proteins
	IGF1R_HUMAN	insulin-like growth factor 1 receptor	Pkinase_Tyr,Furin-like,fn3,Recep_L_domain	alpha-helical transmembrane proteins
	MPRI_HUMAN	insulin-like growth factor 2 receptor	CIMR,fn2	alpha-helical transmembrane proteins
	ITM2A_HUMAN	integral membrane protein 2A	BRICHOS	alpha-helical transmembrane proteins
	ITA1_HUMAN	integrin, alpha 1	FG-GAP,Integrin_alpha2,VWA	alpha-helical transmembrane proteins
	ITA10_HUMAN	integrin, alpha 10	Integrin_alpha2,VWA	alpha-helical transmembrane proteins
	ITA11_HUMAN	integrin, alpha 11	FG-GAP,Integrin_alpha2,VWA	alpha-helical transmembrane proteins
	ITA2_HUMAN	integrin, alpha 2 (CD49B, alpha 2 subunit of VLA-2 receptor)	FG-GAP,Integrin_alpha2,VWA	alpha-helical transmembrane proteins
	ITA2B_HUMAN	integrin, alpha 2b (platelet glycoprotein IIb of IIb/IIIa complex, antigen CD41)	FG-GAP,Integrin_alpha2,Integrin_alpha	alpha-helical transmembrane proteins
	ITA3_HUMAN	integrin, alpha 3 (antigen CD49C, alpha 3 subunit of VLA-3 receptor)	Integrin_alpha2,VCBS	alpha-helical transmembrane proteins
	ITA4_HUMAN	integrin, alpha 4 (antigen CD49D, alpha 4 subunit of VLA-4 receptor)	VCBS,Integrin_alpha2	alpha-helical transmembrane proteins
	ITA5_HUMAN	integrin, alpha 5 (fibronectin receptor, alpha polypeptide)	FG-GAP,Integrin_alpha2	alpha-helical transmembrane proteins
	ITA6_HUMAN	integrin, alpha 6	VCBS,Integrin_alpha2	alpha-helical transmembrane proteins
	ITA7_HUMAN	integrin, alpha 7	FG-GAP,Integrin_alpha2	alpha-helical transmembrane proteins
	ITA8_HUMAN	integrin, alpha 8	FG-GAP,Integrin_alpha2,Integrin_alpha	alpha-helical transmembrane proteins
	ITA9_HUMAN	integrin, alpha 9	Integrin_alpha2,VCBS	alpha-helical transmembrane proteins
	ITAD_HUMAN	integrin, alpha D	Integrin_alpha2,VWA	alpha-helical transmembrane proteins
	ITAE_HUMAN	integrin, alpha E (antigen CD103, human mucosal lymphocyte antigen 1; alpha polypeptide)	Integrin_alpha2,VWA	alpha-helical transmembrane proteins
	ITAL_HUMAN	integrin, alpha L (antigen CD11A (p180), lymphocyte function-associated antigen 1; alpha polypeptide)	FG-GAP,Integrin_alpha2,VWA	alpha-helical transmembrane proteins
	ITAM_HUMAN	integrin, alpha M (complement component 3 receptor 3 subunit)	FG-GAP,Integrin_alpha2,VWA,Integrin_alpha	alpha-helical transmembrane proteins
	ITAV_HUMAN	integrin, alpha V (vitronectin receptor, alpha polypeptide, antigen CD51)	FG-GAP,Integrin_alpha2,Integrin_alpha	alpha-helical transmembrane proteins
	ITAX_HUMAN	integrin, alpha X (complement component 3 receptor 4 subunit)	FG-GAP,Integrin_alpha2,VWA,Integrin_alpha	alpha-helical transmembrane proteins
	ITB1_HUMAN	integrin, beta 1 (fibronectin receptor, beta polypeptide, antigen CD29 includes MDF2, MSK12)	EGF_2,Integrin_B_tail,Integrin_beta	alpha-helical transmembrane proteins
	ITB2_HUMAN	integrin, beta 2 (complement component 3 receptor 3 and 4 subunit)	Integrin_B_tail,Integrin_beta	alpha-helical transmembrane proteins
	ITB3_HUMAN	integrin, beta 3 (platelet glycoprotein IIIa, antigen CD61)	Integrin_B_tail,Integrin_beta	alpha-helical transmembrane proteins
	ITB5_HUMAN	integrin, beta 5	Integrin_B_tail,Integrin_beta	alpha-helical transmembrane proteins
	ITB6_HUMAN	integrin, beta 6	EGF_2,Integrin_B_tail,Integrin_beta	alpha-helical transmembrane proteins
	ITB7_HUMAN	integrin, beta 7	EGF_2,Integrin_beta	alpha-helical transmembrane proteins
	ITB8_HUMAN	integrin, beta 8	EGF_2,Integrin_beta	alpha-helical transmembrane proteins
	ICAM1_HUMAN	intercellular adhesion molecule 1	Ig_2,ICAM_N	alpha-helical transmembrane proteins
	ICAM2_HUMAN	intercellular adhesion molecule 2	ICAM_N	alpha-helical transmembrane proteins
	ICAM3_HUMAN	intercellular adhesion molecule 3	Ig_3,ICAM_N	alpha-helical transmembrane proteins
	ICAM4_HUMAN	intercellular adhesion molecule 4 (Landsteiner-Wiener blood group)	ICAM_N	alpha-helical transmembrane proteins
	ICAM5_HUMAN	intercellular adhesion molecule 5, telencephalin	ICAM_N,Ig_2	alpha-helical transmembrane proteins
	INAR1_HUMAN	interferon (alpha, beta and omega) receptor 1	Tissue_fac,Interfer-bind	alpha-helical transmembrane proteins
	INAR2_HUMAN	interferon (alpha, beta and omega) receptor 2	Tissue_fac,Interfer-bind	alpha-helical transmembrane proteins
	INGR1_HUMAN	interferon gamma receptor 1	Tissue_fac,IFNGR1	alpha-helical transmembrane proteins
	INGR2_HUMAN	interferon gamma receptor 2 (interferon gamma transducer 1)	Tissue_fac,Interfer-bind	alpha-helical transmembrane proteins
	IFM1_HUMAN	interferon induced transmembrane protein 1 (9-27)	Pyr_redox_2,Pyr_redox,CD225	alpha-helical transmembrane proteins
	IFM3_HUMAN	interferon induced transmembrane protein 3 (1-8U)	Pyr_redox_2,Pyr_redox,CD225	alpha-helical transmembrane proteins
	IL1AP_HUMAN	interleukin 1 receptor accessory protein	TIR	alpha-helical transmembrane proteins
	IL1R1_HUMAN	interleukin 1 receptor, type I	I-set,Ig_3,TIR,Ig_2	alpha-helical transmembrane proteins
	ILRL1_HUMAN	interleukin 1 receptor-like 1	I-set,TIR	alpha-helical transmembrane proteins
	ILRL2_HUMAN	interleukin 1 receptor-like 2	TIR,Ig_2	alpha-helical transmembrane proteins
	I10R1_HUMAN	interleukin 10 receptor, alpha	Tissue_fac	alpha-helical transmembrane proteins
	I10R2_HUMAN	interleukin 10 receptor, beta	Tissue_fac,Interfer-bind	alpha-helical transmembrane proteins
	I12R1_HUMAN	interleukin 12 receptor, beta 1	fn3	alpha-helical transmembrane proteins
	I12R2_HUMAN	interleukin 12 receptor, beta 2	IL6Ra-bind,fn3,Lep_receptor_Ig	alpha-helical transmembrane proteins
	Q9UDY5_HUMAN	interleukin 13 receptor, alpha 1	IL6Ra-bind	N/A
	I13R1_HUMAN	interleukin 13 receptor, alpha 1	IL6Ra-bind	alpha-helical transmembrane proteins
	I17RA_HUMAN	interleukin 17 receptor A	SEFIR	alpha-helical transmembrane proteins
	I17RB_HUMAN	interleukin 17 receptor B	SEFIR	alpha-helical transmembrane proteins
	I17RC_HUMAN	interleukin 17 receptor C	SEFIR	alpha-helical transmembrane proteins
	IL18R_HUMAN	interleukin 18 receptor 1	TIR	alpha-helical transmembrane proteins
	Q5W006_HUMAN	interleukin 2 receptor, alpha	Sushi	N/A
	IL2RA_HUMAN	interleukin 2 receptor, alpha	Sushi	alpha-helical transmembrane proteins
	IL2RG_HUMAN	interleukin 2 receptor, gamma (severe combined immunodeficiency)	IL6Ra-bind,fn3	alpha-helical transmembrane proteins
	I27RA_HUMAN	interleukin 27 receptor, alpha	fn3	alpha-helical transmembrane proteins
	IL31R_HUMAN	interleukin 31 receptor A	IL6Ra-bind,fn3	alpha-helical transmembrane proteins
	IL5RA_HUMAN	interleukin 5 receptor, alpha	IL6Ra-bind	alpha-helical transmembrane proteins
	IL6RA_HUMAN	interleukin 6 receptor	IL6Ra-bind	alpha-helical transmembrane proteins
	IL6RB_HUMAN	interleukin 6 signal transducer (gp130, oncostatin M receptor)	IL6Ra-bind,fn3,Lep_receptor_Ig	alpha-helical transmembrane proteins
	IL7RA_HUMAN	interleukin 7 receptor	fn3	alpha-helical transmembrane proteins
	JAG1_HUMAN	jagged 1 (Alagille syndrome)	EGF,MNNL	alpha-helical transmembrane proteins
	JAG2_HUMAN	jagged 2	EGF_CA,EGF,MNNL	alpha-helical transmembrane proteins
	JAM2_HUMAN	junctional adhesion molecule 2	I-set,V-set	alpha-helical transmembrane proteins
	JAM3_HUMAN	junctional adhesion molecule 3	V-set,Ig_2	alpha-helical transmembrane proteins
	JPH1_HUMAN	junctophilin 1	MORN	alpha-helical transmembrane proteins
	JPH2_HUMAN	junctophilin 2	MORN	alpha-helical transmembrane proteins
	JPH3_HUMAN	junctophilin 3	MORN	alpha-helical transmembrane proteins
	KI3L2_HUMAN	killer cell immunoglobulin-like receptor, three domains, long cytoplasmic tail, 2; similar to killer cell immunoglobulin-like receptor 3DL2 precursor (MHC class I NK cell receptor) (Natural killer-associated transcript 4) (NKAT-4) (p70 natural killer cell receptor clone CL-5) (CD158k antigen)	Ig_3,Ig_2	alpha-helical transmembrane proteins
	KI3L3_HUMAN	killer cell immunoglobulin-like receptor, three domains, long cytoplasmic tail, 3	Ig_3,ig,Ig_2	alpha-helical transmembrane proteins
	KI3L1_HUMAN	killer cell immunoglobulin-like receptor, three domains, short cytoplasmic tail, 1; killer cell immunoglobulin-like receptor, three domains, long cytoplasmic tail, 1	ig,Ig_3	alpha-helical transmembrane proteins
	KI3S1_HUMAN	killer cell immunoglobulin-like receptor, three domains, short cytoplasmic tail, 1; killer cell immunoglobulin-like receptor, three domains, long cytoplasmic tail, 1	ig,Ig_3	alpha-helical transmembrane proteins
	Q6IST4_HUMAN	killer cell immunoglobulin-like receptor, two domains, long cytoplasmic tail, 2; killer cell immunoglobulin-like receptor, two domains, long cytoplasmic tail, 1; killer-cell Ig-like receptor; killer cell immunoglobulin-like receptor, three domains, pseudogene 1	ig	N/A
	KI2L1_HUMAN	killer cell immunoglobulin-like receptor, two domains, long cytoplasmic tail, 2; killer cell immunoglobulin-like receptor, two domains, long cytoplasmic tail, 1; killer-cell Ig-like receptor; killer cell immunoglobulin-like receptor, three domains, pseudogene 1	ig	alpha-helical transmembrane proteins
	KI2L2_HUMAN	killer cell immunoglobulin-like receptor, two domains, long cytoplasmic tail, 2; killer cell immunoglobulin-like receptor, two domains, long cytoplasmic tail, 1; killer-cell Ig-like receptor; killer cell immunoglobulin-like receptor, three domains, pseudogene 1	Ig_3	alpha-helical transmembrane proteins
	KI2L3_HUMAN	killer cell immunoglobulin-like receptor, two domains, long cytoplasmic tail, 3	ig,Ig_3	alpha-helical transmembrane proteins
	KI2L4_HUMAN	killer cell immunoglobulin-like receptor, two domains, long cytoplasmic tail, 4	ig,Ig_2	alpha-helical transmembrane proteins
	KI2LB_HUMAN	killer cell immunoglobulin-like receptor, two domains, long cytoplasmic tail, 5B; killer cell immunoglobulin-like receptor, two domains, long cytoplasmic tail, 5A	ig,Ig_2	alpha-helical transmembrane proteins
	KI2LA_HUMAN	killer cell immunoglobulin-like receptor, two domains, long cytoplasmic tail, 5B; killer cell immunoglobulin-like receptor, two domains, long cytoplasmic tail, 5A	ig,Ig_2	alpha-helical transmembrane proteins
	KI2S1_HUMAN	killer cell immunoglobulin-like receptor, two domains, short cytoplasmic tail, 1; killer cell immunoglobulin-like receptor, two domains, short cytoplasmic tail, 3; killer cell immunoglobulin-like receptor, two domains, short cytoplasmic tail, 5	ig	alpha-helical transmembrane proteins
	KI2S5_HUMAN	killer cell immunoglobulin-like receptor, two domains, short cytoplasmic tail, 1; killer cell immunoglobulin-like receptor, two domains, short cytoplasmic tail, 3; killer cell immunoglobulin-like receptor, two domains, short cytoplasmic tail, 5	Ig_3,ig	alpha-helical transmembrane proteins
plasma membrane	KI2S3_HUMAN	killer cell immunoglobulin-like receptor, two domains, short cytoplasmic tail, 1; killer cell immunoglobulin-like receptor, two domains, short cytoplasmic tail, 3; killer cell immunoglobulin-like receptor, two domains, short cytoplasmic tail, 5	Ig_3	alpha-helical transmembrane proteins
	KI2S2_HUMAN	killer cell immunoglobulin-like receptor, two domains, short cytoplasmic tail, 2; killer cell immunoglobulin-like receptor, two domains, short cytoplasmic tail, 4	ig,Ig_3	alpha-helical transmembrane proteins
	KI2S4_HUMAN	killer cell immunoglobulin-like receptor, two domains, short cytoplasmic tail, 2; killer cell immunoglobulin-like receptor, two domains, short cytoplasmic tail, 4	ig	alpha-helical transmembrane proteins
	KLRB1_HUMAN	killer cell lectin-like receptor subfamily B, member 1	Lectin_C	alpha-helical transmembrane proteins
	NKG2A_HUMAN	killer cell lectin-like receptor subfamily C, member 1	Lectin_C	alpha-helical transmembrane proteins
	NKG2C_HUMAN	killer cell lectin-like receptor subfamily C, member 2	Lectin_C	alpha-helical transmembrane proteins
	KLRD1_HUMAN	killer cell lectin-like receptor subfamily D, member 1	Lectin_C	alpha-helical transmembrane proteins
	KLRF1_HUMAN	killer cell lectin-like receptor subfamily F, member 1	Lectin_C	alpha-helical transmembrane proteins
	NKG2D_HUMAN	killer cell lectin-like receptor subfamily K, member 1	Lectin_C	alpha-helical transmembrane proteins
	KIRR1_HUMAN	kin of IRRE like (Drosophila)	C2-set_2,Ig_2,Ig_3,I-set	alpha-helical transmembrane proteins
	Q5W0F9_HUMAN	kin of IRRE like (Drosophila)	Ig_3,Ig_2	N/A
	Q5W0G0_HUMAN	kin of IRRE like (Drosophila)	Ig_3,Ig_2	N/A
	KIRR2_HUMAN	kin of IRRE like 2 (Drosophila)	I-set,C2-set_2,Ig_2	alpha-helical transmembrane proteins
	KIRR3_HUMAN	kin of IRRE like 3 (Drosophila)	C2-set_2,Ig_2,I-set	alpha-helical transmembrane proteins
	VGFR2_HUMAN	kinase insert domain receptor (a type III receptor tyrosine kinase)	Pkinase_Tyr,I-set,V-set,Ig_2	alpha-helical transmembrane proteins
	KLOT_HUMAN	klotho	Glyco_hydro_1	alpha-helical transmembrane proteins
	KLOTB_HUMAN	klotho beta	Glyco_hydro_1	alpha-helical transmembrane proteins

LPH_HUMAN	lactase	Glyco_hydro_1	alpha-helical transmembrane proteins
LPHN1_HUMAN	latrophilin 1	HRM,Gal_Lectin,DUF3497,OLF,Latrophilin	alpha-helical transmembrane proteins
LPHN2_HUMAN	latrophilin 2	Gal_Lectin,HRM,DUF3497,OLF,Latrophilin	alpha-helical transmembrane proteins
LPHN3_HUMAN	latrophilin 3	Gal_Lectin,HRM,DUF3497,OLF,Latrophilin	alpha-helical transmembrane proteins
LAYN_HUMAN	layilin	Lectin_C	alpha-helical transmembrane proteins
LEPR_HUMAN	leptin receptor	Lep_receptor_Ig	alpha-helical transmembrane proteins
LIGO1_HUMAN	leucine rich repeat and Ig domain containing 1	LRR_8,I-set	alpha-helical transmembrane proteins
LRFN1_HUMAN	leucine rich repeat and fibronectin type III domain containing 1	LRR_8,I-set,fn3	alpha-helical transmembrane proteins
LRC32_HUMAN	leucine rich repeat containing 32	LRR_8,LRRNT	alpha-helical transmembrane proteins
LGR4_HUMAN	leucine-rich repeat-containing G protein-coupled receptor 4	LRR_8,LRRNT	alpha-helical transmembrane proteins
LGR5_HUMAN	leucine-rich repeat-containing G protein-coupled receptor 5	LRR_8,LRRNT,LRR_5	alpha-helical transmembrane proteins
LGR6_HUMAN	leucine-rich repeat-containing G protein-coupled receptor 6	LRR_8	alpha-helical transmembrane proteins
LRIG2_HUMAN	leucine-rich repeats and immunoglobulin-like domains 2	LRR_8,I-set	alpha-helical transmembrane proteins
LCAP_HUMAN	leucyl/cystinyl aminopeptidase	Peptidase_M1,DUF3358	alpha-helical transmembrane proteins
LIFR_HUMAN	leukemia inhibitory factor receptor alpha	fn3	alpha-helical transmembrane proteins
LIRB1_HUMAN	leukocyte immunoglobulin-like receptor, subfamily B (with TM and ITIM domains), member 1	Ig_3,Ig_2	alpha-helical transmembrane proteins
LIRB2_HUMAN	leukocyte immunoglobulin-like receptor, subfamily B (with TM and ITIM domains), member 2	Ig_3,Ig_2	alpha-helical transmembrane proteins
LIRB4_HUMAN	leukocyte immunoglobulin-like receptor, subfamily B (with TM and ITIM domains), member 4	Ig_3,Ig_2	alpha-helical transmembrane proteins
LTK_HUMAN	leukocyte receptor tyrosine kinase	Pkinase_Tyr,SAM_1,Gly_rich	alpha-helical transmembrane proteins
LAIR1_HUMAN	leukocyte-associated immunoglobulin-like receptor 1	Ig_2	alpha-helical transmembrane proteins
LSAMP_HUMAN	limbic system-associated membrane protein	I-set	N/A
LDLR_HUMAN	low density lipoprotein receptor	EGF_CA,Ldl_recept_a,Ldl_recept_b	alpha-helical transmembrane proteins
LRP6_HUMAN	low density lipoprotein receptor-related protein 6	Ldl_recept_a,Ldl_recept_b	alpha-helical transmembrane proteins
LRP8_HUMAN	low density lipoprotein receptor-related protein 8, apolipoprotein e receptor	cEGF,Ldl_recept_a,Ldl_recept_b	alpha-helical transmembrane proteins
LRP1_HUMAN	low density lipoprotein-related protein 1 (alpha-2-macroglobulin receptor)	cEGF,Ldl_recept_a,Ldl_recept_b	alpha-helical transmembrane proteins
LRP12_HUMAN	low density lipoprotein-related protein 12	Ldl_recept_a,CUB	alpha-helical transmembrane proteins
LRP2_HUMAN	low density lipoprotein-related protein 2	cEGF,Ldl_recept_a,Ldl_recept_b	alpha-helical transmembrane proteins
LSHR_HUMAN	luteinizing hormone/choriogonadotropin receptor	LRR_5	alpha-helical transmembrane proteins
LYVE1_HUMAN	lymphatic vessel endothelial hyaluronan receptor 1	Xlink	alpha-helical transmembrane proteins
LY6D_HUMAN	lymphocyte antigen 6 complex, locus D	UPAR_LY6	N/A
LY66F_HUMAN	lymphocyte antigen 6 complex, locus G6F; lymphocyte antigen 6 complex, locus G6D	V-set	alpha-helical transmembrane proteins
LRMP_HUMAN	lymphoid-restricted membrane protein	MRVI1	alpha-helical transmembrane proteins
LAMP1_HUMAN	lysosomal-associated membrane protein 1	Lamp	alpha-helical transmembrane proteins
LAMP2_HUMAN	lysosomal-associated membrane protein 2	Lamp	alpha-helical transmembrane proteins
MARCO_HUMAN	macrophage receptor with collagenous structure	SRCR,Collagen	alpha-helical transmembrane proteins
MSRE_HUMAN	macrophage scavenger receptor 1	SRCR,Collagen	alpha-helical transmembrane proteins
RON_HUMAN	macrophage stimulating 1 receptor (c-met-related tyrosine kinase)	Pkinase_Tyr,TIG,Sema	alpha-helical transmembrane proteins
Q5SRN5_HUMAN	major histocompatibility complex, class I, A	C1-set,MHC_I	N/A
1A80_HUMAN	major histocompatibility complex, class I, A	C1-set,MHC_I	alpha-helical transmembrane proteins
1A32_HUMAN	major histocompatibility complex, class I, A	C1-set,MHC_I	alpha-helical transmembrane proteins
1A26_HUMAN	major histocompatibility complex, class I, A	C1-set,MHC_I	alpha-helical transmembrane proteins
1A66_HUMAN	major histocompatibility complex, class I, A	C1-set,MHC_I	alpha-helical transmembrane proteins
1A31_HUMAN	major histocompatibility complex, class I, A	C1-set,MHC_I	alpha-helical transmembrane proteins
1A25_HUMAN	major histocompatibility complex, class I, A	C1-set,MHC_I	alpha-helical transmembrane proteins
1A68_HUMAN	major histocompatibility complex, class I, A	C1-set,MHC_I	alpha-helical transmembrane proteins
1A36_HUMAN	major histocompatibility complex, class I, A	C1-set,MHC_I	alpha-helical transmembrane proteins
1A43_HUMAN	major histocompatibility complex, class I, A	C1-set,MHC_I	alpha-helical transmembrane proteins
1A03_HUMAN	major histocompatibility complex, class I, A	C1-set,MHC_I	alpha-helical transmembrane proteins
1A34_HUMAN	major histocompatibility complex, class I, A	C1-set,MHC_I	alpha-helical transmembrane proteins
1A02_HUMAN	major histocompatibility complex, class I, A	C1-set,MHC_I	alpha-helical transmembrane proteins
1A29_HUMAN	major histocompatibility complex, class I, A	C1-set,MHC_I	alpha-helical transmembrane proteins
1A23_HUMAN	major histocompatibility complex, class I, A	C1-set,MHC_I	alpha-helical transmembrane proteins
1A24_HUMAN	major histocompatibility complex, class I, A	C1-set,MHC_I	alpha-helical transmembrane proteins
1A11_HUMAN	major histocompatibility complex, class I, A	C1-set,MHC_I	alpha-helical transmembrane proteins
1A01_HUMAN	major histocompatibility complex, class I, A	C1-set,MHC_I	alpha-helical transmembrane proteins
1A69_HUMAN	major histocompatibility complex, class I, A	C1-set,MHC_I	alpha-helical transmembrane proteins
1B18_HUMAN	major histocompatibility complex, class I, C; major histocompatibility complex, class I, B	C1-set,MHC_I	alpha-helical transmembrane proteins
1B37_HUMAN	major histocompatibility complex, class I, C; major histocompatibility complex, class I, B	C1-set,MHC_I	alpha-helical transmembrane proteins
1C06_HUMAN	major histocompatibility complex, class I, C; major histocompatibility complex, class I, B	C1-set,MHC_I	alpha-helical transmembrane proteins
1C04_HUMAN	major histocompatibility complex, class I, C; major histocompatibility complex, class I, B	C1-set,MHC_I	alpha-helical transmembrane proteins
1C12_HUMAN	major histocompatibility complex, class I, C; major histocompatibility complex, class I, B	C1-set,MHC_I,Filament	alpha-helical transmembrane proteins
1B08_HUMAN	major histocompatibility complex, class I, C; major histocompatibility complex, class I, B	C1-set,MHC_I	alpha-helical transmembrane proteins
1B51_HUMAN	major histocompatibility complex, class I, C; major histocompatibility complex, class I, B	C1-set,MHC_I	alpha-helical transmembrane proteins
1C08_HUMAN	major histocompatibility complex, class I, C; major histocompatibility complex, class I, B	C1-set,MHC_I	alpha-helical transmembrane proteins
1B55_HUMAN	major histocompatibility complex, class I, C; major histocompatibility complex, class I, B	C1-set,MHC_I	alpha-helical transmembrane proteins
1B15_HUMAN	major histocompatibility complex, class I, C; major histocompatibility complex, class I, B	C1-set,MHC_I	alpha-helical transmembrane proteins
1B44_HUMAN	major histocompatibility complex, class I, C; major histocompatibility complex, class I, B	C1-set,MHC_I	alpha-helical transmembrane proteins
1C16_HUMAN	major histocompatibility complex, class I, C; major histocompatibility complex, class I, B	C1-set,MHC_I,Filament	alpha-helical transmembrane proteins
1B58_HUMAN	major histocompatibility complex, class I, C; major histocompatibility complex, class I, B	C1-set,MHC_I	alpha-helical transmembrane proteins
1B41_HUMAN	major histocompatibility complex, class I, C; major histocompatibility complex, class I, B	C1-set,MHC_I	alpha-helical transmembrane proteins
1B35_HUMAN	major histocompatibility complex, class I, C; major histocompatibility complex, class I, B	C1-set,MHC_I	alpha-helical transmembrane proteins
1C03_HUMAN	major histocompatibility complex, class I, C; major histocompatibility complex, class I, B	C1-set,MHC_I	alpha-helical transmembrane proteins
1C01_HUMAN	major histocompatibility complex, class I, C; major histocompatibility complex, class I, B	C1-set,MHC_I	alpha-helical transmembrane proteins
1B46_HUMAN	major histocompatibility complex, class I, C; major histocompatibility complex, class I, B	C1-set,MHC_I	alpha-helical transmembrane proteins
1B39_HUMAN	major histocompatibility complex, class I, C; major histocompatibility complex, class I, B	C1-set,MHC_I	alpha-helical transmembrane proteins
1C17_HUMAN	major histocompatibility complex, class I, C; major histocompatibility complex, class I, B	C1-set,MHC_I,Filament	alpha-helical transmembrane proteins
1B53_HUMAN	major histocompatibility complex, class I, C; major histocompatibility complex, class I, B	C1-set,MHC_I	alpha-helical transmembrane proteins
1C02_HUMAN	major histocompatibility complex, class I, C; major histocompatibility complex, class I, B	C1-set,MHC_I	alpha-helical transmembrane proteins
1C07_HUMAN	major histocompatibility complex, class I, C; major histocompatibility complex, class I, B	C1-set,MHC_I	alpha-helical transmembrane proteins
1B78_HUMAN	major histocompatibility complex, class I, C; major histocompatibility complex, class I, B	C1-set,MHC_I	alpha-helical transmembrane proteins
1C05_HUMAN	major histocompatibility complex, class I, C; major histocompatibility complex, class I, B	C1-set,MHC_I	alpha-helical transmembrane proteins
1B52_HUMAN	major histocompatibility complex, class I, C; major histocompatibility complex, class I, B	C1-set,MHC_I	alpha-helical transmembrane proteins
1B49_HUMAN	major histocompatibility complex, class I, C; major histocompatibility complex, class I, B	C1-set,MHC_I	alpha-helical transmembrane proteins
1C14_HUMAN	major histocompatibility complex, class I, C; major histocompatibility complex, class I, B	C1-set,MHC_I,Filament	alpha-helical transmembrane proteins
1B40_HUMAN	major histocompatibility complex, class I, C; major histocompatibility complex, class I, B	C1-set,MHC_I	alpha-helical transmembrane proteins
1B38_HUMAN	major histocompatibility complex, class I, C; major histocompatibility complex, class I, B	C1-set,MHC_I	alpha-helical transmembrane proteins
1B42_HUMAN	major histocompatibility complex, class I, C; major histocompatibility complex, class I, B	C1-set,MHC_I	alpha-helical transmembrane proteins
1B14_HUMAN	major histocompatibility complex, class I, C; major histocompatibility complex, class I, B	C1-set,MHC_I	alpha-helical transmembrane proteins
1B27_HUMAN	major histocompatibility complex, class I, C; major histocompatibility complex, class I, B	C1-set,MHC_I	alpha-helical transmembrane proteins
1B07_HUMAN	major histocompatibility complex, class I, C; major histocompatibility complex, class I, B	C1-set,MHC_I	alpha-helical transmembrane proteins
1B54_HUMAN	major histocompatibility complex, class I, C; major histocompatibility complex, class I, B	C1-set,MHC_I	alpha-helical transmembrane proteins
1B13_HUMAN	major histocompatibility complex, class I, C; major histocompatibility complex, class I, B	C1-set,MHC_I	alpha-helical transmembrane proteins
1B73_HUMAN	major histocompatibility complex, class I, C; major histocompatibility complex, class I, B	C1-set,MHC_I	alpha-helical transmembrane proteins
1B57_HUMAN	major histocompatibility complex, class I, C; major histocompatibility complex, class I, B	C1-set,MHC_I	alpha-helical transmembrane proteins
1B81_HUMAN	major histocompatibility complex, class I, C; major histocompatibility complex, class I, B	C1-set,MHC_I	alpha-helical transmembrane proteins
Q6DU44_HUMAN	major histocompatibility complex, class I, E	C1-set,MHC_I	N/A
HLAE_HUMAN	major histocompatibility complex, class I, E	C1-set,MHC_I	alpha-helical transmembrane proteins
HLAF_HUMAN	major histocompatibility complex, class I, F	C1-set,MHC_I	alpha-helical transmembrane proteins
HLAG_HUMAN	major histocompatibility complex, class I, G	C1-set,MHC_I	alpha-helical transmembrane proteins
Q31611_HUMAN	major histocompatibility complex, class I, G	C1-set,MHC_I	N/A
Q29897_HUMAN	major histocompatibility complex, class I, G	MHC_I	N/A
HLAH_HUMAN	major histocompatibility complex, class I, H (pseudogene)	C1-set,MHC_I	alpha-helical transmembrane proteins
HMR1_HUMAN	major histocompatibility complex, class I-related	C1-set,MHC_I	alpha-helical transmembrane proteins
DPA1_HUMAN	major histocompatibility complex, class II, DP alpha 1	C1-set,MHC_II_alpha	alpha-helical transmembrane proteins
DPB1_HUMAN	major histocompatibility complex, class II, DP beta 1	C1-set,MHC_II_beta	alpha-helical transmembrane proteins
Q5Y7D6_HUMAN	major histocompatibility complex, class II, DQ beta 1; similar to major histocompatibility complex, class II, DQ beta 1	C1-set,MHC_II_beta	N/A
Q30118_HUMAN	major histocompatibility complex, class II, DR alpha	C1-set,MHC_II_alpha	N/A
DRB3_HUMAN	major histocompatibility complex, class II, DR beta 3	C1-set,MHC_II_beta	alpha-helical transmembrane proteins
2B17_HUMAN	major histocompatibility complex, class II, DR beta 3	C1-set,MHC_II_beta	alpha-helical transmembrane proteins
2B11_HUMAN	major histocompatibility complex, class II, DR beta 4; major histocompatibility complex, class II, DR beta 1	C1-set,MHC_II_beta	alpha-helical transmembrane proteins
2B1A_HUMAN	major histocompatibility complex, class II, DR beta 4; major histocompatibility complex, class II, DR beta 1	C1-set,MHC_II_beta	alpha-helical transmembrane proteins
2B14_HUMAN	major histocompatibility complex, class II, DR beta 4; major histocompatibility complex, class II, DR beta 1	C1-set,MHC_II_beta	alpha-helical transmembrane proteins
2B18_HUMAN	major histocompatibility complex, class II, DR beta 4; major histocompatibility complex, class II, DR beta 1	C1-set,MHC_II_beta	alpha-helical transmembrane proteins

DRB4_HUMAN	major histocompatibility complex, class II, DR beta 4; major histocompatibility complex, class II, DR beta 1	C1-set,MHC_II_beta	alpha-helical transmembrane proteins
2B1F_HUMAN	major histocompatibility complex, class II, DR beta 4; major histocompatibility complex, class II, DR beta 1	C1-set,MHC_II_beta	alpha-helical transmembrane proteins
2B1B_HUMAN	major histocompatibility complex, class II, DR beta 4; major histocompatibility complex, class II, DR beta 1	C1-set,MHC_II_beta	alpha-helical transmembrane proteins
DRB5_HUMAN	major histocompatibility complex, class II, DR beta 5	C1-set,MHC_II_beta	alpha-helical transmembrane proteins
2B19_HUMAN	major histocompatibility complex, class II, DR beta 5	C1-set,MHC_II_beta	alpha-helical transmembrane proteins
MIP_HUMAN	major intrinsic protein of lens fiber	MIP,RhoGAP,G8	alpha-helical transmembrane proteins
MGA_HUMAN	maltase-glucoamylase (alpha-glucosidase)	Glyco_hydro_31,Trefoil,Granin	alpha-helical transmembrane proteins
MRC1_HUMAN	mannose receptor, C type 1	Ricin_B_lectin,Myb_DNA-binding,fn2,Lectin_C	alpha-helical transmembrane proteins
MPRD_HUMAN	mannose-6-phosphate receptor (cation dependent)	Man-6-P_recep	alpha-helical transmembrane proteins
MMP14_HUMAN	matrix metallopeptidase 14 (membrane-inserted)	Hemopexin,PG_binding_1,Peptidase_M10	alpha-helical transmembrane proteins
MMP15_HUMAN	matrix metallopeptidase 15 (membrane-inserted)	Hemopexin,PG_binding_1,Peptidase_M10	alpha-helical transmembrane proteins
MMP16_HUMAN	matrix metallopeptidase 16 (membrane-inserted)	Hemopexin,PG_binding_1,Peptidase_M10	alpha-helical transmembrane proteins
MMP24_HUMAN	matrix metallopeptidase 24 (membrane-inserted)	Hemopexin,PG_binding_1,Peptidase_M10	alpha-helical transmembrane proteins
MUC18_HUMAN	melanoma cell adhesion molecule	C2-set_2,V-set,ig,Ig_3	alpha-helical transmembrane proteins
NEP_HUMAN	membrane metallo-endopeptidase	Peptidase_M13_N,Peptidase_M13	alpha-helical transmembrane proteins
CD20_HUMAN	membrane-spanning 4-domains, subfamily A, member 1	Cadherin,CD20,K_tetra	alpha-helical transmembrane proteins
FCERB_HUMAN	membrane-spanning 4-domains, subfamily A, member 2 (Fc fragment of IgE, high affinity I, receptor for; beta polypeptide)	CD20	alpha-helical transmembrane proteins
MEP1A_HUMAN	meprin A, alpha (PABA peptide hydrolase)	MATH,MAM,EGF,Astacin	alpha-helical transmembrane proteins
MEP1B_HUMAN	meprin A, beta	MAM,MATH,Astacin	alpha-helical transmembrane proteins
MET_HUMAN	met proto-oncogene (hepatocyte growth factor receptor)	Pkinase_Tyr,TIG,Sema,PSI	alpha-helical transmembrane proteins
MFAP3_HUMAN	microfibrillar-associated protein 3	I-set	alpha-helical transmembrane proteins
MFA3L_HUMAN	microfibrillar-associated protein 3-like	I-set	alpha-helical transmembrane proteins
AOFB_HUMAN	monoamine oxidase B	Amino_oxidase	alpha-helical transmembrane proteins
PAQRB_HUMAN	monocyte to macrophage differentiation-associated	HlyIII	alpha-helical transmembrane proteins
MSPD2_HUMAN	motile sperm domain containing 2	CRAL_TRIO,Motile_Sperm	alpha-helical transmembrane proteins
MUC1_HUMAN	mucin 1, cell surface associated	SEA	alpha-helical transmembrane proteins
MUC12_HUMAN	mucin 12, cell surface associated; similar to mucin 11	SEA	alpha-helical transmembrane proteins
MUC13_HUMAN	mucin 13, cell surface associated	SEA	alpha-helical transmembrane proteins
MUC16_HUMAN	mucin 16, cell surface associated	SEA	alpha-helical transmembrane proteins
MUC17_HUMAN	mucin 17, cell surface associated	SEA	alpha-helical transmembrane proteins
MUC3B_HUMAN	mucin 3B, cell surface associated; similar to Mucin-3A precursor (Intestinal mucin-3A); mucin 3A, cell surface associated; similar to mucin 3	SEA	alpha-helical transmembrane proteins
MUC3A_HUMAN	mucin 3B, cell surface associated; similar to Mucin-3A precursor (Intestinal mucin-3A); mucin 3A, cell surface associated; similar to mucin 3	SEA	alpha-helical transmembrane proteins
MUC4_HUMAN	mucin 4, cell surface associated	VWD,AMOP,NIDO	alpha-helical transmembrane proteins
MCLN1_HUMAN	mucolipin 1	PKD_channel	alpha-helical transmembrane proteins
MCLN3_HUMAN	mucolipin 3	PKD_channel	alpha-helical transmembrane proteins
MUSK_HUMAN	muscle, skeletal, receptor tyrosine kinase	Pkinase_Tyr,Fz,I-set,Ig_2	alpha-helical transmembrane proteins
MAG_HUMAN	myelin associated glycoprotein	C2-set_2,I-set,Ig_2	alpha-helical transmembrane proteins
MOG_HUMAN	myelin oligodendrocyte glycoprotein	V-set	alpha-helical transmembrane proteins
Q5SUK5_HUMAN	myelin oligodendrocyte glycoprotein	V-set	N/A
MYP0_HUMAN	myelin protein zero	Myelin-PO_C,V-set	alpha-helical transmembrane proteins
MPZL1_HUMAN	myelin protein zero-like 1	V-set	alpha-helical transmembrane proteins
Q9UEL6_HUMAN	myelin protein zero-like 1	V-set	N/A
MLF2_HUMAN	myeloid leukemia factor 2	Mlf1IP	N/A
TPOR_HUMAN	myeloproliferative leukemia virus oncogene	EpoR_lig-bind,fn3	alpha-helical transmembrane proteins
Q5JUY5_HUMAN	myeloproliferative leukemia virus oncogene	EpoR_lig-bind,fn3	N/A
MYOF_HUMAN	myoferlin	FerA,C2,FerB	alpha-helical transmembrane proteins
NCTR1_HUMAN	natural cytotoxicity triggering receptor 1	Ig_3,Ig_2	alpha-helical transmembrane proteins
NCTR2_HUMAN	natural cytotoxicity triggering receptor 2	V-set	alpha-helical transmembrane proteins
NEO1_HUMAN	neogenin homolog 1 (chicken)	I-set,fn3,Ig_2	alpha-helical transmembrane proteins
Q59FP8_HUMAN	neogenin homolog 1 (chicken)	I-set,fn3	N/A
NPHN_HUMAN	nephrosis 1, congenital, Finnish type (nephrin)	V-set,C2-set_2,fn3,Ig_2	alpha-helical transmembrane proteins
PODO_HUMAN	nephrosis 2, idiopathic, steroid-resistant (podocin)	Band_7	N/A
TNR16_HUMAN	nerve growth factor receptor (TNFR superfamily, member 16)	TNFR_c6,Death	alpha-helical transmembrane proteins
NCAM1_HUMAN	neural cell adhesion molecule 1	I-set,fn3	alpha-helical transmembrane proteins
NCAM2_HUMAN	neural cell adhesion molecule 2	I-set,fn3	alpha-helical transmembrane proteins
NRG1_HUMAN	neuregulin 1	I-set	alpha-helical transmembrane proteins
NRG2_HUMAN	neuregulin 2	I-set	alpha-helical transmembrane proteins
NRX1B_HUMAN	neurexin 1	Laminin_G_2	alpha-helical transmembrane proteins
NRX1A_HUMAN	neurexin 1	Laminin_G_2	alpha-helical transmembrane proteins
NRX3B_HUMAN	neurexin 3	Laminin_G_2	alpha-helical transmembrane proteins
NRX3A_HUMAN	neurexin 3	Laminin_G_2	alpha-helical transmembrane proteins
NFASC_HUMAN	neurofascin homolog (chicken)	I-set,fn3,Bravo_FIGEY,Ig_2	alpha-helical transmembrane proteins
NLGN1_HUMAN	neuroligin 1	COesterase	alpha-helical transmembrane proteins
NLGNX_HUMAN	neuroligin 4, X-linked	COesterase	alpha-helical transmembrane proteins
NRCAM_HUMAN	neuronal cell adhesion molecule	I-set,fn3,Bravo_FIGEY,Ig_2	alpha-helical transmembrane proteins
NPTXR_HUMAN	neuronal pentraxin receptor	Pentaxin	alpha-helical transmembrane proteins
NETO1_HUMAN	neuropilin (NRP) and tolloid (TLL)-like 1	Ldl_recept_a,CUB	alpha-helical transmembrane proteins
Q5JWQ6_HUMAN	neuropilin 1	F5_F8_type_C,MAM,CUB	N/A
NRP1_HUMAN	neuropilin 1	F5_F8_type_C,MAM,CUB	alpha-helical transmembrane proteins
NRP2_HUMAN	neuropilin 2	F5_F8_type_C,MAM,CUB	alpha-helical transmembrane proteins
NPTN_HUMAN	neuroplastin	I-set,Ig_2	alpha-helical transmembrane proteins
NTRK1_HUMAN	neurotrophic tyrosine kinase, receptor, type 1	Pkinase_Tyr,LRR_8	alpha-helical transmembrane proteins
NTRK2_HUMAN	neurotrophic tyrosine kinase, receptor, type 2	LRR_8,Pkinase_Tyr,LRRNT,I-set	alpha-helical transmembrane proteins
NTRK3_HUMAN	neurotrophic tyrosine kinase, receptor, type 3	Pkinase_Tyr,LRR_8,LRRNT,I-set	alpha-helical transmembrane proteins
NICA_HUMAN	nicastrin	Nicastrin	alpha-helical transmembrane proteins
NUCB2_HUMAN	nucleobindin 2	EF_hand_5	N/A
OCLN_HUMAN	occludin pseudogene; occludin	Occludin_ELL	alpha-helical transmembrane proteins
TEN1_HUMAN	odz, odd Oz/ten-m homolog 1(Drosophila)	Ten_N	alpha-helical transmembrane proteins
OMGP_HUMAN	oligodendrocyte myelin glycoprotein	LRR_8,LRRNT,LRR_4	N/A
OSMR_HUMAN	oncostatin M receptor	fn3	alpha-helical transmembrane proteins
OTOF_HUMAN	otoferlin	C2,FerB	alpha-helical transmembrane proteins
OLR1_HUMAN	oxidized low density lipoprotein (lectin-like) receptor 1	Folate_rec,Lectin_C	alpha-helical transmembrane proteins
PILRA_HUMAN	paired immunoglobin-like type 2 receptor alpha	V-set	alpha-helical transmembrane proteins
PILRB_HUMAN	paired immunoglobin-like type 2 receptor beta	V-set	alpha-helical transmembrane proteins
PKHD1_HUMAN	par-3 partitioning defective 3 homolog (C. elegans)	TIG,Beta_helix,G8	alpha-helical transmembrane proteins
PTH1R_HUMAN	parathyroid hormone 1 receptor	HRM	alpha-helical transmembrane proteins
PTH2R_HUMAN	parathyroid hormone 2 receptor	HRM	alpha-helical transmembrane proteins
PLPL2_HUMAN	patatin-like phospholipase domain containing 2	Patatin	alpha-helical transmembrane proteins
PLPL6_HUMAN	patatin-like phospholipase domain containing 6	cNMP_binding,Patatin	alpha-helical transmembrane proteins
PHEX_HUMAN	phosphate regulating endopeptidase homolog, X-linked	Peptidase_M13_N,Peptidase_M13	alpha-helical transmembrane proteins
STOM_HUMAN	phosphatidylethanolamine binding protein 1	Band_7	N/A
PLA2R_HUMAN	phospholipase A2 receptor 1, 180kDa	fn2,Lectin_C	alpha-helical transmembrane proteins
PLB1_HUMAN	phospholipase B1	Lipase_GDSL,Pkinase_Tyr	alpha-helical transmembrane proteins
PLVAP_HUMAN	plasmalemma vesicle associated protein	PV-1	alpha-helical transmembrane proteins
PGFRA_HUMAN	platelet-derived growth factor receptor, alpha polypeptide	Pkinase_Tyr,I-set	alpha-helical transmembrane proteins
PGFRB_HUMAN	platelet-derived growth factor receptor, beta polypeptide	Pkinase_Tyr,I-set,ig,Ig_2	alpha-helical transmembrane proteins
PECA1_HUMAN	platelet/endothelial cell adhesion molecule	Ig_2	alpha-helical transmembrane proteins
PLXA1_HUMAN	plexin A1	TIG,PSI,Sema,Plexin_cytopl	alpha-helical transmembrane proteins
PLXA2_HUMAN	plexin A2	TIG,PSI,Sema,Plexin_cytopl	alpha-helical transmembrane proteins
PLXA4_HUMAN	plexin A4	Plexin_cytopl,TIG,Sema,PSI	alpha-helical transmembrane proteins
PLXC1_HUMAN	plexin C1	Plexin_cytopl,TIG,PSI	alpha-helical transmembrane proteins
PLXD1_HUMAN	plexin D1	TIG,PSI,Sema,Plexin_cytopl	alpha-helical transmembrane proteins
PVR_HUMAN	poliovirus receptor	C2-set_2,V-set,ig	alpha-helical transmembrane proteins
PVRL1_HUMAN	poliovirus receptor-related 1 (herpesvirus entry mediator C)	C2-set_2,V-set,Ig_2	alpha-helical transmembrane proteins
PVRL2_HUMAN	poliovirus receptor-related 2 (herpesvirus entry mediator B)	C2-set_2,V-set	alpha-helical transmembrane proteins
PVRL3_HUMAN	poliovirus receptor-related 3	C2-set_2,V-set	alpha-helical transmembrane proteins
PVRL4_HUMAN	poliovirus receptor-related 4	C2-set_2,V-set,ig	alpha-helical transmembrane proteins
PKHD1_HUMAN	polycystic kidney and hepatic disease 1 (autosomal recessive)	TIG,Beta_helix,G8	alpha-helical transmembrane proteins
PKD1_HUMAN	polycystic kidney disease 1 (autosomal dominant)	REJ,WSC,Lectin_C,LRR_8,PKD_channel,PKD,PLAT	alpha-helical transmembrane proteins
PKD2_HUMAN	polycystic kidney disease 2 (autosomal dominant)	PKD_channel	alpha-helical transmembrane proteins
PK2L1_HUMAN	polycystic kidney disease 2-like 1	PKD_channel	alpha-helical transmembrane proteins

PIGR_HUMAN	polymeric immunoglobulin receptor	V-set	alpha-helical transmembrane proteins
TRI13_HUMAN	potassium channel regulator	zf-B_box,zf-C3HC4_2	alpha-helical transmembrane proteins
KCNT1_HUMAN	potassium channel, subfamily T, member 1	BK_channel_a	alpha-helical transmembrane proteins
KCNT2_HUMAN	potassium channel, subfamily T, member 2	BK_channel_a	alpha-helical transmembrane proteins
KCNV1_HUMAN	potassium channel, subfamily V, member 1	K_tetra	alpha-helical transmembrane proteins
KCNV2_HUMAN	potassium channel, subfamily V, member 2	K_tetra	alpha-helical transmembrane proteins
KCNN1_HUMAN	potassium intermediate/small conductance calcium-activated channel, subfamily N, member 1	CaMBD	alpha-helical transmembrane proteins
KCNN3_HUMAN	potassium intermediate/small conductance calcium-activated channel, subfamily N, member 3	CaMBD	alpha-helical transmembrane proteins
KCNN4_HUMAN	potassium intermediate/small conductance calcium-activated channel, subfamily N, member 4	CaMBD	alpha-helical transmembrane proteins
KCMB3_HUMAN	potassium large conductance calcium-activated channel, subfamily M beta member 3	CaKB	alpha-helical transmembrane proteins
KCMA1_HUMAN	potassium large conductance calcium-activated channel, subfamily M, alpha member 1	BK_channel_a	alpha-helical transmembrane proteins
KCMB1_HUMAN	potassium large conductance calcium-activated channel, subfamily M, beta member 1	CaKB	alpha-helical transmembrane proteins
KCMB2_HUMAN	potassium large conductance calcium-activated channel, subfamily M, beta member 2	KcnmB2_inactiv,CaKB	alpha-helical transmembrane proteins
KCMB4_HUMAN	potassium large conductance calcium-activated channel, subfamily M, beta member 4	CaKB	alpha-helical transmembrane proteins
KCNQ1_HUMAN	potassium voltage-gated channel, KQT-like subfamily, member 1	KCNQ_channel	alpha-helical transmembrane proteins
KCNQ2_HUMAN	potassium voltage-gated channel, KQT-like subfamily, member 2	KCNQ_channel	alpha-helical transmembrane proteins
KCNQ3_HUMAN	potassium voltage-gated channel, KQT-like subfamily, member 3	KCNQ_channel	alpha-helical transmembrane proteins
KCNQ4_HUMAN	potassium voltage-gated channel, KQT-like subfamily, member 4	KCNQ_channel	alpha-helical transmembrane proteins
A6PVT6_HUMAN	potassium voltage-gated channel, KQT-like subfamily, member 5	KCNQ_channel	N/A
KCNQ5_HUMAN	potassium voltage-gated channel, KQT-like subfamily, member 5	KCNQ_channel	alpha-helical transmembrane proteins
KCNB1_HUMAN	potassium voltage-gated channel, Shab-related subfamily, member 1	K_tetra	alpha-helical transmembrane proteins
KCNB2_HUMAN	potassium voltage-gated channel, Shab-related subfamily, member 2	K_tetra	alpha-helical transmembrane proteins
KCND1_HUMAN	potassium voltage-gated channel, Shal-related subfamily, member 1	DUF3399,K_tetra	alpha-helical transmembrane proteins
KCND2_HUMAN	potassium voltage-gated channel, Shal-related subfamily, member 2	DUF3399,K_tetra	alpha-helical transmembrane proteins
KCND3_HUMAN	potassium voltage-gated channel, Shal-related subfamily, member 3	DUF3399,K_tetra	alpha-helical transmembrane proteins
KCNC1_HUMAN	potassium voltage-gated channel, Shaw-related subfamily, member 1	K_tetra	alpha-helical transmembrane proteins
KCNC2_HUMAN	potassium voltage-gated channel, Shaw-related subfamily, member 2	K_tetra	alpha-helical transmembrane proteins
KCNC3_HUMAN	potassium voltage-gated channel, Shaw-related subfamily, member 3	K_tetra	alpha-helical transmembrane proteins
KCNC4_HUMAN	potassium voltage-gated channel, Shaw-related subfamily, member 4	K_tetra	alpha-helical transmembrane proteins
KCNS1_HUMAN	potassium voltage-gated channel, delayed-rectifier, subfamily S, member 1	K_tetra	alpha-helical transmembrane proteins
KCNS2_HUMAN	potassium voltage-gated channel, delayed-rectifier, subfamily S, member 2	K_tetra	alpha-helical transmembrane proteins
KCNS3_HUMAN	potassium voltage-gated channel, delayed-rectifier, subfamily S, member 3	K_tetra	alpha-helical transmembrane proteins
KCNA1_HUMAN	potassium voltage-gated channel, shaker-related subfamily, member 1 (episodic ataxia with myokymia)	K_tetra	alpha-helical transmembrane proteins
KCA10_HUMAN	potassium voltage-gated channel, shaker-related subfamily, member 10	K_tetra	alpha-helical transmembrane proteins
KCNA2_HUMAN	potassium voltage-gated channel, shaker-related subfamily, member 2	K_tetra	alpha-helical transmembrane proteins
KCNA3_HUMAN	potassium voltage-gated channel, shaker-related subfamily, member 3	K_tetra	alpha-helical transmembrane proteins
KCNA4_HUMAN	potassium voltage-gated channel, shaker-related subfamily, member 4	K_tetra	alpha-helical transmembrane proteins
KCNA5_HUMAN	potassium voltage-gated channel, shaker-related subfamily, member 5	K_tetra	alpha-helical transmembrane proteins
KCNA6_HUMAN	potassium voltage-gated channel, shaker-related subfamily, member 6	K_tetra	alpha-helical transmembrane proteins
KCNA7_HUMAN	potassium voltage-gated channel, shaker-related subfamily, member 7	K_tetra	alpha-helical transmembrane proteins
KCNF1_HUMAN	potassium voltage-gated channel, subfamily F, member 1	K_tetra	alpha-helical transmembrane proteins
KCNG1_HUMAN	potassium voltage-gated channel, subfamily G, member 1	K_tetra	alpha-helical transmembrane proteins
KCNG2_HUMAN	potassium voltage-gated channel, subfamily G, member 2	K_tetra	alpha-helical transmembrane proteins
KCNG3_HUMAN	potassium voltage-gated channel, subfamily G, member 3	K_tetra	alpha-helical transmembrane proteins
KCNG4_HUMAN	potassium voltage-gated channel, subfamily G, member 4	K_tetra	alpha-helical transmembrane proteins
KCNH1_HUMAN	potassium voltage-gated channel, subfamily H (eag-related), member 1	PAS_9,cNMP_binding	alpha-helical transmembrane proteins
KCNH2_HUMAN	potassium voltage-gated channel, subfamily H (eag-related), member 2	PAS_9,cNMP_binding	alpha-helical transmembrane proteins
KCNH4_HUMAN	potassium voltage-gated channel, subfamily H (eag-related), member 4	PAS_9	alpha-helical transmembrane proteins
KCNH6_HUMAN	potassium voltage-gated channel, subfamily H (eag-related), member 6	PAS_9,cNMP_binding	alpha-helical transmembrane proteins
PSN1_HUMAN	presenilin 1	Presenilin	alpha-helical transmembrane proteins
PSN2_HUMAN	presenilin 2 (Alzheimer disease 4)	Presenilin	alpha-helical transmembrane proteins
PRIO_HUMAN	prion protein	Prion,Prion_bPrPp	N/A
PRND_HUMAN	prion protein 2 (dublet)	Prion	N/A
MPRA_HUMAN	progestin and adipoQ receptor family member VII	HlyIII	alpha-helical transmembrane proteins
MPRB_HUMAN	progestin and adipoQ receptor family member VIII	HlyIII	alpha-helical transmembrane proteins
PDCD1_HUMAN	programmed cell death 1	V-set	alpha-helical transmembrane proteins
PD1L2_HUMAN	programmed cell death 1 ligand 2	Ig_3	alpha-helical transmembrane proteins
TMG1_HUMAN	proline rich Gla (G-carboxyglutamic acid) 1	Gla	alpha-helical transmembrane proteins
TMG2_HUMAN	proline rich Gla (G-carboxyglutamic acid) 2	Gla	alpha-helical transmembrane proteins
PGH1_HUMAN	prostaglandin-endoperoxide synthase 1 (prostaglandin G/H synthase and cyclooxygenase)	An_peroxidase	N/A
EPCR_HUMAN	protein C receptor, endothelial (EPCR)	MHC_I	alpha-helical transmembrane proteins
PTN1_HUMAN	protein tyrosine phosphatase, non-receptor type 1	EF_hand_5,Spectrin,Y_phosphatase	N/A
PTPRA_HUMAN	protein tyrosine phosphatase, receptor type, A	Y_phosphatase	alpha-helical transmembrane proteins
PTPRD_HUMAN	protein tyrosine phosphatase, receptor type, D	I-set,fn3,Y_phosphatase	alpha-helical transmembrane proteins
PTPRE_HUMAN	protein tyrosine phosphatase, receptor type, E	Y_phosphatase	alpha-helical transmembrane proteins
PTPRF_HUMAN	protein tyrosine phosphatase, receptor type, F	I-set,fn3,Y_phosphatase	alpha-helical transmembrane proteins
PTPRG_HUMAN	protein tyrosine phosphatase, receptor type, G	Carb_anhydrase,fn3,Y_phosphatase	alpha-helical transmembrane proteins
PTPRH_HUMAN	protein tyrosine phosphatase, receptor type, H	fn3,Y_phosphatase	alpha-helical transmembrane proteins
PTPRJ_HUMAN	protein tyrosine phosphatase, receptor type, J	fn3,Y_phosphatase	alpha-helical transmembrane proteins
PTPRK_HUMAN	protein tyrosine phosphatase, receptor type, K	MAM,Ig_2,fn3,Y_phosphatase	alpha-helical transmembrane proteins
Q5TG12_HUMAN	protein tyrosine phosphatase, receptor type, K	fn3,Ig_2,MAM,Y_phosphatase	N/A
PTPRM_HUMAN	protein tyrosine phosphatase, receptor type, M	MAM,fn3,ig,Y_phosphatase	alpha-helical transmembrane proteins
PTPRN_HUMAN	protein tyrosine phosphatase, receptor type, N	Receptor_IA-2,Y_phosphatase	alpha-helical transmembrane proteins
PTPRO_HUMAN	protein tyrosine phosphatase, receptor type, O	fn3,Y_phosphatase	alpha-helical transmembrane proteins
PTPRR_HUMAN	protein tyrosine phosphatase, receptor type, R	Y_phosphatase	alpha-helical transmembrane proteins
PTPRS_HUMAN	protein tyrosine phosphatase, receptor type, S	I-set,fn3,Y_phosphatase	alpha-helical transmembrane proteins
Q8NHS7_HUMAN	protein tyrosine phosphatase, receptor type, S	I-set	N/A
PTPRU_HUMAN	protein tyrosine phosphatase, receptor type, U	MAM,fn3,Y_phosphatase	alpha-helical transmembrane proteins
PTPRZ_HUMAN	protein tyrosine phosphatase, receptor-type, Z polypeptide 1	fn3,Carb_anhydrase,Y_phosphatase	alpha-helical transmembrane proteins
PCDH1_HUMAN	protocadherin 1	Cadherin,Protocadherin,Cadherin_2	alpha-helical transmembrane proteins
PCD10_HUMAN	protocadherin 10	Cadherin,Cadherin_2	alpha-helical transmembrane proteins
PC11Y_HUMAN	protocadherin 11 Y-linked	Cadherin,Protocadherin,Cadherin_2	alpha-helical transmembrane proteins
PCD12_HUMAN	protocadherin 12	Cadherin,Cadherin_2	alpha-helical transmembrane proteins
A2A3E3_HUMAN	protocadherin 15	Cadherin	N/A
PCD15_HUMAN	protocadherin 15	Cadherin	alpha-helical transmembrane proteins
A2A3E7_HUMAN	protocadherin 15	Cadherin	N/A
A2A3E8_HUMAN	protocadherin 15	Cadherin	N/A
A2A3E6_HUMAN	protocadherin 15	Cadherin	N/A
PCD18_HUMAN	protocadherin 18	Cadherin,Cadherin_2	alpha-helical transmembrane proteins
PCD19_HUMAN	protocadherin 19	Cadherin,Cadherin_2	alpha-helical transmembrane proteins
PCD20_HUMAN	protocadherin 20	Cadherin	alpha-helical transmembrane proteins
PCDH7_HUMAN	protocadherin 7	Cadherin,Protocadherin,Cadherin_2	alpha-helical transmembrane proteins
PCDH8_HUMAN	protocadherin 8	Cadherin,Cadherin_2	alpha-helical transmembrane proteins
Q5VT82_HUMAN	protocadherin 9	Cadherin,Protocadherin,Cadherin_2	N/A
PCDH9_HUMAN	protocadherin 9	Cadherin,Protocadherin,Cadherin_2	alpha-helical transmembrane proteins
PCDAB_HUMAN	protocadherin alpha 11	Cadherin,Cadherin_2	alpha-helical transmembrane proteins
PCDAC_HUMAN	protocadherin alpha 12	Cadherin,Cadherin_2	alpha-helical transmembrane proteins
PCDAA_HUMAN	protocadherin alpha 13; protocadherin alpha 10; protocadherin alpha subfamily C, 1; protocadherin alpha subfamily C, 2	Cadherin,Cadherin_2	alpha-helical transmembrane proteins
PCDC2_HUMAN	protocadherin alpha 13; protocadherin alpha 10; protocadherin alpha subfamily C, 1; protocadherin alpha subfamily C, 2	Cadherin,Cadherin_2	alpha-helical transmembrane proteins
PCDC1_HUMAN	protocadherin alpha 13; protocadherin alpha 10; protocadherin alpha subfamily C, 1; protocadherin alpha subfamily C, 2	Cadherin,Cadherin_2	alpha-helical transmembrane proteins
PCDA4_HUMAN	protocadherin alpha 1; protocadherin alpha 4	Cadherin,Cadherin_2	alpha-helical transmembrane proteins
PCDA5_HUMAN	protocadherin alpha 5	Cadherin,Cadherin_2	alpha-helical transmembrane proteins
PCDA8_HUMAN	protocadherin alpha 8; protocadherin alpha 6	Cadherin,Cadherin_2	alpha-helical transmembrane proteins
PCDA9_HUMAN	protocadherin alpha 9	Cadherin,Cadherin_2	alpha-helical transmembrane proteins
PCDB1_HUMAN	protocadherin beta 1	Cadherin,Cadherin_2	alpha-helical transmembrane proteins
PCDBA_HUMAN	protocadherin beta 10; protocadherin beta 9	Cadherin,Cadherin_2	alpha-helical transmembrane proteins
PCDB9_HUMAN	protocadherin beta 10; protocadherin beta 9	Cadherin,Cadherin_2	alpha-helical transmembrane proteins
PCDBB_HUMAN	protocadherin beta 11	Cadherin,Cadherin_2	alpha-helical transmembrane proteins
PCDBC_HUMAN	protocadherin beta 12	Cadherin,Cadherin_2	alpha-helical transmembrane proteins
PCDBD_HUMAN	protocadherin beta 13	Cadherin,Cadherin_2	alpha-helical transmembrane proteins
PCDBE_HUMAN	protocadherin beta 14	Cadherin,Cadherin_2	alpha-helical transmembrane proteins
PCDBF_HUMAN	protocadherin beta 15	Cadherin,Cadherin_2	alpha-helical transmembrane proteins
PCDBG_HUMAN	protocadherin beta 16	Cadherin,Cadherin_2	alpha-helical transmembrane proteins

PCDBI_HUMAN	protocadherin beta 18 pseudogene	Cadherin,Cadherin_2	alpha-helical transmembrane proteins
PCDB2_HUMAN	protocadherin beta 2	Cadherin,Cadherin_2	alpha-helical transmembrane proteins
PCDB3_HUMAN	protocadherin beta 3	Cadherin,Cadherin_2	alpha-helical transmembrane proteins
PCDB4_HUMAN	protocadherin beta 4	Cadherin,Cadherin_2	alpha-helical transmembrane proteins
PCDB5_HUMAN	protocadherin beta 5	Cadherin,Cadherin_2	alpha-helical transmembrane proteins
PCDB6_HUMAN	protocadherin beta 6	Cadherin,Cadherin_2	alpha-helical transmembrane proteins
PCDB7_HUMAN	protocadherin beta 7	Cadherin,Cadherin_2	alpha-helical transmembrane proteins
PCDB8_HUMAN	protocadherin beta 8	Cadherin,Cadherin_2	alpha-helical transmembrane proteins
PCDG1_HUMAN	protocadherin gamma subfamily A, 1	Cadherin,Cadherin_2	alpha-helical transmembrane proteins
PCDGA_HUMAN	protocadherin gamma subfamily A, 10	Cadherin,Cadherin_2	alpha-helical transmembrane proteins
PCDGB_HUMAN	protocadherin gamma subfamily A, 11	Cadherin,Cadherin_2	alpha-helical transmembrane proteins
PCDG2_HUMAN	protocadherin gamma subfamily A, 2	Cadherin,Cadherin_2	alpha-helical transmembrane proteins
PCDG3_HUMAN	protocadherin gamma subfamily A, 3	Cadherin,Cadherin_2	alpha-helical transmembrane proteins
PCDG4_HUMAN	protocadherin gamma subfamily A, 4	Cadherin,Cadherin_2	alpha-helical transmembrane proteins
PCDG5_HUMAN	protocadherin gamma subfamily A, 5	Cadherin,Cadherin_2	alpha-helical transmembrane proteins
PCDG6_HUMAN	protocadherin gamma subfamily A, 6	Cadherin,Cadherin_2	alpha-helical transmembrane proteins
PCDG7_HUMAN	protocadherin gamma subfamily A, 7	Cadherin,Cadherin_2	alpha-helical transmembrane proteins
PCDG8_HUMAN	protocadherin gamma subfamily A, 8	Cadherin,Cadherin_2	alpha-helical transmembrane proteins
PCDG9_HUMAN	protocadherin gamma subfamily A, 9	Cadherin,Cadherin_2	alpha-helical transmembrane proteins
PCDGD_HUMAN	protocadherin gamma subfamily B, 1	Cadherin,Cadherin_2	alpha-helical transmembrane proteins
PCDGE_HUMAN	protocadherin gamma subfamily B, 2	Cadherin,Cadherin_2	alpha-helical transmembrane proteins
PCDGF_HUMAN	protocadherin gamma subfamily B, 3	Cadherin,Cadherin_2	alpha-helical transmembrane proteins
PCDGG_HUMAN	protocadherin gamma subfamily B, 4	Cadherin,Cadherin_2	alpha-helical transmembrane proteins
PCDGH_HUMAN	protocadherin gamma subfamily B, 5	Cadherin,Cadherin_2	alpha-helical transmembrane proteins
PCDGI_HUMAN	protocadherin gamma subfamily B, 6	Cadherin,Cadherin_2	alpha-helical transmembrane proteins
PCDGJ_HUMAN	protocadherin gamma subfamily B, 7	Cadherin,Cadherin_2	alpha-helical transmembrane proteins
PCDGM_HUMAN	protocadherin gamma subfamily C, 3; protocadherin gamma subfamily C, 5; protocadherin gamma subfamily C, 4; protocadherin gamma subfamily A, 12	Cadherin,Cadherin_2	alpha-helical transmembrane proteins
PCDGK_HUMAN	protocadherin gamma subfamily C, 3; protocadherin gamma subfamily C, 5; protocadherin gamma subfamily C, 4; protocadherin gamma subfamily A, 12	Cadherin,Cadherin_2	alpha-helical transmembrane proteins
PCDGL_HUMAN	protocadherin gamma subfamily C, 3; protocadherin gamma subfamily C, 5; protocadherin gamma subfamily C, 4; protocadherin gamma subfamily A, 12	Cadherin,Cadherin_2	alpha-helical transmembrane proteins
PCDGC_HUMAN	protocadherin gamma subfamily C, 3; protocadherin gamma subfamily C, 5; protocadherin gamma subfamily C, 4; protocadherin gamma subfamily A, 12	Cadherin,Cadherin_2	alpha-helical transmembrane proteins
P2RX1_HUMAN	purinergic receptor P2X, ligand-gated ion channel, 1	P2X_receptor	alpha-helical transmembrane proteins
P2RX3_HUMAN	purinergic receptor P2X, ligand-gated ion channel, 3	P2X_receptor	alpha-helical transmembrane proteins
P2RX4_HUMAN	purinergic receptor P2X, ligand-gated ion channel, 4	P2X_receptor	alpha-helical transmembrane proteins
P2RX5_HUMAN	purinergic receptor P2X, ligand-gated ion channel, 5	P2X_receptor	alpha-helical transmembrane proteins
Q05BG9_HUMAN	purinergic receptor P2X, ligand-gated ion channel, 6	P2X_receptor	N/A
P2RX6_HUMAN	purinergic receptor P2X, ligand-gated ion channel, 6	P2X_receptor	alpha-helical transmembrane proteins
P2RX7_HUMAN	purinergic receptor P2X, ligand-gated ion channel, 7	P2X_receptor	alpha-helical transmembrane proteins
QSOX2_HUMAN	quiescin Q6 sulfhydryl oxidase 2	Evr1_Alr,Thioredoxin	alpha-helical transmembrane proteins
RAMP1_HUMAN	receptor (G protein-coupled) activity modifying protein 1	RAMP	alpha-helical transmembrane proteins
RAMP2_HUMAN	receptor (G protein-coupled) activity modifying protein 2	RAMP	alpha-helical transmembrane proteins
RAMP3_HUMAN	receptor (G protein-coupled) activity modifying protein 3	RAMP	alpha-helical transmembrane proteins
RTP1_HUMAN	receptor (chemosensory) transporter protein 1	RabGAP-TBC,zf-3CxxC	alpha-helical transmembrane proteins
RTP2_HUMAN	receptor (chemosensory) transporter protein 2	zf-3CxxC	alpha-helical transmembrane proteins
ROR1_HUMAN	receptor tyrosine kinase-like orphan receptor 1	Pkinase_Tyr,Fz,I-set,Kringle	alpha-helical transmembrane proteins
ROR2_HUMAN	receptor tyrosine kinase-like orphan receptor 2	Pkinase_Tyr,Fz,Kringle,I-set	alpha-helical transmembrane proteins
RXFP1_HUMAN	relaxin/insulin-like family peptide receptor 1	LRR_8,Ldl_recept_a	alpha-helical transmembrane proteins
RXFP2_HUMAN	relaxin/insulin-like family peptide receptor 2	LRR_8,Ldl_recept_a	alpha-helical transmembrane proteins
N2DL4_HUMAN	retinoic acid early transcript 1E	MHC_I	alpha-helical transmembrane proteins
RET1L_HUMAN	retinoic acid early transcript 1L	MHC_I	N/A
RECK_HUMAN	reversion-inducing-cysteine-rich protein with kazal motifs	Kazal_2	N/A
RHBL1_HUMAN	rhomboid, veinlet-like 1 (Drosophila)	Rhomboid	alpha-helical transmembrane proteins
ROBO2_HUMAN	roundabout, axon guidance receptor, homolog 2 (Drosophila)	I-set,fn3	alpha-helical transmembrane proteins
RYR1_HUMAN	ryanodine receptor 1 (skeletal)	SPRY,RR_TM4-6,Ins145_P3_rec,RYDR_ITPR,RIH_assoc,EF_hand_6,MIR,RyR	alpha-helical transmembrane proteins
RYR2_HUMAN	ryanodine receptor 2 (cardiac)	SPRY,RR_TM4-6,Ins145_P3_rec,RIH_assoc,RYDR_ITPR,MIR,RyR	alpha-helical transmembrane proteins
SGCZ_HUMAN	sarcoglycan zeta	Sarcoglycan_1	alpha-helical transmembrane proteins
Q08AT0_HUMAN	sarcoglycan zeta	Sarcoglycan_1	N/A
SGCA_HUMAN	sarcoglycan, alpha (50kDa dystrophin-associated glycoprotein)	Sarcoglycan_2	alpha-helical transmembrane proteins
SGCB_HUMAN	sarcoglycan, beta (43kDa dystrophin-associated glycoprotein)	Sarcoglycan_1	alpha-helical transmembrane proteins
SGCD_HUMAN	sarcoglycan, delta (35kDa dystrophin-associated glycoprotein)	Sarcoglycan_1	alpha-helical transmembrane proteins
SGCE_HUMAN	sarcoglycan, epsilon	Sarcoglycan_2	alpha-helical transmembrane proteins
SGCG_HUMAN	sarcoglycan, gamma (35kDa dystrophin-associated glycoprotein)	Sarcoglycan_1	alpha-helical transmembrane proteins
SLMAP_HUMAN	sarcolemma associated protein	FHA	alpha-helical transmembrane proteins
SSPN_HUMAN	sarcospan (Kras oncogene-associated gene)	CD20	alpha-helical transmembrane proteins
SCAR5_HUMAN	scavenger receptor class A, member 5 (putative)	SRCR,Collagen	alpha-helical transmembrane proteins
SCTR_HUMAN	secretin receptor	HRM	alpha-helical transmembrane proteins
SCAM5_HUMAN	secretory carrier membrane protein 5	SCAMP	alpha-helical transmembrane proteins
SEZ6_HUMAN	seizure related 6 homolog (mouse)	Sushi,CUB	alpha-helical transmembrane proteins
SE6L2_HUMAN	seizure related 6 homolog (mouse)-like 2	Sushi,CUB	alpha-helical transmembrane proteins
Q5TI75_HUMAN	selectin E	Sushi,EGF,Lectin_C	N/A
LYAM2_HUMAN	selectin E	Sushi,EGF,Lectin_C	alpha-helical transmembrane proteins
LYAM1_HUMAN	selectin L	Sushi,EGF,Lectin_C	alpha-helical transmembrane proteins
LYAM3_HUMAN	selectin P (granule membrane protein 140kDa, antigen CD62)	Sushi,Lectin_C	alpha-helical transmembrane proteins
SEM4A_HUMAN	sema domain, immunoglobulin domain (Ig), transmembrane domain (TM) and short cytoplasmic domain, (semaphorin) 4A	PSI,Sema	alpha-helical transmembrane proteins
Q5TCJ5_HUMAN	sema domain, immunoglobulin domain (Ig), transmembrane domain (TM) and short cytoplasmic domain, (semaphorin) 4A	PSI,Sema	N/A
SEM4D_HUMAN	sema domain, immunoglobulin domain (Ig), transmembrane domain (TM) and short cytoplasmic domain, (semaphorin) 4D	PSI,Sema,ig	alpha-helical transmembrane proteins
SEM4F_HUMAN	sema domain, immunoglobulin domain (Ig), transmembrane domain (TM) and short cytoplasmic domain, (semaphorin) 4F	PSI,Sema	alpha-helical transmembrane proteins
SEM5A_HUMAN	sema domain, seven thrombospondin repeats (type 1 and type 1-like), transmembrane domain (TM) and short cytoplasmic domain, (semaphorin) 5A	TSP_1,Sema	alpha-helical transmembrane proteins
SEM6D_HUMAN	sema domain, transmembrane domain (TM), and cytoplasmic domain, (semaphorin) 6D	PSI,Sema	alpha-helical transmembrane proteins
SERC1_HUMAN	serine incorporator 1	Serinc	alpha-helical transmembrane proteins
Q5H936_HUMAN	serine incorporator 3	Serinc	N/A
SERC3_HUMAN	serine incorporator 3	Serinc	alpha-helical transmembrane proteins
SPIT2_HUMAN	serine peptidase inhibitor, Kunitz type, 2	Kunitz_BPTI	alpha-helical transmembrane proteins
STYK1_HUMAN	serine/threonine/tyrosine kinase 1	Pkinase_Tyr	alpha-helical transmembrane proteins
SN_HUMAN	sialic acid binding Ig-like lectin 1, sialoadhesin	C1-set,C2-set_2,TPR_12,zf-RING_2,LRRNT,V-set,LRR_8,I-set,Ras,Ig_2	alpha-helical transmembrane proteins
SIG10_HUMAN	sialic acid binding Ig-like lectin 10	C2-set_2,V-set,Ig_2	alpha-helical transmembrane proteins
SIGL6_HUMAN	sialic acid binding Ig-like lectin 6	I-set,V-set,ig	alpha-helical transmembrane proteins
SIGL7_HUMAN	sialic acid binding Ig-like lectin 7	V-set,Ig_2	alpha-helical transmembrane proteins
SIGL9_HUMAN	sialic acid binding Ig-like lectin 9	V-set,Ig_2	alpha-helical transmembrane proteins
NEUR1_HUMAN	sialidase 1 (lysosomal sialidase)	BNR_2	N/A
SHPS1_HUMAN	signal-regulatory protein alpha	C1-set,V-set	alpha-helical transmembrane proteins
SIRB1_HUMAN	signal-regulatory protein beta 1	C1-set,V-set	alpha-helical transmembrane proteins
SIRBL_HUMAN	signal-regulatory protein beta 1	C1-set,V-set	alpha-helical transmembrane proteins
SIRPG_HUMAN	signal-regulatory protein gamma	C1-set,V-set	alpha-helical transmembrane proteins
2B17_HUMAN	similar to HLA class II histocompatibility antigen, DRB1-7 beta chain	C1-set,MHC_II_beta	alpha-helical transmembrane proteins
2B17_HUMAN	similar to HLA class II histocompatibility antigen, DRB1-7 beta chain	C1-set,MHC_II_beta	alpha-helical transmembrane proteins
2B19_HUMAN	similar to HLA class II histocompatibility antigen, DRB1-9 beta chain	C1-set,MHC_II_beta	alpha-helical transmembrane proteins
MOT6_HUMAN	similar to MCT; solute carrier family 16, member 5 (monocarboxylic acid transporter 6)	MFS_1	alpha-helical transmembrane proteins
KIT_HUMAN	similar to Mast/stem cell growth factor receptor precursor (SCFR) (Proto-oncogene tyrosine-protein kinase Kit) (c-kit) (CD117 antigen); v-kit Hardy-Zuckerman 4 feline sarcoma viral oncogene homolog	Pkinase_Tyr,Ig_3,ig	alpha-helical transmembrane proteins
DRB3_HUMAN	similar to major histocompatibility complex, class II, DR beta 3	C1-set,MHC_II_beta	alpha-helical transmembrane proteins
STEA2_HUMAN	six transmembrane epithelial antigen of the prostate 2	F420_oxidored	alpha-helical transmembrane proteins
SCNNA_HUMAN	sodium channel, nonvoltage-gated 1 alpha	ASC	alpha-helical transmembrane proteins
SCNNB_HUMAN	sodium channel, nonvoltage-gated 1, beta	ASC	alpha-helical transmembrane proteins
SCNNG_HUMAN	sodium channel, nonvoltage-gated 1, gamma	ASC	alpha-helical transmembrane proteins
SCN2B_HUMAN	sodium channel, voltage-gated, type II, beta	V-set	alpha-helical transmembrane proteins
SCN3B_HUMAN	sodium channel, voltage-gated, type III, beta	V-set	alpha-helical transmembrane proteins

SCN4B_HUMAN	sodium channel, voltage-gated, type IV, beta	V-set	alpha-helical transmembrane proteins
EAA2_HUMAN	solute carrier family 1 (glial high affinity glutamate transporter), member 2	SDF	alpha-helical transmembrane proteins
EAA1_HUMAN	solute carrier family 1 (glial high affinity glutamate transporter), member 3	SDF	alpha-helical transmembrane proteins
SATT_HUMAN	solute carrier family 1 (glutamate/neutral amino acid transporter), member 4	SDF	alpha-helical transmembrane proteins
EAA4_HUMAN	solute carrier family 1 (high affinity aspartate/glutamate transporter), member 6	SDF	alpha-helical transmembrane proteins
EAA3_HUMAN	solute carrier family 1 (neuronal/epithelial high affinity glutamate transporter, system Xag), member 1	SDF	alpha-helical transmembrane proteins
AAAT_HUMAN	solute carrier family 1 (neutral amino acid transporter), member 5	SDF	alpha-helical transmembrane proteins
NRAM1_HUMAN	solute carrier family 11 (proton-coupled divalent metal ion transporters), member 1	Nramp	alpha-helical transmembrane proteins
NRAM2_HUMAN	solute carrier family 11 (proton-coupled divalent metal ion transporters), member 2	Nramp	alpha-helical transmembrane proteins
Q5VZ42_HUMAN	solute carrier family 12 (potassium-chloride transporter), member 5	AA_permease	N/A
S12A5_HUMAN	solute carrier family 12 (potassium-chloride transporter), member 5	AA_permease	alpha-helical transmembrane proteins
S12A4_HUMAN	solute carrier family 12 (potassium/chloride transporters), member 4	AA_permease	alpha-helical transmembrane proteins
S12A6_HUMAN	solute carrier family 12 (potassium/chloride transporters), member 6	AA_permease	alpha-helical transmembrane proteins
S12A7_HUMAN	solute carrier family 12 (potassium/chloride transporters), member 7	AA_permease	alpha-helical transmembrane proteins
S12A9_HUMAN	solute carrier family 12 (potassium/chloride transporters), member 9	AA_permease	alpha-helical transmembrane proteins
S12A3_HUMAN	solute carrier family 12 (sodium/chloride transporters), member 3	AA_permease_N,AA_permease	alpha-helical transmembrane proteins
S12A1_HUMAN	solute carrier family 12 (sodium/potassium/chloride transporters), member 1	AA_permease_N,AA_permease	alpha-helical transmembrane proteins
Q8IUN5_HUMAN	solute carrier family 12 (sodium/potassium/chloride transporters), member 1	AA_permease_N,AA_permease	N/A
S12A2_HUMAN	solute carrier family 12 (sodium/potassium/chloride transporters), member 2	AA_permease_N,AA_permease	alpha-helical transmembrane proteins
UT1_HUMAN	solute carrier family 14 (urea transporter), member 1 (Kidd blood group)	UT	alpha-helical transmembrane proteins
UT2_HUMAN	solute carrier family 14 (urea transporter), member 2	UT,DUF3595,UCH	alpha-helical transmembrane proteins
Q49A45_HUMAN	solute carrier family 16, member 1 (monocarboxylic acid transporter 1)	MFS_1	N/A
Q5T8R4_HUMAN	solute carrier family 16, member 1 (monocarboxylic acid transporter 1)	MFS_1	N/A
Q5T8R5_HUMAN	solute carrier family 16, member 1 (monocarboxylic acid transporter 1)	MFS_1	N/A
MOT1_HUMAN	solute carrier family 16, member 1 (monocarboxylic acid transporter 1)	MFS_1	alpha-helical transmembrane proteins
Q5T8R3_HUMAN	solute carrier family 16, member 1 (monocarboxylic acid transporter 1)	MFS_1	N/A
Q9P1I2_HUMAN	solute carrier family 16, member 10 (aromatic amino acid transporter)	MFS_1	N/A
MOT10_HUMAN	solute carrier family 16, member 10 (aromatic amino acid transporter)	MFS_1	alpha-helical transmembrane proteins
MOT11_HUMAN	solute carrier family 16, member 11 (monocarboxylic acid transporter 11)	MFS_1	alpha-helical transmembrane proteins
MOT12_HUMAN	solute carrier family 16, member 12 (monocarboxylic acid transporter 12)	MFS_1	alpha-helical transmembrane proteins
MOT13_HUMAN	solute carrier family 16, member 13 (monocarboxylic acid transporter 13)	MFS_1	alpha-helical transmembrane proteins
MOT14_HUMAN	solute carrier family 16, member 14 (monocarboxylic acid transporter 14)	MFS_1	alpha-helical transmembrane proteins
Q6ZWE5_HUMAN	solute carrier family 16, member 14 (monocarboxylic acid transporter 14)	MFS_1	N/A
MOT8_HUMAN	solute carrier family 16, member 2 (monocarboxylic acid transporter 8)	MFS_1	alpha-helical transmembrane proteins
MOT4_HUMAN	solute carrier family 16, member 3 (monocarboxylic acid transporter 4)	MFS_1	alpha-helical transmembrane proteins
MOT5_HUMAN	solute carrier family 16, member 4 (monocarboxylic acid transporter 5)	MFS_1	alpha-helical transmembrane proteins
MOT7_HUMAN	solute carrier family 16, member 6 (monocarboxylic acid transporter 7); similar to solute carrier family 16, member 6	MFS_1	alpha-helical transmembrane proteins
MOT3_HUMAN	solute carrier family 16, member 8 (monocarboxylic acid transporter 3)	MFS_1	alpha-helical transmembrane proteins
MOT9_HUMAN	solute carrier family 16, member 9 (monocarboxylic acid transporter 9)	MFS_1	alpha-helical transmembrane proteins
S17A5_HUMAN	solute carrier family 17 (anion/sugar transporter), member 5	MFS_1	alpha-helical transmembrane proteins
NPT1_HUMAN	solute carrier family 17 (sodium phosphate), member 1	MFS_1,KH_1	alpha-helical transmembrane proteins
NPT3_HUMAN	solute carrier family 17 (sodium phosphate), member 2	MFS_1	alpha-helical transmembrane proteins
NPT4_HUMAN	solute carrier family 17 (sodium phosphate), member 3	MFS_1	alpha-helical transmembrane proteins
S17A4_HUMAN	solute carrier family 17 (sodium phosphate), member 4	MFS_1	alpha-helical transmembrane proteins
VGLU2_HUMAN	solute carrier family 17 (sodium-dependent inorganic phosphate cotransporter), member 6	MFS_1	alpha-helical transmembrane proteins
VGLU1_HUMAN	solute carrier family 17 (sodium-dependent inorganic phosphate cotransporter), member 7	MFS_1	alpha-helical transmembrane proteins
VGLU3_HUMAN	solute carrier family 17 (sodium-dependent inorganic phosphate cotransporter), member 8	MFS_1	alpha-helical transmembrane proteins
VACHT_HUMAN	solute carrier family 18 (vesicular acetylcholine), member 3	MFS_1	alpha-helical transmembrane proteins
VMAT2_HUMAN	solute carrier family 18 (vesicular monoamine), member 2	MFS_1	alpha-helical transmembrane proteins
S19A1_HUMAN	solute carrier family 19 (folate transporter), member 1	Folate_carrier	alpha-helical transmembrane proteins
S19A2_HUMAN	solute carrier family 19 (thiamine transporter), member 2	Folate_carrier	alpha-helical transmembrane proteins
Q96KH7_HUMAN	solute carrier family 22 (extraneuronal monoamine transporter), member 3	MFS_1	N/A
S22A3_HUMAN	solute carrier family 22 (extraneuronal monoamine transporter), member 3	MFS_1	alpha-helical transmembrane proteins
Q5T047_HUMAN	solute carrier family 22 (organic anion transporter), member 7	MFS_1	N/A
Q5T051_HUMAN	solute carrier family 22 (organic anion transporter), member 7	MFS_1	N/A
S22A7_HUMAN	solute carrier family 22 (organic anion transporter), member 7	MFS_1	alpha-helical transmembrane proteins
S22AC_HUMAN	solute carrier family 22 (organic anion/urate transporter), member 12	MFS_1	alpha-helical transmembrane proteins
S22AI_HUMAN	solute carrier family 22, member 18	MFS_1	alpha-helical transmembrane proteins
S23A1_HUMAN	solute carrier family 23 (nucleobase transporters), member 1	Xan_ur_permease	alpha-helical transmembrane proteins
S23A2_HUMAN	solute carrier family 23 (nucleobase transporters), member 2	Xan_ur_permease	alpha-helical transmembrane proteins
NCKX1_HUMAN	solute carrier family 24 (sodium/potassium/calcium exchanger), member 1	Na_Ca_ex	alpha-helical transmembrane proteins
NCKX6_HUMAN	solute carrier family 24 (sodium/potassium/calcium exchanger), member 6	Na_Ca_ex	alpha-helical transmembrane proteins
ADT1_HUMAN	solute carrier family 25 (mitochondrial carrier; adenine nucleotide translocator), member 4	Mito_carr	alpha-helical transmembrane proteins
ADT2_HUMAN	solute carrier family 25 (mitochondrial carrier; adenine nucleotide translocator), member 5; solute carrier family 25 (mitochondrial carrier; adenine nucleotide translocator), member 5 pseudogene 8	Mito_carr	alpha-helical transmembrane proteins
PM34_HUMAN	solute carrier family 25 (mitochondrial carrier; peroxisomal membrane protein, 34kDa), member 17	Mito_carr	alpha-helical transmembrane proteins
MPCP_HUMAN	solute carrier family 25 (mitochondrial carrier; phosphate carrier), member 3	Mito_carr	alpha-helical transmembrane proteins
S26A2_HUMAN	solute carrier family 26 (sulfate transporter), member 2	Sulfate_transp,Sulfate_tra_GLY,STAS	alpha-helical transmembrane proteins
S2611_HUMAN	solute carrier family 26, member 11	Sulfate_transp,Sulfate_tra_GLY,STAS	alpha-helical transmembrane proteins
S26A3_HUMAN	solute carrier family 26, member 3	Sulfate_transp,Sulfate_tra_GLY,STAS	alpha-helical transmembrane proteins
S26A4_HUMAN	solute carrier family 26, member 4	Sulfate_transp,Sulfate_tra_GLY,STAS	alpha-helical transmembrane proteins
Q7Z7F4_HUMAN	solute carrier family 26, member 5 (prestin)	Sulfate_transp,Sulfate_tra_GLY,STAS	N/A
Q496J0_HUMAN	solute carrier family 26, member 5 (prestin)	Sulfate_transp,Sulfate_tra_GLY	N/A
S26A5_HUMAN	solute carrier family 26, member 5 (prestin)	Sulfate_transp,Sulfate_tra_GLY,STAS	alpha-helical transmembrane proteins
Q496J3_HUMAN	solute carrier family 26, member 5 (prestin)	Sulfate_transp,Sulfate_tra_GLY	N/A
S26A6_HUMAN	solute carrier family 26, member 6; cadherin, EGF LAG seven-pass G-type receptor 3 (flamingo homolog, Drosophila)	Sulfate_transp,Sulfate_tra_GLY,STAS	alpha-helical transmembrane proteins
CELR3_HUMAN	solute carrier family 26, member 6; cadherin, EGF LAG seven-pass G-type receptor 3 (flamingo homolog, Drosophila)	Cadherin,DUF3497,Laminin_G_2,EGF,Laminin_EGF	alpha-helical transmembrane proteins
S26A7_HUMAN	solute carrier family 26, member 7	Sulfate_transp,Sulfate_tra_GLY,STAS	alpha-helical transmembrane proteins
S26A8_HUMAN	solute carrier family 26, member 8	Sulfate_transp,Sulfate_tra_GLY,STAS	alpha-helical transmembrane proteins
S26A9_HUMAN	solute carrier family 26, member 9	Sulfate_transp,Sulfate_tra_GLY,STAS	alpha-helical transmembrane proteins
S27A1_HUMAN	solute carrier family 27 (fatty acid transporter), member 1	AMP-binding	alpha-helical transmembrane proteins
4F2_HUMAN	solute carrier family 3 (activators of dibasic and neutral amino acid transport), member 2	Homeobox,Alpha-amylase,p450	alpha-helical transmembrane proteins
SLC31_HUMAN	solute carrier family 3 (cystine, dibasic and neutral amino acid transporters, activator of cystine, dibasic and neutral amino acid transport), member 1	Alpha-amylase	alpha-helical transmembrane proteins
ZNT1_HUMAN	solute carrier family 30 (zinc transporter), member 1	Cation_efflux	alpha-helical transmembrane proteins
ZNT3_HUMAN	solute carrier family 30 (zinc transporter), member 3	Cation_efflux	alpha-helical transmembrane proteins
ZNT5_HUMAN	solute carrier family 30 (zinc transporter), member 5	Cation_efflux	alpha-helical transmembrane proteins
ZNT8_HUMAN	solute carrier family 30 (zinc transporter), member 8	Cation_efflux	alpha-helical transmembrane proteins
ZNT10_HUMAN	solute carrier family 30, member 10	Cation_efflux	alpha-helical transmembrane proteins
S35A1_HUMAN	solute carrier family 35 (CMP-sialic acid transporter), member A1	Nuc_sug_transp	alpha-helical transmembrane proteins
Q5W1L7_HUMAN	solute carrier family 35 (CMP-sialic acid transporter), member A1	Nuc_sug_transp	N/A
S39A5_HUMAN	solute carrier family 39 (metal ion transporter), member 5	Zip	alpha-helical transmembrane proteins
S39A1_HUMAN	solute carrier family 39 (zinc transporter), member 1	Zip	alpha-helical transmembrane proteins
Q5T4K4_HUMAN	solute carrier family 39 (zinc transporter), member 1	Zip	N/A
Q5T4K3_HUMAN	solute carrier family 39 (zinc transporter), member 1	Zip	N/A
Q5T4K2_HUMAN	solute carrier family 39 (zinc transporter), member 1	Zip	N/A
S39AE_HUMAN	solute carrier family 39 (zinc transporter), member 14	Zip	alpha-helical transmembrane proteins
S39A2_HUMAN	solute carrier family 39 (zinc transporter), member 2	Zip	alpha-helical transmembrane proteins
S39A4_HUMAN	solute carrier family 39 (zinc transporter), member 4	Zip	alpha-helical transmembrane proteins
S39A6_HUMAN	solute carrier family 39 (zinc transporter), member 6	Zip	alpha-helical transmembrane proteins
B3AT_HUMAN	solute carrier family 4, anion exchanger, member 1 (erythrocyte membrane protein band 3, Diego blood group)	Band_3_cyto	alpha-helical transmembrane proteins
B3A2_HUMAN	solute carrier family 4, anion exchanger, member 2 (erythrocyte membrane protein band 3-like 1)	Band_3_cyto	alpha-helical transmembrane proteins
B3A3_HUMAN	solute carrier family 4, anion exchanger, member 3	Band_3_cyto	alpha-helical transmembrane proteins
S4A4_HUMAN	solute carrier family 4, sodium bicarbonate cotransporter, member 4	Band_3_cyto	alpha-helical transmembrane proteins
S4A5_HUMAN	solute carrier family 4, sodium bicarbonate cotransporter, member 5	Band_3_cyto,CD20	alpha-helical transmembrane proteins
S4A7_HUMAN	solute carrier family 4, sodium bicarbonate cotransporter, member 7	Band_3_cyto,CD20	alpha-helical transmembrane proteins
B3A4_HUMAN	solute carrier family 4, sodium bicarbonate cotransporter, member 9	Band_3_cyto	alpha-helical transmembrane proteins
S4A10_HUMAN	solute carrier family 4, sodium bicarbonate transporter, member 10	Band_3_cyto	alpha-helical transmembrane proteins
S40A1_HUMAN	solute carrier family 40 (iron-regulated transporter), member 1	FPN1	alpha-helical transmembrane proteins
LAT3_HUMAN	solute carrier family 43, member 1	MFS_1	alpha-helical transmembrane proteins
LAT4_HUMAN	solute carrier family 43, member 2	MFS_1	alpha-helical transmembrane proteins
PCFT_HUMAN	solute carrier family 46 (folate transporter), member 1	MFS_1	alpha-helical transmembrane proteins
TSCOT_HUMAN	solute carrier family 46, member 2	MFS_1	alpha-helical transmembrane proteins

S6A14_HUMAN	solute carrier family 6 (amino acid transporter), member 14	SNF	alpha-helical transmembrane proteins
SC6A1_HUMAN	solute carrier family 6 (neurotransmitter transporter, GABA), member 1	SNF	alpha-helical transmembrane proteins
Q8IYC9_HUMAN	solute carrier family 6 (neurotransmitter transporter, GABA), member 11	SNF	N/A
S6A11_HUMAN	solute carrier family 6 (neurotransmitter transporter, GABA), member 11	SNF	alpha-helical transmembrane proteins
S6A13_HUMAN	solute carrier family 6 (neurotransmitter transporter, GABA), member 13	SNF	alpha-helical transmembrane proteins
SC6A7_HUMAN	solute carrier family 6 (neurotransmitter transporter, L-proline), member 7	SNF	alpha-helical transmembrane proteins
S6A12_HUMAN	solute carrier family 6 (neurotransmitter transporter, betaine/GABA), member 12	SNF	alpha-helical transmembrane proteins
SC6A8_HUMAN	solute carrier family 6 (neurotransmitter transporter, creatine), member 8	SNF	alpha-helical transmembrane proteins
SC6A3_HUMAN	solute carrier family 6 (neurotransmitter transporter, dopamine), member 3	SNF	alpha-helical transmembrane proteins
SC6A5_HUMAN	solute carrier family 6 (neurotransmitter transporter, glycine), member 5	SNF	alpha-helical transmembrane proteins
SC6A9_HUMAN	solute carrier family 6 (neurotransmitter transporter, glycine), member 9	SNF	alpha-helical transmembrane proteins
SC6A2_HUMAN	solute carrier family 6 (neurotransmitter transporter, noradrenalin), member 2	SNF	alpha-helical transmembrane proteins
SC6A4_HUMAN	solute carrier family 6 (neurotransmitter transporter, serotonin), member 4	SNF	alpha-helical transmembrane proteins
SC6A6_HUMAN	solute carrier family 6 (neurotransmitter transporter, taurine), member 6	SNF	alpha-helical transmembrane proteins
S6A15_HUMAN	solute carrier family 6 (neutral amino acid transporter), member 15	SNF	alpha-helical transmembrane proteins
S6A19_HUMAN	solute carrier family 6 (neutral amino acid transporter), member 19	SNF	alpha-helical transmembrane proteins
S6A20_HUMAN	solute carrier family 6 (proline IMINO transporter), member 20	SNF	alpha-helical transmembrane proteins
Q8IYV4_HUMAN	solute carrier family 6, member 16	SNF	N/A
S6A16_HUMAN	solute carrier family 6, member 16	SNF	alpha-helical transmembrane proteins
S6A17_HUMAN	solute carrier family 6, member 17	SNF	alpha-helical transmembrane proteins
S6A18_HUMAN	solute carrier family 6, member 18	SNF	alpha-helical transmembrane proteins
CTR1_HUMAN	solute carrier family 7 (cationic amino acid transporter, y+ system), member 1	AA_permease_C,Ig_3,Ig_2	alpha-helical transmembrane proteins
CTR2_HUMAN	solute carrier family 7 (cationic amino acid transporter, y+ system), member 2	V-set,AA_permease_C	alpha-helical transmembrane proteins
CTR3_HUMAN	solute carrier family 7 (cationic amino acid transporter, y+ system), member 3	V-set,AA_permease_C	alpha-helical transmembrane proteins
NAC1_HUMAN	solute carrier family 8 (sodium/calcium exchanger), member 1	Calx-beta,Na_Ca_ex	alpha-helical transmembrane proteins
SL9A1_HUMAN	solute carrier family 9 (sodium/hydrogen exchanger), member 1	Na_H_Exchanger	alpha-helical transmembrane proteins
SL9A3_HUMAN	solute carrier family 9 (sodium/hydrogen exchanger), member 3	Na_H_Exchanger	alpha-helical transmembrane proteins
SL9A4_HUMAN	solute carrier family 9 (sodium/hydrogen exchanger), member 4	Na_H_Exchanger	alpha-helical transmembrane proteins
SL9A6_HUMAN	solute carrier family 9 (sodium/hydrogen exchanger), member 6	Na_H_Exchanger	alpha-helical transmembrane proteins
SO1A2_HUMAN	solute carrier organic anion transporter family, member 1A2	Kazal_2,OATP	alpha-helical transmembrane proteins
SO1B1_HUMAN	solute carrier organic anion transporter family, member 1B1	Kazal_2,OATP	alpha-helical transmembrane proteins
SO1B3_HUMAN	solute carrier organic anion transporter family, member 1B3	Kazal_2,OATP	alpha-helical transmembrane proteins
Q5JAR4_HUMAN	solute carrier organic anion transporter family, member 1B3	Kazal_2,OATP	N/A
SO2A1_HUMAN	solute carrier organic anion transporter family, member 2A1	Kazal_2,OATP	alpha-helical transmembrane proteins
SO4C1_HUMAN	solute carrier organic anion transporter family, member 4C1	OATP	alpha-helical transmembrane proteins
SHH_HUMAN	sonic hedgehog homolog (Drosophila)	HH_signal,Hint	N/A
SORL_HUMAN	sortilin-related receptor, L(DLR class) A repeats-containing	fn3,Ldl_recept_a,Ldl_recept_b	alpha-helical transmembrane proteins
HYALP_HUMAN	sperm adhesion molecule 1 (PH-20 hyaluronidase, zona pellucida binding)	Glyco_hydro_56	N/A
NSMA_HUMAN	sphingomyelin phosphodiesterase 2, neutral membrane (neutral sphingomyelinase)	Exo_endo_phos	alpha-helical transmembrane proteins
NSMA2_HUMAN	sphingomyelin phosphodiesterase 3, neutral membrane (neutral sphingomyelinase II)	Exo_endo_phos	N/A
STAB1_HUMAN	stabilin 1	EGF_3,Fasciclin,Xlink	alpha-helical transmembrane proteins
STAB2_HUMAN	stabilin 2	EGF_3,Fasciclin,Xlink	alpha-helical transmembrane proteins
STBD1_HUMAN	starch binding domain 1	CBM_20	alpha-helical transmembrane proteins
STS_HUMAN	steroid sulfatase (microsomal), isozyme S	Sulfatase	alpha-helical transmembrane proteins
STOM_HUMAN	stomatin	Band_7	N/A
STML3_HUMAN	stomatin (EPB72)-like 3	Band_7	alpha-helical transmembrane proteins
SERP1_HUMAN	stress-associated endoplasmic reticulum protein 1	RAMP4	alpha-helical transmembrane proteins
SERP2_HUMAN	stress-associated endoplasmic reticulum protein family member 2	RAMP4	alpha-helical transmembrane proteins
CAB45_HUMAN	stromal cell derived factor 4	EF_hand_5	N/A
STIM1_HUMAN	stromal interaction molecule 1	SAM_2	alpha-helical transmembrane proteins
SUIS_HUMAN	sucrase-isomaltase (alpha-glucosidase)	Glyco_hydro_31,Trefoil	alpha-helical transmembrane proteins
ST14_HUMAN	suppression of tumorigenicity 14 (colon carcinoma)	Trypsin,Ldl_recept_a,CUB,SEA	alpha-helical transmembrane proteins
SUSD2_HUMAN	sushi domain containing 2	VWD,Somatomedin_B,Sushi,AMOP	alpha-helical transmembrane proteins
SV2A_HUMAN	synaptic vesicle glycoprotein 2A	MFS_1	alpha-helical transmembrane proteins
SV2B_HUMAN	synaptic vesicle glycoprotein 2B; hypothetical protein LOC100128403	MFS_1	alpha-helical transmembrane proteins
SV2C_HUMAN	synaptic vesicle glycoprotein 2C	MFS_1	alpha-helical transmembrane proteins
SYT1_HUMAN	synaptotagmin I	C2	alpha-helical transmembrane proteins
SYT2_HUMAN	synaptotagmin II	C2	alpha-helical transmembrane proteins
SYT3_HUMAN	synaptotagmin III	C2	alpha-helical transmembrane proteins
SYT4_HUMAN	synaptotagmin IV	C2	alpha-helical transmembrane proteins
SYT9_HUMAN	synaptotagmin IX	C2	alpha-helical transmembrane proteins
SYT5_HUMAN	synaptotagmin V	C2	alpha-helical transmembrane proteins
SYT6_HUMAN	synaptotagmin VI	C2	alpha-helical transmembrane proteins
SYT8_HUMAN	synaptotagmin VIII	C2	alpha-helical transmembrane proteins
SYT10_HUMAN	synaptotagmin X	C2	alpha-helical transmembrane proteins
SYT11_HUMAN	synaptotagmin XI	C2	alpha-helical transmembrane proteins
SYT12_HUMAN	synaptotagmin XII	C2	alpha-helical transmembrane proteins
SYT13_HUMAN	synaptotagmin XIII	C2	alpha-helical transmembrane proteins
SYT15_HUMAN	synaptotagmin XV	C2	alpha-helical transmembrane proteins
SYT7_HUMAN	synaptotagmin XVII; synaptotagmin VII	C2	alpha-helical transmembrane proteins
SDC1_HUMAN	syndecan 1	Syndecan	alpha-helical transmembrane proteins
SDC2_HUMAN	syndecan 2	Syndecan	alpha-helical transmembrane proteins
SDC4_HUMAN	syndecan 4	Syndecan	alpha-helical transmembrane proteins
SDCB1_HUMAN	syndecan binding protein (syntenin)	PDZ	N/A
STX1A_HUMAN	syntaxin 1A (brain)	Syntaxin,SNARE	alpha-helical transmembrane proteins
STX1B_HUMAN	syntaxin 1B	Syntaxin,SNARE	alpha-helical transmembrane proteins
STX2_HUMAN	syntaxin 2	Syntaxin,SNARE	alpha-helical transmembrane proteins
STX3_HUMAN	syntaxin 3	Syntaxin,SNARE	alpha-helical transmembrane proteins
STX4_HUMAN	syntaxin 4	Syntaxin,SNARE	alpha-helical transmembrane proteins
STX6_HUMAN	syntaxin 6	SNARE,Syntaxin-6_N	alpha-helical transmembrane proteins
STX8_HUMAN	syntaxin 8	SNARE	alpha-helical transmembrane proteins
TS1R1_HUMAN	taste receptor, type 1, member 1	NCD3G,ANF_receptor	alpha-helical transmembrane proteins
TS1R2_HUMAN	taste receptor, type 1, member 2	NCD3G,ANF_receptor	alpha-helical transmembrane proteins
TS1R3_HUMAN	taste receptor, type 1, member 3	NCD3G,ANF_receptor	alpha-helical transmembrane proteins
TDGF1_HUMAN	teratocarcinoma-derived growth factor 3, pseudogene; teratocarcinoma-derived growth factor 1	CFC	N/A
TRBM_HUMAN	thrombomodulin	EGF_CA	alpha-helical transmembrane proteins
PERT_HUMAN	thyroid peroxidase	EGF_CA,An_peroxidase	alpha-helical transmembrane proteins
TSHR_HUMAN	thyroid stimulating hormone receptor	LRR_8	alpha-helical transmembrane proteins
TRHDE_HUMAN	thyrotropin-releasing hormone degrading enzyme	Peptidase_M1,DUF3358	alpha-helical transmembrane proteins
TLR1_HUMAN	toll-like receptor 1	LRR_8,TIR	alpha-helical transmembrane proteins
TLR10_HUMAN	toll-like receptor 10	LRR_8,TIR	alpha-helical transmembrane proteins
TLR2_HUMAN	toll-like receptor 2	LRR_8,TIR	alpha-helical transmembrane proteins
TLR3_HUMAN	toll-like receptor 3	LRR_8,TIR	alpha-helical transmembrane proteins
TLR4_HUMAN	toll-like receptor 4	LRR_8,LRR_4,TIR	alpha-helical transmembrane proteins
TLR5_HUMAN	toll-like receptor 5	LRR_8,TIR	alpha-helical transmembrane proteins
TLR6_HUMAN	toll-like receptor 6	LRR_8,TIR	alpha-helical transmembrane proteins
TLR9_HUMAN	toll-like receptor 9	LRR_8,TIR	alpha-helical transmembrane proteins
TFR1_HUMAN	transferrin receptor (p90, CD71)	TFR_dimer,Peptidase_M28,PA	alpha-helical transmembrane proteins
TFR2_HUMAN	transferrin receptor 2	TFR_dimer,Peptidase_M28,PA	alpha-helical transmembrane proteins
Q8WV88_HUMAN	transforming growth factor, beta 3	TGFb_propeptide	N/A
TGFB3_HUMAN	transforming growth factor, beta 3	TGF_beta,TGFb_propeptide	N/A
TGFR1_HUMAN	transforming growth factor, beta receptor 1	TGF_beta_GS,Pkinase,Activin_recp	alpha-helical transmembrane proteins
TGFR2_HUMAN	transforming growth factor, beta receptor II (70/80kDa)	Pkinase	alpha-helical transmembrane proteins
TGBR3_HUMAN	transforming growth factor, beta receptor III	Zona_pellucida	alpha-helical transmembrane proteins
TRPC1_HUMAN	transient receptor potential cation channel, subfamily C, member 1	TRP_2,Ank_2	alpha-helical transmembrane proteins
TRPC3_HUMAN	transient receptor potential cation channel, subfamily C, member 3	TRP_2,Ank_2	alpha-helical transmembrane proteins
TRPC4_HUMAN	transient receptor potential cation channel, subfamily C, member 4	TRP_2,Ank_2	alpha-helical transmembrane proteins
Q3MHB9_HUMAN	transient receptor potential cation channel, subfamily C, member 4	TRP_2,Ank_2	N/A
TRPC5_HUMAN	transient receptor potential cation channel, subfamily C, member 5	TRP_2,Ank_2	alpha-helical transmembrane proteins
TRPC6_HUMAN	transient receptor potential cation channel, subfamily C, member 6	TRP_2,Ank_2	alpha-helical transmembrane proteins
TRPM6_HUMAN	transient receptor potential cation channel, subfamily M, member 6	Alpha_kinase	alpha-helical transmembrane proteins
TRPM7_HUMAN	transient receptor potential cation channel, subfamily M, member 7	Alpha_kinase	alpha-helical transmembrane proteins
TRPV1_HUMAN	transient receptor potential cation channel, subfamily V, member 1	Ank	alpha-helical transmembrane proteins

TRPV2_HUMAN	transient receptor potential cation channel, subfamily V, member 2	Ank_2	alpha-helical transmembrane proteins
TRPV4_HUMAN	transient receptor potential cation channel, subfamily V, member 4	Ank	alpha-helical transmembrane proteins
TRPV5_HUMAN	transient receptor potential cation channel, subfamily V, member 5	Ank_2	alpha-helical transmembrane proteins
TRPV6_HUMAN	transient receptor potential cation channel, subfamily V, member 6	Ank_2	alpha-helical transmembrane proteins
ERG24_HUMAN	transmembrane 7 superfamily member 2	ERG4_ERG24	alpha-helical transmembrane proteins
TM9S2_HUMAN	transmembrane 9 superfamily member 2	EMP70	alpha-helical transmembrane proteins
TMED1_HUMAN	transmembrane emp24 protein transport domain containing 1	EMP24_GP25L	alpha-helical transmembrane proteins
TMED7_HUMAN	transmembrane emp24 protein transport domain containing 7; toll-like receptor adaptor molecule 2	EMP24_GP25L	alpha-helical transmembrane proteins
B5MDI9_HUMAN	transmembrane protease, serine 11A	Trypsin,SEA	N/A
TM11A_HUMAN	transmembrane protease, serine 11A	Trypsin,SEA	alpha-helical transmembrane proteins
TM11D_HUMAN	transmembrane protease, serine 11D	Trypsin,SEA	alpha-helical transmembrane proteins
TM11E_HUMAN	transmembrane protease, serine 11E	Trypsin,SEA	alpha-helical transmembrane proteins
TM11F_HUMAN	transmembrane protease, serine 11F	Trypsin,SEA	alpha-helical transmembrane proteins
TMPS2_HUMAN	transmembrane protease, serine 2	SRCR,Trypsin	alpha-helical transmembrane proteins
TMPS5_HUMAN	transmembrane protease, serine 5	Trypsin	alpha-helical transmembrane proteins
TMPS6_HUMAN	transmembrane protease, serine 6	Trypsin,Ldl_recept_a,SEA	alpha-helical transmembrane proteins
TMPS7_HUMAN	transmembrane protease, serine 7	Trypsin,Ldl_recept_a,CUB,SEA	alpha-helical transmembrane proteins
TMPS9_HUMAN	transmembrane protease, serine 9	Trypsin,Ldl_recept_a	alpha-helical transmembrane proteins
TMM25_HUMAN	transmembrane protein 25	C2-set_2	alpha-helical transmembrane proteins
TMM53_HUMAN	transmembrane protein 53	DUF829	alpha-helical transmembrane proteins
TEFF1_HUMAN	transmembrane protein with EGF-like and two follistatin-like domains 1; chromosome 9 open reading frame 30; hypothetical LOC729538	Kazal_2	alpha-helical transmembrane proteins
TAP1_HUMAN	transporter 1, ATP-binding cassette, sub-family B (MDR/TAP)	ABC_tran	alpha-helical transmembrane proteins
TAP2_HUMAN	transporter 2, ATP-binding cassette, sub-family B (MDR/TAP)	ABC_tran	alpha-helical transmembrane proteins
TRDN_HUMAN	triadin	Asp-B-Hydro_N	alpha-helical transmembrane proteins
TREM1_HUMAN	triggering receptor expressed on myeloid cells 1	V-set	alpha-helical transmembrane proteins
TREM2_HUMAN	triggering receptor expressed on myeloid cells 2	V-set	alpha-helical transmembrane proteins
TRML1_HUMAN	triggering receptor expressed on myeloid cells-like 1	V-set	alpha-helical transmembrane proteins
TRML2_HUMAN	triggering receptor expressed on myeloid cells-like 2 pseudogene; triggering receptor expressed on myeloid cells-like 2	V-set	alpha-helical transmembrane proteins
TRI13_HUMAN	tripartite motif-containing 13	zf-B_box,zf-C3HC4_2	alpha-helical transmembrane proteins
TPBG_HUMAN	trophoblast glycoprotein	LRR_8,LRRNT	alpha-helical transmembrane proteins
TRYG1_HUMAN	tryptase gamma 1	Trypsin	alpha-helical transmembrane proteins
TNFA_HUMAN	tumor necrosis factor (TNF superfamily, member 2)	TNF	alpha-helical transmembrane proteins
TNF10_HUMAN	tumor necrosis factor (ligand) superfamily, member 10	TNF	alpha-helical transmembrane proteins
TNF11_HUMAN	tumor necrosis factor (ligand) superfamily, member 11	TNF	alpha-helical transmembrane proteins
TN13B_HUMAN	tumor necrosis factor (ligand) superfamily, member 13b	TNF	alpha-helical transmembrane proteins
TNF14_HUMAN	tumor necrosis factor (ligand) superfamily, member 14	TNF	alpha-helical transmembrane proteins
TNF15_HUMAN	tumor necrosis factor (ligand) superfamily, member 15	TNF	alpha-helical transmembrane proteins
TNFL4_HUMAN	tumor necrosis factor (ligand) superfamily, member 4	TNF	alpha-helical transmembrane proteins
TNFL8_HUMAN	tumor necrosis factor (ligand) superfamily, member 8	TNF	alpha-helical transmembrane proteins
TR10B_HUMAN	tumor necrosis factor receptor superfamily, member 10b	TNFR_c6,Death	alpha-helical transmembrane proteins
TR10C_HUMAN	tumor necrosis factor receptor superfamily, member 10c, decoy without an intracellular domain	TNFR_c6	N/A
TR13B_HUMAN	tumor necrosis factor receptor superfamily, member 13B	TACI-CRD2	alpha-helical transmembrane proteins
TNR14_HUMAN	tumor necrosis factor receptor superfamily, member 14 (herpesvirus entry mediator)	TNFR_c6	alpha-helical transmembrane proteins
TNR1A_HUMAN	tumor necrosis factor receptor superfamily, member 1A	TNFR_c6,Death	alpha-helical transmembrane proteins
TNR1B_HUMAN	tumor necrosis factor receptor superfamily, member 1B	TNFR_c6	alpha-helical transmembrane proteins
TNR25_HUMAN	tumor necrosis factor receptor superfamily, member 25	Death	alpha-helical transmembrane proteins
TNR4_HUMAN	tumor necrosis factor receptor superfamily, member 4	TNFR_c6	alpha-helical transmembrane proteins
TNR8_HUMAN	tumor necrosis factor receptor superfamily, member 8	TNFR_c6	alpha-helical transmembrane proteins
TNR9_HUMAN	tumor necrosis factor receptor superfamily, member 9	TNFR_c6	alpha-helical transmembrane proteins
TACD2_HUMAN	tumor-associated calcium signal transducer 2	Thyroglobulin_1	alpha-helical transmembrane proteins
TTYH1_HUMAN	tweety homolog 1 (Drosophila)	Tweety	alpha-helical transmembrane proteins
TTYH2_HUMAN	tweety homolog 2 (Drosophila)	Tweety	alpha-helical transmembrane proteins
TTYH3_HUMAN	tweety homolog 3 (Drosophila)	Tweety	alpha-helical transmembrane proteins
TIE1_HUMAN	tyrosine kinase with immunoglobulin-like and EGF-like domains 1	Pkinase_Tyr,fn3	alpha-helical transmembrane proteins
UNC5A_HUMAN	unc-5 homolog A (C. elegans)	ZU5,Death,I-set	alpha-helical transmembrane proteins
UROL1_HUMAN	uromodulin-like 1	EGF_CA,EMI,SEA,Zona_pellucida,WAP	alpha-helical transmembrane proteins
ERBB4_HUMAN	v-erb-a erythroblastic leukemia viral oncogene homolog 4 (avian)	Pkinase_Tyr,Furin-like,Recep_L_domain	N/A
ERBB2_HUMAN	v-erb-b2 erythroblastic leukemia viral oncogene homolog 2, neuro/glioblastoma derived oncogene homolog (avian)	Pkinase_Tyr,Furin-like,Recep_L_domain	alpha-helical transmembrane proteins
ERBB3_HUMAN	v-erb-b2 erythroblastic leukemia viral oncogene homolog 3 (avian)	Pkinase_Tyr,Furin-like,Recep_L_domain	alpha-helical transmembrane proteins
VNN2_HUMAN	vanin 2	CN_hydrolase	N/A
VNN3_HUMAN	vanin 3	CN_hydrolase	N/A
VCAM1_HUMAN	vascular cell adhesion molecule 1	C2-set,I-set,Ig_2	alpha-helical transmembrane proteins
VIPR1_HUMAN	vasoactive intestinal peptide receptor 1	HRM	alpha-helical transmembrane proteins
VIPR2_HUMAN	vasoactive intestinal peptide receptor 2	HRM	alpha-helical transmembrane proteins
VLDLR_HUMAN	very low density lipoprotein receptor	EGF_CA,Ldl_recept_a,Ldl_recept_b	alpha-helical transmembrane proteins
VAMP1_HUMAN	vesicle-associated membrane protein 1 (synaptobrevin 1)	Synaptobrevin	alpha-helical transmembrane proteins
VAMP2_HUMAN	vesicle-associated membrane protein 2 (synaptobrevin 2)	Synaptobrevin	alpha-helical transmembrane proteins
VAMP3_HUMAN	vesicle-associated membrane protein 3 (cellubrevin)	Synaptobrevin	alpha-helical transmembrane proteins
VAMP5_HUMAN	vesicle-associated membrane protein 5 (myobrevin)	Synaptobrevin	alpha-helical transmembrane proteins
ZACN_HUMAN	zinc activated ligand-gated ion channel	Neur_chan_LBD	alpha-helical transmembrane proteins
ZP1_HUMAN	zona pellucida glycoprotein 1 (sperm receptor)	RRM_1,Zona_pellucida	alpha-helical transmembrane proteins
ZP2_HUMAN	zona pellucida glycoprotein 2 (sperm receptor)	Alpha-amylase,Zona_pellucida	alpha-helical transmembrane proteins
ZP3_HUMAN	zona pellucida glycoprotein 3 (sperm receptor)	zf-B_box,zf-C3HC4_2,Zona_pellucida	alpha-helical transmembrane proteins
ZP4_HUMAN	zona pellucida glycoprotein 4	Trefoil,KRAB,Zona_pellucida	alpha-helical transmembrane proteins
ZAN_HUMAN	zonadhesin	TIL,VWD,C8,TILa,MAM	alpha-helical transmembrane proteins
GPAT3_HUMAN	1-acylglycerol-3-phosphate O-acyltransferase 9	Acyltransferase	alpha-helical transmembrane proteins
DHC24_HUMAN	24-dehydrocholesterol reductase	FAD_binding_4	alpha-helical transmembrane proteins
HMDH_HUMAN	3-hydroxy-3-methylglutaryl-Coenzyme A reductase	HMG-CoA_red	alpha-helical transmembrane proteins
KDSR_HUMAN	3-ketodihydrosphingosine reductase	adh_short	alpha-helical transmembrane proteins
DHCR7_HUMAN	7-dehydrocholesterol reductase	ERG4_ERG24	alpha-helical transmembrane proteins
AT2A1_HUMAN	ATPase, Ca++ transporting, cardiac muscle, fast twitch 1	Cation_ATPase_N,E1-E2_ATPase	alpha-helical transmembrane proteins
AT2A2_HUMAN	ATPase, Ca++ transporting, cardiac muscle, slow twitch 2	E1-E2_ATPase,Cation_ATPase_N	alpha-helical transmembrane proteins
BCL2_HUMAN	B-cell CLL/lymphoma 2	Bcl-2,BH4	alpha-helical transmembrane proteins
BAP31_HUMAN	B-cell receptor-associated protein 31	Bap31	alpha-helical transmembrane proteins
BAX_HUMAN	BCL2-associated X protein	Bcl-2	alpha-helical transmembrane proteins
SEC20_HUMAN	BCL2/adenovirus E1B 19kDa interacting protein 1	Sec20	alpha-helical transmembrane proteins
BSCL2_HUMAN	Bernardinelli-Seip congenital lipodystrophy 2 (seipin)	Seipin	alpha-helical transmembrane proteins
CD4_HUMAN	CD4 molecule	C2-set,RabGAP-TBC,ABC_tran,HECT,V-set,ABC_membrane_2,C2,PH	alpha-helical transmembrane proteins
DERL1_HUMAN	Der1-like domain family, member 1	DER1	alpha-helical transmembrane proteins
DERL2_HUMAN	Der1-like domain family, member 2	DER1	alpha-helical transmembrane proteins
DERL3_HUMAN	Der1-like domain family, member 3	DER1	alpha-helical transmembrane proteins
EDEM1_HUMAN	ER degradation enhancer, mannosidase alpha-like 1	Glyco_hydro_47	alpha-helical transmembrane proteins
ERLN1_HUMAN	ER lipid raft associated 1	Band_7	alpha-helical transmembrane proteins
FKBP8_HUMAN	FK506 binding protein 8, 38kDa	TPR_16,FKBP_C	alpha-helical transmembrane proteins
HTRA2_HUMAN	HtrA serine peptidase 2	PDZ_2,Trypsin_2	alpha-helical transmembrane proteins
NSDHL_HUMAN	NAD(P) dependent steroid dehydrogenase-like	3Beta_HSD	alpha-helical transmembrane proteins
ORML1_HUMAN	ORM1-like 1 (S. cerevisiae)	ORMDL	alpha-helical transmembrane proteins
SAC1_HUMAN	SAC1 suppressor of actin mutations 1-like (yeast)	Syja_N	alpha-helical transmembrane proteins
SC11A_HUMAN	SEC11 homolog A (S. cerevisiae)	Peptidase_S24	alpha-helical transmembrane proteins
SC11C_HUMAN	SEC11 homolog C (S. cerevisiae)	Peptidase_S24	alpha-helical transmembrane proteins
SC22A_HUMAN	SEC22 vesicle trafficking protein homolog A (S. cerevisiae)	Longin	alpha-helical transmembrane proteins
TPSN_HUMAN	TAP binding protein (tapasin)	C1-set	alpha-helical transmembrane proteins
VAPA_HUMAN	VAMP (vesicle-associated membrane protein)-associated protein A, 33kDa	Motile_Sperm	alpha-helical transmembrane proteins
VAPB_HUMAN	VAMP (vesicle-associated membrane protein)-associated protein B and C	Motile_Sperm	alpha-helical transmembrane proteins
AWAT2_HUMAN	acyl-CoA wax alcohol acyltransferase 2	DAGAT	alpha-helical transmembrane proteins
ACER1_HUMAN	alkaline ceramidase 1	Ceramidase	alpha-helical transmembrane proteins
ACER3_HUMAN	alkaline ceramidase 3	Ceramidase	alpha-helical transmembrane proteins
APOL2_HUMAN	apolipoprotein L, 2	ApoL	N/A
AAAD_HUMAN	arylacetamide deacetylase (esterase)	Abhydrolase_3	alpha-helical transmembrane proteins
ALG12_HUMAN	asparagine-linked glycosylation 12, alpha-1,6-mannosyltransferase homolog (S. cerevisiae)	Glyco_transf_22	alpha-helical transmembrane proteins
ALG6_HUMAN	asparagine-linked glycosylation 6, alpha-1,3-glucosyltransferase homolog (S. cerevisiae)	Alg6_Alg8	alpha-helical transmembrane proteins

	ALG8_HUMAN	asparagine-linked glycosylation 8, alpha-1,3-glucosyltransferase homolog (S. cerevisiae)	Alg6_Alg8	alpha-helical transmembrane proteins
	ALG9_HUMAN	asparagine-linked glycosylation 9, alpha-1,2-mannosyltransferase homolog (S. cerevisiae)	Glyco_transf_22	alpha-helical transmembrane proteins
	ASPH_HUMAN	aspartate beta-hydroxylase	Asp_Arg_Hydrox,TPR_16,Asp-B-Hydro_N	alpha-helical transmembrane proteins
	ATLA1_HUMAN	atlastin GTPase 1	GBP	alpha-helical transmembrane proteins
	CSTN2_HUMAN	calsyntenin 2	Cadherin,Laminin_G_3	alpha-helical transmembrane proteins
	CSTN3_HUMAN	calsyntenin 3	Laminin_G_3	alpha-helical transmembrane proteins
	Q9UBD8_HUMAN	ceroid-lipofuscinosis, neuronal 3	CLN3	N/A
	CLN3_HUMAN	ceroid-lipofuscinosis, neuronal 3	PKD_channel,CLN3	alpha-helical transmembrane proteins
	Q2TA70_HUMAN	ceroid-lipofuscinosis, neuronal 3	CLN3	N/A
	CEPT1_HUMAN	choline/ethanolamine phosphotransferase 1	CDP-OH_P_transf	alpha-helical transmembrane proteins
	CFTR_HUMAN	cystic fibrosis transmembrane conductance regulator (ATP-binding cassette sub-family C, member 7)	ABC_tran	alpha-helical transmembrane proteins
	CP1A2_HUMAN	cytochrome P450, family 1, subfamily A, polypeptide 2	p450	N/A
	CP2AD_HUMAN	cytochrome P450, family 2, subfamily A, polypeptide 13	p450	N/A
	CP2A6_HUMAN	cytochrome P450, family 2, subfamily A, polypeptide 6	p450	N/A
	CP2C8_HUMAN	cytochrome P450, family 2, subfamily C, polypeptide 8	p450	N/A
	CP2E1_HUMAN	cytochrome P450, family 2, subfamily E, polypeptide 1	p450	N/A
	CP2F1_HUMAN	cytochrome P450, family 2, subfamily F, polypeptide 1	p450	N/A
	Q32MN5_HUMAN	cytochrome P450, family 2, subfamily F, polypeptide 1	p450	N/A
	CP2R1_HUMAN	cytochrome P450, family 2, subfamily R, polypeptide 1	p450	N/A
	CP2S1_HUMAN	cytochrome P450, family 2, subfamily S, polypeptide 1	p450	N/A
	CP2U1_HUMAN	cytochrome P450, family 2, subfamily U, polypeptide 1	p450	alpha-helical transmembrane proteins
	CP26A_HUMAN	cytochrome P450, family 26, subfamily A, polypeptide 1	p450	N/A
	CP26C_HUMAN	cytochrome P450, family 26, subfamily C, polypeptide 1	p450	alpha-helical transmembrane proteins
	CP3A4_HUMAN	cytochrome P450, family 3, subfamily A, polypeptide 4	p450	alpha-helical transmembrane proteins
	CP3A5_HUMAN	cytochrome P450, family 3, subfamily A, polypeptide 5	p450	N/A
	CP3A7_HUMAN	cytochrome P450, family 3, subfamily A, polypeptide 7	p450	N/A
	CP39A_HUMAN	cytochrome P450, family 39, subfamily A, polypeptide 1	p450	N/A
	CP4AB_HUMAN	cytochrome P450, family 4, subfamily A, polypeptide 11	p450	N/A
	Q8IZB0_HUMAN	cytochrome P450, family 4, subfamily B, polypeptide 1	p450	N/A
	CP4B1_HUMAN	cytochrome P450, family 4, subfamily B, polypeptide 1	p450	N/A
	CP4FB_HUMAN	cytochrome P450, family 4, subfamily F, polypeptide 11	p450	alpha-helical transmembrane proteins
	CP4F2_HUMAN	cytochrome P450, family 4, subfamily F, polypeptide 2	p450	N/A
	CP4F3_HUMAN	cytochrome P450, family 4, subfamily F, polypeptide 3	p450	alpha-helical transmembrane proteins
	CP51A_HUMAN	cytochrome P450, family 51, subfamily A, polypeptide 1	p450	alpha-helical transmembrane proteins
	CP7A1_HUMAN	cytochrome P450, family 7, subfamily A, polypeptide 1	p450	N/A
	CP7B1_HUMAN	cytochrome P450, family 7, subfamily B, polypeptide 1	p450	N/A
	CP8B1_HUMAN	cytochrome P450, family 8, subfamily B, polypeptide 1	p450	alpha-helical transmembrane proteins
	DAD1_HUMAN	defender against cell death 1	DAD	alpha-helical transmembrane proteins
	DEGS2_HUMAN	degenerative spermatocyte homolog 2, lipid desaturase (Drosophila)	Lipid_DES,FA_desaturase	alpha-helical transmembrane proteins
	EDA_HUMAN	ectodysplasin A	Collagen,EMP24_GP25L,TNF	alpha-helical transmembrane proteins
	ERN1_HUMAN	endoplasmic reticulum to nucleus signaling 1	HECT,Pkinase,Ribonuc_2-5A	alpha-helical transmembrane proteins
	E2AK3_HUMAN	eukaryotic translation initiation factor 2-alpha kinase 3	Pkinase	alpha-helical transmembrane proteins
ER membrane	EXT1_HUMAN	exostoses (multiple) 1	Exostosin,Glyco_transf_64	alpha-helical transmembrane proteins
	EXT2_HUMAN	exostoses (multiple) 2	Exostosin,Glyco_transf_64	alpha-helical transmembrane proteins
	EXTL1_HUMAN	exostoses (multiple)-like 1	Exostosin,Glyco_transf_64	alpha-helical transmembrane proteins
	EXTL2_HUMAN	exostoses (multiple)-like 2	Glyco_transf_64	alpha-helical transmembrane proteins
	EXTL3_HUMAN	exostoses (multiple)-like 3	Exostosin,Glyco_transf_64	alpha-helical transmembrane proteins
	FMO3_HUMAN	flavin containing monooxygenase 3	FMO-like	N/A
	FMO4_HUMAN	flavin containing monooxygenase 4	FMO-like	alpha-helical transmembrane proteins
	FMO5_HUMAN	flavin containing monooxygenase 5	FMO-like	N/A
	FURIN_HUMAN	furin (paired basic amino acid cleaving enzyme)	P_proprotein,Peptidase_S8	alpha-helical transmembrane proteins
	GOSR2_HUMAN	golgi SNAP receptor complex member 2	V-SNARE_C	alpha-helical transmembrane proteins
	Q5TDG2_HUMAN	hydroxy-delta-5-steroid dehydrogenase, 3 beta- and steroid delta-isomerase 1	3Beta_HSD	N/A
	3BHS1_HUMAN	hydroxy-delta-5-steroid dehydrogenase, 3 beta- and steroid delta-isomerase 1	3Beta_HSD	alpha-helical transmembrane proteins
	3BHS2_HUMAN	hydroxy-delta-5-steroid dehydrogenase, 3 beta- and steroid delta-isomerase 2	3Beta_HSD	alpha-helical transmembrane proteins
	DHI1_HUMAN	hydroxysteroid (11-beta) dehydrogenase 1	adh_short	alpha-helical transmembrane proteins
	DHB2_HUMAN	hydroxysteroid (17-beta) dehydrogenase 2	Peptidase_S74,MRF_C2,MRF_C1,adh_short	alpha-helical transmembrane proteins
	ITPR1_HUMAN	inositol 1,4,5-triphosphate receptor, type 1	RIH_assoc,RYDR_ITPR,MIR,Ins145_P3_rec	alpha-helical transmembrane proteins
	ITPR2_HUMAN	inositol 1,4,5-triphosphate receptor, type 2	RIH_assoc,RYDR_ITPR,MIR,Ins145_P3_rec	alpha-helical transmembrane proteins
	JPH1_HUMAN	junctophilin 1	MORN	alpha-helical transmembrane proteins
	JPH2_HUMAN	junctophilin 2	MORN	alpha-helical transmembrane proteins
	JPH3_HUMAN	junctophilin 3	MORN	alpha-helical transmembrane proteins
	LMA2L_HUMAN	lectin, mannose-binding 2-like	Lectin_leg-like	alpha-helical transmembrane proteins
	LMAN1_HUMAN	lectin, mannose-binding, 1	Lectin_leg-like	alpha-helical transmembrane proteins
	LRIT1_HUMAN	leucine-rich repeat, immunoglobulin-like and transmembrane domains 1	LRR_8,I-set,fn3	alpha-helical transmembrane proteins
	LRMP_HUMAN	lymphoid-restricted membrane protein	MRVI1	alpha-helical transmembrane proteins
	MPU1_HUMAN	mannose-P-dolichol utilization defect 1	PQ-loop	alpha-helical transmembrane proteins
	MBOA4_HUMAN	membrane bound O-acyltransferase domain containing 4	MBOAT	alpha-helical transmembrane proteins
	MRVI1_HUMAN	murine retrovirus integration site 1 homolog	MRVI1	alpha-helical transmembrane proteins
	OS9_HUMAN	osteosarcoma amplified 9, endoplasmic reticulum associated protein	PRKCSH	N/A
	PLPL6_HUMAN	patatin-like phospholipase domain containing 6	cNMP_binding,Patatin	alpha-helical transmembrane proteins
	B3KUV7_HUMAN	phosphatidylinositol glycan anchor biosynthesis, class A	Glycos_transf_1	N/A
	PIGA_HUMAN	phosphatidylinositol glycan anchor biosynthesis, class A	Glycos_transf_1	alpha-helical transmembrane proteins
	PIGB_HUMAN	phosphatidylinositol glycan anchor biosynthesis, class B	Glyco_transf_22	alpha-helical transmembrane proteins
	PIGF_HUMAN	phosphatidylinositol glycan anchor biosynthesis, class F	PIG-F	alpha-helical transmembrane proteins
	PIGG_HUMAN	phosphatidylinositol glycan anchor biosynthesis, class G	Phosphodiest	alpha-helical transmembrane proteins
	GPI8_HUMAN	phosphatidylinositol glycan anchor biosynthesis, class K	Peptidase_C13	alpha-helical transmembrane proteins
	PIGN_HUMAN	phosphatidylinositol glycan anchor biosynthesis, class N	Phosphodiest	alpha-helical transmembrane proteins
	PIGO_HUMAN	phosphatidylinositol glycan anchor biosynthesis, class O	Phosphodiest	alpha-helical transmembrane proteins
	PIGT_HUMAN	phosphatidylinositol glycan anchor biosynthesis, class T	Gpi16	alpha-helical transmembrane proteins
	PIGV_HUMAN	phosphatidylinositol glycan anchor biosynthesis, class V	Mannosyl_trans2	alpha-helical transmembrane proteins
	PIGX_HUMAN	phosphatidylinositol glycan anchor biosynthesis, class X	PIG-X	alpha-helical transmembrane proteins
	PIGZ_HUMAN	phosphatidylinositol glycan anchor biosynthesis, class Z	Glyco_transf_22	alpha-helical transmembrane proteins
	PORCN_HUMAN	porcupine homolog (Drosophila)	MBOAT	alpha-helical transmembrane proteins
	B7ZAR3_HUMAN	porcupine homolog (Drosophila)	MBOAT	N/A
	PGAP1_HUMAN	post-GPI attachment to proteins 1	PGAP1	alpha-helical transmembrane proteins
	PGAP3_HUMAN	post-GPI attachment to proteins 3	Per1	alpha-helical transmembrane proteins
	POMT1_HUMAN	protein-O-mannosyltransferase 1	MIR	alpha-helical transmembrane proteins
	RETST_HUMAN	retinol saturase (all-trans-retinol 13,14-reductase)	NAD_binding_8	N/A
	RPN1_HUMAN	ribophorin I	Ribophorin_I	alpha-helical transmembrane proteins
	Q5JYR7_HUMAN	ribophorin II	Ribophorin_II	N/A
	RPN2_HUMAN	ribophorin II	Ribophorin_II	alpha-helical transmembrane proteins
	RN180_HUMAN	ring finger protein 180	zf-C3HC4_2	alpha-helical transmembrane proteins
	RYR1_HUMAN	ryanodine receptor 1 (skeletal)	SPRY,RR_TM4-6,Ins145_P3_rec,RYDR_ITPR,RIH_assoc,EF_hand_6,MIR,RyR	alpha-helical transmembrane proteins
	RYR2_HUMAN	ryanodine receptor 2 (cardiac)	SPRY,RR_TM4-6,Ins145_P3_rec,RIH_assoc,RYDR_ITPR,MIR,RyR	alpha-helical transmembrane proteins
	RYR3_HUMAN	ryanodine receptor 3	SPRY,RR_TM4-6,Ins145_P3_rec,RYDR_ITPR,RIH_assoc,MIR,RyR	alpha-helical transmembrane proteins
	SERC1_HUMAN	serine incorporator 1	Serinc	alpha-helical transmembrane proteins
	SPTC1_HUMAN	serine palmitoyltransferase, long chain base subunit 1	Aminotran_1_2	alpha-helical transmembrane proteins
	SPCS2_HUMAN	signal peptidase complex subunit 2 homolog (S. cerevisiae); signal peptidase complex subunit 2 homolog pseudogene	SPC25	alpha-helical transmembrane proteins
	SSRB_HUMAN	signal sequence receptor, beta (translocon-associated protein beta)	TRAP_beta	alpha-helical transmembrane proteins
	ACER2_HUMAN	similar to alkaline ceramidase 2; alkaline ceramidase 2	Ceramidase	alpha-helical transmembrane proteins
	CP4FC_HUMAN	similar to cytochrome P450, family 4, subfamily F, polypeptide 12; cytochrome P450, family 4, subfamily F, polypeptide 12	p450	alpha-helical transmembrane proteins
	S27A5_HUMAN	solute carrier family 27 (fatty acid transporter), member 5	AMP-binding	alpha-helical transmembrane proteins
	G6PT1_HUMAN	solute carrier family 37 (glucose-6-phosphate transporter), member 4	MFS_1	alpha-helical transmembrane proteins
	SL9A6_HUMAN	solute carrier family 9 (sodium/hydrogen exchanger), member 6	Na_H_Exchanger	alpha-helical transmembrane proteins
	ERG1_HUMAN	squalene epoxidase	SE	alpha-helical transmembrane proteins
	STS_HUMAN	steroid sulfatase (microsomal), isozyme S	Sulfatase	alpha-helical transmembrane proteins
	SOAT1_HUMAN	sterol O-acyltransferase 1	MBOAT	alpha-helical transmembrane proteins
	SOAT2_HUMAN	sterol O-acyltransferase 2	MBOAT	alpha-helical transmembrane proteins
	STIM1_HUMAN	stromal interaction molecule 1	SAM_2	alpha-helical transmembrane proteins

	TMX1_HUMAN	thioredoxin-related transmembrane protein 1	Thioredoxin	alpha-helical transmembrane proteins
	TRAM1_HUMAN	translocation associated membrane protein 1	TRAM_LAG1_CLN8	alpha-helical transmembrane proteins
	TRAM2_HUMAN	translocation associated membrane protein 2	TRAM_LAG1_CLN8	alpha-helical transmembrane proteins
	ERG24_HUMAN	transmembrane 7 superfamily member 2	ERG4_ERG24	alpha-helical transmembrane proteins
	TMPS3_HUMAN	transmembrane protease, serine 3	SRCR,Trypsin,Ldl_recept_a	alpha-helical transmembrane proteins
	TM38A_HUMAN	transmembrane protein 38A	TRIC	alpha-helical transmembrane proteins
	TM38B_HUMAN	transmembrane protein 38B	TRIC	alpha-helical transmembrane proteins
	TUSC3_HUMAN	tumor suppressor candidate 3	Thioredoxin	alpha-helical transmembrane proteins
	VAMP7_HUMAN	vesicle-associated membrane protein 7	Synaptobrevin,Longin	alpha-helical transmembrane proteins
	BGAT_HUMAN	ABO blood group (transferase A, alpha 1-3-N-acetylgalactosaminyltransferase; transferase B, alpha 1-3-galactosyltransferase)	Glyco_transf_6	alpha-helical transmembrane proteins
	MDR3_HUMAN	ATP-binding cassette, sub-family B (MDR/TAP), member 4	ABC_tran	alpha-helical transmembrane proteins
	AT2C1_HUMAN	ATPase, Ca++ transporting, type 2C, member 1	Cation_ATPase_N,E1-E2_ATPase	alpha-helical transmembrane proteins
	BAP31_HUMAN	B-cell receptor-associated protein 31	Bap31	alpha-helical transmembrane proteins
	LFNG_HUMAN	LFNG O-fucosylpeptide 3-beta-N-acetylglucosaminyltransferase	Fringe	alpha-helical transmembrane proteins
	RFNG_HUMAN	RFNG O-fucosylpeptide 3-beta-N-acetylglucosaminyltransferase	Fringe	alpha-helical transmembrane proteins
	SIA4A_HUMAN	ST3 beta-galactoside alpha-2,3-sialyltransferase 1	Glyco_transf_29	alpha-helical transmembrane proteins
	SIA4B_HUMAN	ST3 beta-galactoside alpha-2,3-sialyltransferase 2	Glyco_transf_29	alpha-helical transmembrane proteins
	SIAT6_HUMAN	ST3 beta-galactoside alpha-2,3-sialyltransferase 3	Glyco_transf_29	alpha-helical transmembrane proteins
	SIA4C_HUMAN	ST3 beta-galactoside alpha-2,3-sialyltransferase 4	Glyco_transf_29	alpha-helical transmembrane proteins
	SIAT9_HUMAN	ST3 beta-galactoside alpha-2,3-sialyltransferase 5	Glyco_transf_29	alpha-helical transmembrane proteins
	SIA10_HUMAN	ST3 beta-galactoside alpha-2,3-sialyltransferase 6	Glyco_transf_29	alpha-helical transmembrane proteins
	SIA7A_HUMAN	ST6 (alpha-N-acetyl-neuraminyl-2,3-beta-galactosyl-1,3)-N-acetylgalactosaminide alpha-2,6-sialyltransferase 1	Glyco_transf_29	alpha-helical transmembrane proteins
	SIA7B_HUMAN	ST6 (alpha-N-acetyl-neuraminyl-2,3-beta-galactosyl-1,3)-N-acetylgalactosaminide alpha-2,6-sialyltransferase 2	Glyco_transf_29	alpha-helical transmembrane proteins
	SIA7C_HUMAN	ST6 (alpha-N-acetyl-neuraminyl-2,3-beta-galactosyl-1,3)-N-acetylgalactosaminide alpha-2,6-sialyltransferase 3	Glyco_transf_29	alpha-helical transmembrane proteins
	SIA7D_HUMAN	ST6 (alpha-N-acetyl-neuraminyl-2,3-beta-galactosyl-1,3)-N-acetylgalactosaminide alpha-2,6-sialyltransferase 4	Glyco_transf_29	alpha-helical transmembrane proteins
	SIA7E_HUMAN	ST6 (alpha-N-acetyl-neuraminyl-2,3-beta-galactosyl-1,3)-N-acetylgalactosaminide alpha-2,6-sialyltransferase 5	Glyco_transf_29	alpha-helical transmembrane proteins
	SIA7F_HUMAN	ST6 (alpha-N-acetyl-neuraminyl-2,3-beta-galactosyl-1,3)-N-acetylgalactosaminide alpha-2,6-sialyltransferase 6	Glyco_transf_29	alpha-helical transmembrane proteins
	SIAT2_HUMAN	ST6 beta-galactosamide alpha-2,6-sialyltranferase 2	Glyco_transf_29	alpha-helical transmembrane proteins
	SIA8A_HUMAN	ST8 alpha-N-acetyl-neuraminide alpha-2,8-sialyltransferase 1	Glyco_transf_29	alpha-helical transmembrane proteins
	SIA8B_HUMAN	ST8 alpha-N-acetyl-neuraminide alpha-2,8-sialyltransferase 2	Glyco_transf_29	alpha-helical transmembrane proteins
	SIA8C_HUMAN	ST8 alpha-N-acetyl-neuraminide alpha-2,8-sialyltransferase 3	Glyco_transf_29	alpha-helical transmembrane proteins
	SIA8E_HUMAN	ST8 alpha-N-acetyl-neuraminide alpha-2,8-sialyltransferase 5	Glyco_transf_29	alpha-helical transmembrane proteins
	SIA8F_HUMAN	ST8 alpha-N-acetyl-neuraminide alpha-2,8-sialyltransferase 6	Glyco_transf_29	alpha-helical transmembrane proteins
	TPSN_HUMAN	TAP binding protein (tapasin)	C1-set	alpha-helical transmembrane proteins
	B3GT1_HUMAN	UDP-Gal:betaGlcNAc beta 1,3-galactosyltransferase, polypeptide 1	Galactosyl_T	alpha-helical transmembrane proteins
	B3GN1_HUMAN	UDP-GlcNAc:betaGal beta-1,3-N-acetylglucosaminyltransferase 1; UDP-GlcNAc:betaGal beta-1,3-N-acetylglucosaminyltransferase 2	Glyco_transf_49	alpha-helical transmembrane proteins
	B3GN2_HUMAN	UDP-GlcNAc:betaGal beta-1,3-N-acetylglucosaminyltransferase 1; UDP-GlcNAc:betaGal beta-1,3-N-acetylglucosaminyltransferase 2	Galactosyl_T	alpha-helical transmembrane proteins
	GALT2_HUMAN	UDP-N-acetyl-alpha-D-galactosamine:polypeptide N-acetylgalactosaminyltransferase 2 (GalNAc-T2)	Ricin_B_lectin,Glycos_transf_2	alpha-helical transmembrane proteins
	ACER1_HUMAN	alkaline ceramidase 1	Ceramidase	alpha-helical transmembrane proteins
	ACER3_HUMAN	alkaline ceramidase 3	Ceramidase	alpha-helical transmembrane proteins
	BASI_HUMAN	basigin (Ok blood group)	I-set	alpha-helical transmembrane proteins
	B4GN1_HUMAN	beta-1,4-N-acetyl-galactosaminyl transferase 1	Glycos_transf_2	alpha-helical transmembrane proteins
	B4GN2_HUMAN	beta-1,4-N-acetyl-galactosaminyl transferase 2	Glycos_transf_2	alpha-helical transmembrane proteins
	B4GN3_HUMAN	beta-1,4-N-acetyl-galactosaminyl transferase 3	PA14,CHGN	alpha-helical transmembrane proteins
	B4GN4_HUMAN	beta-1,4-N-acetyl-galactosaminyl transferase 4	PA14,CHGN	alpha-helical transmembrane proteins
	Q5SR54_HUMAN	calsyntenin 1	Cadherin,Laminin_G_3	N/A
	CSTN1_HUMAN	calsyntenin 1	Cadherin,Laminin_G_3	alpha-helical transmembrane proteins
	CSTN2_HUMAN	calsyntenin 2	Cadherin,Laminin_G_3	alpha-helical transmembrane proteins
	CSTN3_HUMAN	calsyntenin 3	Laminin_G_3	alpha-helical transmembrane proteins
	CHST4_HUMAN	carbohydrate (N-acetylglucosamine 6-O) sulfotransferase 4	Sulfotransfer_1	alpha-helical transmembrane proteins
	CHST7_HUMAN	carbohydrate (N-acetylglucosamine 6-O) sulfotransferase 7	Sulfotransfer_1	alpha-helical transmembrane proteins
	CHST2_HUMAN	carbohydrate (N-acetylglucosamine-6-O) sulfotransferase 2	Sulfotransfer_1	alpha-helical transmembrane proteins
	CHSTC_HUMAN	carbohydrate (chondroitin 4) sulfotransferase 12	Sulfotransfer_2	alpha-helical transmembrane proteins
	CHST3_HUMAN	carbohydrate (chondroitin 6) sulfotransferase 3	Sulfotransfer_1	alpha-helical transmembrane proteins
	CHST1_HUMAN	carbohydrate (keratan sulfate Gal-6) sulfotransferase 1	Sulfotransfer_1	alpha-helical transmembrane proteins
	Q9UBD8_HUMAN	ceroid-lipofuscinosis, neuronal 3	CLN3	N/A
	CLN3_HUMAN	ceroid-lipofuscinosis, neuronal 3	PKD_channel,CLN3	alpha-helical transmembrane proteins
	Q2TA70_HUMAN	ceroid-lipofuscinosis, neuronal 3	CLN3	N/A
	CGAT1_HUMAN	chondroitin sulfate N-acetylgalactosaminyltransferase 1	CHGN	alpha-helical transmembrane proteins
	CGAT2_HUMAN	chondroitin sulfate N-acetylgalactosaminyltransferase 2; novel protein similar to chondroitin sulfate GalNAcT-2 (GALNACT-2)	CHGN	alpha-helical transmembrane proteins
	CHSS1_HUMAN	chondroitin sulfate synthase 1	CHGN	alpha-helical transmembrane proteins
	CHSS3_HUMAN	chondroitin sulfate synthase 3	CHGN	alpha-helical transmembrane proteins
	CP2E1_HUMAN	cytochrome P450, family 2, subfamily E, polypeptide 1	p450	N/A
Golgi membrane	Q8NE73_HUMAN	ectonucleoside triphosphate diphosphohydrolase 4	GDA1_CD39	N/A
	ENTP4_HUMAN	ectonucleoside triphosphate diphosphohydrolase 4	GDA1_CD39	alpha-helical transmembrane proteins
	EXT1_HUMAN	exostoses (multiple) 1	Exostosin,Glyco_transf_64	alpha-helical transmembrane proteins
	FUT10_HUMAN	fucosyltransferase 10 (alpha (1,3) fucosyltransferase)	Glyco_transf_10	alpha-helical transmembrane proteins
	FURIN_HUMAN	furin (paired basic amino acid cleaving enzyme)	P_proprotein,Peptidase_S8	alpha-helical transmembrane proteins
	GOSR1_HUMAN	golgi SNAP receptor complex member 1	V-SNARE_C	alpha-helical transmembrane proteins
	GOSR2_HUMAN	golgi SNAP receptor complex member 2	V-SNARE_C	alpha-helical transmembrane proteins
	GSLG1_HUMAN	golgi apparatus protein 1	Cys_rich_FGFR	alpha-helical transmembrane proteins
	GOGA5_HUMAN	golgi autoantigen, golgin subfamily a, 5	Golgin_A5	alpha-helical transmembrane proteins
	BET1_HUMAN	hypothetical protein LOC100128542; blocked early in transport 1 homolog (S. cerevisiae)	SNARE	alpha-helical transmembrane proteins
	LMAN1_HUMAN	lectin, mannose-binding, 1	Lectin_leg-like	alpha-helical transmembrane proteins
	LARGE_HUMAN	like-glycosyltransferase	Glyco_transf_8,Glyco_transf_49	alpha-helical transmembrane proteins
	Q5SRN5_HUMAN	major histocompatibility complex, class I, A	C1-set,MHC_I	N/A
	1A80_HUMAN	major histocompatibility complex, class I, A	C1-set,MHC_I	alpha-helical transmembrane proteins
	1A32_HUMAN	major histocompatibility complex, class I, A	C1-set,MHC_I	alpha-helical transmembrane proteins
	1A26_HUMAN	major histocompatibility complex, class I, A	C1-set,MHC_I	alpha-helical transmembrane proteins
	1A66_HUMAN	major histocompatibility complex, class I, A	C1-set,MHC_I	alpha-helical transmembrane proteins
	1A31_HUMAN	major histocompatibility complex, class I, A	C1-set,MHC_I	alpha-helical transmembrane proteins
	1A25_HUMAN	major histocompatibility complex, class I, A	C1-set,MHC_I	alpha-helical transmembrane proteins
	1A68_HUMAN	major histocompatibility complex, class I, A	C1-set,MHC_I	alpha-helical transmembrane proteins
	1A36_HUMAN	major histocompatibility complex, class I, A	C1-set,MHC_I	alpha-helical transmembrane proteins
	1A43_HUMAN	major histocompatibility complex, class I, A	C1-set,MHC_I	alpha-helical transmembrane proteins
	1A03_HUMAN	major histocompatibility complex, class I, A	C1-set,MHC_I	alpha-helical transmembrane proteins
	1A34_HUMAN	major histocompatibility complex, class I, A	C1-set,MHC_I	alpha-helical transmembrane proteins
	1A02_HUMAN	major histocompatibility complex, class I, A	C1-set,MHC_I	alpha-helical transmembrane proteins
	1A29_HUMAN	major histocompatibility complex, class I, A	C1-set,MHC_I	alpha-helical transmembrane proteins
	1A23_HUMAN	major histocompatibility complex, class I, A	C1-set,MHC_I	alpha-helical transmembrane proteins
	1A24_HUMAN	major histocompatibility complex, class I, A	C1-set,MHC_I	alpha-helical transmembrane proteins
	1A11_HUMAN	major histocompatibility complex, class I, A	C1-set,MHC_I	alpha-helical transmembrane proteins
	1A01_HUMAN	major histocompatibility complex, class I, A	C1-set,MHC_I	alpha-helical transmembrane proteins
	1A69_HUMAN	major histocompatibility complex, class I, A	C1-set,MHC_I	alpha-helical transmembrane proteins
	MA1A1_HUMAN	mannosidase, alpha, class 1A, member 1	Glyco_hydro_47	alpha-helical transmembrane proteins
	Q6P052_HUMAN	mannosidase, alpha, class 1A, member 1	Glyco_hydro_47	N/A
	MA1A2_HUMAN	mannosidase, alpha, class 1A, member 2	Glyco_hydro_47	alpha-helical transmembrane proteins
	MA1C1_HUMAN	mannosidase, alpha, class 1C, member 1	Glyco_hydro_47	alpha-helical transmembrane proteins
	MA2A1_HUMAN	mannosidase, alpha, class 2A, member 1	Glyco_hydro_38,Alpha-mann_mid,Glyco_hydro_38C	alpha-helical transmembrane proteins
	MA2A2_HUMAN	mannosidase, alpha, class 2A, member 2	Glyco_hydro_38,Alpha-mann_mid,Glyco_hydro_38C	alpha-helical transmembrane proteins
	MGAT1_HUMAN	mannosyl (alpha-1,3-)-glycoprotein beta-1,2-N-acetylglucosaminyltransferase	GNT-I	alpha-helical transmembrane proteins
	PCSK7_HUMAN	proprotein convertase subtilisin/kexin type 7 pseudogene; proprotein convertase subtilisin/kexin type 7	P_proprotein,Peptidase_S8	alpha-helical transmembrane proteins
	QSOX1_HUMAN	quiescin Q6 sulfhydryl oxidase 1	Evr1_Alr,Thioredoxin	alpha-helical transmembrane proteins
	SCAM2_HUMAN	secretory carrier membrane protein 2	SCAMP	alpha-helical transmembrane proteins
	SCAM3_HUMAN	secretory carrier membrane protein 3	SCAMP	alpha-helical transmembrane proteins
	SCAM5_HUMAN	secretory carrier membrane protein 5	SCAMP	alpha-helical transmembrane proteins
	ACER2_HUMAN	similar to alkaline ceramidase 2; alkaline ceramidase 2	Ceramidase	alpha-helical transmembrane proteins
	STEA2_HUMAN	six transmembrane epithelial antigen of the prostate 2	F420_oxidored	alpha-helical transmembrane proteins
	S35A1_HUMAN	solute carrier family 35 (CMP-sialic acid transporter), member A1	Nuc_sug_transp	alpha-helical transmembrane proteins
	Q5W1L7_HUMAN	solute carrier family 35 (CMP-sialic acid transporter), member A1	Nuc_sug_transp	N/A
	S35A3_HUMAN	solute carrier family 35 (UDP-N-acetylglucosamine (UDP-GlcNAc) transporter), member A3	Nuc_sug_transp	alpha-helical transmembrane proteins

	S35A2_HUMAN	solute carrier family 35 (UDP-galactose transporter), member A2	Nuc_sug_transp	alpha-helical transmembrane proteins
	S35A4_HUMAN	solute carrier family 35, member A4	Nuc_sug_transp	alpha-helical transmembrane proteins
	S35A5_HUMAN	solute carrier family 35, member A5	Nuc_sug_transp	alpha-helical transmembrane proteins
	STX10_HUMAN	syntaxin 10	SNARE,Syntaxin-6_N	alpha-helical transmembrane proteins
	STX5_HUMAN	syntaxin 5	Syntaxin,SNARE	alpha-helical transmembrane proteins
	VAMP4_HUMAN	vesicle-associated membrane protein 4	Synaptobrevin	alpha-helical transmembrane proteins
	VAMP7_HUMAN	vesicle-associated membrane protein 7	Synaptobrevin,Longin	alpha-helical transmembrane proteins
	VAMP8_HUMAN	vesicle-associated membrane protein 8 (endobrevin)	Synaptobrevin	alpha-helical transmembrane proteins
	AKAP1_HUMAN	A kinase (PRKA) anchor protein 1	TUDOR,KH_1	N/A
	AFG32_HUMAN	AFG3 ATPase family gene 3-like 2 (yeast)	Peptidase_M41,AAA	alpha-helical transmembrane proteins
	Q5QNZ2_HUMAN	ATP synthase, H+ transporting, mitochondrial F0 complex, subunit B1	Mt_ATP-synt_B	N/A
	ATPK_HUMAN	ATP synthase, H+ transporting, mitochondrial F0 complex, subunit F2	WRW	alpha-helical transmembrane proteins
	ABCBA_HUMAN	ATP-binding cassette, sub-family B (MDR/TAP), member 10	ABC_tran	alpha-helical transmembrane proteins
	ABCB6_HUMAN	ATP-binding cassette, sub-family B (MDR/TAP), member 6	ABC_tran	alpha-helical transmembrane proteins
	ABCB7_HUMAN	ATP-binding cassette, sub-family B (MDR/TAP), member 7	ABC_tran	alpha-helical transmembrane proteins
	ABCB8_HUMAN	ATP-binding cassette, sub-family B (MDR/TAP), member 8	ABC_tran	alpha-helical transmembrane proteins
	BCL2_HUMAN	B-cell CLL/lymphoma 2	Bcl-2,BH4	alpha-helical transmembrane proteins
	BAK_HUMAN	BCL2-antagonist/killer 1; BCL2-like 7 pseudogene 1	Bcl-2,KRAB,zf-H2C2_2	alpha-helical transmembrane proteins
	BAX_HUMAN	BCL2-associated X protein	Bcl-2	alpha-helical transmembrane proteins
	B2CL1_HUMAN	BCL2-like 1	Bcl-2,BH4	alpha-helical transmembrane proteins
	B2CL2_HUMAN	BCL2-like 2	Bcl-2,BH4	N/A
	BNIP3_HUMAN	BCL2/adenovirus E1B 19kDa interacting protein 3	BNIP3	alpha-helical transmembrane proteins
	CISD1_HUMAN	CDGSH iron sulfur domain 1	MitoNEET_N,zf-CDGSH	alpha-helical transmembrane proteins
	B4DI26_HUMAN	COX11 homolog, cytochrome c oxidase assembly protein (yeast)	CtaG_Cox11	N/A
	COX11_HUMAN	COX11 homolog, cytochrome c oxidase assembly protein (yeast)	CtaG_Cox11	alpha-helical transmembrane proteins
	GIMA5_HUMAN	GTPase, IMAP family member 5	AIG1	alpha-helical transmembrane proteins
	MPV17_HUMAN	MpV17 mitochondrial inner membrane protein	Mpv17_PMP22	alpha-helical transmembrane proteins
	NDUAB_HUMAN	NADH dehydrogenase (ubiquinone) 1 alpha subcomplex, 11, 14.7kDa	Tim17	alpha-helical transmembrane proteins
	NDUAD_HUMAN	NADH dehydrogenase (ubiquinone) 1 alpha subcomplex, 13	GRIM-19	alpha-helical transmembrane proteins
	NDUB3_HUMAN	NADH dehydrogenase (ubiquinone) 1 beta subcomplex, 3, 12kDa	NDUF_B12	alpha-helical transmembrane proteins
	NDUB5_HUMAN	NADH dehydrogenase (ubiquinone) 1 beta subcomplex, 5, 16kDa	NDUF_B5	alpha-helical transmembrane proteins
	NDUB8_HUMAN	NADH dehydrogenase (ubiquinone) 1 beta subcomplex, 8, 19kDa	NDUF_B8	alpha-helical transmembrane proteins
	NDUC2_HUMAN	NADH dehydrogenase (ubiquinone) 1, subcomplex unknown, 2, 14.5kDa	NDUF_C2	alpha-helical transmembrane proteins
	ACSL1_HUMAN	acyl-CoA synthetase long-chain family member 1	AMP-binding	alpha-helical transmembrane proteins
	ACSL3_HUMAN	acyl-CoA synthetase long-chain family member 3	AMP-binding	alpha-helical transmembrane proteins
	ACSL4_HUMAN	acyl-CoA synthetase long-chain family member 4	AMP-binding	alpha-helical transmembrane proteins
	ACSL5_HUMAN	acyl-CoA synthetase long-chain family member 5	AMP-binding	alpha-helical transmembrane proteins
	ACSL6_HUMAN	acyl-CoA synthetase long-chain family member 6	AMP-binding	alpha-helical transmembrane proteins
	AL3A2_HUMAN	aldehyde dehydrogenase 3 family, member A2	Aldedh	alpha-helical transmembrane proteins
	AIFM2_HUMAN	apoptosis-inducing factor, mitochondrion-associated, 2	Pyr_redox_2,Pyr_redox	alpha-helical transmembrane proteins
	CRLS1_HUMAN	cardiolipin synthase 1	CDP-OH_P_transf	alpha-helical transmembrane proteins
	CPT1A_HUMAN	carnitine palmitoyltransferase 1A (liver)	Carn_acyltransf	alpha-helical transmembrane proteins
	CPT1C_HUMAN	carnitine palmitoyltransferase 1C	Carn_acyltransf	alpha-helical transmembrane proteins
	CPT1B_HUMAN	choline kinase beta; carnitine palmitoyltransferase 1B (muscle)	Carn_acyltransf	alpha-helical transmembrane proteins
	CYB5_HUMAN	cytochrome b5 type A (microsomal)	Cyt-b5	alpha-helical transmembrane proteins
	CYB5B_HUMAN	cytochrome b5 type B (outer mitochondrial membrane)	Cyt-b5	alpha-helical transmembrane proteins
	COX41_HUMAN	cytochrome c oxidase subunit IV isoform 1	COX4	N/A
	COX42_HUMAN	cytochrome c oxidase subunit IV isoform 2 (lung)	COX4	N/A
	CX7A1_HUMAN	cytochrome c oxidase subunit VIIa polypeptide 1 (muscle)	COX7a	alpha-helical transmembrane proteins
	CX7A2_HUMAN	cytochrome c oxidase subunit VIIa polypeptide 2 (liver)	COX7a	N/A
	COX7R_HUMAN	cytochrome c oxidase subunit VIIa polypeptide 2 like	COX7a	N/A
	CX6A1_HUMAN	cytochrome c oxidase subunit VIa polypeptide 1	COX6A	N/A
	PYRD_HUMAN	dihydroorotate dehydrogenase	DHO_dh	alpha-helical transmembrane proteins
	EXOG_HUMAN	endo/exonuclease (5'-3'), endonuclease G-like	Endonuclease_NS	N/A
	GPDM_HUMAN	glycerol-3-phosphate dehydrogenase 2 (mitochondrial)	EF_hand_5,DAO	N/A
	Q5TDG2_HUMAN	hydroxy-delta-5-steroid dehydrogenase, 3 beta- and steroid delta-isomerase 1	3Beta_HSD	N/A
	3BHS1_HUMAN	hydroxy-delta-5-steroid dehydrogenase, 3 beta- and steroid delta-isomerase 1	3Beta_HSD	alpha-helical transmembrane proteins
	3BHS2_HUMAN	hydroxy-delta-5-steroid dehydrogenase, 3 beta- and steroid delta-isomerase 2	3Beta_HSD	alpha-helical transmembrane proteins
	NDUB4_HUMAN	hypothetical gene supported by AF044957; NM_004547; NADH dehydrogenase (ubiquinone) 1 beta subcomplex, 4, 15kDa	NDUF_B4	alpha-helical transmembrane proteins
	KMO_HUMAN	kynurenine 3-monooxygenase (kynurenine 3-hydroxylase)	FAD_binding_3	alpha-helical transmembrane proteins
	LETM1_HUMAN	leucine zipper-EF-hand containing transmembrane protein 1	LETM1	alpha-helical transmembrane proteins
	LETM2_HUMAN	leucine zipper-EF-hand containing transmembrane protein 2	LETM1	alpha-helical transmembrane proteins
mitochondrial membrane	MARH5_HUMAN	membrane-associated ring finger (C3HC4) 5	RINGv	alpha-helical transmembrane proteins
	MTX1_HUMAN	metaxin 1	Tom37,Tom37_C	alpha-helical transmembrane proteins
	MGST1_HUMAN	microsomal glutathione S-transferase 1	MAPEG	alpha-helical transmembrane proteins
	MUL1_HUMAN	mitochondrial E3 ubiquitin ligase 1	zf-C3HC4_3	alpha-helical transmembrane proteins
	MTCH1_HUMAN	mitochondrial carrier homolog 1 (C. elegans)	Mito_carr	alpha-helical transmembrane proteins
	MFF_HUMAN	mitochondrial fission factor	Miff	alpha-helical transmembrane proteins
	MFN2_HUMAN	mitofusin 2	Dynamin_N,Fzo_mitofusin	alpha-helical transmembrane proteins
	AOFB_HUMAN	monoamine oxidase B	Amino_oxidase	alpha-helical transmembrane proteins
	MCL1_HUMAN	myeloid cell leukemia sequence 1 (BCL2-related)	Bcl-2,BACK,BTB	alpha-helical transmembrane proteins
	NNTM_HUMAN	nicotinamide nucleotide transhydrogenase	AlaDh_PNT_N,AlaDh_PNT_C	alpha-helical transmembrane proteins
	STOM_HUMAN	phosphatidylethanolamine binding protein 1	Band_7	N/A
	PSN1_HUMAN	presenilin 1	Presenilin	alpha-helical transmembrane proteins
	PARL_HUMAN	presenilin associated, rhomboid-like	Rhomboid	alpha-helical transmembrane proteins
	MIRO1_HUMAN	ras homolog gene family, member T1	Miro,Ras	alpha-helical transmembrane proteins
	MIRO2_HUMAN	ras homolog gene family, member T2	Miro,Ras	alpha-helical transmembrane proteins
	SFXN1_HUMAN	sideroflexin 1	Mtc	alpha-helical transmembrane proteins
	SFXN2_HUMAN	sideroflexin 2	Mtc	alpha-helical transmembrane proteins
	SFXN5_HUMAN	sideroflexin 5	Mtc	alpha-helical transmembrane proteins
	TIM23_HUMAN	similar to translocase of inner mitochondrial membrane 23 (yeast) homolog	Tim17	alpha-helical transmembrane proteins
	TIM14_HUMAN	similar to translocase of the inner mitochondrial membrane 14; DnaJ (Hsp40) homolog, subfamily C, member 19	DnaJ	alpha-helical transmembrane proteins
	EAA1_HUMAN	solute carrier family 1 (glial high affinity glutamate transporter), member 3	SDF	alpha-helical transmembrane proteins
	MCAT_HUMAN	solute carrier family 25 (carnitine/acylcarnitine translocase), member 20	Mito_carr	alpha-helical transmembrane proteins
	GHC2_HUMAN	solute carrier family 25 (mitochondrial carrier), member 18	Mito_carr	alpha-helical transmembrane proteins
	GHC1_HUMAN	solute carrier family 25 (mitochondrial carrier: glutamate), member 22	Mito_carr	alpha-helical transmembrane proteins
	ADT4_HUMAN	solute carrier family 25 (mitochondrial carrier; adenine nucleotide translocator), member 31	Mito_carr	alpha-helical transmembrane proteins
	ADT1_HUMAN	solute carrier family 25 (mitochondrial carrier; adenine nucleotide translocator), member 4	Mito_carr	alpha-helical transmembrane proteins
	ADT2_HUMAN	solute carrier family 25 (mitochondrial carrier; adenine nucleotide translocator), member 5; solute carrier family 25 (mitochondrial carrier; adenine nucleotide translocator), member 5 pseudogene 8	Mito_carr	alpha-helical transmembrane proteins
	ADT3_HUMAN	solute carrier family 25 (mitochondrial carrier; adenine nucleotide translocator), member 6	Mito_carr	alpha-helical transmembrane proteins
	ORNT2_HUMAN	solute carrier family 25 (mitochondrial carrier; ornithine transporter) member 2	Mito_carr	alpha-helical transmembrane proteins
	PM34_HUMAN	solute carrier family 25 (mitochondrial carrier; peroxisomal membrane protein, 34kDa), member 17	Mito_carr	alpha-helical transmembrane proteins
	MPCP_HUMAN	solute carrier family 25 (mitochondrial carrier; phosphate carrier), member 3	Mito_carr	alpha-helical transmembrane proteins
	SAMC_HUMAN	solute carrier family 25, member 26	Mito_carr	alpha-helical transmembrane proteins
	S2533_HUMAN	solute carrier family 25, member 33	Mito_carr	alpha-helical transmembrane proteins
	S2534_HUMAN	solute carrier family 25, member 34	Mito_carr	alpha-helical transmembrane proteins
	S2536_HUMAN	solute carrier family 25, member 36	Mito_carr	alpha-helical transmembrane proteins
	S2539_HUMAN	solute carrier family 25, member 39	Mito_carr	alpha-helical transmembrane proteins
	S2543_HUMAN	solute carrier family 25, member 43	Mito_carr	alpha-helical transmembrane proteins
	S2544_HUMAN	solute carrier family 25, member 44	Mito_carr	alpha-helical transmembrane proteins
	S2545_HUMAN	solute carrier family 25, member 45	Mito_carr	alpha-helical transmembrane proteins
	SLC31_HUMAN	solute carrier family 3 (cystine, dibasic and neutral amino acid transporters, activator of cystine, dibasic and neutral amino acid transport), member 1	Alpha-amylase	alpha-helical transmembrane proteins
	SPNS1_HUMAN	spinster homolog 1 (Drosophila)	MFS_1	alpha-helical transmembrane proteins
	SYJ2B_HUMAN	synaptojanin 2 binding protein	PDZ	alpha-helical transmembrane proteins
	TI17A_HUMAN	translocase of inner mitochondrial membrane 17 homolog A (yeast)	Tim17	alpha-helical transmembrane proteins
	TI17B_HUMAN	translocase of inner mitochondrial membrane 17 homolog B (yeast)	Tim17	alpha-helical transmembrane proteins
	TIM22_HUMAN	translocase of inner mitochondrial membrane 22 homolog (yeast)	Tim17	alpha-helical transmembrane proteins
	TIM23_HUMAN	translocase of inner mitochondrial membrane 23 homolog (yeast); translocase of inner mitochondrial membrane 23 homolog B (yeast)	Tim17	alpha-helical transmembrane proteins
	TI23B_HUMAN	translocase of inner mitochondrial membrane 23 homolog (yeast); translocase of inner mitochondrial membrane 23 homolog B (yeast)	Tim17	alpha-helical transmembrane proteins
	TIM50_HUMAN	translocase of inner mitochondrial membrane 50 homolog (S. cerevisiae)	NIF	alpha-helical transmembrane proteins

	TOM7_HUMAN	translocase of outer mitochondrial membrane 7 homolog (yeast)	Tom7	alpha-helical transmembrane proteins
	TOM70_HUMAN	translocase of outer mitochondrial membrane 70 homolog A (S. cerevisiae)	TPR_11	alpha-helical transmembrane proteins
	UBP30_HUMAN	ubiquitin specific peptidase 30	UCH	alpha-helical transmembrane proteins
	VAMP1_HUMAN	vesicle-associated membrane protein 1 (synaptobrevin 1)	Synaptobrevin	alpha-helical transmembrane proteins
nuclear membrane	DHCR7_HUMAN	7-dehydrocholesterol reductase	ERG4_ERG24	alpha-helical transmembrane proteins
	AT11B_HUMAN	ATPase, class VI, type 11B	E1-E2_ATPase	alpha-helical transmembrane proteins
	BCL2_HUMAN	B-cell CLL/lymphoma 2	Bcl-2,BH4	alpha-helical transmembrane proteins
	B2L10_HUMAN	BCL2-like 10 (apoptosis facilitator)	Bcl-2	alpha-helical transmembrane proteins
	LEMD2_HUMAN	LEM domain containing 2	MSC,LEM	alpha-helical transmembrane proteins
	MAN1_HUMAN	LEM domain containing 3	Lectin_leg-like,MSC,LEM	alpha-helical transmembrane proteins
	AL5AP_HUMAN	arachidonate 5-lipoxygenase-activating protein	MAPEG	alpha-helical transmembrane proteins
	EMD_HUMAN	emerin	LEM	alpha-helical transmembrane proteins
	ERN1_HUMAN	endoplasmic reticulum to nucleus signaling 1	HECT,Pkinase,Ribonuc_2-5A	alpha-helical transmembrane proteins
	GHRHR_HUMAN	growth hormone releasing hormone receptor	HRM	alpha-helical transmembrane proteins
	GUC2D_HUMAN	guanylate cyclase 2D, membrane (retina-specific)	Pkinase_Tyr,HNOBA,ANF_receptor,Guanylate_cyc	alpha-helical transmembrane proteins
	GUC2F_HUMAN	guanylate cyclase 2F, retinal	Pkinase_Tyr,HNOBA,ANF_receptor,Guanylate_cyc	alpha-helical transmembrane proteins
	ITPR1_HUMAN	inositol 1,4,5-triphosphate receptor, type 1	RIH_assoc,RYDR_ITPR,MIR,Ins145_P3_rec	alpha-helical transmembrane proteins
	ITPR3_HUMAN	inositol 1,4,5-triphosphate receptor, type 3	RIH_assoc,RYDR_ITPR,MIR,Ins145_P3_rec	alpha-helical transmembrane proteins
	LBR_HUMAN	lamin B receptor	ERG4_ERG24,LBR_tudor	alpha-helical transmembrane proteins
	NUCB2_HUMAN	nucleobindin 2	EF_hand_5	N/A
	PSN1_HUMAN	presenilin 1	Presenilin	alpha-helical transmembrane proteins
	P2RX7_HUMAN	purinergic receptor P2X, ligand-gated ion channel, 7	P2X_receptor	alpha-helical transmembrane proteins
	RETST_HUMAN	retinol saturase (all-trans-retinol 13,14-reductase)	NAD_binding_8	N/A
	SHSA5_HUMAN	shisa homolog 5 (Xenopus laevis)	Shisa	alpha-helical transmembrane proteins
	ACOD_HUMAN	stearoyl-CoA desaturase (delta-9-desaturase)	FA_desaturase	alpha-helical transmembrane proteins
	LAP2B_HUMAN	thymopoietin	Thymopoietin,LEM	alpha-helical transmembrane proteins
	TM38A_HUMAN	transmembrane protein 38A	TRIC	alpha-helical transmembrane proteins
	TM38B_HUMAN	transmembrane protein 38B	TRIC	alpha-helical transmembrane proteins
